# Computational design of metallohydrolases

**DOI:** 10.1038/s41586-025-09746-w

**Published:** 2025-12-03

**Authors:** Donghyo Kim, Seth M. Woodbury, Woody Ahern, Doug Tischer, Alex Kang, Emily Joyce, Asim K. Bera, Nikita Hanikel, Saman Salike, Rohith Krishna, Jason Yim, Samuel J. Pellock, Anna Lauko, Indrek Kalvet, Donald Hilvert, David Baker

**Affiliations:** 1 Department of Biochemistry, University of Washington, Seattle, WA USA; 2 Institute for Protein Design, University of Washington, Seattle, WA USA; 3 Graduate Program in Bioengineering, University of Washington, Seattle, WA USA; 4 Paul G. Allen School of Computer Science and Engineering, University of Washington, Seattle, WA USA; 5 Graduate Program in Chemical Engineering, University of Washington, Seattle, WA USA; 6 Massachusetts Institute of Technology, Cambridge, MA USA; 7 Graduate Program in Biological Physics, Structure and Design, University of Washington, Seattle, WA USA; 8 Howard Hughes Medical Institute, University of Washington, Seattle, WA USA; 9 Laboratory of Organic Chemistry, ETH Zurich, Zurich, Switzerland

**Keywords:** Protein design, Hydrolases

## Abstract

De novo enzyme design seeks to build proteins containing ideal active sites with catalytic residues surrounding and stabilizing the transition state(s) of the target chemical reaction^1–7^. The generative artificial intelligence method RFdiffusion^8,9^ solves this problem, but requires specifying both the sequence position and backbone coordinates for each catalytic residue, limiting sampling. Here we introduce RFdiffusion2, which eliminates these requirements, and use it to design zinc metallohydrolases starting from quantum chemistry-derived active site geometries. From an initial set of 96 designs tested experimentally, the most active has a catalytic efficiency (*k*_cat_/*K*_M_) of 16,000 M^−1^ s^−1^, orders of magnitude higher than previously designed metallohydrolases^6,7,10,11^. A second round of 96 designs yielded 3 additional highly active enzymes, with *k*_cat_/*K*_M_ values of up to 53,000 M^−1^ s^−1^ and a catalytic rate constant (*k*_cat_) of up to 1.5 s^−1^. The design models of the four most active designs differ from known structures and from each other, and the crystal structure of the most active design is very close to the design model, demonstrating the accuracy of the design method. The most active enzymes are predicted by PLACER^12^ and Chai-1 (ref. ^13^) to have preorganized active sites that effectively position the substrate for nucleophilic attack by a water molecule activated by the bound metal. The ability to generate highly active enzymes directly from the computer, without experimental optimization, should enable a new generation of potent designer catalysts^14,15^.

## Abstract (web summary)

A generative artificial intelligence-powered method enables de novo design of highly active enzymes based on information about the geometry of residues in the active site, without requiring protein backbone or sequence information.

## Main

Metallohydrolases catalyse some of the most difficult hydrolysis reactions in biology by using their bound metal ions to activate a water molecule positioned adjacent to the substrate bond to be cleaved^16–18^. Engineering new metallohydrolases is currently of considerable interest for degrading human-generated environmental pollutants, for which there has not been sufficient time for efficient hydrolytic enzymes to evolve^19–21^. Protein engineering has expanded the scope of substrates that can be hydrolysed by metallohydrolases, but this often requires initial promiscuous activity^22,23^. De novo enzyme design has been used to generate new metallohydrolases^6,10,24^, but these have had relatively low activity and efficiency, and have required extensive directed evolution to match the activity and efficiency of native enzymes^24^. Given an ideal metallohydrolase active site, de novo enzyme design seeks to identify or generate a protein scaffold that positions the catalytic residues, metals, and substrates in optimal catalytic geometries with high accuracy^25,26^. RFdiffusion has been used successfully to scaffold active sites, but the search has been limited by the need to specify the sequence positions and conformations of the catalytic residues^8,9,27^.

We reasoned that a generative artificial intelligence design method that only required the specification of side-chain functional group positions around a reaction transition state, and was capable of sampling over all possible sequence positions and conformations of these residues, could more readily satisfy complex catalytic constraints^14,15,28,29^. We set out to develop such an approach, and used it to design new metallohydrolases starting from a quantum chemistry-generated active site description with a bound metal cofactor.

To enable sequence-position and side-chain rotamer-agnostic enzyme design, we developed a generative artificial intelligence flow-matching model called RFdiffusion2^30^. RFdiffusion2 extends the capabilities of RFdiffusion to generate scaffolds that position a set of functional residues (a ‘motif’) in two key ways. First, it enables atomic substructure scaffolding: RFdiffusion can only scaffold backbone-level motifs (with the side-chain and backbone atom N-Cα-C=O positions specified), whereas RFdiffusion2 can scaffold arbitrary atom-level motifs (any subset of amino acid heavy atoms). This is important for enzyme design because it allows users to specify only the positions of the key functional groups that interact with the reaction transition state, rather than the full side-chain and backbone conformation. Second, RFdiffusion2 enables sequence-position-agnostic scaffolding: RFdiffusion requires specification of the primary sequence positions of the motif residues, but RFdiffusion2 can scaffold motifs whose primary sequence positions are unknown. RFdiffusion2 replaces diffusion with flow matching^31,32^ and achieves sequence-position-agnostic atomic substructure scaffolding by providing randomly selected native atomic coordinates (but not their sequence positions) during training in addition to the partially noised, sequence-labelled atomic coordinates. With these improvements, RFdiffusion2 generates diverse proteins starting directly from catalytic configurations that consist of input functional group positions and substrate coordinates. Allowing the model to resolve the a priori unknown degrees of freedom (that is, the primary sequence positions and side-chain rotamer conformations of the catalytic residues) is considerably more effective at generating self-consistent design solutions than randomly sampling those degrees of freedom before inference, because the space is far too large to enumerate, as was necessitated with RFdiffusion. A detailed description of RFdiffusion2 training and benchmarking results for a wide range of active site scaffolding problems is described elsewhere^30^.

**Fig. 1:**
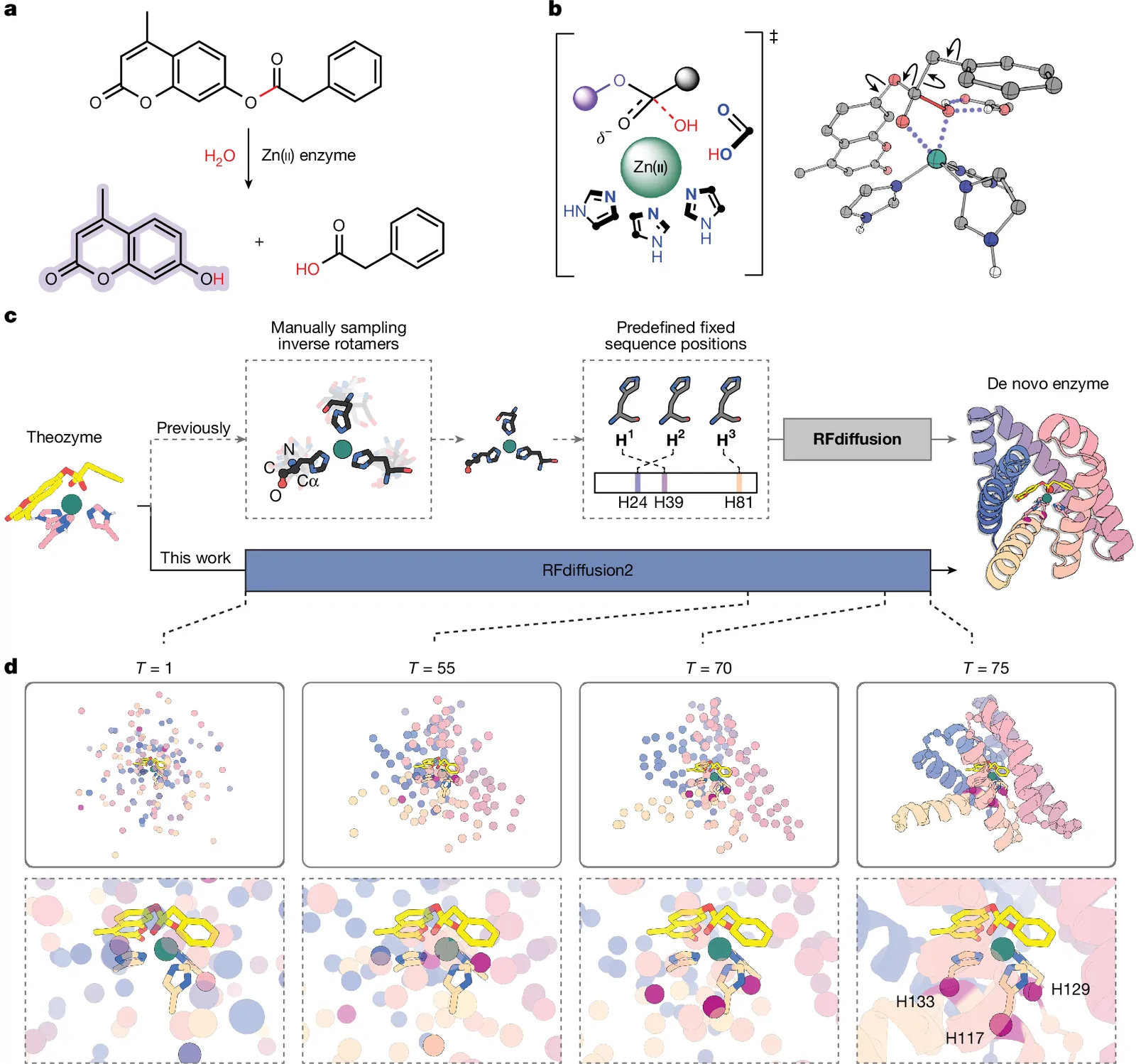
RFdiffusion2 design method. **a**, Hydrolysis of 4MU-PA yields phenylacetic acid and a fluorescent coumarin product. **b**, Example theozyme for Zn(II)-hydroxide nucleophilic attack on the 4MU-PA ester. Two-dimensional representation (left) and 3D DFT model (right). Arrows on the 3D model represent sampled conformational flexibility. **c**, Comparison of scaffold generation around an input theozyme using previous backbone centric RFdiffusion (top row) versus interaction functional group centric RFdiffusion2. RFdiffusion requires explicit upfront sampling of side-chain conformations and residue sequence positions, whereas RFdiffusion2 only requires the transition-state complex and the catalytic side-chain functional groups, implicitly sampling sequence space and rotameric space during inference. **d**, Snapshots of the global structure and active site from model X_T_ during an RFdiffusion2 inference trajectory. The coordinates of the input transition-state complex and catalytic functional groups stay fixed during inference while the backbone structure, sequence positions, and unspecified atoms of the catalytic side chains are sampled by RFdiffusion2. The Cα atoms that host the catalytic histidines at the end of the trajectory are retrospectively highlighted as red spheres; these Cα atoms are not predetermined but rather move into the frame to host the fixed side chains as the global structure forms around the motifs.

**Extended Data Fig. 1:**
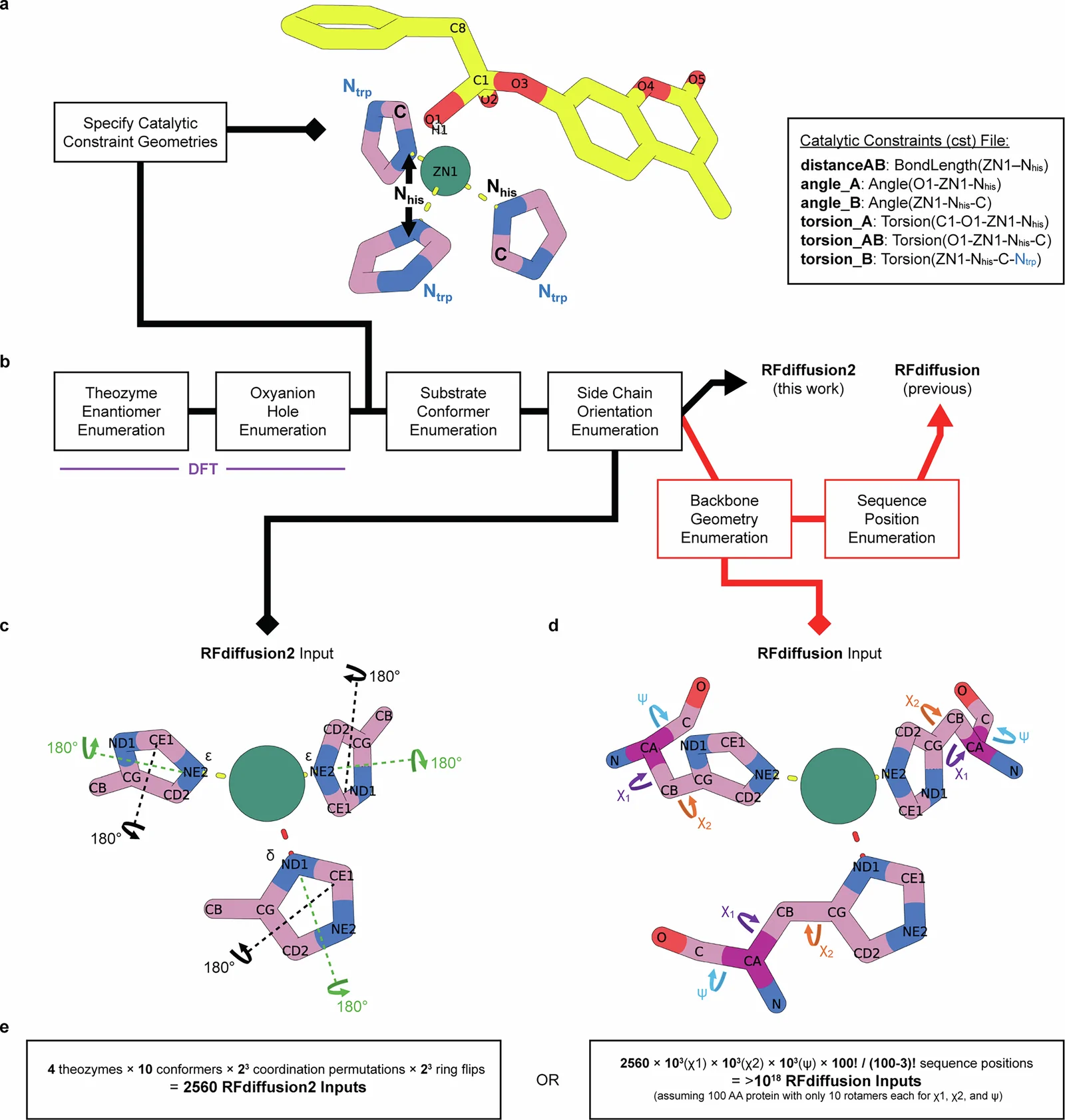
Catalytic constraints and enumerative catalytic motif scaffolding around theozymes for RFdiffusion2 compared to RFdiffusion. **a**, Example theozyme from DFT with relevant ligand atoms labeled and Rosetta constraint file labeling for the histidine side chains. Catalytic constraint files specifying the coordination geometry of the histidines were created at this step for each of the 4 theozymes from DFT. Note that N_his_ is a general Rosetta label that accommodates epsilon and delta coordination geometries with zinc. **b**, Theozyme sampling enumeration pipeline with diverging paths for RFdiffusion2 and RFdiffusion. **c**, Side chain enumeration sampling different combinations of zinc coordination and imidazole orientations. Note that there are only 64 permutations of coordination and Cβ positioning. **d**, Backbone enumeration for RFdiffusion. This is required in addition to the sampling in (**c**). **e**, Calculation of the total number of possible RFdiffusion2 and RFdiffusion inputs. It should be noted that not all combinations of rotamers and sequence positions will be viable, somewhat reducing the total number, but to nowhere near the tiny enumeration space needed for RFdiffusion2.

As an initial test of RFdiffusion2, we chose to design a zinc metallohydrolase for the hydrolysis of a fluorogenic ester, 4-methylumbelliferyl phenylacetate (4MU-PA), as a target reaction (Fig. 1a). We began by using density functional theory (DFT) to identify the transition-state geometry of the rate-determining Zn(II)-OH nucleophilic attack on the substrate ester. Four distinct catalytic arrangements based on the stereochemistry of the tetrahedral intermediate and the nature of the oxyanion hole were considered (Fig. 1b, Supplementary Figs. 1 and 2, Supplementary Data 1 and Supplementary Methods 4.1). These calculations provide the coordinates of the three Zn(II)-binding imidazole rings, the metal, and the transition state. Our previous RFdiffusion approach required the backbone coordinates and residue positions as inputs, which would require upfront sampling of the rotameric states and sequence position for each histidine. This cannot be done exhaustively: even with relatively coarse sampling around the side-chain chi angles *χ*_1_, *χ*_2_, and the backbone torsion angle *ψ*, there are on the order of 10^18^ possible choices for the side-chain conformations and sequence placements of our full catalytic site (Fig. 1c and Extended Data Fig. 1). Whereas each RFdiffusion run has to be initialized with a specific (and generally randomly selected) choice from this enormous set of combinations, RFdiffusion2 as described above searches the entire space in each trajectory.

**Extended Data Fig. 2:**
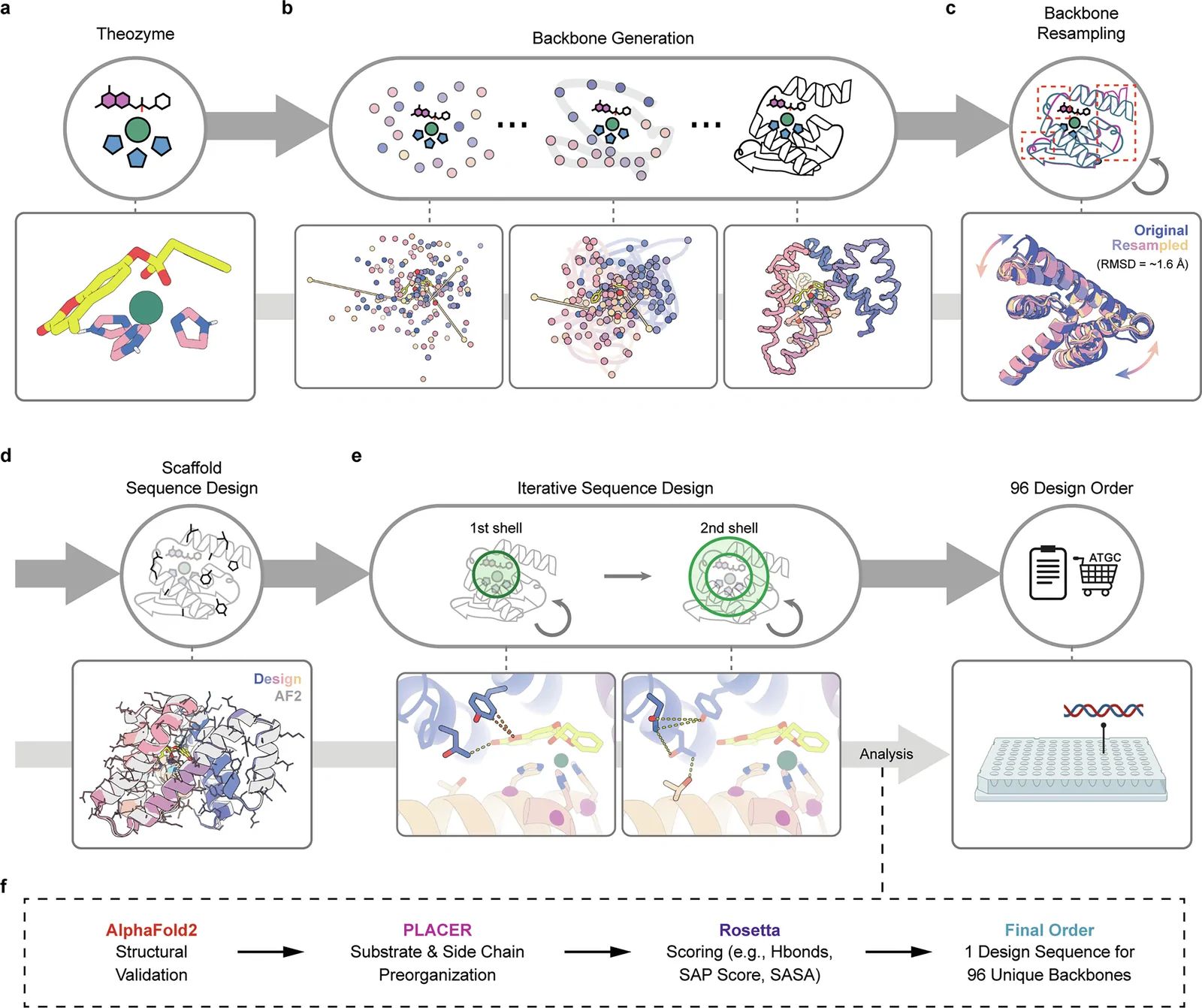
Computational design pipeline for metallohydrolases. **a-f**, Visual illustrations of the key steps in the computational design pipeline, with examples showing A1 (ZETA_1) at each stage in the pipeline.

RFdiffusion2 inference trajectories were used to build protein scaffolds housing the DFT-generated minimal active site configurations, referred to as theozymes^2,33^. Several snapshots from a representative trajectory are shown in Fig. 1d, transforming random noise on the left into the final backbone on the right (Supplementary Video 1). The Cα atoms of each residue (shown as coloured spheres representing final sequence position) are initially sampled from a Gaussian distribution, and the target functional atom positions (shown in sticks) stay fixed. As the trajectory proceeds from left to right, the global structure takes shape around the motif, with the fixed histidine side chains eventually connecting to Cα atoms of the protein backbone at sequence positions of the network’s choosing. A total of 5,120 RFdiffusion2 inference trajectories were carried out starting from different random seeds and for each of the resulting protein scaffolds, sequences were generated using ProteinMPNN^34^. The catalytic geometry and interactions with the transition state of those designs for which the AlphaFold2^35^ predicted structure was close to the design model were further optimized using iterative LigandMPNN^36^ and constrained Rosetta repacking and minimization^37^ (Extended Data Fig. 2 and Supplementary Methods 4.1). Designs containing a proposed general base positioned to activate the water molecule (that is, Glu, Asp or His within hydrogen bonding distance of the Zn(II)-bound water) and side-chain hydrogen bonds stabilizing the transition-state oxyanion (if applicable), and that AlphaFold2 predicted to adopt the target structure, were characterized with PLACER^12^ to assess active site preorganization. A total of 96 designs were selected for experimental characterization on the basis of predicted active site geometry and preorganization (Supplementary Fig. 3, Supplementary Data 2 and 3 and Supplementary Methods 4.1).

**Fig. 2:**
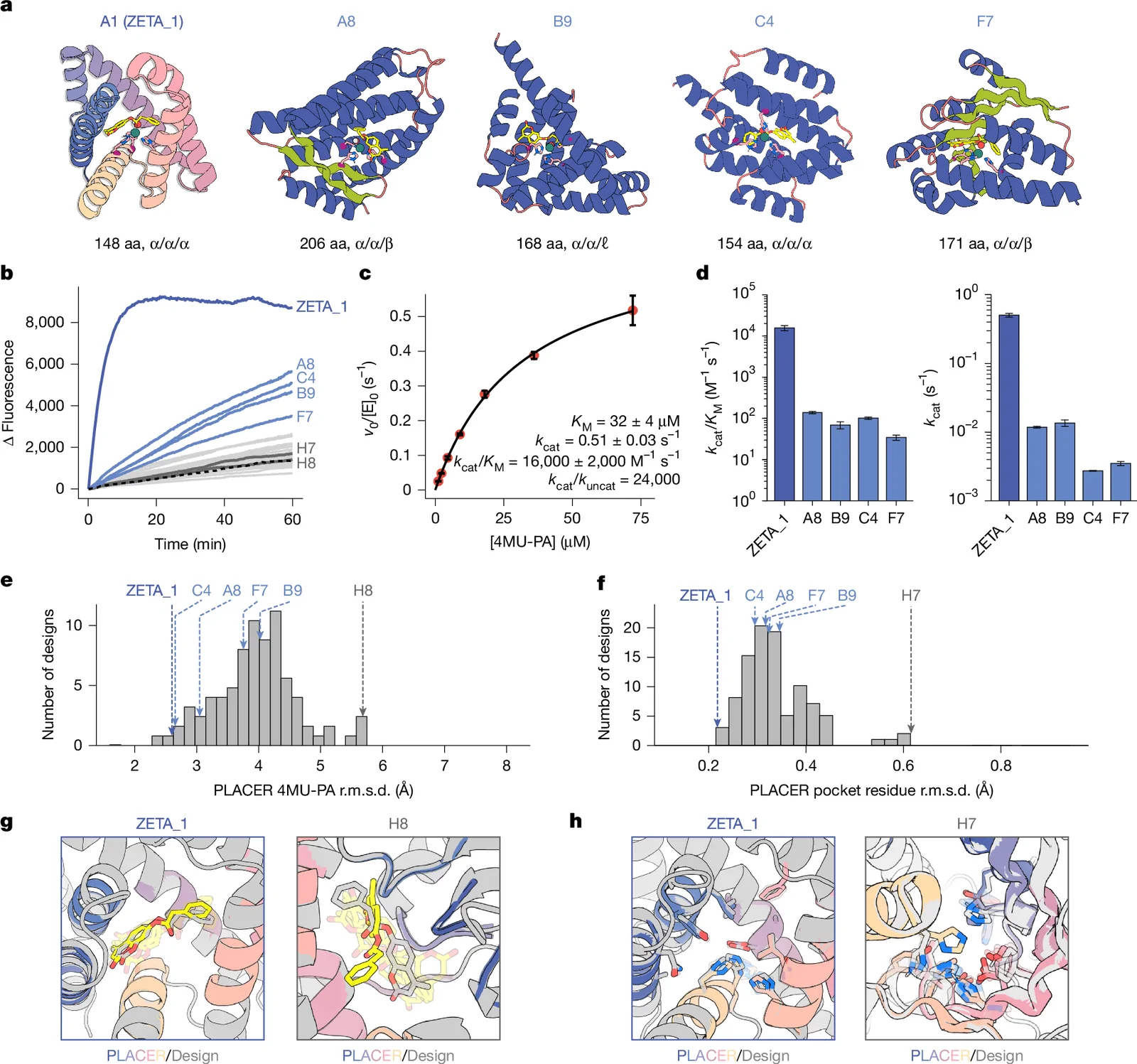
Activity characterization and PLACER preorganization assessment. **a**, Design models of the most active designs. Sequence length (in amino acids (aa)) and the secondary structure harbouring each catalytic histidine are indicated below. **b**, Reaction progress curves. The dashed black line is the buffer background. **c**, Michaelis–Menten characterization of A1 (ZETA_1). The *y* axis shows the initial rate *v*_0_ divided by the total enzyme concentration ([E]_0_). **d**, Michaelis–Menten parameters of most active designs. **e**,**f**, Distribution of PLACER active site preorganization ensemble metrics for the ordered designs. Average design-prediction substrate r.m.s.d. (**e**) and average catalytic and binding residue design-prediction r.m.s.d. (**f**) across all predicted ensembles generated by PLACER for each design. **g**, For ZETA_1, the substrate position in PLACER ensembles is close to the design model, whereas in the inactive design H7, the substrate position fluctuates widely. **h**, For ZETA_1, the side chains surrounding the active site are largely fixed in positions close to the original design model, whereas in the inactive H8 design, the side-chain positions vary considerably. Note that only the first five randomly generated, unranked ensemble predictions are shown for PLACER in **g**,**h**. Data represent the mean ± s.d. of three independent measurements of initial velocity (**c**) and Michaelis–Menten parameters (**d**).

**Fig. 3:**
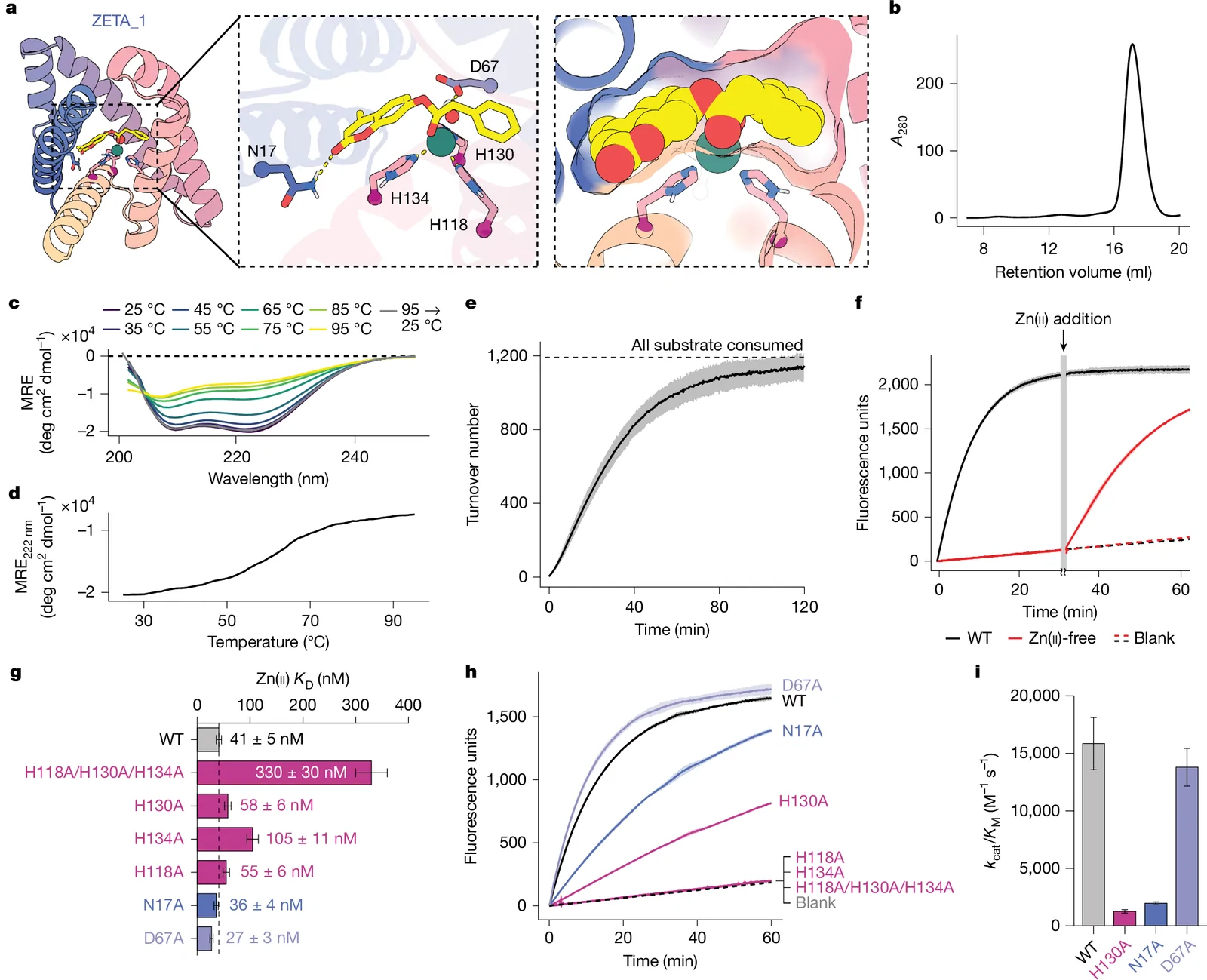
Characterization of ZETA_1 activity. **a**, ZETA_1 design model (left) with close-up view of the active site showing the catalytic residues (middle) and a surface view of the designed pocket revealing high shape-complementarity to the substrate (right). **b**, Size-exclusion chromatogram of ZETA_1 showing a single peak corresponding to monomeric protein. **c**, Circular dichroism spectra of ZETA_1 recorded every 10 °C from 25 °C to 95 °C (viridis colour gradient), and after recooling to 25 °C (grey). The spectra suggest that ZETA_1 has an α-helical secondary structure and that it can refold after heating and partial unfolding. MRE, mean residue ellipticity. **d**, Circular dichroism signal at 222 nm measured every 1 °C and plotted as a function of temperature. **e**, [Product]:[enzyme] progress curve shows that ZETA_1 hydrolyses more than 1,000 4MU-PA molecules per enzyme. Note that the background reaction has been subtracted from the spectra so that every turnover can be attributed to the enzyme (Supplementary Fig. 8; further details in Supplementary Information, section 4.3). **f**, Reaction progress curves for the Zn(II) holo- and zinc-free apo ZETA_1 proteins showing zinc-dependent activity. Adding excess Zn(II) to the apo ZETA_1 sample after 30 min re-establishes the activity, demonstrating that zinc is essential for the catalytic mechanism of ZETA_1. WT, wild type. **g**, Zinc affinity of wild-type and mutant ZETA_1, measured as the dissociation constant (*K*_D_), where a lower value indicates tighter binding. **h**, Fluorescence progress curves comparing the activity of wild-type and mutant ZETA_1. **i**, *k*_cat_/*K*_M_ for active ZETA_1 mutants compared with the wild type. Data represent the mean ± s.d. of three independent measurements of turnover number (**e**), fluorescence progress curves (**f**,**h**), Zn(II)-binding dissociation constant (**g**) and Michaelis–Menten parameters (**i**).

**Extended Data Fig. 3:**
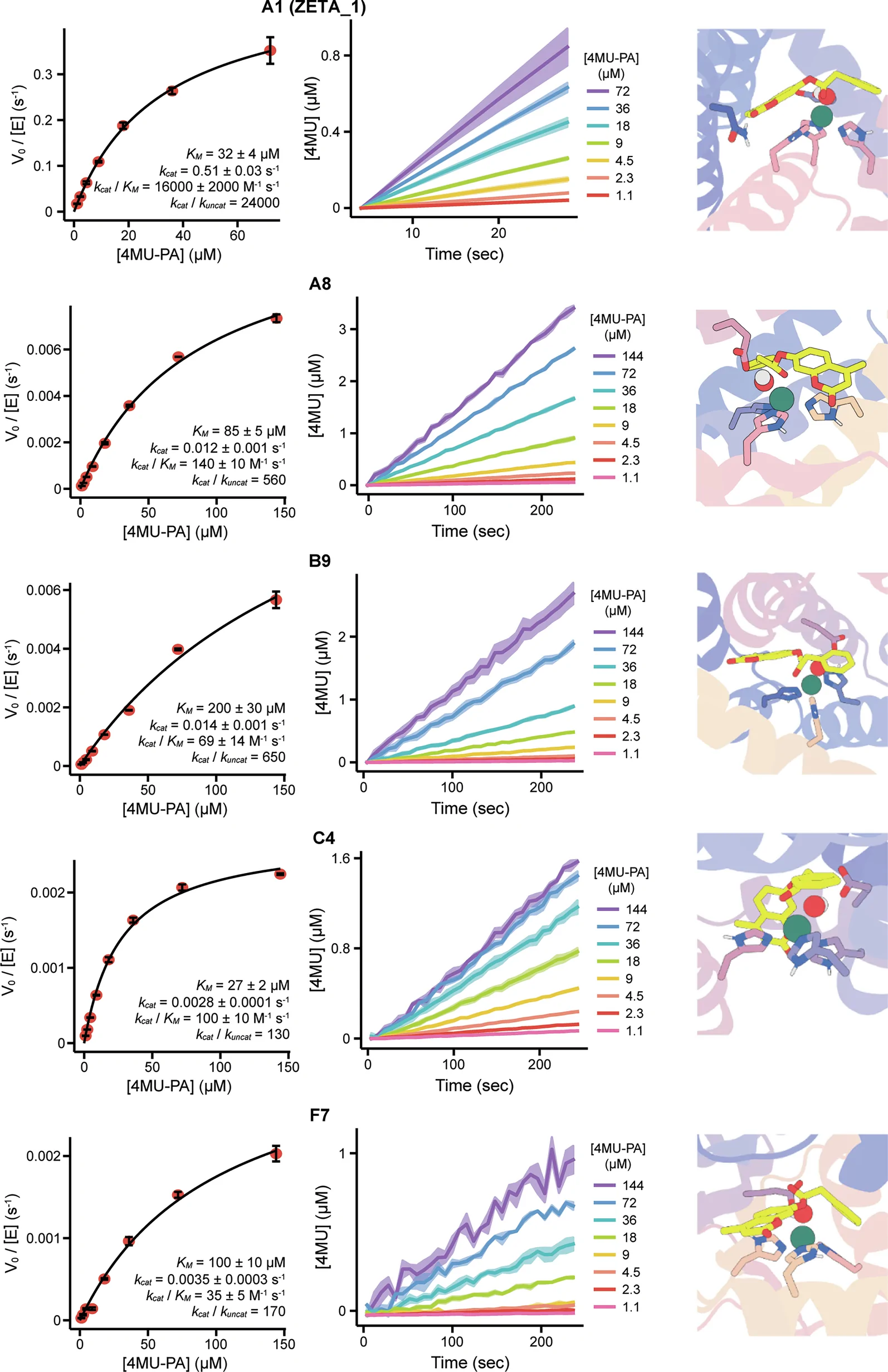
Michaelis-Menten kinetic characterization of the five design hits from the first design campaign. Michaelis-Menten plots from these data are shown on the left. The progress curves for the measured initial velocities are shown in the middle. Designed active sites with the histidines and general base are displayed on the right.

**Extended Data Fig. 4:**
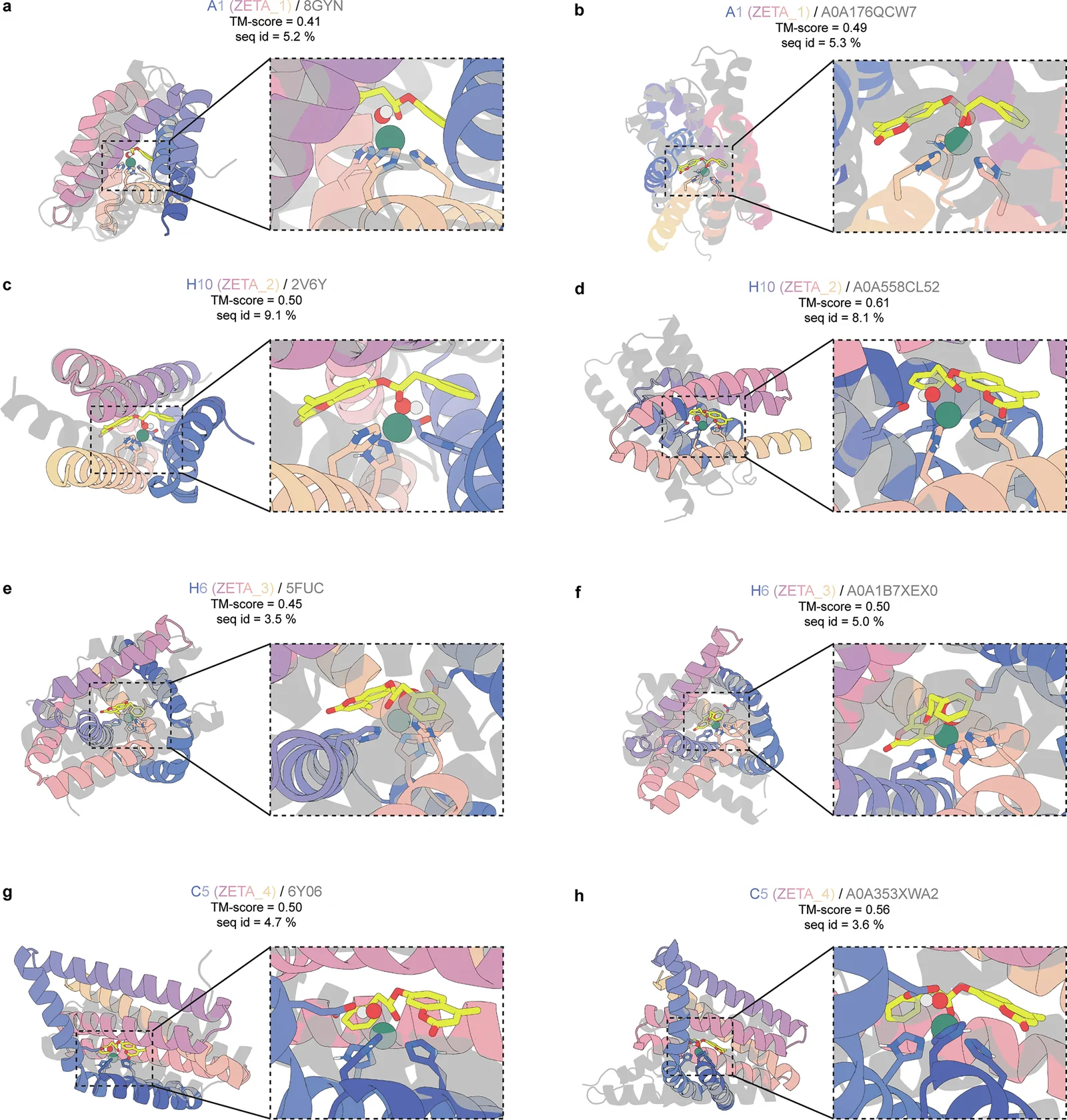
Structural novelty of the ZETA_1-4 designs. Global Cα alignment of the ZETA_1-4 design models with their most similar structures in the Protein Data Bank and AlphaFold2 Database based on TM-score, respectively. **a**,**b**, ZETA_1 aligned with (**a**) PDB structure 8GYM and (**b**) AF2 prediction A0A176QCW7. **c**,**d**, ZETA_2 aligned with (**c**) PDB structure 2V6Y and (**d**) AF2 prediction A0A558CL52. **e**,**f**, ZETA_3 aligned with (**e**) PDB structure 5FUC and (**f**) AF2 prediction A0A1B7XEX0. **g**,**h**, ZETA_4 aligned with (**g**) PDB structure 6Y06 and (**h**) AF2 prediction A0A353XWA2. In all cases, the designed proteins are shown in a colour gradient, and the PDB/AF2 structures are shown in grey. The poor alignment, low sequence identity, and modest TM-scores highlight the novelty of these de novo scaffolds.

Linear DNA fragments encoding the 96 designs were cloned into a plasmid encoding a C-terminal Strep-tag and used to transform *Escherichia coli*, and the resulting proteins were purified using Strep-tag affinity chromatography. Eighty-six out of ninety-six designs were expressed and soluble as judged by SDS–PAGE analysis of the eluants (Supplementary Fig. 4). Purified designs were supplemented with zinc sulfate, and hydrolysis of 4MU-PA was monitored by fluorescence. Five designs (A1, A8, B9, C4 and F7) had activity well above background (Fig. 2b and Supplementary Fig. 5). Sequence-verified single clones for each of these were expressed and purified by affinity chromatography followed by size-exclusion chromatography to obtain pure, monomeric protein fractions (Supplementary Figs. 6 and 7 and Supplementary Table 1). Michaelis–Menten kinetic characterization of the purified variants revealed a *k*_cat_/*K*_M_ of 16,000 ± 2,000 M^−1^ s^−1^ for A1, the most active design, and *k*_cat_/*K*_M_ values in the range of 35–140 M^−1^ s^−1^ for the other four designs (Fig. 2c,d, Extended Data Fig. 3 and Extended Data Table 1). For comparison, the *k*_cat_/*K*_M_ of previously designed metallohydrolases^24^ ranged from 3 to 60 M^−1^ s^−1^ (Supplementary Table 2). A1 is also a relatively robust enzyme, and retains activity for at least 1,000 turnovers (Fig. 3e and Supplementary Fig. 8). A1 differs considerably from previously described proteins: the most similar structures identified through template modelling (TM) alignment with the Protein Data Bank (PDB) and AlphaFold Protein Structure Database (AFDB) have TM scores^38^ of 0.41 and 0.49, respectively, and do not have analogous arrangements of catalytic residues (Extended Data Fig. 4a,b). We refer to A1 as zinc metalloesterase 1 (ZETA_1) throughout the remainder of the text.

**Extended Data Fig. 5:**
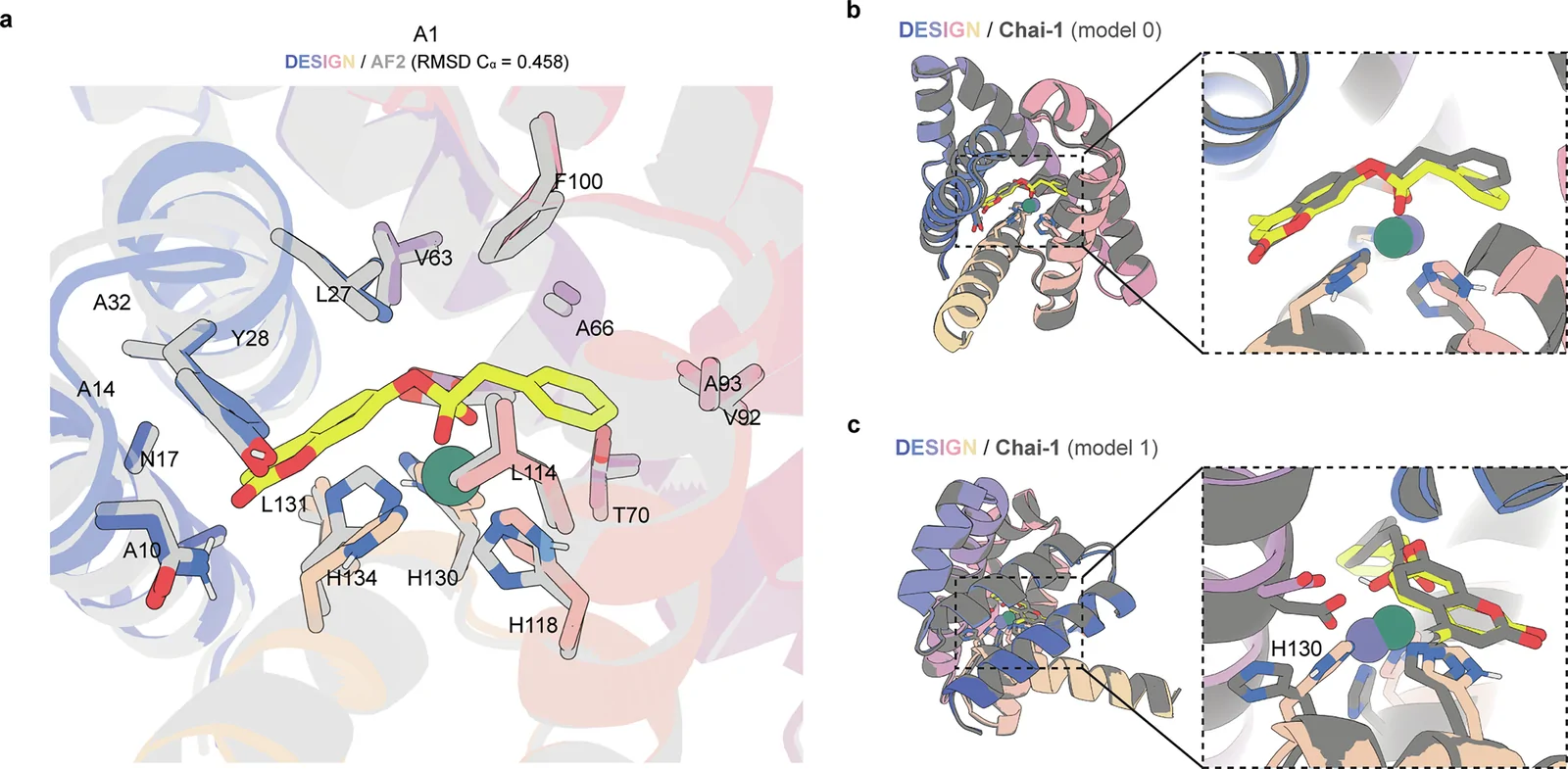
Predicted structures of the A1 (ZETA_1) design. **a**, Active site of the A1 design model with pocket residues labeled. Designed structure is coloured and AlphaFold2 apo-predicted structure is grey. The structures are in high agreement, suggesting that A1 has a high degree of preorganization. **b**, Global and active site comparison of the A1 design (colored) with model 0 of the Chai-1^28^ structure sequence/scaffold) with Zn(II) and 4MU-PA. The substrate SMILES file was provided to Chai-1, not the tetrahedral intermediate, whereas the A1 design model contains the transition state of the nucleophilic attack of hydroxide to the substrate. The global scaffold fold prediction by Chai-1 is nearly identical to the design model scaffold (r.m.s.d. Cα = 0.413). Additionally, the zinc and substrate docking positions by Chai-1 are nearly identical to the design model; model 0 of Chai-1 also predicts the zinc being coordinated by the 3 histidines (H118, H130, H134) in nearly the same orientations as the design model. **c**, Global and active site comparison of the A1 design model (coloured) with model 1 of the Chai-1 structural prediction (grey) in the presence of zinc and 4MU-PA. Note this is shown from the side/back view. Again, the global scaffold fold prediction and substrate docking position by Chai-1 are nearly identical to the design model. Interestingly, Chai-1 predicts a slightly different zinc docking position in model 1 with H130 flipped away from the active site, and D67 is instead coordinating zinc with the other two histidines (H118 and H134). This supports the hypothesis that H130 and D67 are competitively binding zinc, explaining the results observed from the mutant activity and zinc binding experiments. Thus, it is unlikely that D67 is functioning efficiently, if at all, as a general base, providing a rational starting point for potential redesign.

Design ZETA_1 not only has remarkably high activity but was also the top-ranked design in our in silico ranking. The structure in the absence of substrate was predicted to be very close to the design model by AlphaFold2 (Extended Data Fig. 5a and Supplementary Figs. 9 and 10), and the designed active site of ZETA_1 was predicted to be highly preorganized by PLACER, with the catalytic side chains fixed in place and the substrate held closely in its designed position, adjacent to the proposed Zn(II) site. PLACER^12^ is a deep neural network that, given a protein backbone containing a substrate, fully randomizes the positions of the substrate and all side chains within a 600-atom sphere, and then generates new coordinates for these groups^12^; repeated PLACER trajectories generate an ensemble of possible side-chain conformations and small molecule docks. Design ZETA_1 stood out from the other designs in both the extent of catalytic site preorganization (the catalytic side chains were largely fixed in space in catalytically competent conformations) and the positioning of the substrate–transition state in the active site (in the ZETA_1 ensemble, the substrate remained largely fixed in space in the active site, whereas in the inactive designs H7 and H8, it fluctuated considerably) (Fig. 2e–h and Supplementary Videos 2–5). Seven designs based on the same ZETA_1 backbone family were initially filtered out during the design selection phase, as they had suboptimal PLACER metrics; we retrospectively expressed and purified these designs and found that they had very low or no activity, further highlighting the utility of PLACER ensemble calculations for identifying active designs (Supplementary Fig. 11). These findings suggest that combining global structure prediction with detailed PLACER modelling of the active site provides a powerful approach to assessing the catalytic machinery and substrate binding geometry for design selection (Supplementary Fig. 10).

The ZETA_1 active site consists of a primarily hydrophobic pocket with three histidines binding Zn(II) with their Nε atoms, an aspartate as a potential general base, and an asparagine that forms a hydrogen bond to the coumarin ring (Fig. 3a). As in the original theozyme model used to generate ZETA_1, the Zn(II) ion also acts as an oxyanion hole, stabilizing the developing negative charge at the transition state; there are no nearby side-chain hydrogen bond donors (Extended Data Fig. 5). Zinc is absolutely critical for ZETA_1 activity: extraction of bound Zn(II) by dialysis in the presence of the chelator 1,10-phenanthroline completely eliminated activity, and activity was subsequently restored by addition of zinc to the solution (Fig. 3f). Zinc titration experiments measured a dissociation constant (*K*_D_) for Zn(II) of 41 ± 5 nM, which is similar to those of previous designed zinc enzymes^26,39^, but higher than native zinc hydrolases^18,40–42^, which typically have *K*_D_ values less than 10 nM.

We carried out mutagenesis experiments to probe the contributions of the designed catalytic residues to Zn(II)-binding and catalysis (Fig. 3g–i and Supplementary Figs. 12–14). In the design model, N17 positions the substrate by hydrogen bonding with the lactone carbonyl of the coumarin moiety and could stabilize the developing negative charge on the leaving group; the N17A mutation led to a 8.1-fold decrease in *k*_cat_/*K*_M_ (Supplementary Fig. 13). Mutation of all three metal-coordinating histidine residues to alanine simultaneously (H118A/H130A/H134A), as well as two of the three single histidine-to-alanine substitutions (H118A/H134A), completely inactivated the enzyme, as expected. Mutating the third Zn(II)-coordinating residue to alanine (H130A) resulted in a decrease of only 13-fold in *k*_cat_/*K*_M_, and mutation of the proposed general base D67 to alanine had little effect on *k*_cat_/*K*_M_ and increased Zn(II)-binding affinity. These results suggest that H134/H118/H130 and H134/H118/D67 may be competing Zn(II)-binding sites owing to the close proximity of the coordinating side chains of H130 and D67, which was corroborated by Chai-1 (ref. ^13^) predictions of the protein–Zn(II)–substrate complex (Extended Data Fig. 5b,c); the D67A mutation may confine the zinc to the originally designed coordination sphere with the three histidines, which is more catalytically competent. In the H130A mutant, D67 is likely to coordinate Zn(II) and maintain binding, albeit in a less optimal binding geometry, lowering the zinc affinity and enzyme activity.

**Fig. 4:**
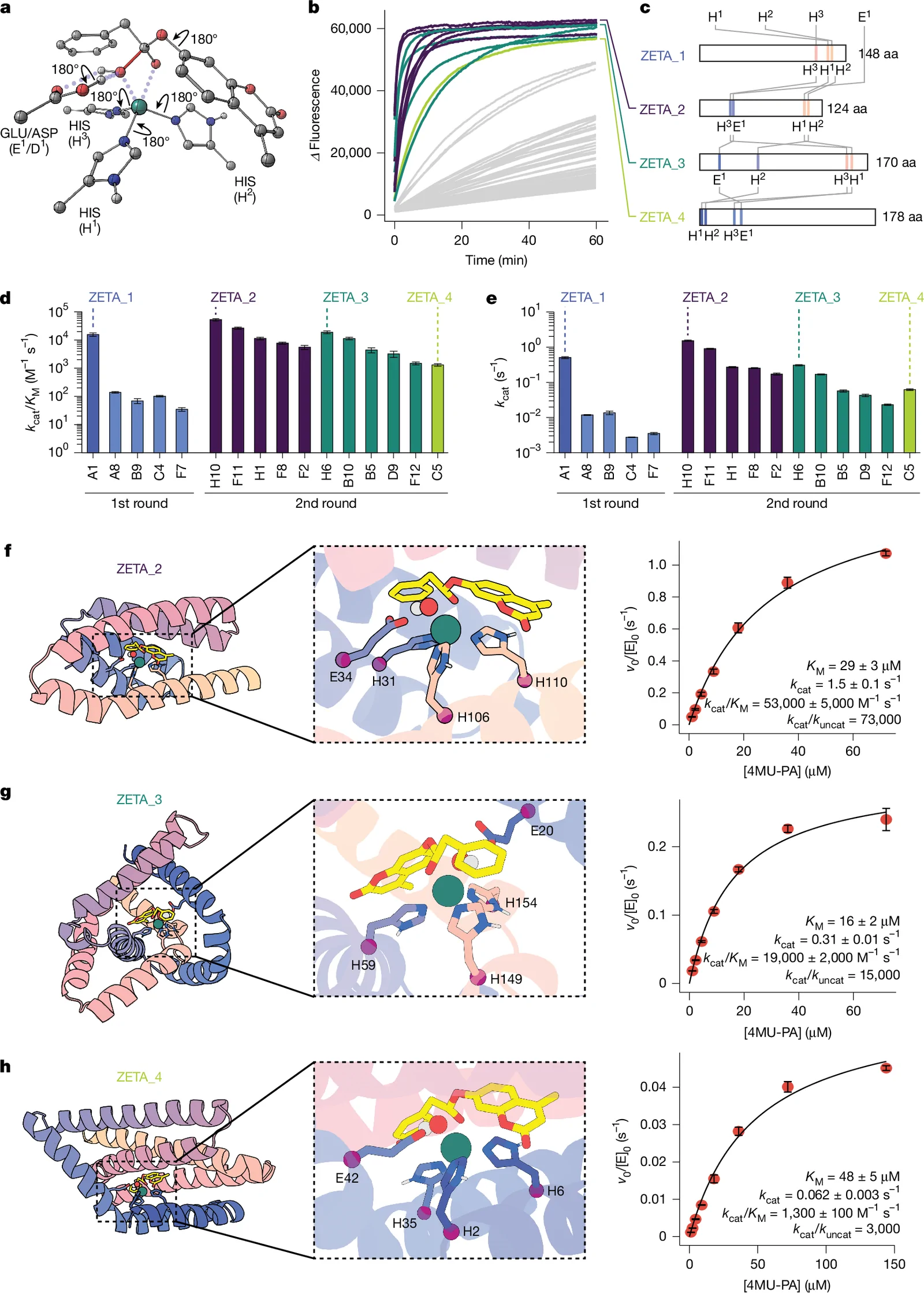
Characterization of second round designs. **a**, Second round DFT theozymes containing the catalytic base. Zn(II) is coordinated by the His Nε atoms in these theozymes, and thus C_β_ is explicitly modelled. **b**, Reaction progress curves coloured by scaffold family. **c**, Sequence length and catalytic residue positioning for the top design in each scaffold, named ZETA_2-4. These designs differ from each other and from ZETA_1. **d**,**e**, Steady-state *k*_cat_/*K*_M_ and *k*_cat_ parameters for the 11 second round hits, with colours corresponding to scaffolds using the scheme in **b**,**c**. Activities are higher, on average, than in the first round of designs (blue). **f**–**h**, Design model (left) with close-up view of the active site (middle) for ZETA_2 (**f**), ZETA_3 (**g**) and ZETA_4 (**h**). Right, Michaelis–Menten plots and parameters. Data represent the mean ± s.d. of three independent measurements of Michaelis–Menten parameters (**d**,**e**) and initial velocity (**f**–**h**).

**Extended Data Fig. 6:**
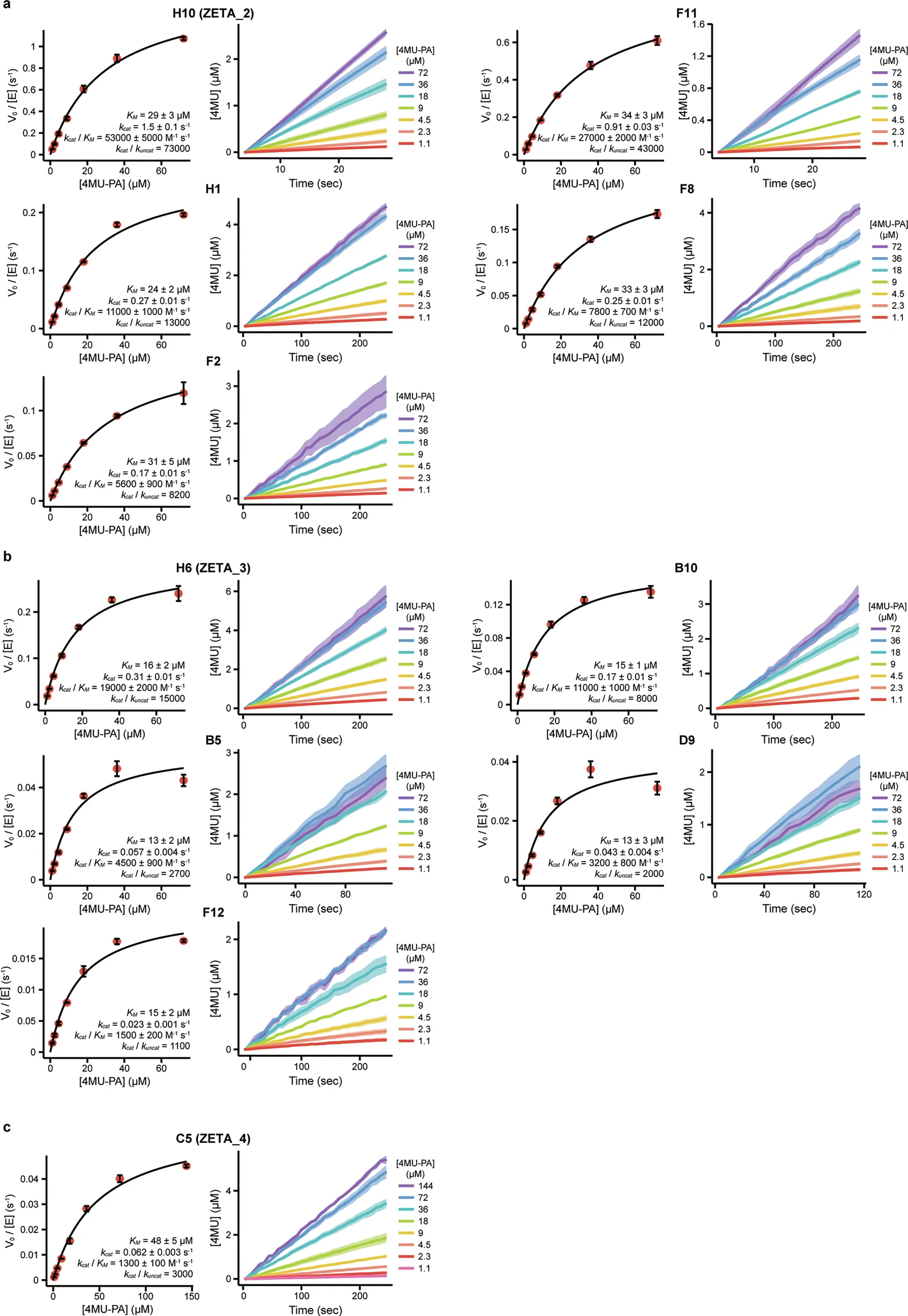
Michaelis-Menten kinetics of eleven major hits from the second design campaign. **a**-**c**, Kinetics of designs grouped by (**a**) ZETA_2 scaffold, (**b**) ZETA_3 scaffold, and (**c**) ZETA_4 scaffold.

**Extended Data Fig. 7:**
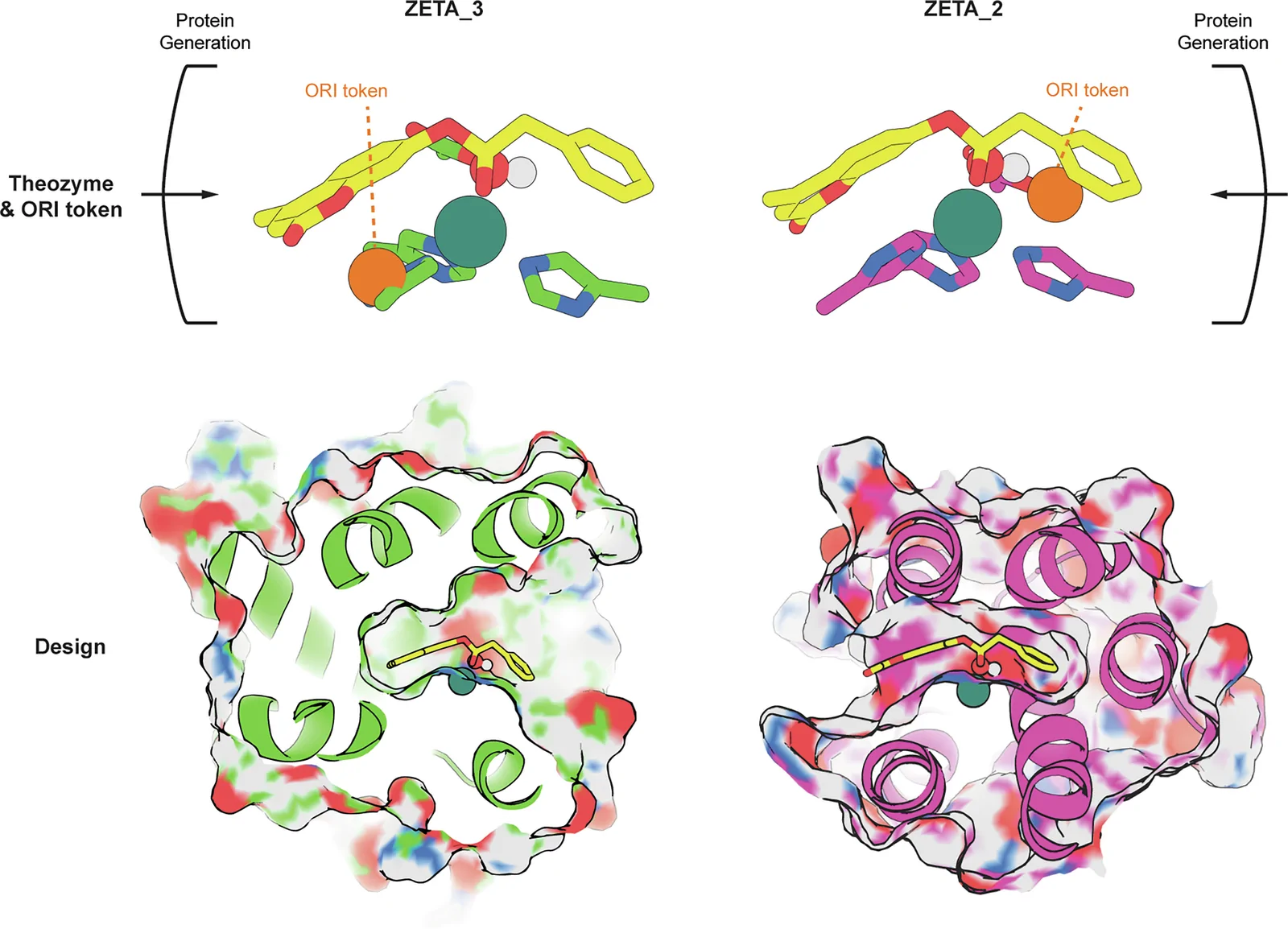
Opposite substrate binding modes in ZETA_2 and ZETA_3 design models result from ORI placement. The ORI token is an optional coordinate fed to the updated RFdiffusion2, which instructs the model on where it should aim to generate the protein center of mass (Supplementary Methods 4.2). Thus, the ORI token can be used to specify which parts of the substrate/theozyme should be more surrounded by protein (buried) on average. ZETA_2 (right) and ZETA_3 (left) are designed to bind the substrate in opposite orientations; this arose from the opposite placement of ORI tokens with respect to the substrate. The bottom panels show a cross-section of the design models for these enzymes with the surface view to observe the designed substrate binding pocket. In ZETA_3 (left) the PA is solvent-exposed and the 4MU is buried because the ORI token is next to the 4MU. In ZETA_2 (right) the 4MU is solvent-exposed and the PA is buried because the ORI token is next to the PA.

Guided by these observations, we started from new DFT theozymes explicitly containing the catalytic base, and generated protein structures scaffolding these theozymes using a newer version of RFdiffusion2 trained from random weight initialization on a threefold-larger dataset (previous versions were fine-tuned from structure prediction weights) (Fig. 4a, Supplementary Data 1 and Supplementary Methods 4.2). Designs whose Chai-1 predictions of the protein–Zn(II)–substrate phosphonate ester complex, mimicking the reaction transition state, closely matched the design models with high confidence were identified by PLACER to have highly preorganized active sites (Supplementary Figs. 15 and 16). Ninety-six such designs spanning 37 RFdiffusion2-generated backbones were selected for experimental characterization (Supplementary Fig. 17 and Supplementary Data 2 and 3). Eighty-five of the 96 designs were expressed and soluble (Supplementary Fig. 18), and 11 designs spanning 3 different folds had substantial zinc-dependent 4MU-PA hydrolysis activity (Fig. 4b,c and Supplementary Fig. 19). Michaelis–Menten analysis revealed that 5 designs had a *k*_cat_/*K*_M_ greater than 10^4 ^M^−1^ s^−1^ and 6 designs had a *k*_cat_/*K*_M_ greater than 10^3 ^M^−1^ s^−1^ (Fig. 4d, Extended Data Fig. 6 and Extended Data Table 1). The most active designs for each backbone had a *k*_cat_/*K*_M_ = 53,000 ± 5,000 M^−1^ s^−1^ (ZETA_2), *k*_cat_/*K*_M_ = 19,000 ± 2,000 M^−1^ s^−1^ (ZETA_3), and *k*_cat_/*K*_M_ = 1,100 ± 200 M^−1^ s^−1^ (ZETA_4) (Fig. 4f–h and Supplementary Fig. 20). ZETA_2 has a *k*_cat_ = 1.5 ± 0.1 s^−1^, a threefold increase over the *k*_cat_ of ZETA_1, and close to that of the metallohydrolase MID1sc10 obtained after 10 rounds of directed evolution^24^. RFdiffusion2 enables specification of the position of the substrate relative to the centre of mass of the designed protein; for ZETA_2 and ZETA_3, the protein was centred near the phenylacetate and 4-methylumbelliferyl moieties, respectively, of 4MU-PA, resulting in opposite substrate binding modes in the design models (that is, the 4-methylumbelliferyl is exposed in ZETA_2 and the phenylacetate is exposed in ZETA_3) (Extended Data Fig. 7).

The success rate in the second design campaign was considerably higher than the first campaign (11 out of 96 versus 1 out of 96 designs with *k*_cat_/*K*_M_ greater than 10^3 ^M^−1^ s^−1^), supporting the conclusions from the first round analysis (Supplementary Figs. 21–26, Supplementary Table 3, Supplementary Discussion 2 and Supplementary Methods 4.2). Circular dichroism experiments confirmed that all active enzyme scaffolds from both design campaigns possess secondary structures consistent with their design models, indicating proper folding (Supplementary Fig. 21). The structures of ZETA_1-4 are rather different from each other and previously known metallohydrolases (Extended Data Fig. 4). The sequence positions of the catalytic residues in each of these enzymes are also very different, highlighting the diversity of RFdiffusion2 generated design solutions (Fig. 4c and Supplementary Tables 4 and 5).

**Fig. 5:**
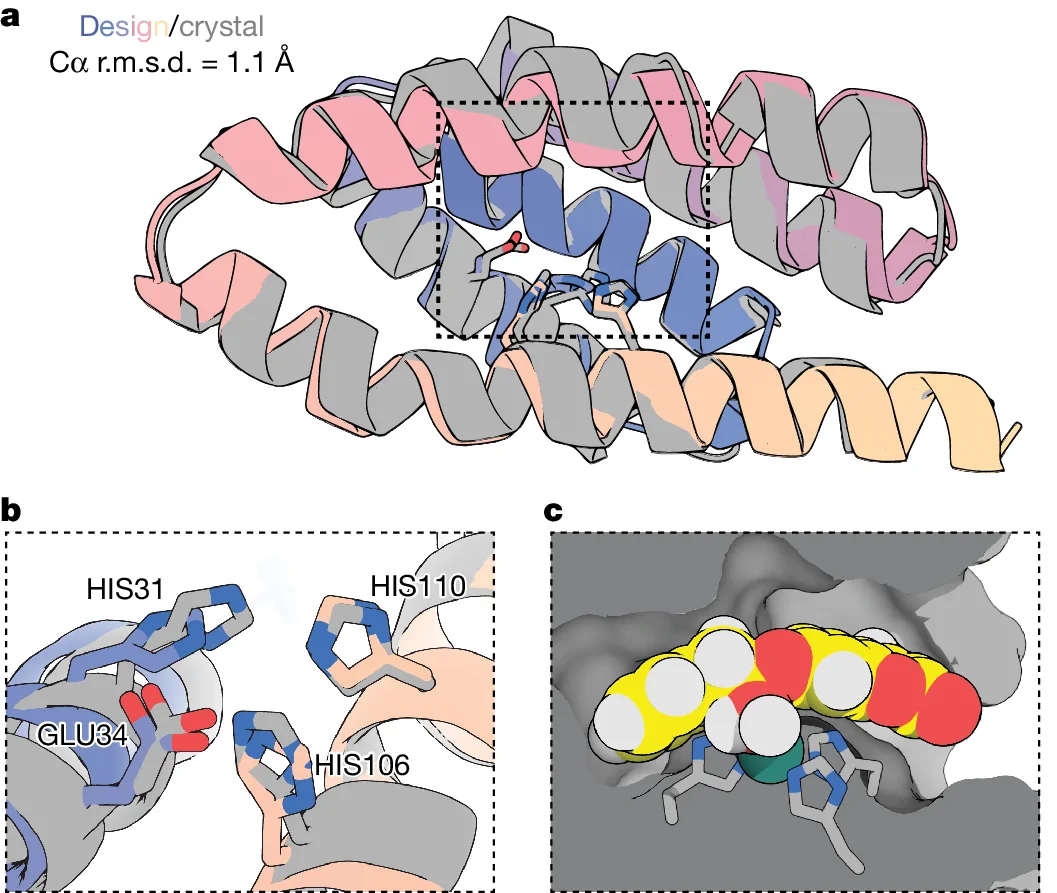
Crystal structure of ZETA_2 closely resembles the design model. **a**, Cα superposition of the design model and the X-ray crystal structure of ZETA_2 (PDB: 9PYJ) in the apo state, resolved at 3.5 Å resolution. Catalytic residues are shown as sticks. The structures are in close agreement (Cα r.m.s.d. = 1.1 Å) and the catalytic residues in the experimental structure are preorganized close to their designed catalytic geometry. **b**,**c**, Magnification of the active site of ZETA_2, highlighting the agreement between the experimental and designed catalytic geometry (**b**) and the shape-complementarity of the designed binding pocket and the transition state model (superimposed in from the design model) (**c**). The crystal structure is shown in grey and the design model is in colour in all panels.

**Extended Data Fig. 8:**
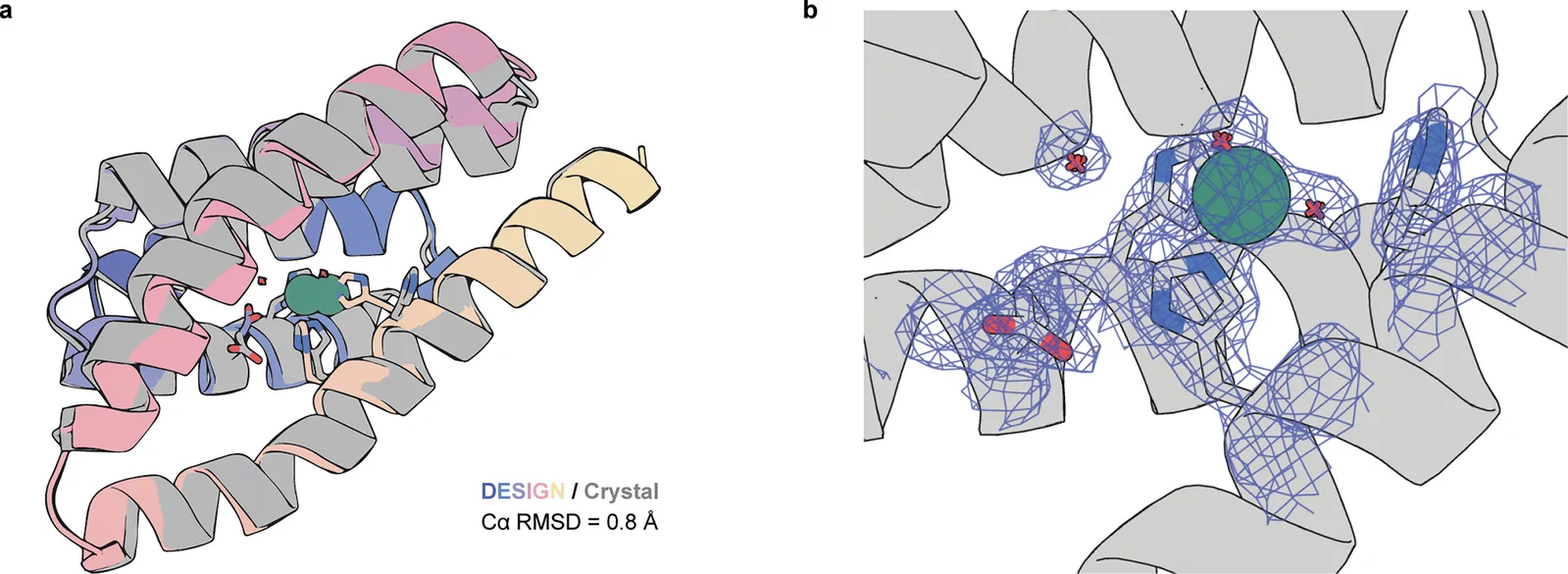
Crystal structure of Zn(II)-bound ZETA_2 after soaking. **a**, Cα superposition of the design model and the X-ray crystal structure of ZETA_2 (PDB: 9PYL) in the Zn(II)-bound state from crystal soaking, resolved at 2.1 Å resolution. Catalytic residues shown as sticks and Zn(II) ions shown as green spheres. The structures are in close agreement (Cα r.m.s.d. = 0.8 Å) and the Zn(II) ion is present with 100% occupancy in the correct binding location (r.m.s.d. = 1.7 Å). **b**, Active site close-up view with the 2mFobs-DFcalc electron-density map (blue mesh) contoured at 1.0 σ for the catalytic residues, Zn(II) ion, and water molecules. Surprisingly, one of the catalytic histidines (H110) is flipped out in the experimental structure to interact with a Zn(II) ion in solution, likely because of the high Zn(II) concentration used in the crystal-soaking buffer. We hypothesize that this may be an intermediate conformational state relevant to Zn(II)-binding that was captured and stabilized in the crystal. Additionally, the catalytic base (E34) adopts a different conformation from the catalytic conformation in this crystal structure; however, conformational flexibility for this residue was also observed in the apo-ZETA_2 crystal structure, with one of the conformations being the catalytic conformation (Fig. 5b). These conformations for E34 and H110 could be artifacts from the crystallization and soaking process or may suggest that ZETA_2 has dynamic properties. If it is dynamic, this poses an interesting question of whether the dynamics accelerate or hinder the activity of ZETA_2. Future work could try to buttress these residues with redesign or new de novo scaffolds to investigate if this would increase or decrease activity. Crystal structure shown in grey and design model shown in colour in all panels.

We determined the structure of ZETA_2, the most active design, in the apo state at 3.5 Å using X-ray crystallography (PDB: 9PYJ; Fig. 5). The experimental structure is in good agreement with the design model, with nearly superimposable backbones (Cα root mean squared deviation (r.m.s.d.) = 1.1 Å) and the catalytic residues preorganized in the designed geometry (Fig. 5a,b). The binding pocket is complementary to the superimposed transition state from the design model (Fig. 5c). We also solved a 2.1 Å structure after soaking ZETA_2 in Zn(II) (PDB: 9PYL; Extended Data Fig. 8); whereas the backbone was again nearly superimposable with the design model (Cα r.m.s.d. = 0.8 Å) and a Zn(II) ion was present with 100% occupancy at the designed location (r.m.s.d. = 1.7 Å), one of the Zn(II)-coordinating histidines (H110) was flipped out to interact with a Zn(II) ion bound at the surface of the protein, probably because of the high Zn(II) concentration in the crystal soaking buffer (250 mM) (Extended Data Fig. 8).

## Conclusion

Here we demonstrate that RFdiffusion2 can generate highly active metallohydrolases directly from active site configurations obtained by quantum chemistry calculations. The zero-shot design of an enzyme (ZETA_1) with a *k*_cat_/*K*_M_ greater than 10^4 ^M^−1^ s^−1^—the top-ranked in silico design out of a total of 96 tested straight from the computer, with no experimental optimization—is a considerable advance for de novo enzyme design, which has previously required extensive design screening and directed evolution to achieve activities at this level^14,24^. The robustness of our design strategy is demonstrated by the zero-shot design of 3 additional enzymes from a new set of 96 designs tested straight from the computer: ZETA_2 and ZETA_3, with a *k*_cat_/*K*_M_ of more than 10^4 ^M^−1^ s^−1^, and ZETA_4, with a *k*_cat_/*K*_M_ greater than 10^3 ^M^−1^ s^−1^. ZETA_1–4 have structures that are very different from each other and from previously known structures. The catalytic efficiencies of ZETA_1–3 are within the range typically observed for native metallohydrolases with similar substrates (*k*_cat_/*K*_M_ = 10^4^ ~ 10^6^ M^−1^ s^−1^) and greater than for all previously designed metallohydrolases prior to optimization^43–47^ (Supplementary Table 2). Experimental characterization of ZETA_1 and ZETA_2 provide strong evidence that they function as designed, utilizing a bound Zn(II) ion to activate a water molecule for nucleophilic attack and to stabilize the resulting oxyanion intermediate and flanking transition states. The results from PLACER and the Chai-1 ensembles suggest that the key to obtaining *k*_cat_/*K*_M_ of over 10^4 ^M^−1^ s^−1^ is precise substrate placement relative to the activated water and the Zn(II) ion. Future metallohydrolase design efforts that: (1) position the general base such that it cannot reconfigure to interact with the bound metal ion (as in the case of ZETA_1); (2) better preorganize the metal-binding residues; and (3) incorporate further side-chain oxyanion stabilization, could increase activity to levels comparable to the most active native metallohydrolases.

Our RFdiffusion2–PLACER design approach has many advantages over previous de novo enzyme design methods and should be broadly applicable for generating efficient catalysts for a wide diversity of chemical reactions. By enabling direct scaffolding of side-chain functional groups, rather than backbone N-Cα-C=O coordinates as required in enzyme design calculations with RFdiffusion^1,27^, RFdiffusion2 bypasses the need for explicit enumeration of catalytic side-chain rotamer conformations and placement of the catalytic residues along the linear sequence—this enables each design trajectory to sample from the enormous space of possibilities rather than being confined to a small subspace. Assessment of active site preorganization and substrate–transition state positioning with PLACER and Chai-1 proved remarkably effective at identifying the most active designs; this result, together with similar observations with de novo designed serine hydrolases^1^ and retroaldolases^12^, suggests that PLACER and related deep learning approaches will be widely useful for design ranking. In our laboratory, we have found the RFdiffusion2–PLACER design approach described here to yield biocatalysts for a variety of bond-breaking and bond-making chemical reactions, and we look forward to seeing what the broader design community can generate with these tools, which we are making freely available.

### Reporting summary

Further information on research design is available in the Nature Portfolio Reporting Summary linked to this article.

## Online content

Any methods, additional references, Nature Portfolio reporting summaries, source data, extended data, supplementary information, acknowledgements, peer review information; details of author contributions and competing interests; and statements of data and code availability are available at https://doi.org/10.1038/s41586-025-09746-w.

## Supplementary information

Supplementary Information Supplementary Figs. 1–27, Tables 1–5, Supplementary discussion, materials and methods, and references. The Supplementary Information file is intended to aid readers in understanding and reproducing the in silico and experimental metallohydrolase design results.

Reporting Summary

Supplementary Video 1 RFdiffusion2 inference trajectory for the generation of the ZETA_1 scaffold. The minimal theozyme from quantum chemistry, shown as sticks with Zn(II) depicted as a green sphere, is fixed in place during inference; RFdiffusion2 applies flow matching to generate a scaffold containing this theozyme, resolving the positions and remaining unspecified coordinates of the catalytic residues. Residue C_α_ atoms are depicted as spheres and colored with a gradient that reflects their final sequence position.

Supplementary Video 2 Video of PLACER ensemble predictions of the substrate in the ZETA_1 active site, ranked from best to worst by predicted substrate confidence (pRMSD). Predictions (colour) are superimposed on the design model (grey), with increasing red intensity indicating lower-ranking confidence (higher pRMSD). Most ensemble predictions, particularly the most confident ones, position the substrate close to that in the design model.

Supplementary Video 3 Video of PLACER ensemble predictions of the substrate in the ZETA_1 active site, ranked from best to worst by predicted substrate confidence (pRMSD), highlighting the catalytic residue predictions. Predictions (colour) are superimposed on the design model (grey), with increasing red intensity indicating lower-ranking confidence (higher pRMSD). Most ensemble predictions, particularly the most confident ones, position the catalytic residues close to those in the design model.

Supplementary Video 4 Video of PLACER ensemble predictions of the substrate in the active site of the catalytically-inactive H8 design, ranked from best to worst by predicted substrate confidence (pRMSD). Predictions (colour) are superimposed on the design model (grey), with increasing red intensity indicating lower-ranking confidence (higher pRMSD). The predicted substrate position varies widely and does not consistently align with the design model, suggesting that the design may be inactive because it does not properly bind and orient the substrate for hydrolysis.

Supplementary Video 5 Video of PLACER ensemble predictions of the substrate in the active site of the catalytically-inactive H7 design, ranked from best to worst by predicted substrate confidence (pRMSD), highlighting the catalytic residue predictions. Predictions (colour) are superimposed on the design model (grey), with increasing red intensity indicating lower-ranking confidence (higher pRMSD). One of the catalytic histidines in the top right (purple) is consistently predicted in a much different rotamer than designed, which would no longer allow it to coordinate Zn(II), potentially explaining why this design could be inactive.

Supplementary Data 1 Coordinates for all transition state models calculated by density functional theory. XYZ file containing 36 sets of transition state coordinates for the theozymes optimized by density functional theory and used as inputs for the metallohydrolase design campaigns. The first 4 sets of coordinates correspond to the inputs used in the first design campaign and the second 32 sets of coordinates correspond to the inputs used in the second design campaign.

Supplementary Data 2 Protein and DNA sequences for all ordered designs. Excel spreadsheet containing 4 sheets of protein and DNA sequences for all designs ordered from the first and second design campaigns, including the ZETA_1 mutants and variants. The sheets are labelled with their corresponding contents. DNA sequences are provided as the exact coding sequences used for expression in plasmids (beginning with ATG corresponding to the M1 residue).

Supplementary Data 3 PDB design models for all ordered designs. Zip archive containing PDB design models for all ordered designs from both design campaigns, including ZETA_1 mutants and variants. The models were generated in the final FastMPNNdesign step using Rosetta catalytic constraints during fastrelax, positioning the catalytic residue functional groups and Zn(II)–TS complex as close as possible to the density functional theory geometry while respecting the Rosetta energy function. These design models are considered hypotheses for the protein-metal-transition state complexes.

Peer Review File

## Figures and Tables

**Extended Data Table 1:**
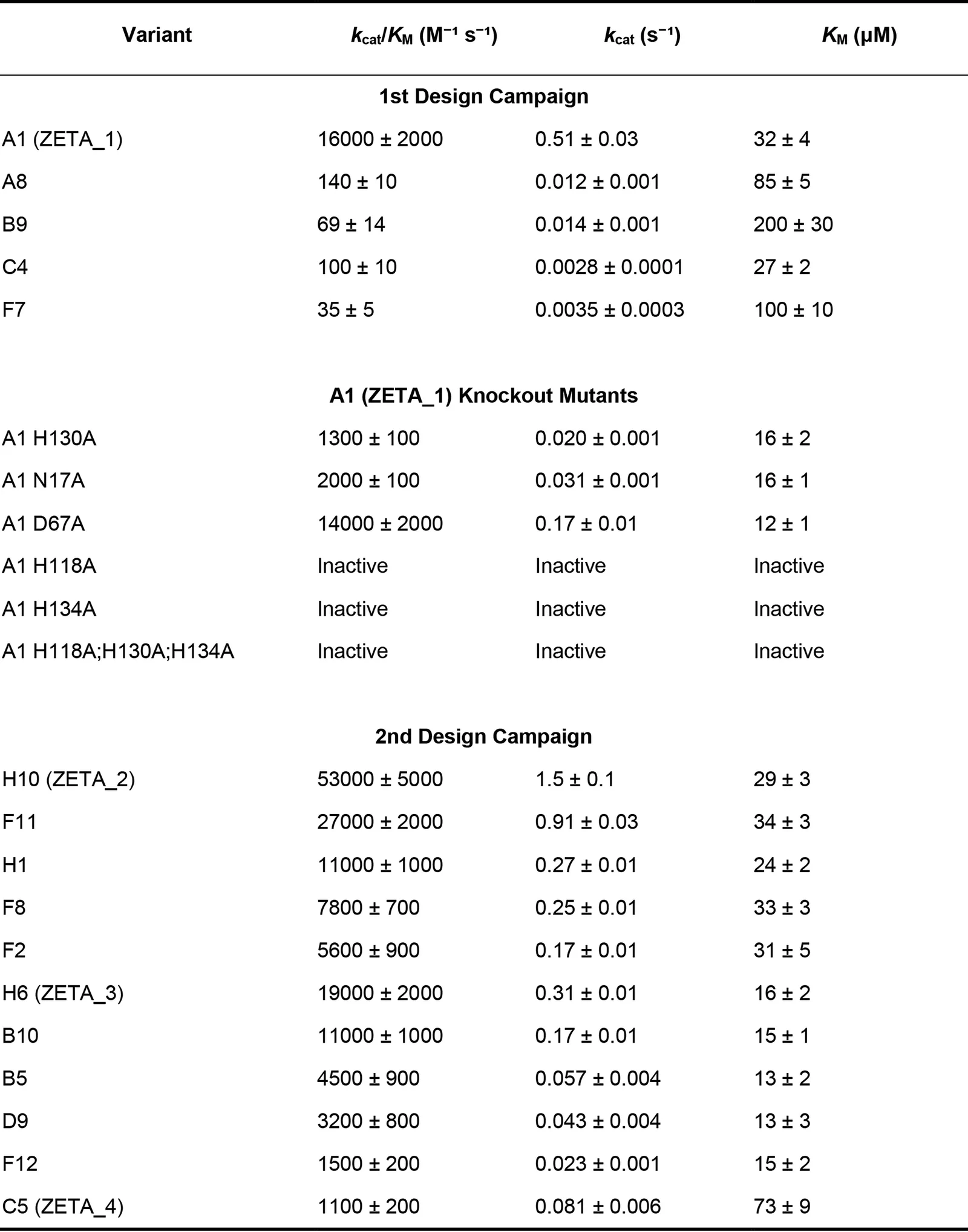
Michaelis-Menten parameters for the hydrolysis of 4MU-PA by the designed de novo metallohydrolases and mutants

**Extended Data Table 2:**
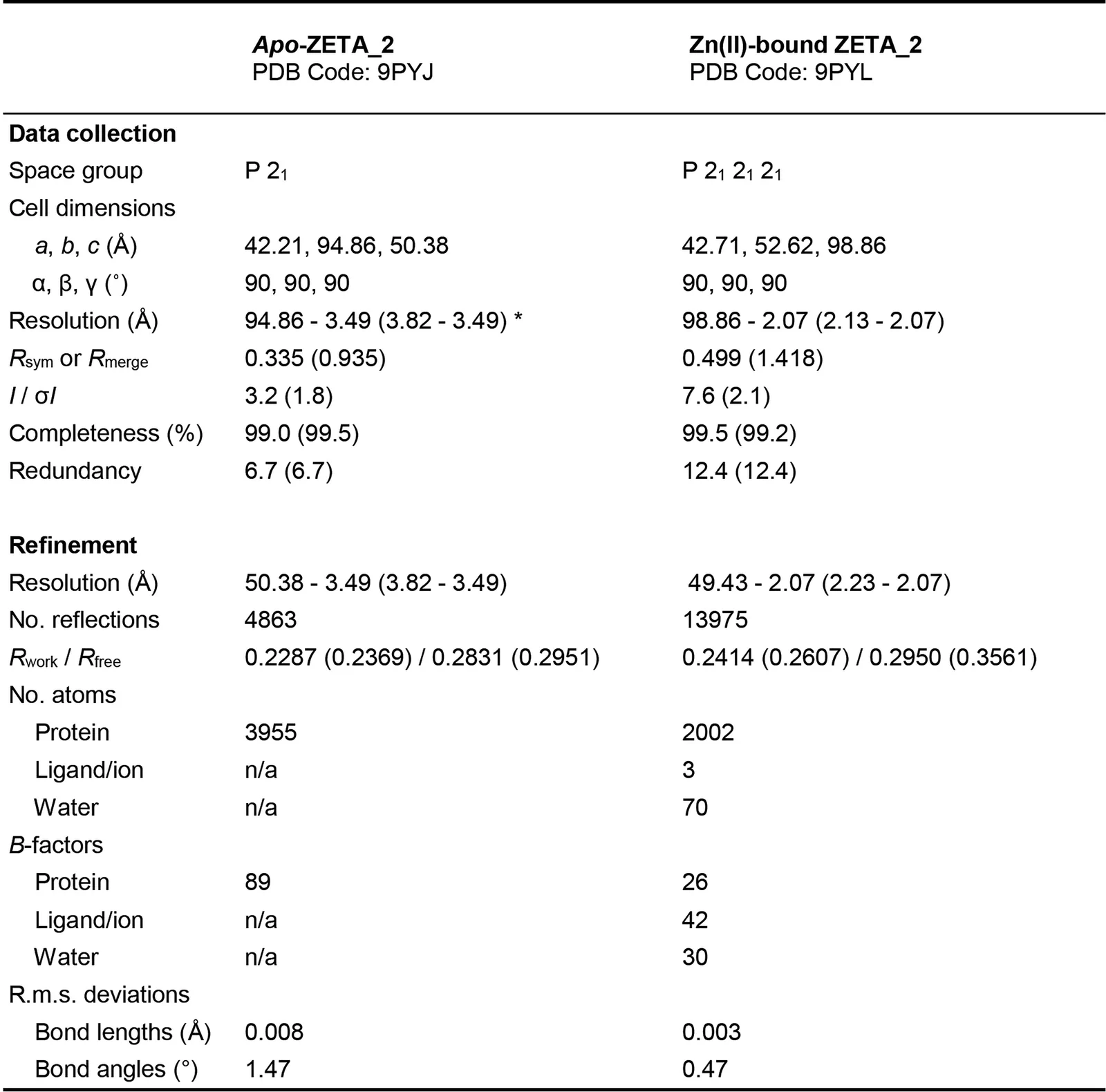
Crystallography data collection and refinement statistics

*Single xtal used for each data/structure. *Values in parentheses are for highest-resolution shell.

## Data Availability

All data are available in the main text or as supplementary materials, including the DFT coordinates of the theozymes from each design campaign (Supplementary Data 1), the ordered protein sequences from each design campaign (Supplementary Data 2) and the design models of the ordered sequences from each design campaign (Supplementary Data 3). For gel source data, see Supplementary Fig. 27. Protein crystal structure coordinates and structure factors are available in the Protein Data Bank with PDB IDs 9PYJ (apo ZETA_2) and 9PYL (Zn(II)-bound ZETA_2).

## Code availability

RFdiffusion2 is publicly available on GitHub at https://github.com/RosettaCommons/RFdiffusion2. PLACER is publicly available on GitHub at https://github.com/baker-laboratory/PLACER. Other design scripts and JupyterHub notebooks of the design pipelines used to design metallohydrolases are publicly available on GitHub at https://github.com/baker-laboratory/Metallohydrolase_Enzyme_Design.
