# Supplementary material for: Computational design of metallohydrolases
Source: Nature. 2025 Dec 3;649(8095):246–53. doi: 10.1038/s41586-025-09746-w (PMC12727532; doi:10.1038/s41586-025-09746-w)
Supplement: Supplementary file 1 [file 41586_2025_9746_MOESM11_ESM.pdf]

nature portfolio

Peer Review File

Computational Design of Metallohydrolases

Corresponding Author: Professor David Baker

Any redactions in this file are there to maintain patient confidentiality, the confidentiality of unpublished data, or to remove third-party material.

This file contains all reviewer reports in order by version, followed by all author rebuttals in order by version.

Version 0:

Reviewer comments:

Referee #1

(Remarks to the Author)
Summary of the key results:

In their manuscript, entitled 'Computational Design of Metallohydrolases' Kim et al. detail the use of an unpublished tool, RFflow, to scaffold the active site of a zinc-dependent hydrolase. In contrast to previous studies, they could start from a minimal quantum-chemistry generated active site description. The authors elegantly show that their approach yielded five active metallohydrolases in a set of 96 designs, one of which exhibits rate-accelerations orders of magnitude higher than previously reported designed metallohydrolases.

Kim et al. highlight the big advantage of their sequence position agnostic design approach, which simplifies the whole process to scaffold-calculated transition states. They also stress the possibility of enhanced backbone sampling and make it clear that detailed knowledge about rotameric states of sidechains is obsolete with their approach. Similar to other recent preprints, Kim et al. also use a hybrid ML and energy-based sequence design approach to find optimal structure-sequence pairs.

In addition, they report on the importance of ChemNet (available as preprint, no code available) for the selection of designs in combination with more ‘classic’ (Rosetta-based) metrics.

To verify their findings, the authors performed thorough biochemical analysis of their designs, including detailed kinetics of variants of their most active design, which is one of the most important aspects of the manuscript.

Originality and significance:

This study elegantly highlights the potential of de novo enzyme design and introduces the use of a new ML-based backbone generator. While I believe that this work can become suitable for publication in Nature, it appears to be incomplete in its present form and a little ‘premature’, with all the focus on RFflow, without RFflow described in detail. In addition, the manuscript is very short und lacks substantial detail in the discussion of the transition state calculations, hypothesis-driven interpretation of the results (besides A1 as the best design), holistic insights and detail on the analysis of the activity data and, most importantly, lack of biophysical and structural data of the designs. If the authors can address these shortcomings, I recommend publishing this study in Nature.
In its present form though, I suggest including this as a terrific experimental validation of the yet unpublished RFflow manuscript, or submitting it to a more specialized journal, such as Nature Chemistry or Nature Catalysis.

Overall comments on the manuscript:

Kim et al. convincingly show the benefit of having a sequence position agnostic backbone generation tool at hand, which can be used to scaffold a minimal active site description coming from quantum chemistry calculations (minimal in this case means starting from a subset of atoms of the individual side chains involved in stabilizing the transition state). The argument that exhaustively searching the astronomically large space of backbone geometries around an active site is impossible with previous approaches, is valid. However, there have been recent preprints that made use of RFdiffusion and similar

approaches, and they arrived at similar results, for different reactions, while screening a similar or even smaller number of designs. With the additional step of using RFJoint to subsample loop regions, etc. it is not quite clear if Kim et al.'s approach is more efficient than what people used before. It would be beneficial to have statistics on sampling and running times to be able to compare the efficiency of individual approaches.

In addition to simplifying the design problem, the real interesting finding here is that the 'best' design also displayed the highest activity by a large margin. The authors do a good job in analyzing this particular design. Yet, the study falls short in a comparative analysis of the best (A1) design vs. the less active designs vs. the inactive designs (e.g. knock-out mutants of all active designs similar to the ones made for A1). Given that all designs were selected according to the same criteria, this dataset holds great potential to decipher intricate details about the origin of catalysis in metallohydrolases.

Suggested improvements:

• The manuscript would benefit tremendously from a more detailed biophysical and structural analysis of at least the most active design. Ideally, the authors would determine a crystal structure of the design, to have experimental proof about their claims of pre-organization and design accuracy. I understand that this is a lot to ask, so at least a more detailed solution-based study should be performed – this includes SAXS and CD data on the active designs. As far as CD is concerned, it would be good to show temperature ramp plots, too. Fig. 3 of the manuscript currently holds the only structural information, which are CD spectra at 20C and 95C. However, without the actual temperature ramp there, it is hard to judge from this plot if this protein is stable or a molten globule, since it loses a significant amount of CD signal at 95C but refolds after cooling back down. An even better proof of proper foldedness with and without the addition of Zn would be to record HSQC spectra for the active designs. The His peaks should easily be identifiable, if the peaks are well dispersed.

• For the active designs, it would be great to know which of the four transition states they originated from. Is there a trend? Could it be that one of the QM-based TS is superior than the others for scaffolding? If yes, can the authors identify why this might be?

• The section about using RFJoint to diversify the folds needs more explanation. It is not clear why this is necessary at all, if RFflow is superior to previous methods. Was it essential for making better pockets? Upon examination of some of the design models that were not ordered by the authors (thanks for providing them), it looked as if most of the RFflow-generated pockets were heavily buried/occluded. Was the RFJoint step necessary to make them more accessible for substrate binding? If yes/no please clarify.

• The filter/selection criteria for the initial rounds of designs (plddt, pTM, etc) are rather bad/low and need a more extensive explanation. Previous studies mostly focused on generated backbones with higher/better values from the beginning on. The strategy described here is very interesting but needs more explanation.

• The argument of why the general base was put into the TS calculation, but removed from the minimal active site during scaffolding is not clear to me. Were the backbones that RFflow produced much worse when the general base residue was included? The same applies to the H-bonding residues (for the applicable TS).

• A more in-depth analysis and discussion of why other sequences on the same backbone as the A1 design were not active, would be very interesting. Do the metrics of these alternative sequences differ a lot (e.g. in the authors composite score, AF2, ChemNet, etc)?

• What is the speed of the background reaction in water? A comparison over kcat vs kuncut would be beneficial in light of the many inactive designs.

• A comparative discussion of how the best design compares to natural metallohydrolases would provide a more complete picture about how good the best design really is.

• It is surprising that the triple-H variant still shows high affinity for Zn. Thus, the authors should either fit their titration curves of this variant to a two-binding site model or perform other experiments, such as native MS, to decipher the actual number of Zn binding sites of A1.

• A general discussion about why only one out of 96 ordered designs is highly active, or what sets this design apart from the rest, would be very informative. Is there another design with very similar metrics, which is not active at all in the dataset of 96? Can there be any conclusion drawn from it?

Minor comments:

• In Fig. S12, what does this ddG correspond to? Should it be dG of binding?

• Fig. S13 would be more insightful if the same plots could be done with Chai-1 (prediction vs design with ligand) vs AF2, since the authors also use Chai-1 as a validation tool in their study.

• Table S3 is highly informative, but mixes some de novo designs with designs based on natural scaffolds in the section 'Computationally designed non-hydrolytic enzymes (from existing scaffolds, no laboratory evolution)'. This section should be properly split between de novo and natural scaffolds. Also the values reported should be double checked; there is some mix-

up between s-1 and min-1.

• A table with statistics about pocket volumes, ligand burial, and substrate-bb contacts of the generated and designed scaffolds would be very informative, particularly since they are of varying length and only one in 96 is really active.

(Remarks on code availability)
My code review is limited to running the demo, as code is not yet available for neither RFflow nor ChemNet. The installation instructions and demo are good to follow and execute, if you have performed protein design tasks with similar tools before.

Referee #2

(Remarks to the Author)
This paper introduces RFflow, a novel generative model for protein design, and applies it to the challenging problem of engineering histidine-bound zinc metallohydrolases. The work is notable for its conceptual innovations and experimental validation, which demonstrate improvements over previous methods in both methodology and outcomes.

The authors' workflow integrates quantum chemistry-based active site modeling with generative AI. Specifically, density functional theory (DFT) is used to define the active site geometry and substrate poses--an extension of the theozyme concept. The model simplifies the histidine residues in the active site, representing them solely by their imidazole rings, excluding the sidechain and backbone atoms. This abstraction is then used to generate protein scaffolds around the catalytic motif, and the scaffolds are populated with sequences optimized using ProteinMPNN. The workflow includes a design validation step, comparing the generated sequences with predicted structures from AlphaFold2 (AF2). This serves as a proxy for "designability," assessing whether the designed sequence is likely to fold into the intended structure.

The novel aspect of RFflow lies in its conceptual departure from previous models like RFdiffusion. Diffusion models typically introduce noise into protein structures, represented as a set of frames centered on Cα atoms, and gradually reverse this noise to generate novel scaffolds. RFflow, in contrast, uses flow models from computer science, which rely on vector fields to guide atomic positions without requiring explicit sequence information. This approach enables the model to focus on the placement of functional groups and atomic coordinates, bypassing the need for sequence-specific constraints during scaffold generation.

The authors experimentally characterized their designs, focusing on 96 zinc metallohydrolase candidates. Of these, 4-5 showed catalytic activity, with the best design achieving a catalytic efficiency (kcat/Km) of 2 x10^4 M^{-1}s^{-1}. While this efficiency is 2–4 orders of magnitude lower than that of natural enzymes, it represents a remarkable improvement over previously designed metallohydrolases. The standout design, A1, displayed the most preorganized active site, as assessed by ChemNet analysis. Mutagenesis studies confirmed the critical positioning of active site residues, suggesting a role for an aspartate residue in catalysis. Alternative sequences for the same fold showed lower ChemNet scores and reduced activity, highlighting the importance of active site preorganization. Additional structural and biophysical characterization of the A1 scaffold by circular dichroism (CD) spectroscopy confirmed its high helical content and showed reversible thermal unfolding. Notably, A1's fold is not found in the Protein Data Bank (PDB).

RFflow is a significant conceptual advancement over RFdiffusion, introducing flow models for sequence-agnostic atomic scaffolding. The catalytic efficiency of the best design is an order of magnitude better than previous de novo metallohydrolases. The authors discuss future directions for improving activity, such as additional rounds of directed evolution. However, the description of RFflow is somewhat unclear, particularly regarding how the vector field is trained and applied. The main reference for RFflow is a "preprint in preparation," which limits the reader's ability to fully understand and evaluate the method. It would be helpful to clarify how RFflow differs in implementation from Jaakkola's flow models.

(Remarks on code availability)

Referee #3

(Remarks to the Author)
This is very interesting work. The designed metallohydrolase is a significant achievement. The designed protein has excellent activity, which is a notable achievement for a truly de novo metalloenzyme. The biochemistry and molecular biology characterizing the proteins is thorough and detailed. The authors provide good evidence to confirm the protein residues are functioning as designed, which is important verification of the design approach.

The work would be strengthened by the addition of structural biology to verify overall structure, ligand binding, etc: in particular, this would provide details of the active site structure (testing the ChemNet predictions), and verify that it is a novel fold. Perhaps the authors have been able to solve a crystal structure in the interim? However, the AlphaFold2 predicted structure is likely to be reasonably accurate overall. It would be useful to mention briefly the expected accuracy of structure prediction for zinc proteins.

RosettaFlow looks a very useful tool, which should prove widely applicable. I was somewhat confused that a different name was used in the preprint of this work. It is not clear that the approach described, however, will be broadly applicable beyond fairly simple zinc hydrolases. Ref 33 (Ahern et al.) is crucial to understanding the basis of this method. The successful designed enzyme is a small protein with a simple and common active site motif (three histidines). During the design process,

eventually the zinc binding site is fixed, so the design procedure seems to be really a process of designing/redesigning the first and second sphere residues. This could limit the range of accessible activities as targets for design. Could this approach be used to design a more complex activity than a monozinc hydrolase? Would that require significantly more computational power? Also, (see further below), would more detailed, e.g. multistate templates be required (as in the multistate template used by Baker et al. in their recent designs of serine hydrolases)? This does not undermine the impressive work here, but is important for understanding the range of applicability.

Notably, only a single structure (for the TS of four variants of the reaction) from a density functional theory (DFT) calculation is used as the template. These TS structures are optimized with a reasonable level of DFT, and conformers are generated with lower level, approximate DFT. The authors make the coordinates of these "theozymes" available, which is good and appropriate. More details should be given about this structure in the main text, and the reasons for the choice should be stated (for example, it should be stated that it is a transition state structure, as described in the SI). The template here contrasts with the serine hydrolase work, which used several structures e.g. of different states in the reaction. Again this indicates that the main success of the design here is of second-sphere and surrounding residues, for a defined zinc site.

As with the serine hydrolase designs, it would be useful to comment on the sensitivity of the design to the template. Has this been tested? What factors make a good template? This is important to guide for future work. For example, TS structures are used as a template. This is a reasonable choice, but tetrahedral intermediate structures could be offer advantages (e.g. the stabilization by the protein would be expected to be largest for the TI).

It would be useful to compare to the serine hydrolyase design process (which applied a different, diffusion model; there is already good discussion of RFflow versus the RFdiffusion) and discuss recommendations for enzyme design, including choice of template(s)/theozyme(s). This would enable the wider community to make use of these powerful design methods effectively.

(Remarks on code availability)

Version 1:

Reviewer comments:

Referee #1

(Remarks to the Author)
In their revised version of 'Computational Design of Metallohydrolases' Kim et al. addressed pretty much all issues/questions the other reviewers and I were concerned about.

Overall, I think this is a great study, showing the capabilities of scaffolding functional groups alone in a sequences position agnostic way using RFdiffusion2. I especially would like to highlight the additional insight the authors gained from their analysis of the first round of designs and their subsequent generation of a second round with an - arguably - more complex TS. Also the added Supplementary Figs. S30-S33 are highly informative!

While I can relate to the frustrations of not being able to obtain diffraction quality crystals, even with a high screening effort, the lack of *any* global structural analysis of any of their designs is still disappointing, given that the statement 'our designs are too small for SAXS analysis' is just not true and 1H,15N HSQC samples are very easy to produce and measure.... Yet all controls show that their designs work for the right reasons and thus I think validations are ok.

Moreover, I still think that it would have been great if Kim et al. not only showed that inpainting of loops with RFJoint2 led to higher convergence in AF2 predictions, but also analyzed what fraction of their active designs came from back bones directly out of RFdiffusion2 vs. RFdiffusion2+RFjoint2, to get a better idea if this was a necessary design step to achieve high catalytic efficiencies or not.

Things I'd recommend to take another look at, before publication:

1. The MM plot of Zeta_4 in Fig. 4h shows that this titration clearly was not finished. The reported Km is above the highest measured data point. This needs to be repeated with higher substrate concentrations, or should be removed since it does not add too much to the story. Unless the authors start discussing intrinsic features of their folds and why some (Zeta_2 and Zeta_3 in particular) seem to result in higher activity designs after all, even when the sequence identity of the same back bone is fairly low (<80%).

2. Some of the reported CD-spectra show very high negative signals around the 209-nm peak - lower than what one would expect from purely alpha-helical spectra. This is fine, if there are lots of unstructured regions present in the designs as well. A conclusion which could be drawn if the spectra would have been recorded to 190 nm.

(Remarks on code availability)
Instructions to install and run the individual programs are fine.

The only issue I had was while running PLACER. Initially, I ran into chemical connection issues of the used substrate(s), but

could fix it by supplying extra ligand information in additional formats. And it seems this is a known phenomenon and addressed in the FAQs on the github repo.

Referee #3

(Remarks to the Author)
The authors provide a detailed and convincing response. They have performed an additional, second design campaign which significantly illuminates the process and findings, and strengthens the conclusions. The insightful and detailed discussion in sections 2.1 and 2.2 of the supplementary information describes the implications and approach in detail and will be very useful across the field and in future applications. They have answered all questions and points that I raised in my initial review. The renaming of the computational tools is appropriate and clarifies matters, as does the additional details on (what is now called) RFdiffusion2.

This is important, groundbreaking work and is suitable for publication in Nature essentially as is.

There is a minor but important error in the SI: the term ChemNet is still used in places (e.g. p. 25 and elsewhere). Please ensure that the terminology/naming is updated for the computational tools throughout.

A minor but interesting question is whether there is any indication of spodium bonding or similar effects, as found to be important in some natural zinc enzymes (e.g. insulin-regulated aminopeptidase, IRAP) if indeed predicted structures can capture such effects).

Very minor is that the symbols for kcat, KM in Fig. 4 are very small.

(Remarks on code availability)

Version 2:

Reviewer comments:

Referee #1

(Remarks to the Author)
In their revised version of 'Computational Design of Metallohydrolases' Kim et al. addressed all my previous comments and exceeded my expectations by adding an apo and a Zn-bound structure of their most active design. Both structures are in good agreement with their design model. I want to thank the authors for their additional efforts and congratulate them to this impressive study! The additional analysis and insights through it are of high importance to the field.

(Remarks on code availability)
for a previous version. There has been no changes to the computational approaches reported here.

Referee #4

(Remarks to the Author)
This reviewer has been asked to comment on the structural data added to the revisions of “Computational Design of Metallohydrolases” by Kim et al.

The premise of the manuscript is to use a new generative AI-based algorithm to design functional zinc hydrolases. Indeed, the algorithm successfully design both a first- and second-generation zinc-dependent hydrolase. The structure of one, ZETA_2, was determined in the apo- and Zn-bound forms by X-ray crystallography.

The apo structure agreed with the design with an RMSD of 1.1 Å, revealing a pre-formed zinc binding site. The crystal form in the P2 space group could not be co-crystallized in the presence of high concentrations of zinc because that resulted in precipitation. The designed tetrahedral binding site appears zinc-specific, but no metal competition experiments were shown to analyze the degree of this specificity.

The zinc-bound structure was determined by soaking the crystals in high zinc acetate (250 mM), which revealed a zinc bound at the expected site. A second zinc forms a crystal contact, perhaps explaining the higher resolution of this structure. Binding the second zinc required flipping of one of the designed histidine ligands and changed the space group to P2(1)2(1)2(1).

The alignment of the fold to the designed fold supports the authors' claims about the potential for their AI method as a useful tool for designing novel metalloenzymes with native-like activities in a streamlined fashion. That the most active design is a helical bundle may not be all that surprising given that more labor-intensive de novo designs from all the way back to the early days of protein design were also helical bundles. Nevertheless, those designs required quite a lot of a priori knowledge not needed for this new method, so it represents a significant step forward.

The discrepancy between the apo and zinc-bound structures and the adventitiously bound zinc at a crystal contact brings up

a few questions. Some are easily addressed by including a little more detail or minor edits, others might be better suited for a detailed follow-up study. My opinions about the importance of the comments are added in parenthesis.

1) Are the biophysical analyses (i.e. SEC studies (Fig. S8) and CD studies (Fig. S29)) performed on the apo or metal-bound structure? (Buffer compositions could simply be included in the figure legend.)

2) How many metals are bound in solution? (editor's purview)

3) How specific for zinc is the designed scaffold? (editor's purview)

4) Is the protein oligomeric state metal-dependent in solution, and does this impact the enzyme activity? (editor's purview)

- Points 2 and 3 address a larger question about the design process: can off-design possibilities be addressed in the design stage? That is, are there residue variants that could be built that inhibit this other zinc? To address this, the zinc binding and protein oligomerization need further experimental characterization.

5) Why is the His33 rotated ever-so slightly so its coordination to the zinc is not quite ideal? (additional clarification in the text might be warranted)

6) Are all the supplemental figures cited? For example, I cannot find Figs S2, S17, or S19. I also do not think they are called in the order presented in the supplemental document (please check carefully)

7) Fig. 5's legend needs a small edit – panels b and c are called together, then (b) is explained but (c) is not. (minor edits) "(B,C) Active site zoom-in highlighting the agreement between the experimental and designed catalytic geometry (B) and the shape-complementarity of the designed binding pocket and the transition state model (superimposed in from the design model). Crystal structure shown in grey and design model in color in all panels."

M. ELizabeth Stroupe

(Remarks on code availability)

Open Access This Peer Review File is licensed under a Creative Commons Attribution 4.0 International License, which permits use, sharing, adaptation, distribution and reproduction in any medium or format, as long as you give appropriate credit to the original author(s) and the source, provide a link to the Creative Commons license, and indicate if changes were made.
In cases where reviewers are anonymous, credit should be given to 'Anonymous Referee' and the source.
The images or other third party material in this Peer Review File are included in the article's Creative Commons license, unless indicated otherwise in a credit line to the material. If material is not included in the article's Creative Commons license and your intended use is not permitted by statutory regulation or exceeds the permitted use, you will need to obtain permission directly from the copyright holder.
To view a copy of this license, visit https://creativecommons.org/licenses/by/4.0/

Dear Angela,

Thank you for the constructive feedback on our manuscript entitled “Computational Design of Metallohydrolases”. We have addressed the reviewer suggestion and believe that the revised manuscript has been significantly strengthened. We have significantly strengthened the conclusions of the original manuscript by carrying out a second design campaign from an updated set of quantum chemistry theozymes. From a new set of 96 designs, we identified several highly active enzymes across diverse scaffolds with the best design outperforming the catalytic efficiency and rate enhancement of the previous best design, A1. These results are highlighted in the newly added Figure 4 of the main text alongside several other supplemental information figures and methods.

Each reviewer suggested that a structural biology study of the most active design during the time of submission, A1 (ZETA_1), would greatly improve the manuscript. We have been working rigorously toward this end with crystallography but we have not yet solved a crystal structure. We have had to concentrate A1 to >45 mg/mL to obtain crystals due to its very high solubility and we have screened several mL of these samples over an 8 month span over hundreds of conditions. A challenge has been that raising the $ZnSO_4$ concentration to match the protein stoichiometry at this concentration has consistently led to aggregation and precipitation, and we have failed to obtain Zn(II) containing crystals diffracting to high resolution.

Below we provide a detailed response to the reviewer comments. We note that RFflow is now named RFdiffusion2 and ChemNet is now named PLACER. We refer to the software by these names throughout the rebuttal letter and updated manuscript. Additionally, A1 is now referred to as zinc metalloesterase 1 (ZETA_1). The manuscript describing the training and benchmarking of RFdiffusion2 has now been posted in the *bioRxiv* (Ahern et al. *bioRxiv* **2025**) and has been submitted to *Nature Methods*. Please refer to the link below:
https://www.biorxiv.org/content/10.1101/2025.04.09.648075v2

We hope that you believe, as we do, that the manuscript is now suitable for publication in *Nature*.

Sincerely,

David Baker, Don Hilvert, Seth Woodbury, Donghyo Kim

Referee #1:

Summary of the key results:

In their manuscript, entitled 'Computational Design of Metallohydrolases' Kim et al. detail the use of an unpublished tool, RFflow, to scaffold the active site of a zinc-dependent hydrolase. In contrast to previous studies, they could start from a minimal quantum-chemistry generated active site description. The authors elegantly show that their approach yielded five active metallohydrolases in a set of 96 designs, one of which exhibits rate-accelerations orders of magnitude higher than previously reported designed metallohydrolases.

Kim et al. highlight the big advantage of their sequence position agnostic design approach, which simplifies the whole process to scaffold-calculated transition states. They also stress the possibility of enhanced backbone sampling and make it clear that detailed knowledge about rotameric states of sidechains is obsolete with their approach. Similar to other recent preprints, Kim et al. also use a hybrid ML and energy-based sequence design approach to find optimal structure-sequence pairs.

In addition, they report on the importance of ChemNet (available as preprint, no code available) for the selection of designs in combination with more 'classic' (Rosetta-based) metrics.

We appreciate the thorough summary provided by reviewer 1 and thank them for the kind words about our manuscript. Indeed, we wanted to emphasize that RFdiffusion2 (previously called RFflow) does not require prespecified rotameric states or sequence position for input amino acids. This is ideal for applications such as enzyme design, where the user is focused on the primary interactions between the transition state and sidechain functional groups as an input for the generative model.

We included the code for ChemNet in the zip file provided to the reviewers. However, the code for ChemNet has now been made publicly available on GitHub, under the new name, PLACER (Protein-Ligand Atomistic Conformational Ensemble Resolver). We updated the manuscript to reflect the new name and direct readers to the GitHub repository (https://github.com/baker-laboratory/PLACER).

To verify their findings, the authors performed thorough biochemical analysis of their designs, including detailed kinetics of variants of their most active design, which is one of the most important aspects of the manuscript.

Originality and significance:

This study elegantly highlights the potential of de novo enzyme design and introduces the use of a new ML-based backbone generator. While I believe that this work can become suitable for publication in Nature, it appears to be incomplete in its present form and a little 'premature', with all the focus on RFflow, without RFflow described in detail. In addition, the manuscript is very short und lacks substantial detail in the discussion of the transition state calculations, hypothesis-driven interpretation of the results (besides A1 as the best design), holistic insights and detail on the analysis of the activity data and, most importantly, lack of biophysical and structural data of the designs. If the authors can address these shortcomings, I recommend publishing this study in Nature.
In its present form though, I suggest including this as a terrific experimental validation of the yet

unpublished RFflow manuscript, or submitting it to a more specialized journal, such as Nature Chemistry or Nature Catalysis.

We revised our manuscript in several ways to address these concerns:

(1) We noted several observations and trends among the design models of the 5 active hits and wrote a highly detailed analysis in the supplementary information (see SI section 4.2, "Observations and trends from the 1st design campaign"). Briefly, we observed trends regarding the oxyanion stabilization, the enantiomer of the hydroxide attack, the Zn(II) coordination from histidine, the substrate conformation, the general base positioning, and the sequence length of the scaffolds.

(2) We used this information to create a new set of theozymes for DFT (Fig. 4a; described in more detail in SI section 4.2 "Theozyme preparation…"). We then used these theozymes to perform another design campaign where we tested a new set of 96 designs and saw substantial improvements in activity and success rate. Notably, we obtained 11 major hits among 3 unique scaffolds: 5 designs had a k_{cat}/K_M >10^4 M^{-1} s^{-1} and 6 designs had a k_{cat}/K_M >10^3 M^{-1} s^{-1} (Fig. 4B,D-E). The best design, ZETA_2, had a k_{cat}/K_M = 53,000 ± 5000 M^{-1} s^{-1} and a k_{cat} = 1.5 ± 0.1 s^{-1}, a 3-fold increase over the k_{cat} of ZETA_1 (A1). These results are summarized in new Figure 4 (see part of Fig. 4 below), and further demonstrate that our design pipeline is robust and reproducible. We also added 14 new supplemental figures regarding this second design campaign.

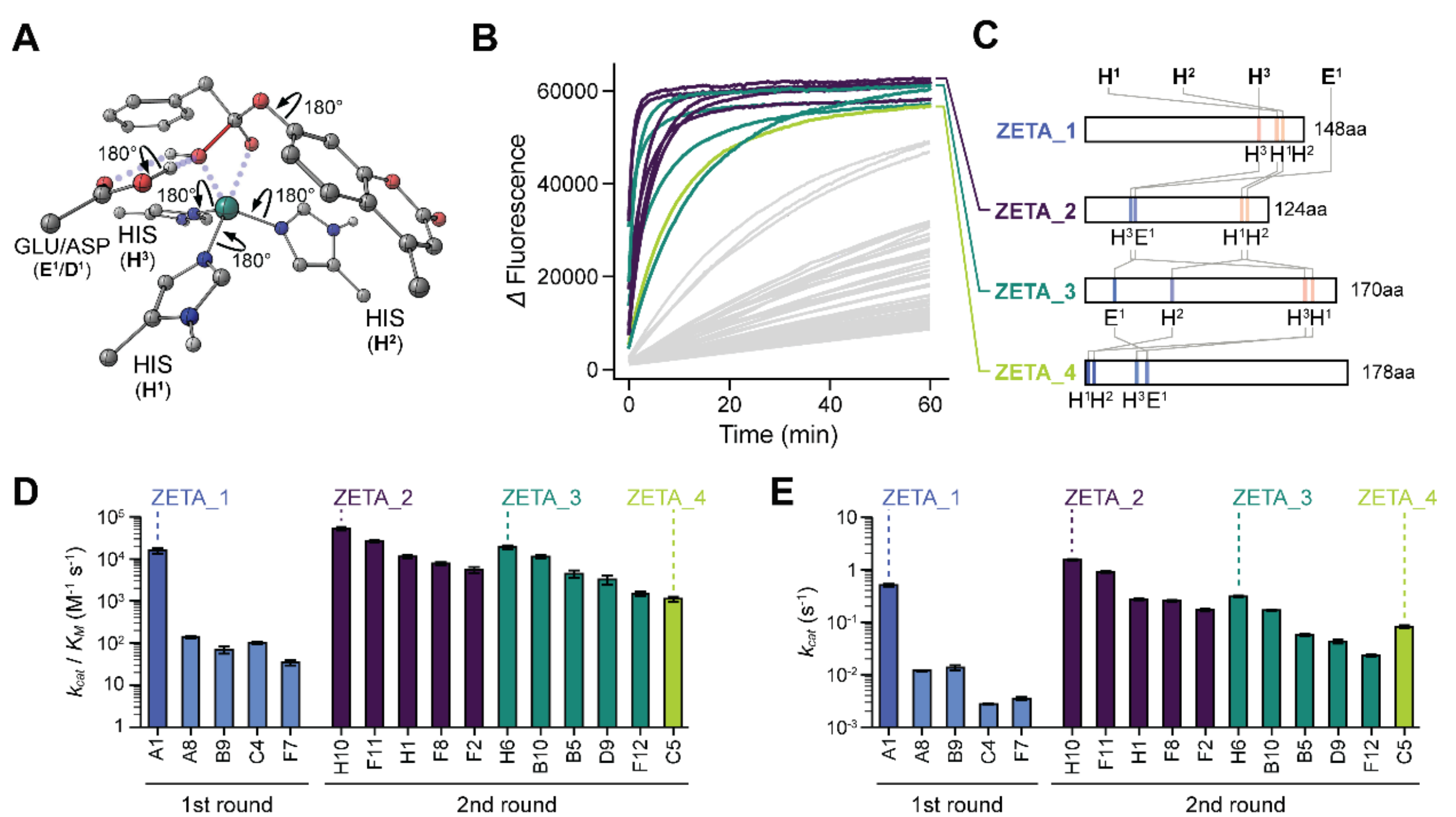

Figure 4: (**A**) Second round DFT theozymes containing the catalytic base. Zn(II) is coordinated by histidine Nε atoms in these theozymes and thus Cβ is explicitly modeled. (**B**) Reaction progress curves colored by scaffold family. (**C**) Sequence length and catalytic residue positioning for the top design in each scaffold, named ZETA_2-4. These designs differ from each other and from ZETA_1. (**D**,**E**) Michaelis-Menten (**D**) k_{cat}/K_M and (**E**) k_{cat} for the 11 second round hits with colors corresponding to scaffolds using the scheme in (**B**,**C**).

(3) As noted above, crystallization trials were unsuccessful. However, we added circular dichroism (CD) data for each of the 4 other hits from the first round of designs and the best design from each of the 3 unique scaffolds present in the 11 major hits from the 2nd round (i.e., ZETA_2-4; Fig. S29). In total, this is CD data for 7 unique scaffolds (8 including ZETA_1). All of the CD scans suggest that the designs have the correct secondary structure as designed, which is alpha-helical in most cases.

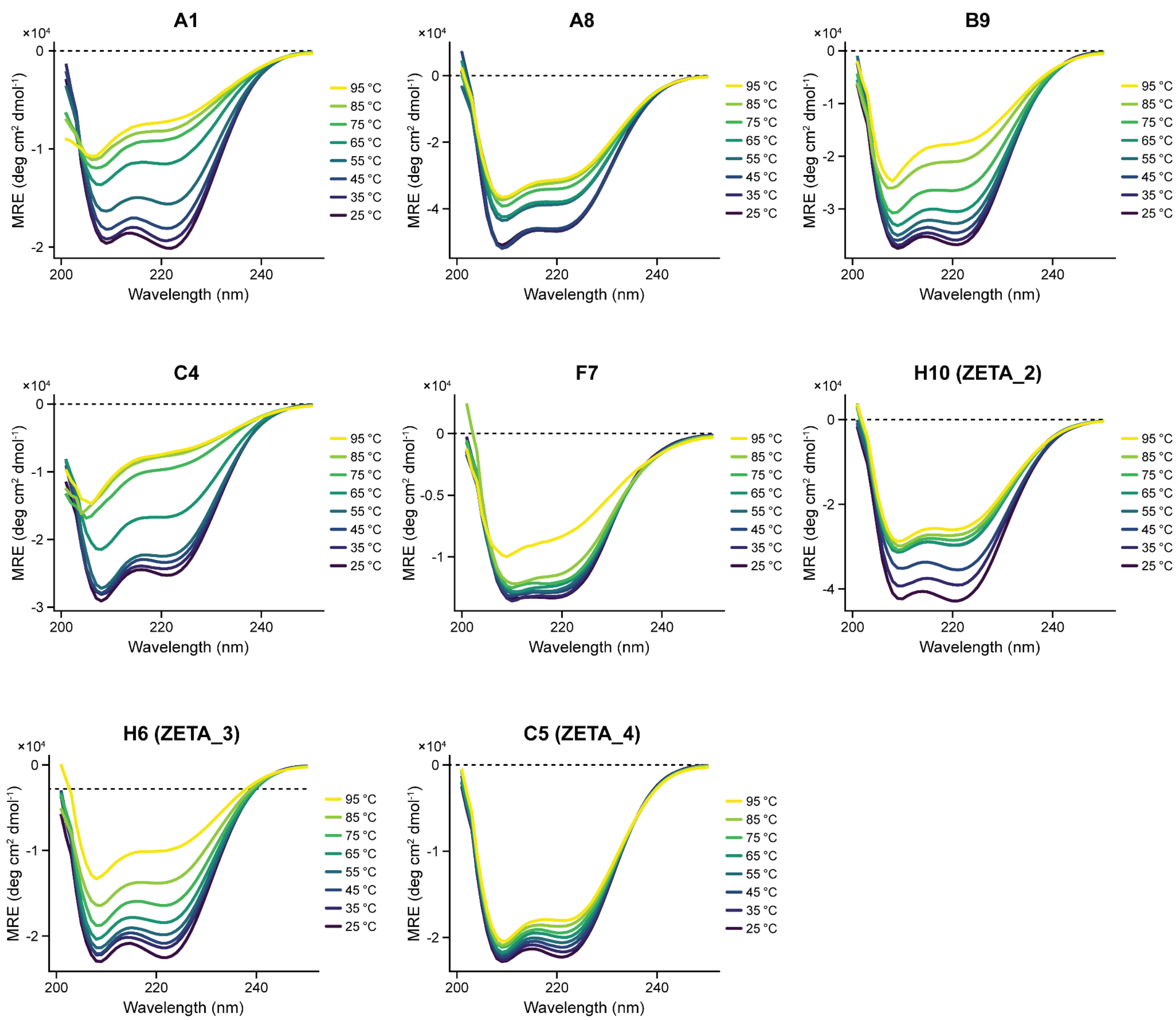

Fig. S29. Circular dichroism spectra for all characterized enzymes at increasing temperatures. A1 (ZETA_1), A8, B9, C4, and F7 are from the first design campaign. ZETA_2-4 are from the second design campaign. The CD spectra were recorded in 10 °C increments from 25 °C to 95 °C. In all cases, the CD spectra are consistent with the expected secondary structure from the design models, which is alpha-helical in most cases.

(4) We added a new section in the supplementary information entitled “Extended Discussion and Analysis” (see section 2) which details the unique features of the active designs from the design model perspective and the computational metric perspective.

Overall comments on the manuscript:

Kim et al. convincingly show the benefit of having a sequence position agnostic backbone generation tool at hand, which can be used to scaffold a minimal active site description coming from quantum chemistry calculations (minimal in this case means starting from a subset of atoms of the individual side chains involved in stabilizing the transition state). The argument that exhaustively searching the astronomically large space of backbone geometries around an active site is impossible with previous approaches, is valid. However, there have been recent preprints that made use of RFdiffusion and similar approaches, and they arrived at similar results, for different reactions, while screening a similar or even smaller number of designs. With the additional step of using RFJoint to subsample loop regions, etc. it is not quite clear if Kim et al.'s approach is more efficient than what people used before. It would be beneficial to have statistics on sampling and running times to be able to compare the efficiency of individual approaches.

We know of two recent preprints/publications in enzyme design that come to mind in this context: (1) Lauko et al. *Science* **2025** and (2) Braun et al. *bioRxiv* **2024**. We will briefly critique these recent preprints/publications in enzyme design to argue what novelty and advantages that ours offers.

(1) The recent *Science* publication by Lauko, Pellock, Sumida et al. entailing the design of serine hydrolases using RFdiffusion indeed yielded impressive de novo serine hydrolases, but it required many rounds of experimental iteration, sequence optimization, partial redesign, and refinement of their methods, especially with motif generation (see their methods section). They had to generate 10,000 backbones at one point using RFdiffusion for the single purpose of obtaining more backbone substructures that could host the oxyanion hole, which they used as motif inputs into additional design campaigns, implying that they had to generate several 10,000s of backbones in total to achieve their results.

For comparison, we made <5100 backbones once using RFdiffusion2 for the entire 1st design campaign, which yielded all the designs and results of this paper for Fig. 2-3. This is a noteworthy computational speedup and great simplification of the design process, which did not require any experimental iteration. We made a similar number of scaffolds in the 2nd design campaign and achieved even better results from that order of 96 designs. It is important to note that none of the results achieved in this manuscript came from redesigned scaffolds that were demonstrated to work, as was the case with the serine hydrolases.

We obtained significantly higher k_{cat} values for the best metallohydrolase designs compared to the best serine hydrolase designs (momi120-103 serine hydrolase has a k_{cat} = 0.057 s^{-1}; ZETA_1 metallohydrolase has a k_{cat} = ~0.5 s^{-1}). Furthermore, when we performed a second design campaign from the updated theozymes our new best design displayed an additional 3-fold improvement in k_{cat} (ZETA_2 k_{cat} = 1.5 s^{-1}) demonstrating that we were able to improve the rate enhancement. In contrast, the serine hydrolase momi120-103 redesign of momi120 achieved its high catalytic efficiency through decreasing K_M rather than increasing k_{cat}, which is undesirable and just indicates that it binds the substrate tighter but without a catalytic rate enhancement.

(2) The recent preprint from Braun & Tripp et al. introduces a pipeline called Riff-Diff that uses RFdiffusion to generate de novo backbones around catalytic residues that are pre-grafted onto idealized alpha-helices. They used this pipeline to design retro-aldolases from a catalytic tetrad of a previous retro-aldolase crystal structure and ordered a single batch of designs (<48), several of which had remarkable activity and rate enhancement. These were excellent results, but the authors' justification for pre-grafting the motifs was that the "success rates with RFdiffusion have been shown to correlate with increasing size of the selected motifs," which

suggests that RFdiffusion necessitates more structural constraints to perform competent inference. We show precisely the opposite with RFdiffusion2: we can specify even less structural information and search through more diverse structural space while still achieving extremely competent scaffolds. Many native enzymes have complex secondary structural features around their active site, such as beta sheets and loops, which RFdiffusion2 can generate for the catalytic residues but Riff-Diff may struggle with due to the idealized helices. Indeed, we had 2 active designs from the first design campaign (A8 and F7) that have at least one catalytic histidine on a beta sheet (Fig 2A).

If reviewer 1 knows of another publication/preprint relating to enzyme design that they believe to arrive at similar conclusions, we would be happy to address it.

In addition to simplifying the design problem, the real interesting finding here is that the 'best' design also displayed the highest activity by a large margin. The authors do a good job in analyzing this particular design. Yet, the study falls short in a comparative analysis of the best (A1) design vs. the less active designs vs. the inactive designs (e.g. knock-out mutants of all active designs similar to the ones made for A1). Given that all designs were selected according to the same criteria, this dataset holds great potential to decipher intricate details about the origin of catalysis in metallohydrolases.

We added a section to the supplemental information, section 2, which contains multiple subsections of this requested analysis from a computational metric perspective and a structural design model perspective. We perform this analysis for the results of the first design campaign and the new second design campaign. Additionally, the datasets, sequences, and design models will be publicly available upon publication.

Suggested improvements:

• The manuscript would benefit tremendously from a more detailed biophysical and structural analysis of at least the most active design. Ideally, the authors would determine a crystal structure of the design, to have experimental proof about their claims of pre-organization and design accuracy. I understand that this is a lot to ask, so at least a more detailed solution-based study should be performed – this includes SAXS and CD data on the active designs. As far as CD is concerned, it would be good to show temperature ramp plots, too. Fig. 3 of the manuscript currently holds the only structural information, which are CD spectra at 20C and 95C. However, without the actual temperature ramp there, it is hard to judge from this plot if this protein is stable or a molten globule, since it loses a significant amount of CD signal at 95C but refolds after cooling back down. An even better proof of proper foldedness with and without the addition of Zn would be to record HSQC spectra for the active designs. The His peaks should easily be identifiable, if the peaks are well dispersed.

As noted in the introduction, despite considerable effort, we were unable to obtain crystals diffracting to high resolution. Our designs are too small to perform SAXS and we also did not obtain HSQC spectra. However, as the reviewer suggested, we repeated the CD experiments and measured the temperature melting for ZETA_1, shown below and in Fig 3D, which shows a broad transition with a melting temperature around 60C.

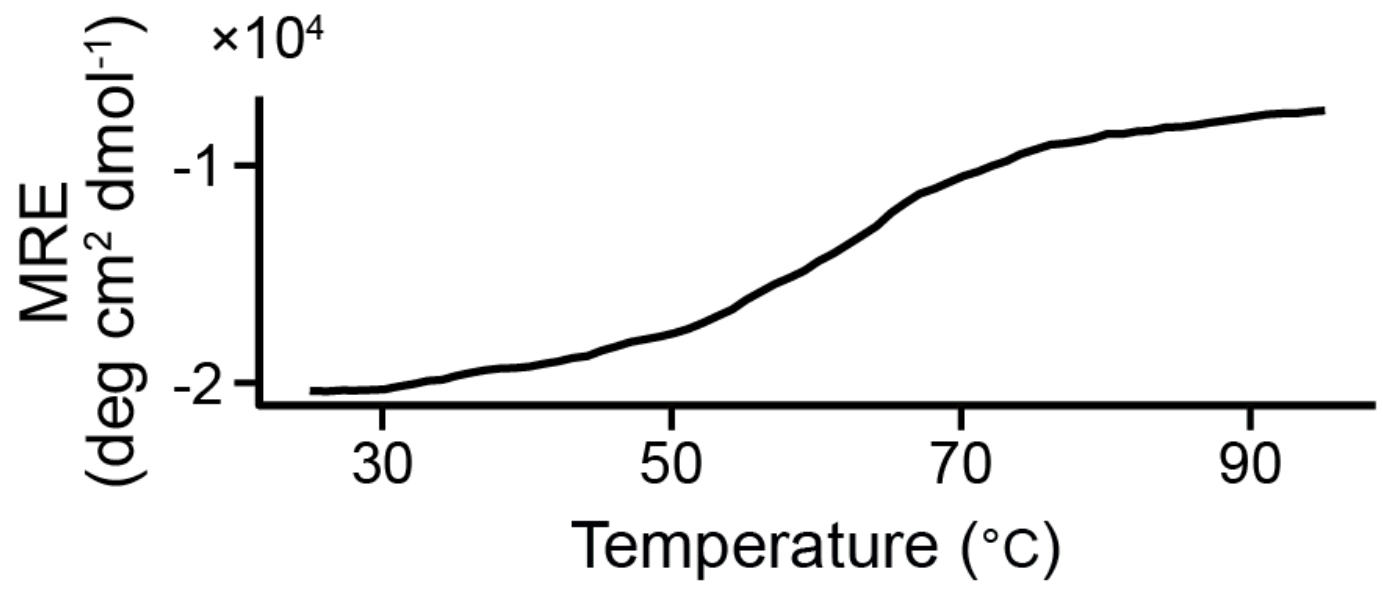

Fig. 3. (**D**) Circular dichroism signal at 222 nm measured every 1 °C and plotted as a function of temperature.

• For the active designs, it would be great to know which of the four transition states they originated from. Is there a trend? Could it be that one of the QM-based TS is superior than the others for scaffolding? If yes, can the authors identify why this might be?

We analyzed this and other trends from the active hits in the first round, see section 4.2 "Observations and trends from the 1st design campaign" which we excerpt below:

"(1) All of the hits except for F7 came from the class of DFT theozymes using Zn(II) to stabilize the oxyanion, rather than 2 protein hydrogen bonds. Perhaps this was because it was difficult to precisely position 2 protein hydrogen bonds, either from sidechains and/or backbones, to the substrate oxyanion hole during sequence design after the scaffold had already been generated. Even in the case of F7, the design model only predicts 1 hydrogen bond from a serine residue to the oxyanion, not 2 as intended by the DFT theozyme. We allowed these cases, like F7, to pass since it was extremely rare to get a well-behaved design with 2 hydrogen bonds in place stabilizing the oxyanion."

"(2) Excluding F7, the 4 hits using Zn(II) to stabilize the oxyanion were derived from the DFT theozyme of the S- enantiomer of the hydroxide attack. That is, the design models of A1 (ZETA_1), A8, B9, and C4 all contain the S- enantiomer of the hydroxide attack. F7 models the R- enantiomer of the hydroxide attack, but note that it was designed for a different mode of oxyanion stabilization."

"(3) The design models of every hit coordinate the Zn(II) with the N_ε atom of the His residues, with the exception of B9 which has 2/3 His residues coordinating Zn(II) with their N_ε atom and the other His residue coordinating with its N_δ atom. The all-N_ε atom coordination and 2/3 N_ε atom coordination geometries for zinc hydrolases with three Zn(II)-coordinating His residues are very common (e.g., PDB: 1AST (pdb_00001ast); 2VVB (pdb_00002vvb))."

"(4) The scaffold for A1 (ZETA_1) was generated around its theozyme with the exact conformation from DFT (i.e., NOT from one of the extra conformers generated from the original DFT model using GFN2-XTB semiempirical QM). However, during FastMPNNdesign, Rosetta chose a different conformer with the 4MU flipped approximately 180 degrees. Nonetheless, this is almost an equivalent geometry in terms of the interactions that were optimized during DFT such as pi-stacking with the His residues. The models of the other hits also had the substrate approximately in the geometry that was optimized out of DFT, rather than one of the more drastically-different conformers. We interpreted this to suggest that designing around the exact DFT theozyme, instead of a different conformer of the DFT theozyme, is optimal and that any conformational sampling should be done at the DFT step (i.e., creating different conformer input

models for DFT). This better ensures that the energy is minimized for each theozyme complex of the different conformers (i.e., interactions are optimized)."

• The section about using RFJoint to diversify the folds needs more explanation. It is not clear why this is necessary at all, if RFflow is superior to previous methods. Was it essential for making better pockets? Upon examination of some of the design models that were not ordered by the authors (thanks for providing them), it looked as if most of the RFflow-generated pockets were heavily buried/occluded. Was the RFJoint step necessary to make them more accessible for substrate binding? If yes/no please clarify.

We used RFJoint (inpainting) to diversify and idealize loop regions away from the active site while not touching any regions in direct contact with the active site. It is computationally inexpensive compared to RFdiffusion2, RFdiffusion, and partial RFdiffusion and leads to greater AF2 passing rates (Peer Review Figure 1). RFjoint modified scaffolds were added to the original RFdiffusion2 scaffolds for sequence design (e.g., 1000 parent scaffolds + 4x1000 inpainted variations = 5000 scaffolds for sequence design). RFJoint was not necessary to make the scaffolds more accessible for substrate binding; as stated above, it is not meant to modify the pocket around the substrate, so it did not make significant modifications to the burial/occlusion of the substrate. We added context in the supplemental information where this method is mentioned to clarify the purpose of RFJoint.

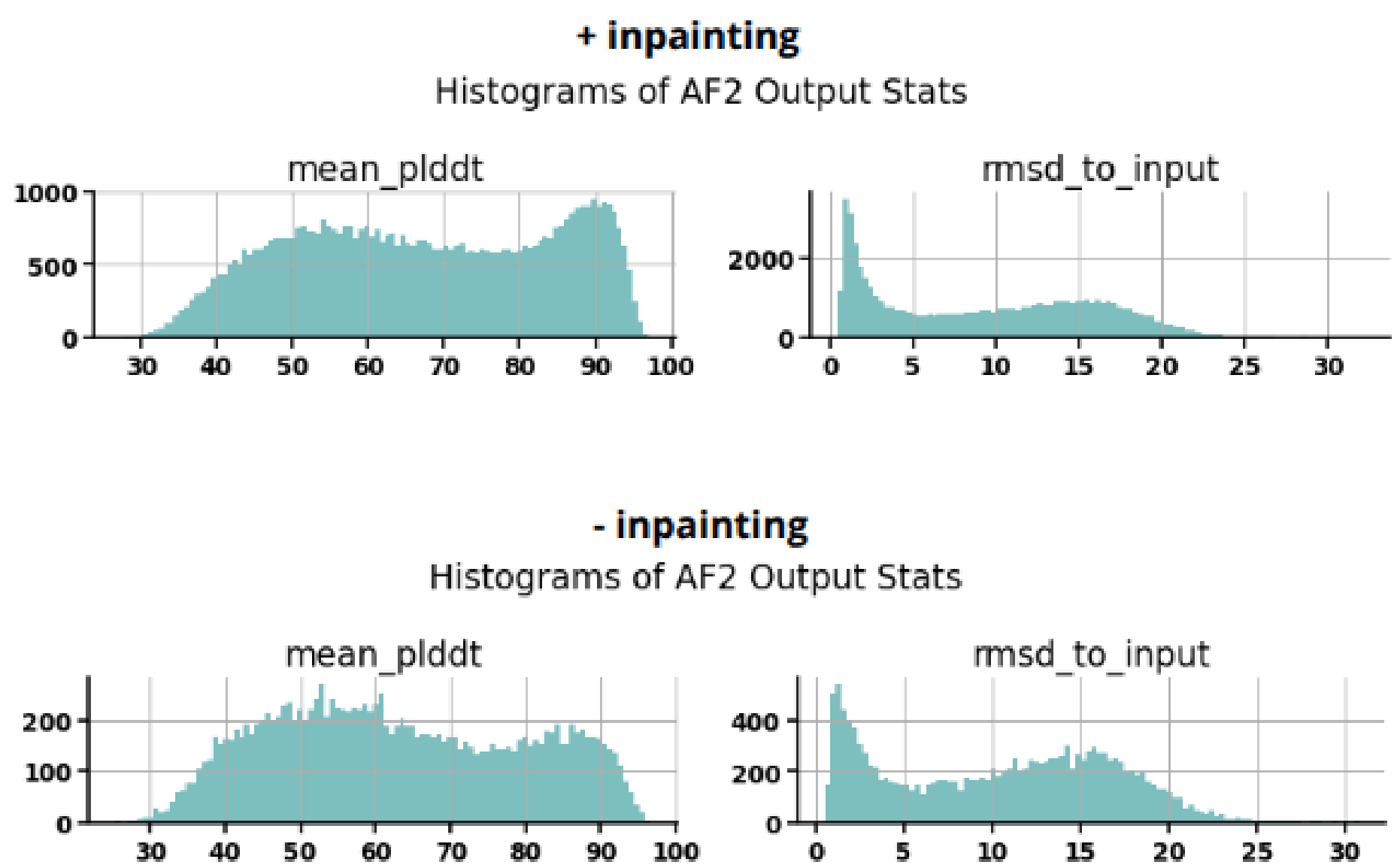

Peer Review Figure 1: AlphaFold2 (AF2) statistics for sequences with RFdiffusion2 designed structures that were inpainted (+ inpainting; top) and not inpainted (- inpainting; bottom). These data are from the 1st design campaign. The mean pLDDT distribution shifts higher and the RMSD to the design distribution shifts lower for structures that were inpainted.

• The filter/selection criteria for the initial rounds of designs (plddt, pTM, etc) are rather bad/low and need a more extensive explanation. Previous studies mostly focused on generated backbones with higher/better values from the beginning on. The strategy described here is very interesting but needs more explanation.

We appreciate this reviewer's very detailed review of our manuscript and code. We decided to be lenient with the AF2 filtering at this step because iterative ligandMPNN and Rosetta

FastRelax was going to take the passing AF2 structures (with the metal-substrate complex copied in) and significantly change their sequences and adjust the structures to reposition the catalytic machinery in the right place and optimize the active site. Thus, we wanted to give as many scaffolds a chance at this process as possible, as long as they were able to initially fold into approximately the right shape. Additionally, we noticed that many larger designs (> ~220 amino acids) tended to have worse AF2 metrics on average, but comparison with the designed structures many times revealed a decent looking pocket/catalytic arrangement. Thus, we thought making the filters more lenient would be accommodating for larger and more sophisticated scaffolds, which may be predicted less confidently in AF2. However, we were much more strict about these metrics in the second design campaign, partially because our average scaffold size was much smaller. We added detail in the supplemental information to briefly explain this rationale.

• The argument of why the general base was put into the TS calculation, but removed from the minimal active site during scaffolding is not clear to me. Were the backbones that RFflow produced much worse when the general base residue was included? The same applies to the H-bonding residues (for the applicable TS).

In retrospect, we agree with the reviewer that this was suboptimal and we now state that future design campaigns should specify the general base from the beginning (or H-bonding residues). At the time, our reasoning was that since Asp/Glu/His could all potentially serve as general bases, it would be more efficient to sample these combinations during sequence design and iterative active site optimization (FastMPNNdesign) within the context of scaffolds. In fact, we did apply a positive bias toward these amino acids for ligandMPNN at positions near the hydroxide to promote the installation of a general base, and we did select the designs based on the presence of such a residue. However, we did not expect the positioning of the general base (or H-bonding residues) to be so delicate. Now it is clear to us that even an angstrom or sub-angstrom shift in the position of the general base or H-bonding residues can dramatically hinder their ability to contribute to catalysis, and thus the positions of these residues should be fixed from TS calculation. We explicitly acknowledged the limitation of the poorly positioned general base in section 4.2 of the SI entitled “Observations and trends from the 1st design campaign.”

To further address this concern, we explicitly modeled the general base (Glu/Asp) as part of the motif for RFdiffusion2 in the second design campaign. This guaranteed its positioning during scaffold generation and allowed it to be kept fixed during the pipeline. The scaffold quality did not notably diminish and we had much better success in the results of the second design campaign order of 96 (see Figure 4).

• A more in-depth analysis and discussion of why other sequences on the same backbone as the A1 design were not active, would be very interesting. Do the metrics of these alternative sequences differ a lot (e.g. in the authors composite score, AF2, ChemNet, etc)?

We agree that this analysis should be useful and thus we added a new chapter to our supplemental information entitled “Extended Discussion and Analysis” (see section 2). Within this section, we provided two-fold discussion about what makes A1 (ZETA_1) unique from the 4 other hits and its other variants: (1) discussion from analysis of the design models, and (2) discussion from the computational metrics. We additionally provide this discussion for the new design campaign results as well. Finally, we provide this discussion for the collectively pooled results over both design campaigns. We thank Reviewer 1 for this suggestion and believe that it

significantly strengthens the manuscript.

• What is the speed of the background reaction in water? A comparison over kcat vs kuncut would be beneficial in light of the many inactive designs.

We now have explicitly provided k_{uncat} = 0.000021 ± 0.000001 s^{-1} in the supplemental information (see section 4.3 entitled "Kinetic analysis"). You can see the linear fit for it below and the reaction traces. We provide the rate acceleration (k_{cat}/k_{uncat}) in every Michaelis-Menten plot shown in this manuscript (Fig. 2C; Fig. 4F-H; Fig. S10; Fig. S23), except for the ZETA_1 knockouts and in Table S3 for ZETA_1-4.

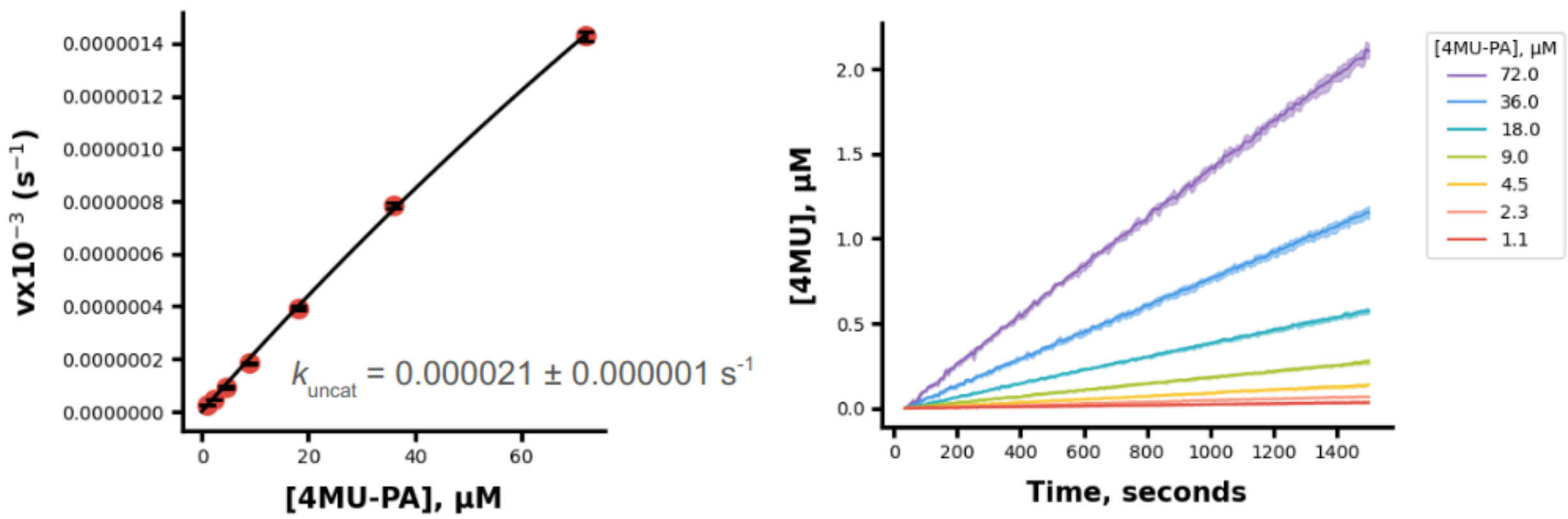

Peer Review Figure 2: Uncatalyzed hydrolysis reactions for 4MU-PA in the reaction buffer conditions. (Left) Linear fit for the initial velocities used to obtain k_{uncat} and (right) the traces for the reactions at different substrate concentrations.

• A comparative discussion of how the best design compares to natural metallohydrolases would provide a more complete picture about how good the best design really is.

In the discussion section of the main text we mentioned that "the catalytic efficiencies of ZETA_1-3 are within the range observed for native metallohydrolases (k_{cat}/K_M = 10^4~10^8 M^{-1} s^{-1})" where we cite Ref 46-50 and Table S3 with select examples. Although these enzymes are on the lower side of catalytic efficiency in comparison to native metallohydrolases, they are far better than previously designed (metallo)hydrolases prior to optimization (Table S3).

• It is surprising that the triple-H variant still shows high affinity for Zn. Thus, the authors should either fit their titration curves of this variant to a two-binding site model or perform other experiments, such as native MS, to decipher the actual number of Zn binding sites of A1.

This was also a surprising result to us. We believe that there might be some weak promiscuous binding on the surface near our C-terminal streptactin tag that we expressed our designs with, which is predicted with moderate confidence by Chai-1 (Peer Review Figure 3). This promiscuous surface Zn(II) binding is predicted with higher confidence in the triple histidine -> alanine knockout Chai-1 model predicted with 1x Zn(II); in contrast, the Chai-1 model of the wild-type enzyme predicted with 2x Zn(II) lacks confidence in the predictions of both Zn(II) at once, and the predicted models with both Zn(II) ions in the active site do not look physically reasonable (the second Zn(II) lacks proper coordination).

These Chai-1 results also provide us with reasonable evidence to assume that the two Zn(II)-binding sites, if there are two at all, would be independent since they are likely to be in

different regions of the protein. This allows us to fit 2 binding sites, with one binding site being set to 330 nM (corresponding to the triple-H mutant Kd for the surface site), and the other active site binding site being set as a fit parameter. These results do not deviate significantly from the currently reported data with only 1 binding site (Peer Review Table 1).

Mutant	Zn(II) Kd (1:1 Fit) (nM)	Zn(II) Kd (2:1 Fit) (nM)
ZETA_1 (A1) Wild Type	41 ± 5	41 ± 6
Triple H->A Knockout	330 ± 30	n.a.
H130A	58 ± 6	57 ± 8
H134A	105 ± 11	108 ± 16
H118A	55 ± 6	54 ± 2
N17A	36 ± 4	36 ± 2
D67A	27 ± 3	27 ± 2

Peer Review Table 1: Active site binding affinity for Zn(II) of ZETA_1 and its knockout mutants for different fit models of Zn(II):protein binding stoichiometry (1:1 and 2:1). Note that the 2:1 fit model assumes an independent, promiscuous surface binding Zn(II) site with a Kd of 330 nM.

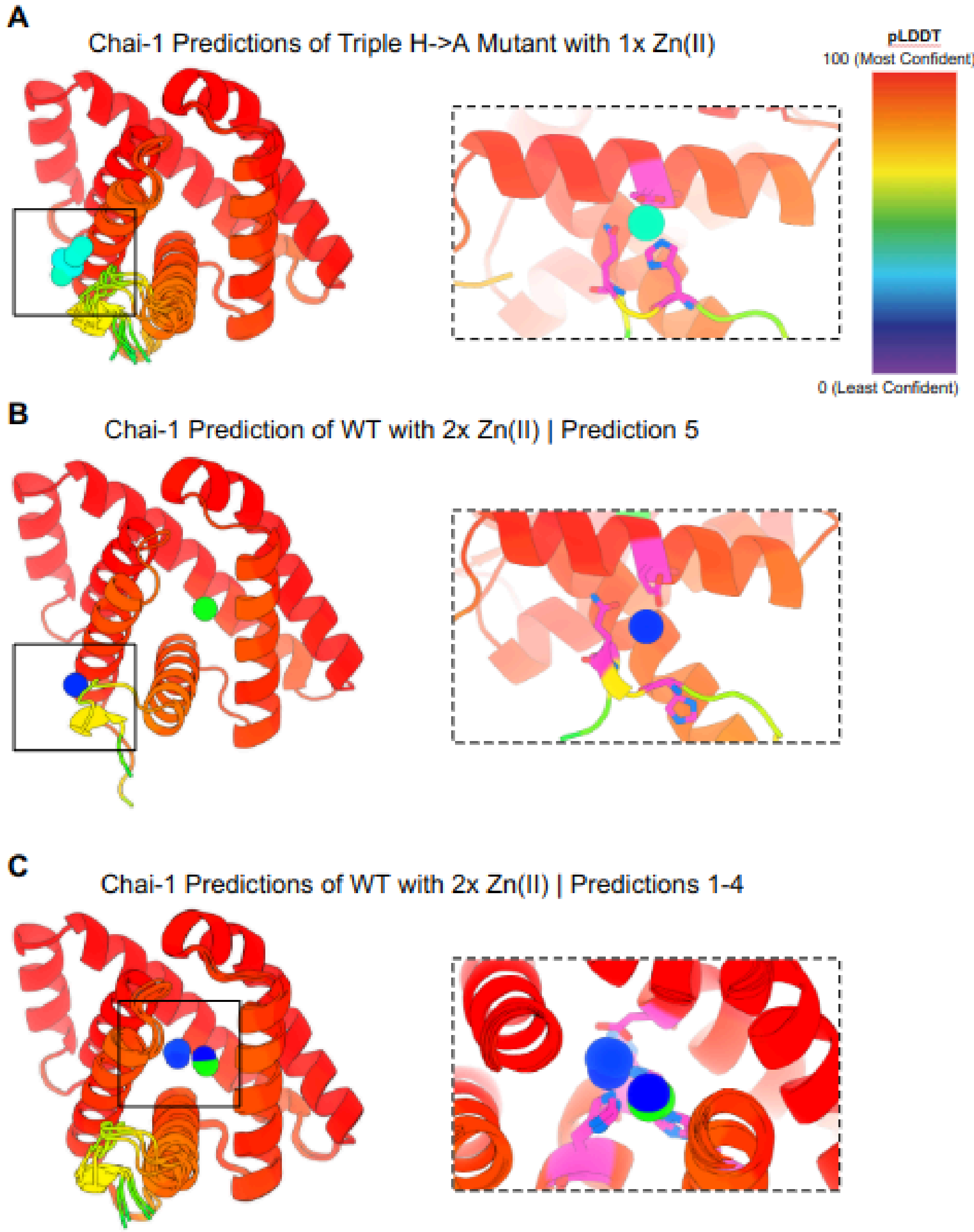

Peer Review Figure 3: Chai-1 predictions of the expressed ZETA_1 (**A**) triple H->A knockout mutant predicted with 1 Zn(II), (**B**) the wild-type predicted with 2 Zn(II) ions, one of which is on the surface near the C-terminal streptactin tag, and (**C**) the wild-type predicted with 2 Zn(II) ions both in the active site of the enzyme. The scaffold and Zn(II) ions are colored by pLDDT (local confidence), the magnified active site residues shown in sticks (right) are just colored pink except for heteroatoms. The pLDDT of the 1x Zn(II) in (A) is approximately 53, moderately confident. The pLDDT of the 2x Zn(II) ions predicted in the wild-type enzyme is very low, the properly positioned one that is coordinated by the His residues is about ~60 but the other ones are predicted with <40 pLDDT; interestingly, predicting with 2x Zn(II) in the wild-type drastically lowers the confidence of the catalytic Zn(II) than if the structure is predicted with 1x Zn(II). We believe that these results likely suggest that there may be some weak promiscuous Zn(II) binding on the surface of the ZETA_1 enzyme near the flexible C-terminal streptactin tag, which may be enhanced in the triple histidine knockout.

• A general discussion about why only one out of 96 ordered designs is highly active, or what sets this design apart from the rest, would be very informative. Is there another design with very similar metrics, which is not active at all in the dataset of 96? Can there be any conclusion drawn from it?

As previously mentioned, we added a section to the supplemental information, section 2, containing multiple subsections of this analysis from a computational metric perspective and a structural design model perspective. We observed several trends among the active designs from this campaign and used them to guide a new design campaign, summarized in Figure 4 of the main text. The results of the second design campaign significantly outperformed those from the previous design campaign: we observed a higher success rate and achieved designs with improved catalytic efficiencies and higher rate enhancements compared to the previous best design, ZETA_1 (A1). This strengthens our analysis and conclusions from the first design campaign.

Minor comments:

• In Fig. S12, what does this ddG correspond to? Should it be dG of binding?

ddG (ΔΔG) is a Rosetta metric, we believe our axis title is correct but we appreciate the detailed reviews. In Rosetta's context, ΔG refers to the difference in Gibbs free energy (G) between the unfolded and folded state so $\Delta G = G_{folded} - G_{unfolded}$. Thus, ΔΔG is the difference between these ΔG's for the holo and apo protein: $\Delta\Delta G = \Delta G_{holo} - \Delta G_{apo} = (G_{holo\_folded} - G_{holo\_unfolded}) - (G_{apo\_folded} - G_{apo\_unfolded})$.

• Fig. S13 would be more insightful if the same plots could be done with Chai-1 (prediction vs design with ligand) vs AF2, since the authors also use Chai-1 as a validation tool in their study.

We added (**C**) and (**D**) to Fig. S13 which compares AF2 RMSD (x-axis) to Chai-1 metrics from predictions with the transition state phosphoanalog (TSA) of our substrate (see below).

Additionally, we did an in-depth analysis for many more metrics for the first design campaign from Chai-1 in Fig. S20 using different prediction strategies (i.e., substrate, products, phosphoanalog, tetrahedral intermediate) which informed how we used Chai-1 in the second design campaign.

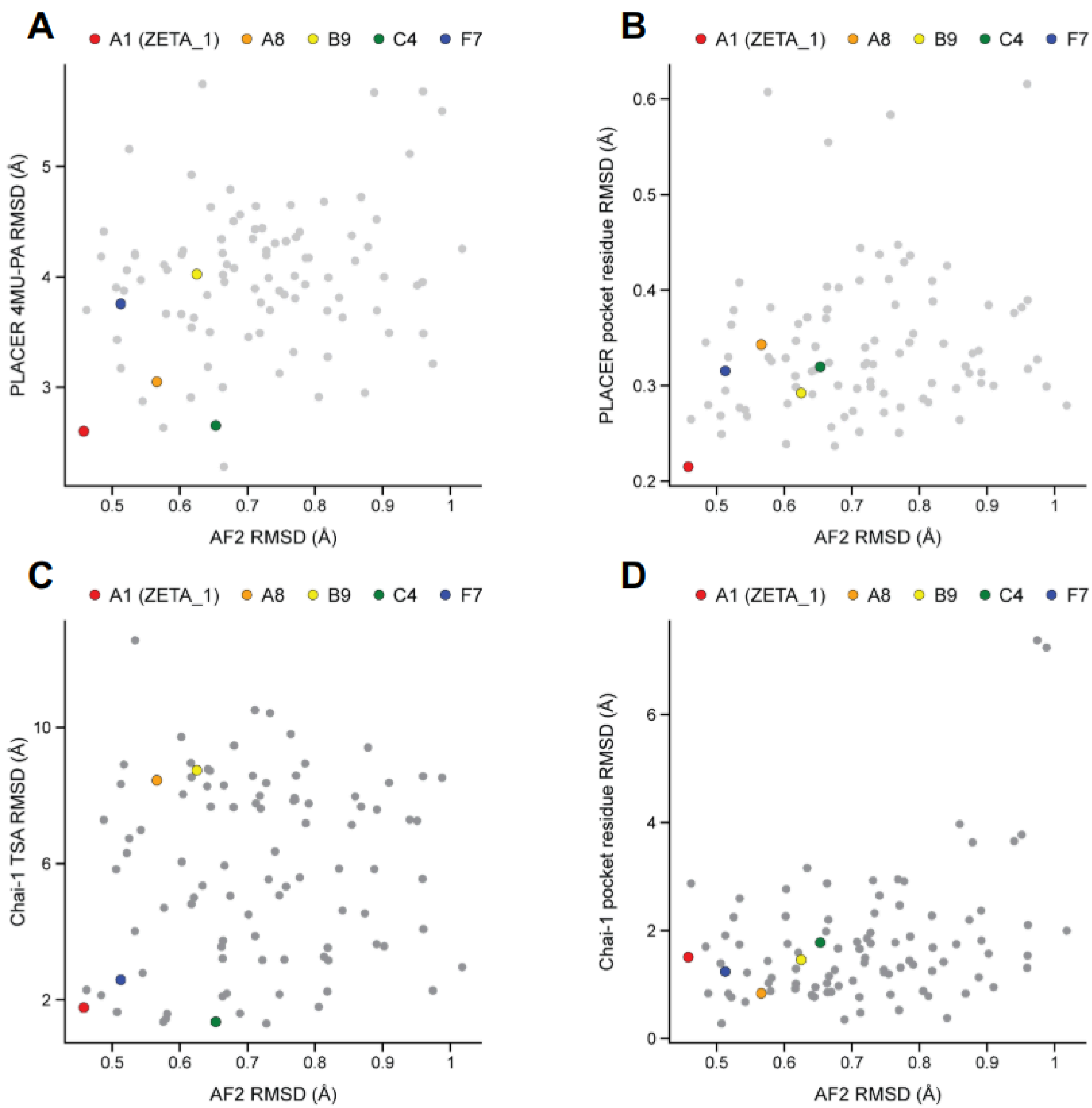

Fig. S13. Comparison of AlphaFold2 metrics with PLACER metrics and Chai-1 for ordered designs. (**A**) Average substrate RMSD between the design model and the predicted PLACER ensembles (PLACER 4MU-PA RMSD) vs the global C_α RMSD between the design model and the AlphaFold2 prediction (AF2 RMSD). (**B**) Average active site residue RMSD between the design model and the predicted PLACER ensembles (PLACER pocket residue RMSD) vs the AF2 RMSD. (**C**) Average transition state phosphoanalog (TSA) RMSD between the design model and the predicted Chai-1 ensembles with Zn(II) and the TSA vs the AF2 RMSD; the TSA is a good way to predict with something that mimics the transition state (see Fig. S20). (**D**) Average protein pocket residue RMSD between the design model and the predicted Chai-1 ensembles with Zn(II) and the TSA vs the AF2 RMSD. Note that the bottom left corner is ideal in all plots, indicating high agreement between the design and predictions. Interestingly, A1 (ZETA_1) is the closest to the bottom left in 3/4 of these plots, suggesting that high agreement between AlphaFold2/PLACER/Chai-1 may be useful for predicting activity.

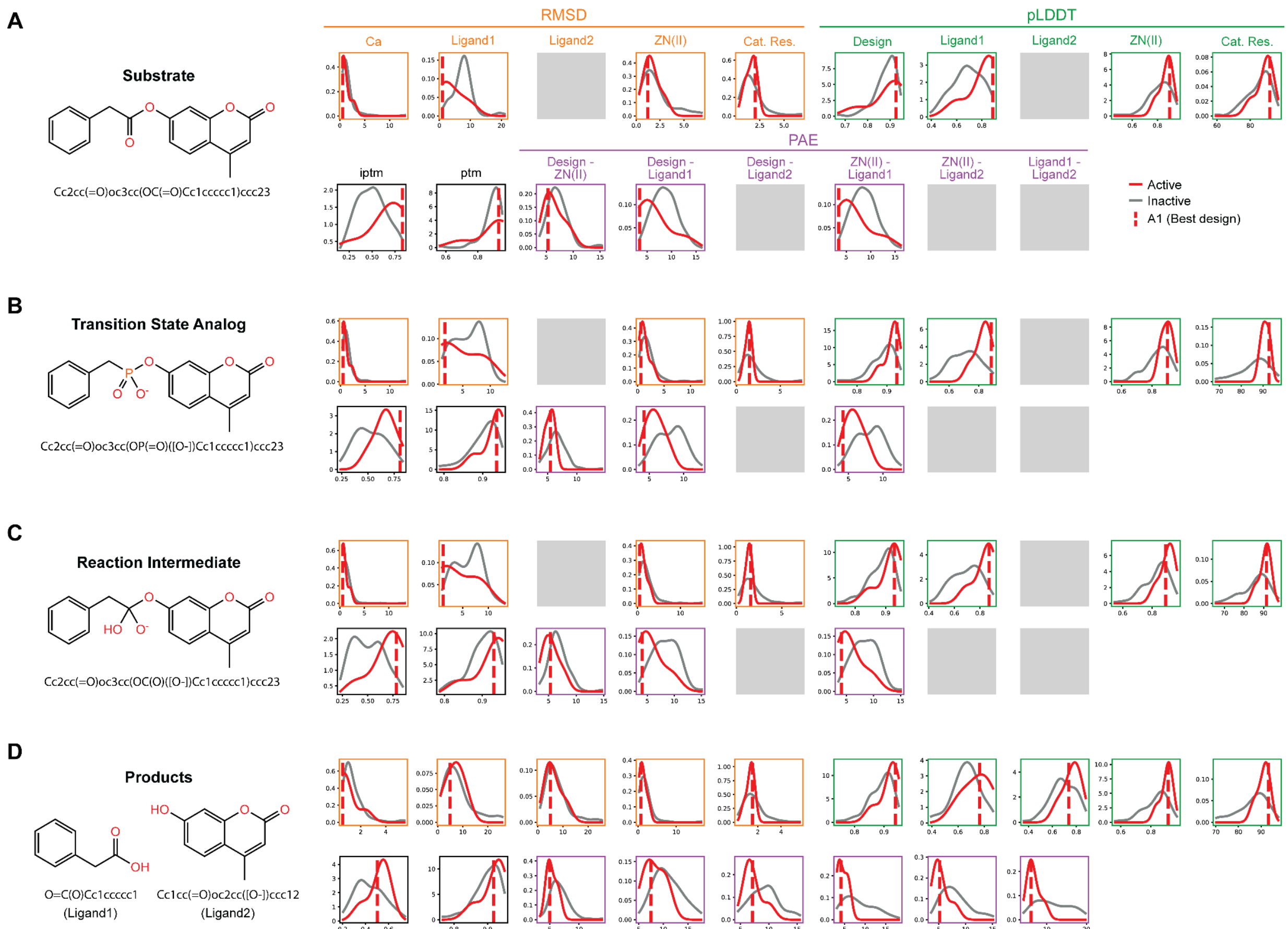

Fig. S20. Chai-1 prediction distributions with different ligands for the active and inactive designs from the first campaign. Averaged metrics across five Chai-1 prediction ensembles of the designed protein sequence, one Zn(II) ion, and either (**A**) the substrate, (**B**) the transition state phosphoanalog, (**C**) the tetrahedral reaction intermediate, or (**D**) the two products. The ligand strings are provided for each small molecule used in the prediction. The black line represents the metric distribution for the inactive designs from the first design campaign (91 designs), the red line represents the metric distribution for the active designs from the first design campaign (5 designs), and the dashed red vertical line represents the metric value for ZETA_1 (A1). The metrics are labeled in (A) and organized the same way in the rest figure. There are five main classes of metrics evaluated: RMSD between the design model and predicted model (orange border; lower is better), pLDDT of the predicted model (green border; higher is better), pTM of the prediction and interface pTM (ipTM) of the prediction (black borders; higher is better), and PAE of the prediction (purple border; lower is better). Note that (D) has 2 small molecules corresponding to the two products from the hydrolyzed substrate, these positions are grayed out in (A-C). We believe that predicting with the transition state analog is the most rational decision (see section 4.2). This informed the second design campaign filtering, which actively used Chai-1 for filtering rather than retrospective analysis.

• Table S3 is highly informative, but mixes some de novo designs with designs based on natural scaffolds in the section 'Computationally designed non-hydrolytic enzymes (from existing scaffolds, no laboratory evolution)'. This section should be properly split between de novo and natural scaffolds. Also the values reported should be double checked; there is some mix-up between s-1 and min-1.

We appreciate this comment and thank the reviewer for identifying some of these minor mistakes with Table S3. We identified one enzyme, RAD29, which we believe should move to the "Computationally Designed/Engineered Non-Hydrolytic Enzymes (from scratch)". If this is not what the reviewer intended, or if there are other cases they believe are miscategorized, we welcome further suggestions and discussion. Additionally, we identified two instances of retroaldolases (RA95 and cRA-50) with kcat values reported in min-1 in their original publications; we have corrected these to s-1 in the table. We believe these adjustments address the reviewer's concerns, but we remain open to any additional feedback.

• A table with statistics about pocket volumes, ligand burial, and substrate-bb contacts of the generated and designed scaffolds would be very informative, particularly since they are of varying length and only one in 96 is really active.

We performed this analysis for the design models from the first design campaign using CAVER 3.0 (https://caver.cz/) and Rosetta, included as Table S6 in the supplemental information (also shown below). Unfortunately, this pocket analysis did not reveal anything significantly distinct about ZETA_1 or the top 5 designs from this order when compared to the inactive designs.

Measurement	Design					
	**A1 (ZETA_1)**	**A8**	**B9**	**C4**	**F7**	**Inactive**
Substrate Relative SASA	2.4	54	6.2	12	3.4	11 ± 14
Contact Molecular Surface	255	229	250	273	250	248 ± 19
Pocket Volume (Å^3)	1316	1337	2500	1108	1008	1294 ± 616
Pocket Hydrophobicity	38	35	30	34	27	28 ± 12

Referee #1 comments on code availability:

My code review is limited to running the demo, as code is not yet available for neither RFflow nor ChemNet. The installation instructions and demo are good to follow and execute, if you have performed protein design tasks with similar tools before.

PLACER (formerly called ChemNet) is now publicly available on GitHub at the link:
https://github.com/baker-laboratory/PLACER.

Referee #2:

This paper introduces RFflow, a novel generative model for protein design, and applies it to the challenging problem of engineering histidine-bound zinc metallohydrolases. The work is notable for its conceptual innovations and experimental validation, which demonstrate improvements over previous methods in both methodology and outcomes.

The authors' workflow integrates quantum chemistry-based active site modeling with generative AI. Specifically, density functional theory (DFT) is used to define the active site geometry and substrate poses--an extension of the theozyme concept. The model simplifies the histidine residues in the active site, representing them solely by their imidazole rings, excluding the sidechain and backbone atoms. This abstraction is then used to generate protein scaffolds around the catalytic motif, and the scaffolds are populated with sequences optimized using ProteinMPNN. The workflow includes a design validation step, comparing the generated sequences with predicted structures from AlphaFold2 (AF2). This serves as a proxy for "designability," assessing whether the designed sequence is likely to fold into the intended structure.

The novel aspect of RFflow lies in its conceptual departure from previous models like RFdiffusion. Diffusion models typically introduce noise into protein structures, represented as a set of frames centered on Cα atoms, and gradually reverse this noise to generate novel scaffolds. RFflow, in contrast, uses flow models from computer science, which rely on vector fields to guide atomic positions without requiring explicit sequence information. This approach enables the model to focus on the placement of functional groups and atomic coordinates, bypassing the need for sequence-specific constraints during scaffold generation.

The authors experimentally characterized their designs, focusing on 96 zinc metallohydrolase candidates. Of these, 4-5 showed catalytic activity, with the best design achieving a catalytic efficiency (kcat/Km) of 2 x10^4 M^{-1}s^{-1}. While this efficiency is 2–4 orders of magnitude lower than that of natural enzymes, it represents a remarkable improvement over previously designed metallohydrolases. The standout design, A1, displayed the most preorganized active site, as assessed by ChemNet analysis. Mutagenesis studies confirmed the critical positioning of active site residues, suggesting a role for an aspartate residue in catalysis. Alternative sequences for the same fold showed lower ChemNet scores and reduced activity, highlighting the importance of active site preorganization. Additional structural and biophysical characterization of the A1 scaffold by circular dichroism (CD) spectroscopy confirmed its high helical content and showed reversible thermal unfolding. Notably, A1's fold is not found in the Protein Data Bank (PDB).

RFflow is a significant conceptual advancement over RFdiffusion, introducing flow models for sequence-agnostic atomic scaffolding. The catalytic efficiency of the best design is an order of magnitude better than previous de novo metallohydrolases. The authors discuss future directions for improving activity, such as additional rounds of directed evolution. However, the description of RFflow is somewhat unclear, particularly regarding how the vector field is trained and applied. The main reference for RFflow is a "preprint in preparation," which limits the reader's ability to fully understand and evaluate the method. It would be helpful to clarify how RFflow differs in implementation from Jaakkola's flow models.

We appreciate reviewer 2's summary of our manuscript. We apologize about the miscommunication regarding the preprint for RFdiffusion2 (previously called RFflow): a rough draft was late-added to the Google Drive that was shared with the editing team but it appears

this never made it to the reviewers. A detailed description of the methodology for the training and application of RFdiffusion2 has now been published at the link:
https://www.biorxiv.org/content/10.1101/2025.04.09.648075v2

Referee #3:

This is very interesting work. The designed metallohydrolase is a significant achievement. The designed protein has excellent activity, which is a notable achievement for a truly de novo metalloenzyme. The biochemistry and molecular biology characterizing the proteins is thorough and detailed. The authors provide good evidence to confirm the protein residues are functioning as designed, which is important verification of the design approach.

The work would be strengthened by the addition of structural biology to verify overall structure, ligand binding, etc: in particular, this would provide details of the active site structure (testing the ChemNet predictions), and verify that it is a novel fold. Perhaps the authors have been able to solve a crystal structure in the interim? However, the AlphaFold2 predicted structure is likely to be reasonably accurate overall. It would be useful to mention briefly the expected accuracy of structure prediction for zinc proteins.

We appreciate reviewer 3's enthusiasm about our manuscript and thank them for the kind words. We are working on a crystal structure for ZETA_1, but as previously mentioned, it has been quite elusive despite our extensive efforts. As shown in Fig. S15, the AF2 and Chai-1 predicted structures for ZETA_1 agree remarkably well with the design model and are predicted with high confidence. It is difficult to precisely speculate about the expected accuracy of zinc proteins, especially in a *de novo* enzyme context, but we believe that they should be predicted fairly accurately and confidently due to the abundance of zinc proteins in the PDB. A quick search in AF2 database reveals many diverse zinc metalloenzymes that are predicted with high confidence by AlphaFold2 (e.g., astacin: AF-Q20459-F1-v4, thermolysin: AF-V6SV72-F1-v4, carboxypeptidase: AF-Q04609-F1-v4, carbonic anhydrase: AF-P23280-F1-v4, matrix metalloproteinase: AF-P50281-F1-v4).

RosettaFlow looks a very useful tool, which should prove widely applicable. I was somewhat confused that a different name was used in the preprint of this work. It is not clear that the approach described, however, will be broadly applicable beyond fairly simple zinc hydrolases. Ref 33 (Ahern et al.) is crucial to understanding the basis of this method. The successful designed enzyme is a small protein with a simple and common active site motif (three histidines). During the design process, eventually the zinc binding site is fixed, so the design procedure seems to be really a process of designing/redesigning the first and second sphere residues. This could limit the range of accessible activities as targets for design. Could this approach be used to design a more complex activity than a monozinc hydrolase? Would that require significantly more computational power? Also, (see further below), would more detailed, e.g. multistate templates be required (as in the multistate template used by Baker et al. in their recent designs of serine hydrolases)? This does not undermine the impressive work here, but is important for understanding the range of applicability.

We apologise for the naming confusion. Indeed, the initial preprint used the updated name RFam (RosettaFlow atomic motif), whereas the version we submitted to Nature used the name RFflow. However, we discovered that RFam also refers to an RNA database, so we once again updated the name to RFdiffusion2. We have updated the manuscript to make the naming consistent with RFdiffusion2.

These are great questions about the design process and the generalization of RFdiffusion2. The zinc binding site is effectively fixed from the beginning: most of the substrate conformational sampling and all of the histidine-zinc coordination (i.e., delta or epsilon coordination) is done

before RFdiffusion2 to generate a bunch of permutations as different model inputs. Beyond that, any step that involves Rosetta can “see” the other pre-generated substrate conformations and decide to swap that in if it thinks a certain substrate conformation will fit better in the designed pocket or it can decide to flip the histidine imidazole ring(s) 180 degrees if it will help its preorganization (eg make a hydrogen bond on one side), but the histidine-zinc coordination is fixed from the beginning. However, the most important takeaway is that the core groups involved in the reaction (hydroxide, ester group atoms, zinc, imidazole rings besides a 180 flip) have their geometries completely fixed during these “sampling” processes and throughout the design to respect the DFT model, which we consider to be a theoretical “ground truth.” In other words, we suppose that if these groups came together this way, the reaction should happen. The sampling of the substrate conformers (ie non-ester atoms) and imidazole 180 degree flips could be argued as arbitrary decisions that technically betray the DFT models, but we reasoned that this sampling flexibility may help account for different substrate/imidazole conformations that would be preferred in the context of a complete enzyme active site.

Regarding this reviewer’s question about if this approach could be used for more complex reactions, the short answer is yes. In the RFdiffusion2 methods manuscript, RFdiffusion2 was additionally used to generate experimentally-verified retroaldolases and cysteine hydrolases, although these designs went through slightly different design pipelines after the RFdiffusion2 step than what we presented in this manuscript. Nonetheless, RFdiffusion2 and this pipeline should be generalizable for “targeting” the rate-limiting step of any reaction. This should not require significantly more or less computational power for each scaffold generation, but the success rates from filtering (which are user-defined) may differ between different reactions/substrates/theozymes and depending on the number of catalytic functional groups that are fixed.

There is not an explicit strategy to provide multistate templates to RFdiffusion2 or RFdiffusion yet. For the design of serine hydrolases, those authors used a similar strategy to us for the scaffold generation step: they diffused scaffolds with RFdiffusion around the catalytic triad poised for the rate-limiting nucleophilic attack on their substrate. They maintained this geometry throughout design, never explicitly designing for the other steps. Instead, they used PLACER to predict the behavior of these designs at every step in the reaction, carefully filtering for designs that were predicted to have the correct geometry at each step in the reaction. Thus, this was a demonstration of clever filtering for designs that were predicted to be competent at each step in the multistate reaction rather than explicitly designing for each step of the reaction from scratch.

However, we believe that future studies could (and should) try to integrate RFdiffusion2 into a design pipeline that explicitly considers and designs for each step in a multistate reaction. One could model the geometry of the rate-limiting step for the initial scaffold generation. Then, one could substitute models of the geometries for the other reaction steps into these scaffolds and use the RFdiffusion2 version of “partial RFdiffusion” to noise and denoise these scaffolds into folds that are better suited to host those other reaction step geometries. At this point, users would have an ensemble of conformations for each scaffold, one conformation for each reaction geometry. They could then use “tied-MPNN” to search for sequences that are predicted to fold into proteins that would adopt all of these conformers and follow the rest of the design pipeline for validating the folds of these sequences and using PLACER to predict ensembles at each step in the reaction as done in the serine hydrolase paper. This strategy is analogous to what was performed in a previous paper making two-state hinge proteins (Praetorius *et al. Science* **2023**).

Notably, only a single structure (for the TS of four variants of the reaction) from a density

functional theory (DFT) calculation is used as the template. These TS structures are optimized with a reasonable level of DFT, and conformers are generated with lower level, approximate DFT. The authors make the coordinates of these "theozymes" available, which is good and appropriate. More details should be given about this structure in the main text, and the reasons for the choice should be stated (for example, it should be stated that it is a transition state structure, as described in the SI). The template here contrasts with the serine hydrolase work, which used several structures e.g. of different states in the reaction. Again this indicates that the main success of the design here is of second-sphere and surrounding residues, for a defined zinc site.

We added explicit wording in the main text to indicate that the DFT models were of the transition state.

As mentioned previously, the main difference between the template in this study and the one in the serine hydrolase paper is that they needed to specify the atoms of the full residues for RFdiffusion and had to manually generate all of those combinations of conformers, whereas we only had to specify the "tip atom" functional groups for RFdiffusion2 (see Fig. 1A & S4). They did not use a multistate template, but rather filtered for designs with PLACER that were predicted to perform competently for different states of the reaction (besides the one explicitly designed for: the nucleophilic serine attack). In that sense, both the serine hydrolase paper and us designed for a single state.

As with the serine hydrolase designs, it would be useful to comment on the sensitivity of the design to the template. Has this been tested? What factors make a good template? This is important to guide for future work. For example, TS structures are used as a template. This is a reasonable choice, but tetrahedral intermediate structures could be offer advantages (e.g. the stabilization by the protein would be expected to be largest for the TI).

We have not explicitly benchmarked the sensitivity of RFdiffusion2 to the template, as we believed that would be out of the scope for this paper. However, RFdiffusion2 should be quite generalizable to many templates as demonstrated in the separate manuscript where it was used to successfully scaffold 41 diverse enzyme active sites across many EC numbers and experimentally-validated to make cysteine hydrolases and retroaldolases in addition to our metallohydrolases.

We believed that designing for the transition state structure was the most ideal template based on enzyme catalysis and transition state theory (Lienhard *Science* **1973**). Enzymes catalyze reactions by binding the transition state tighter than the substrate or products. Thus, we believe designing for the transition state is best when possible.

It would be useful to compare to the serine hydrolyase design process (which applied a different, diffusion model; there is already good discussion of RFflow versus the RFdiffusion) and discuss recommendations for enzyme design, including choice of template(s)/theozyme(s). This would enable the wider community to make use of these powerful design methods effectively.

A detailed comparison of the two methods is provided in the RFdiffusion2 *bioRxiv* preprint (Ahern et al. *bioRxiv* **2025**).

Referee #1:

In their revised version of 'Computational Design of Metallohydrolases' Kim et al. addressed pretty much all issues/questions the other reviewers and I were concerned about.

Overall, I think this is a great study, showing the capabilities of scaffolding functional groups alone in a sequences position agnostic way using RFdiffusion2. I especially would like to highlight the additional insight the authors gained from their analysis of the first round of designs and their subsequent generation of a second round with an - arguably - more complex TS. Also the added Supplementary Figs. S30-S33 are highly informative!

We appreciate the enthusiasm from reviewer 1 and thank them for their invaluable feedback during the review process. Their constructive critiques have significantly strengthened the manuscript.

*While I can relate to the frustrations of not being able to obtain diffraction quality crystals, even with a high screening effort, the lack of *any* global structural analysis of any of their designs is still disappointing, given that the statement 'our designs are too small for SAXS analysis' is just not true and 1H,15N HSQC samples are very easy to produce and measure.... Yet all controls show that their designs work for the right reasons and thus I think validations are ok.*

Fortunately, we have solved the structure of the most active design, ZETA_2, in the *apo*-state at 3.5 Å using X-ray crystallography (PDB: 9PYJ, Fig. 5). We had to solve it in the *apo*-state because ZETA_2 was extremely soluble (observed >50-65 mg/mL), but zinc sulfate precipitates out in our buffer conditions above 300-400 uM, so we could not add zinc sulfate to match the protein stoichiometry during the initial crystal screening (see SI section 4.3). Nonetheless, the experimental structure is in good agreement with the design model, with nearly superimposable backbones (Cα RMSD = 1.1 Å) and the catalytic residues preorganized in the designed geometry (Fig. 5 A,B). The binding pocket is highly complementary to the superimposed transition state from the design model (Fig. 5C).

We were then able to solve a 2.1 Å structure after soaking crystals in Zn(II) (PDB: 9PYL, Fig. S35); while the backbone was again nearly superimposable with the design model (Cα RMSD = 0.8 Å) and a Zn(II) ion was present with 100% occupancy at the designed location (RMSD = 1.7 Å), one of the Zn(II)-coordinating histidines (H110) was flipped out to interact with a Zn(II) ion on the surface of the protein, likely because of the high Zn(II) concentration in the crystal-soaking buffer (250 mM) (Fig. S35 A,B). We hypothesize that this could also be an intermediate conformational state in the Zn(II)-binding that was captured and stabilized in the crystal. Nonetheless, it suggests that future design efforts should try to further improve the buttressing of the metal-binding residues, which may help bridge the gap in activity between native and designed metallohydrolases. We added additional text in the main and supplementary information describing these results and methods. Both crystal structures have been submitted to the PDB with access codes 9PYJ (*apo*-ZETA_2) and 9PYL (Zn(II)-bound ZETA_2).

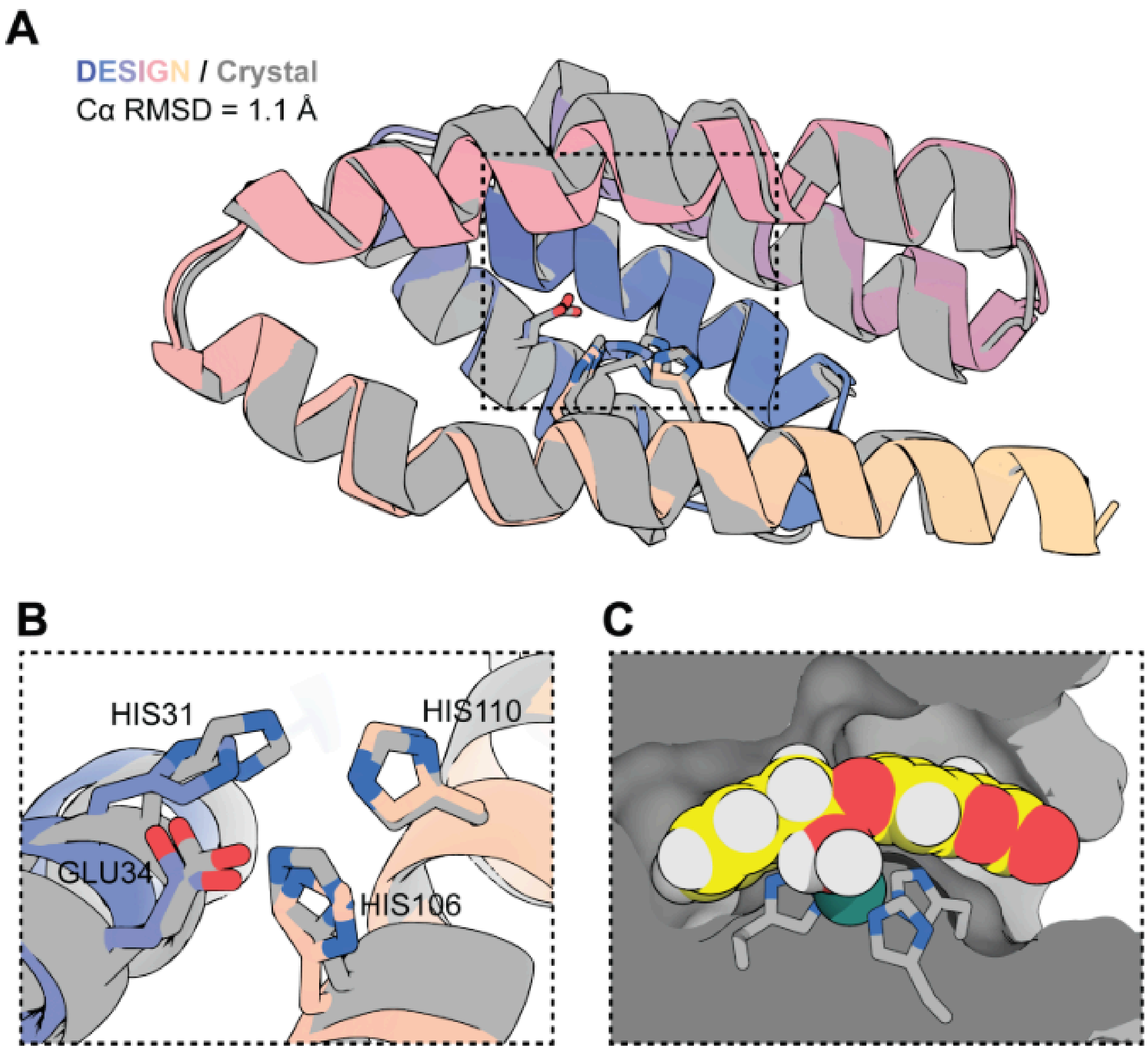

Fig. 5. Crystal structure of ZETA_2 is close to the design model. (**A**) C_α superposition of the design model and the X-ray crystal structure of ZETA_2 (PDB: 9PYJ) in the *apo*-state, resolved at 3.5 Å resolution. Catalytic residues shown as sticks. The structures are in close agreement (C_α RMSD = 1.1 Å), and the catalytic residues in the experimental structure are preorganized close to their designed catalytic geometry. (**B**,**C**) Active site zoom-in highlighting the agreement between the experimental and designed catalytic geometry (B) and the shape-complimentarity of the designed binding pocket and the transition state model (superimposed in from the design model). Crystal structure shown in grey and design model shown in color in all panels.

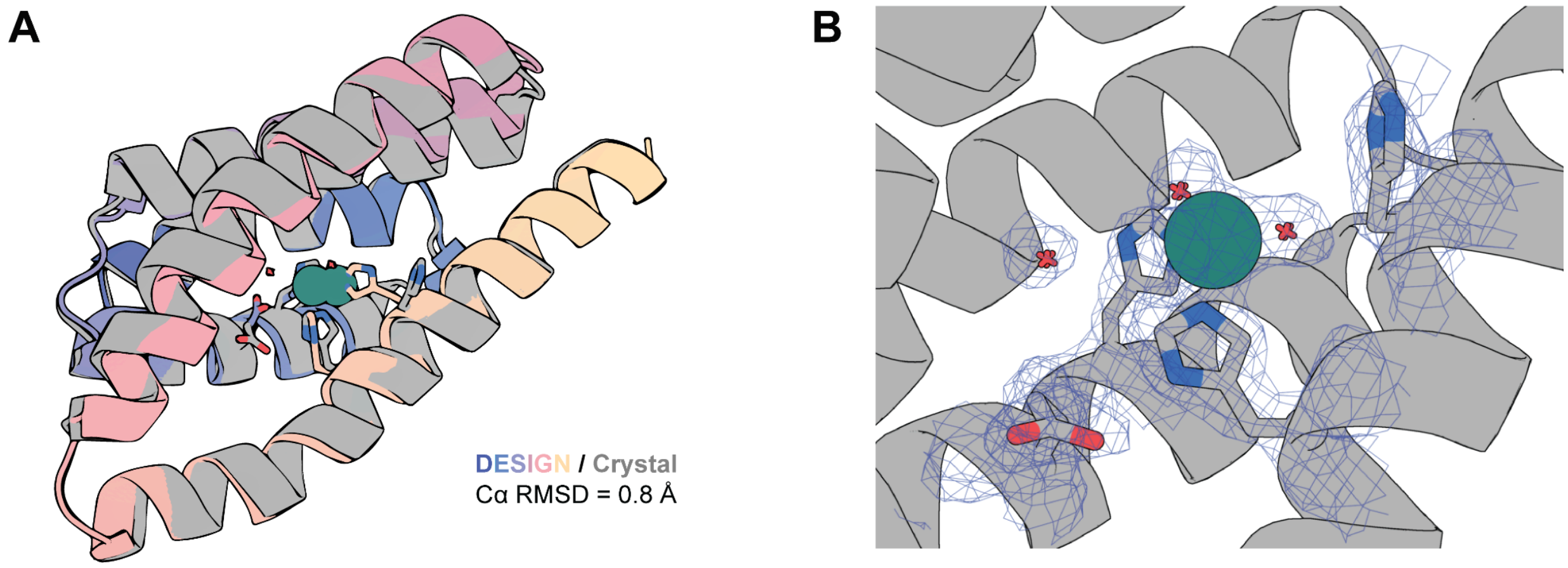

Fig. S34. Crystal structure of Zn(II)-bound ZETA_2 after soaking. (**A**) C_α superposition of the design model and the X-ray crystal structure of ZETA_2 (PDB: 9PYL) in the Zn(II)-bound state from crystal soaking, resolved at 2.1 Å resolution. Catalytic residues shown as sticks and Zn(II) ions shown in green spheres. The structures are in close agreement (C_α RMSD = 0.8 Å) and the Zn(II) ion is present with 100% occupancy in the correct binding location (RMSD = 1.7 Å). (**B**) Active site zoom-in with the 2mFobs-DFcalc electron-density map (blue mesh) contoured at 1.0 σ for the catalytic residues, Zn(II) ion, and water molecules. One of the catalytic histidines (H110) is flipped out in the experimental structure to interact with a Zn(II) ion in solution, perhaps because of the high Zn(II) concentration used in the crystal-soaking buffer. We hypothesize that this may be an intermediate conformational state in the Zn(II)-binding that was captured and stabilized in the crystal. Crystal structure shown in grey and design model shown in color in all panels.

Moreover, I still think that it would have been great if Kim et al. not only showed that inpainting of loops with RFJoint2 led to higher convergence in AF2 predictions, but also analyzed what fraction of their active designs came from back bones directly out of RFdiffusion2 vs. RFdiffusion2+RFjoint2, to get a better idea if this was a necessary design step to achieve high catalytic efficiencies or not.

Please see our new supplemental figure, Fig. S34, which displays the AF2 distributions for the inpainted vs uninpainted structures.

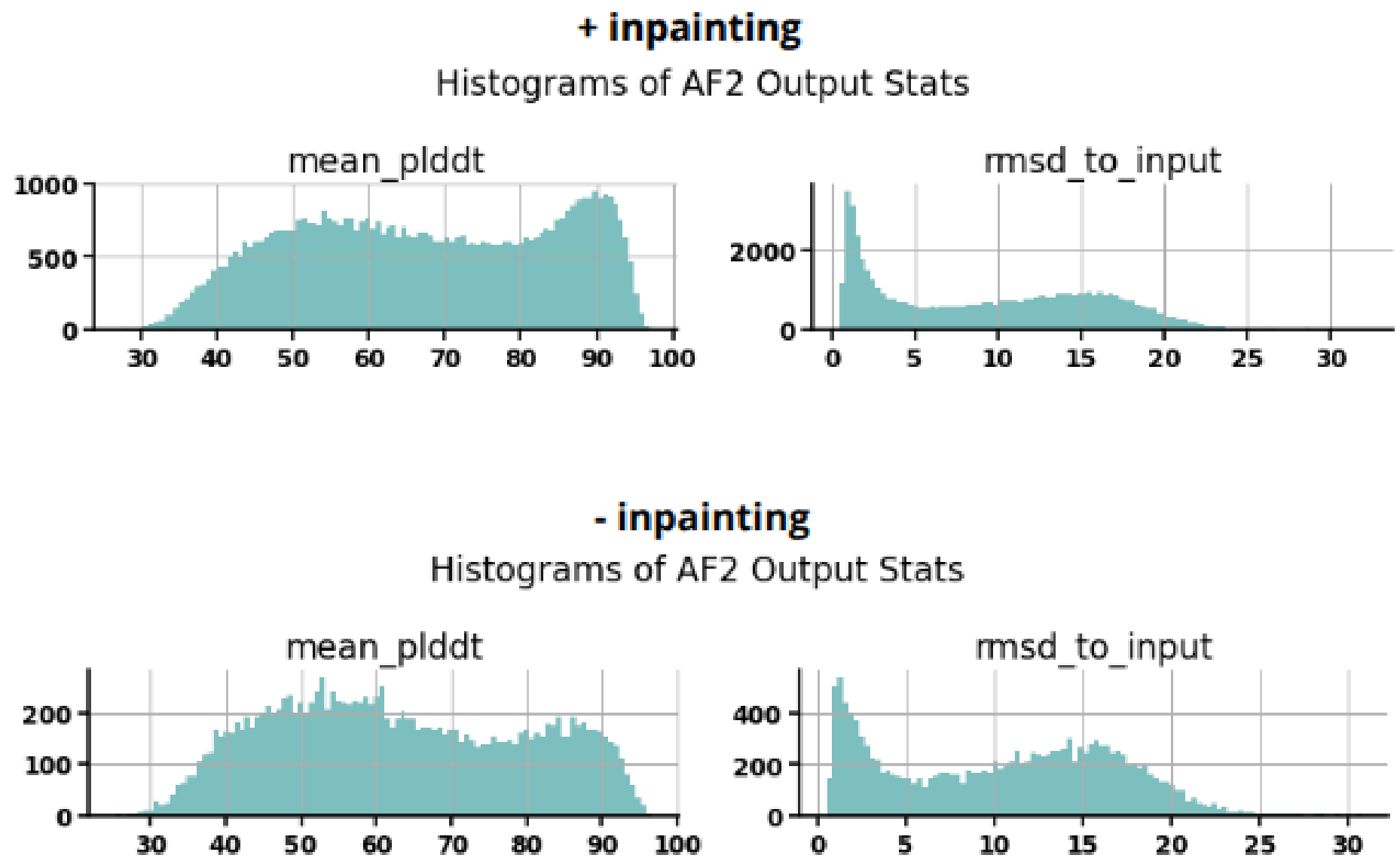

Fig S34: Histograms of AF2 metrics from the first design campaign comparing designs with and without RFjoint2 inpainting applied.

Aside from showing improved convergence for the first AF2 prediction for structures that underwent RFjoint2 loop inpainting, it is difficult and potentially misleading to attribute anything else to RFjoint2 because of statistical bias. Each RFdiffusion2 structure was inpainted at least four times (i.e., one RFd2 structure yielded four inpainted variants), and in cases with more loops, this number was allowed to be higher. As a result, there were more than four inpainted variants per RFdiffusion2 structure, and this overrepresentation became even more pronounced after applying the first round of AF2 filtering. Consequently, inpainted structures were disproportionately represented downstream in the design process, making it challenging to determine whether a structure passed our final *in-silico* filters, was ordered, and showed high activity due to genuine improvements from inpainting or merely by chance.

Moreover, inpainting did not always alter the sequence length; in some cases, it only introduced minor changes to loop conformations, resulting in structures that closely resembled the original RFd2 models. Therefore, we do not consider the use of RFjoint2 essential for achieving high catalytic efficiency. Notably, the RFdiffusion2 preprint (Ahern et al., *bioRxiv* **2025**) did not utilize inpainting for any of its reported results, except for the zinc hydrolases, which were designed using this pipeline. That said, we would encourage protein designers to consider employing a scaffold “diversification” method like RFjoint2 loop inpainting as we did; in the second design campaign, 89/96 ordered structures were inpainted and all of the hits came from inpainted structures, so there is a possibility that it does help.

Things I'd recommend to take another look at, before publication:

1. The MM plot of Zeta_4 in Fig. 4h shows that this titration clearly was not finished. The reported Km is above the highest measured data point. This needs to be repeated with higher substrate concentrations, or should be removed since it does not add too much to the story. Unless the authors start discussing intrinsic features of their folds and why some (Zeta_2 and Zeta_3 in particular) seem to result in higher activity designs after all, even when the sequence identity of the same back bone is fairly low (<80%).

This was a valid critique. We measured an additional point at 144 uM of substrate concentration for ZETA_4. The new Michaelis-Menten plot shows clear saturation with slightly altered values of kcat and KM. Figures 4C-E, Fig S25, and Table S1 have been updated to reflect the new plot and measurements.

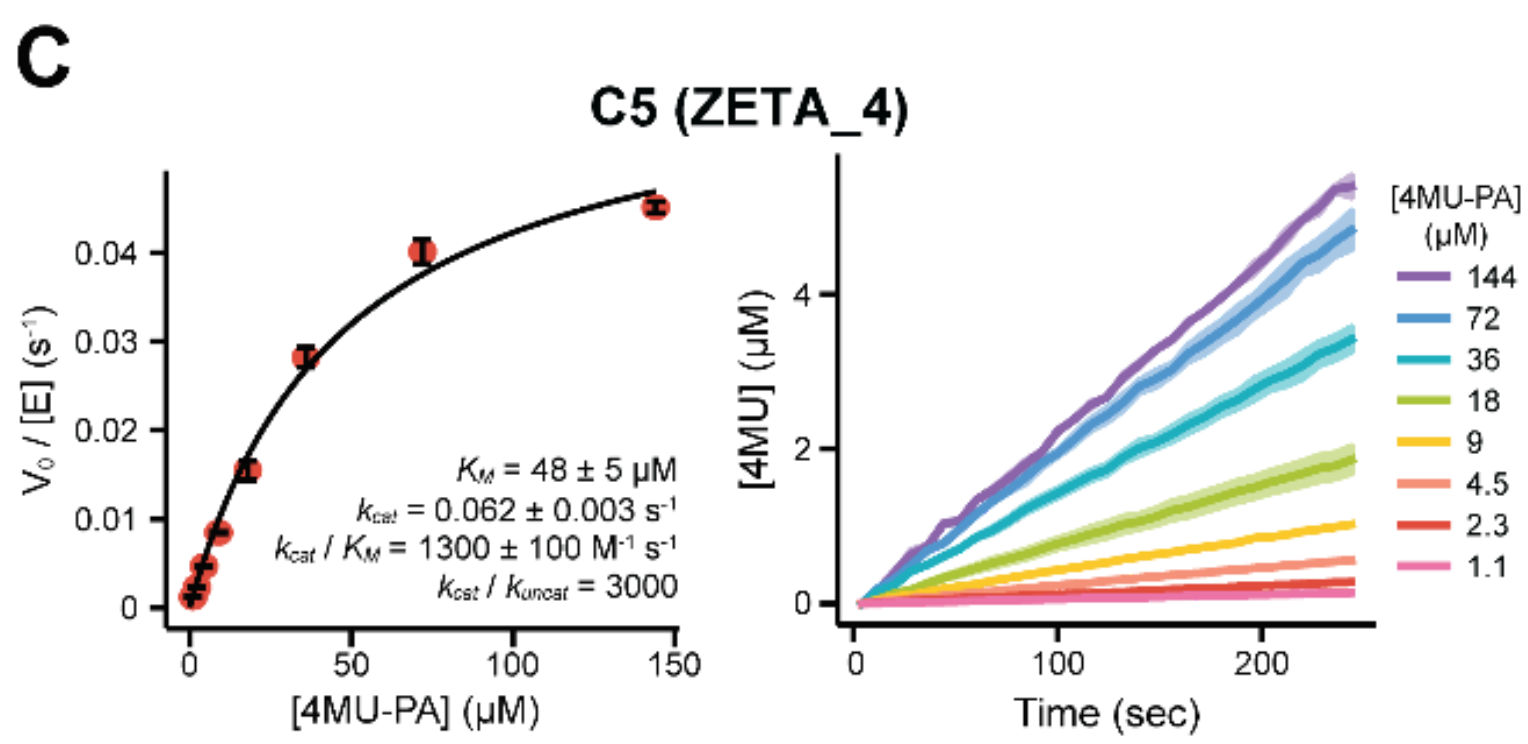

Figure 4C: Updated Michaelis-Menten plot for ZETA_4.

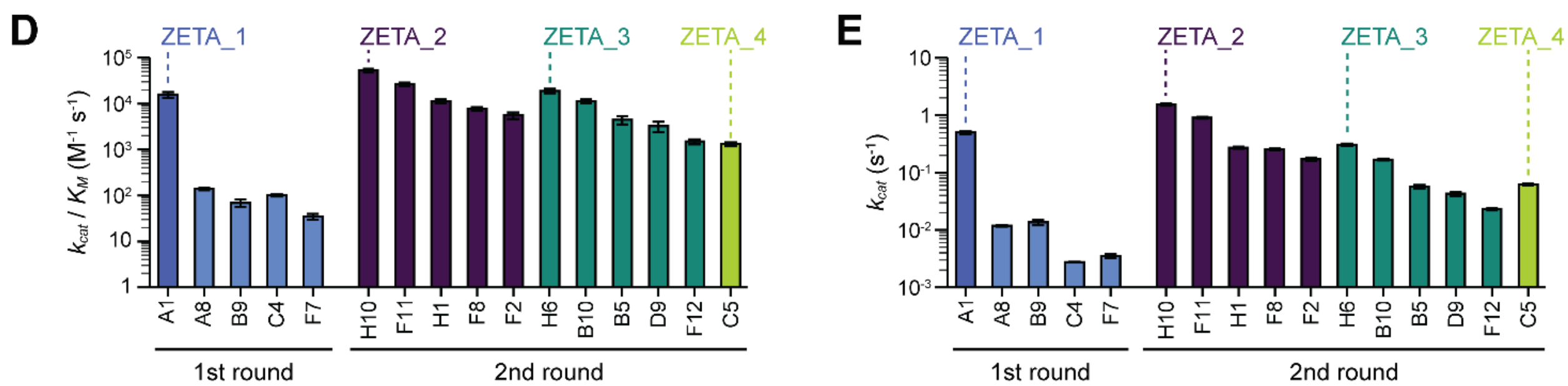

Figure 4D-E: Updated bar plots for the catalytic efficiency (kcat/KM) and kcat with ZETA_4.

2. Some of the reported CD-spectra show very high negative signals around the 209-nm peak - lower than what one would expect from purely alpha-helical spectra. This is fine, if there are lots of unstructured regions present in the designs as well. A conclusion which could be drawn if the spectra would have been recorded to 190 nm.

We agree that A8 and C4, two minor hits from the first design campaign, appear to show distinctly sharper negative peaks at 209 nm than their 222 nm peaks. We attempted to measure the spectra of these designs to 190 nm, but below ~202 nm the instrument voltage becomes too high, resulting in noisy data; we had a lot of difficulty finding the optimal buffer conditions to measure clean spectra for all the designs to 204 nm after the first round of review, and we did not want to deviate drastically from our typical buffer conditions, beyond just swapping HEPES for Tris, to keep everything consistent with the buffers used in other experiments in this work.

Looking at the design models for A8 and C4, it is definitely reasonable to imagine that they may contain some partly disordered regions, as the reviewer pointed out (Peer Review Figure 1). In A8 (left), the "hairpin-like" beta-sheet could exhibit flexibility, and the N-terminal helix might represent a flexible or partially disordered region that moves freely in solution. Similarly, C4 (right) features an N-terminal helix connected by a long loop, which could also contribute to partial disorder or high flexibility. Regardless, we believe that the spectra still suggest the structures are mostly helical and are likely folding as designed.

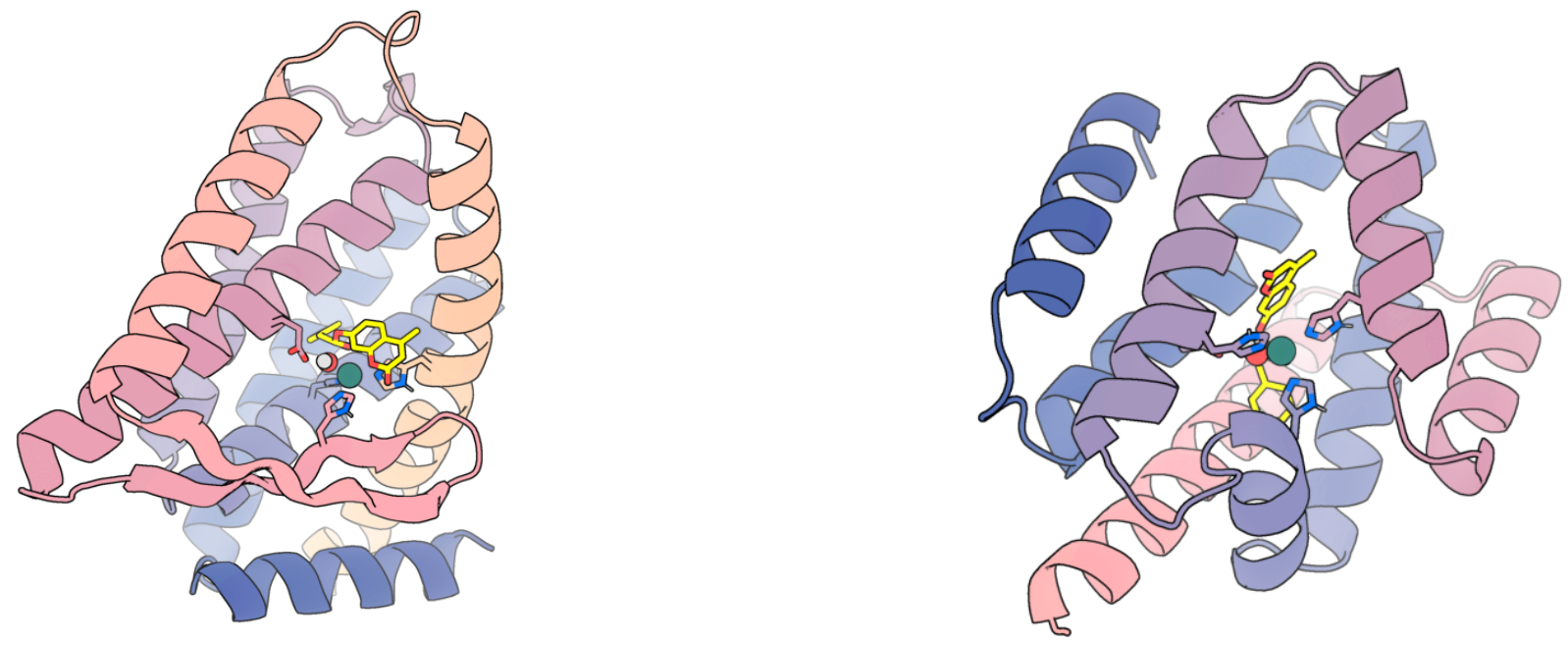

Peer Review Figure 1: Design models of A8 (left) and C4 (right). The A8 N-terminus is at the bottom right of the structure (blue helix) and the C4 N-terminus is at the top left of the structure (blue helix).

Comments on code:

Instructions to install and run the individual programs are fine.

The only issue I had was while running PLACER. Initially, I ran into chemical connection issues of the used substrate(s), but could fix it by supplying extra ligand information in additional formats. And it seems this is a known phenomenon and addressed in the FAQs on the github repo.

We are glad that reviewer 1 was able to run the software we provided. We will refine the examples to avoid these issues upon publication of the metallohydrolase design pipeline demo on GitHub.

Referee #2:

No comments were provided by Referee #2 to respond to.

Referee #3:

The authors provide a detailed and convincing response. They have performed an additional, second design campaign which significantly illuminates the process and findings, and strengthens the conclusions. The insightful and detailed discussion in sections 2.1 and 2.2 of the supplementary information describes the implications and approach in detail and will be very useful across the field and in future applications. They have answered all questions and points that I raised in my initial review. The renaming of the computational tools is appropriate and clarifies matters, as does the additional details on (what is now called) RFdiffusion2.

This is important, groundbreaking work and is suitable for publication in Nature essentially as is.

We greatly appreciate the generous words from Reviewer 3 and their useful comments. We agree that the manuscript has been significantly strengthened by the review process.

There is a minor but important error in the SI: the term ChemNet is still used in places (e.g. p. 25 and elsewhere). Please ensure that the terminology/naming is updated for the computational tools throughout.

Thank you for catching this error that we missed, we have corrected the terminology to be consistent throughout the manuscript.

A minor but interesting question is whether there is any indication of spodium bonding or similar effects, as found to be important in some natural zinc enzymes (e.g. insulin-regulated aminopeptidase, IRAP) if indeed predicted structures can capture such effects).

This is a very interesting question that we had not considered until now. Upon inspecting the design models for ZETA_1-4, it appears that there could possibly be a spodium bond between the Zn(II) and a serine in ZETA_1 and a spodium bond between the Zn(II) and a tyrosine in ZETA_4 (Peer Review Figure 2). There were not any obvious spodium bonds in the ZETA_2 and ZETA_3 design models. Nonetheless, these effects are too often overlooked and we appreciate the reviewer bringing this to our attention, as this provides an interesting future direction to consider for metallohydrolase design; perhaps one could test the importance of spodium bonding by repeating this design pipeline from our theozymes, either explicitly adding a spodium bonding residue or omitting it, and comparing the design success rates and most active enzymes from each pipeline.

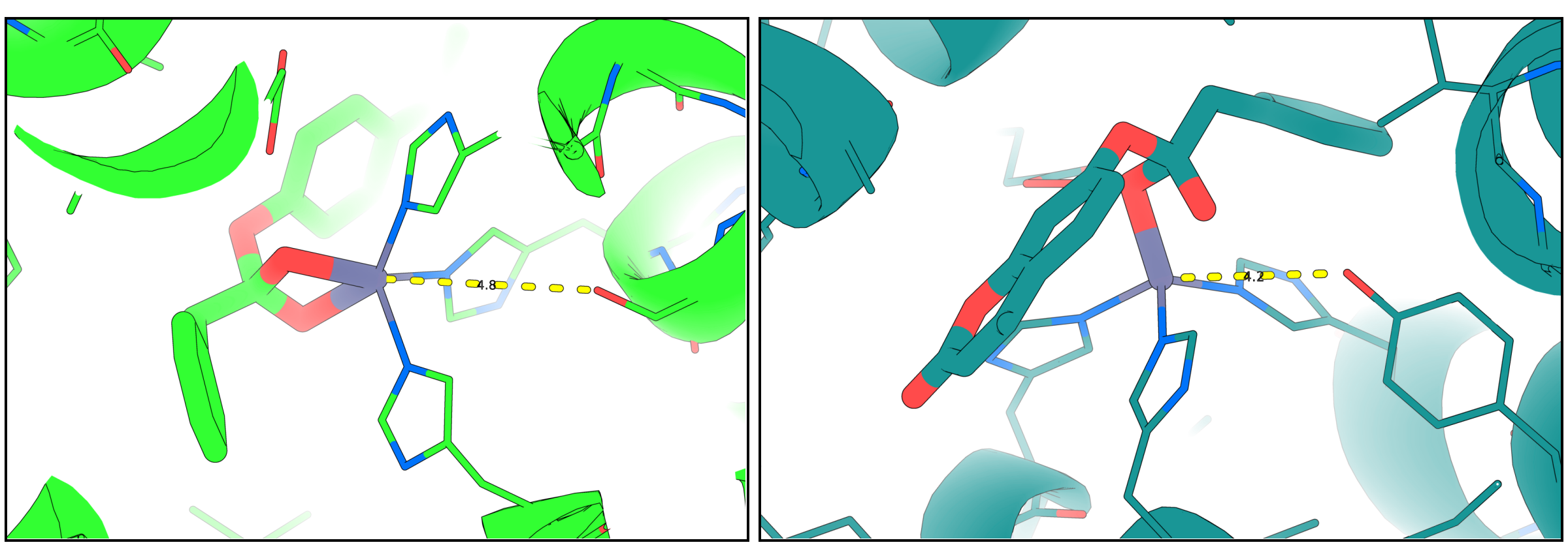

Peer Review Figure 2: Active sites of the design models for ZETA_1 (left) and ZETA_4 (right) highlighting potential spodium bonding interactions (dashed yellow line) with the Zn(II) ion. In

the case of ZETA_1, a serine is hypothesized to make a 4.8 Å spodium bond with the Zn(II) ion. In the case of ZETA_4, a tyrosine is hypothesized to make a 4.2 Å spodium bond with the Zn(II) ion.

Very minor is that the symbols for kcat, KM in Fig. 4 are very small.

We have enlarged the symbols for kcat and KM in Figure 4.

Dear Angela,

Thank you for the constructive feedback and consideration of our manuscript entitled "Computational Design of Metallohydrolases". We have addressed the minor comments provided by the new reviewer in this third round of review, but minimal changes were required. Any changes are shown as pink text, in addition to the blue and red text marking changes from rounds 1 and 2 of review, respectively. Additionally, we have updated the manuscript to adopt the format requirements set by the *Nature* guidelines such as picking up to 10 supplemental figures/tables to provide as extended data and shortening the abstract. Finally, please note that this manuscript cross-references the RFdiffusion2 manuscript accepted in principle at *Nature Methods* (NMETH-A60771) entitled "Atom level enzyme active site scaffolding using RFdiffusion2" by Ahern et al. Currently, Reference 30 cross references this work in the *BioRxiv*, and needs to be updated when the citation is available.

This manuscript has been significantly strengthened by the peer review process. We hope that you believe, as we do, that the manuscript is now suitable for publication in *Nature*.

Sincerely,

David Baker, Don Hilvert, Seth Woodbury, Donghyo Kim

Editorial Checklist:

1. The final manuscript must be in .docx format.
Done

2. The Abstract should be reduced to 230 words or less; currently there are 259 words.
Done

3. The methods references in the SI must not overlap references in the main list and must be continuously numbered.
In progress

4. You will need to remove the main figures from the article file and resupply them individually in an acceptable format such as EPS, AI, PS, PDF, PPT, PSD or XLS (for graphs) with editable vector files.
Done

5. You need to provide a Data Availability statement at the end of the Methods.
Done, please see the Data and Materials Availability statement

6. You need to provide Author contributions and Competing interests statements. If there are any patents, the inventor(s) last name, first initial; year; patent title; and patent number needs to be provided.
Done, please see the Competing Interests section.

7. The Code Availability Statement needs to be updated to indicate where it can be freely obtained. Is the related paper published?
Done

8. Currently there is no Extended Data. The structural data table (SI Table 7) must be ED, and then we ask that 9 of the most important figures in the SI be included as Extended Data. The titles/legends of the Extended Data need to be at the end of the main text file.

Extended Data Fig. 1-8 and Tables 1-2 are provided.

9. An SI Guide is required.
Done

10. The SI file is missing titles for the supplementary videos.
Done, please refer to the SI guide.

11. A third party rights table (see below) must be provided.
Done

12. Please ensure that the text size in all figures is at least 5 pt Arial.
Done

13. Please see below for guidance on chemical structures (present in Fig. 1).
Done

Referee #1:

In their revised version of 'Computational Design of Metallohydrolases' Kim et al. addressed all my previous comments and exceeded my expectations by adding an apo and a Zn-bound structure of their most active design. Both structures are in good agreement with their design model. I want to thank the authors for their additional efforts and congratulate them to this impressive study! The additional analysis and insights through it are of high importance to the field.

We appreciate the enthusiasm and support for the final manuscript and thank this reviewer for all of their constructive feedback throughout the peer review process.

Referee #1 (Remarks on code availability):

for a previous version. There has been no changes to the computational approaches reported here.

Referee #2:

No comments were provided by Referee #2 to respond to.

Referee #3:

No comments were provided by Referee #3 to respond to.

Referee #4:

This reviewer has been asked to comment on the structural data added to the revisions of "Computational Design of Metallohydrolases" by Kim et al.

The premise of the manuscript is to use a new generative AI-based algorithm to design functional zinc hydrolases. Indeed, the algorithm successfully design both a first- and second-generation zinc-dependent hydrolase. The structure of one, ZETA_2, was determined in the apo- and Zn-bound forms by X-ray crystallography.

The apo structure agreed with the design with an RMSD of 1.1 Å, revealing a pre-formed zinc binding site. The crystal form in the P2 space group could not be co-crystallized in the presence of high concentrations of zinc because that resulted in precipitation. The designed tetrahedral binding site appears zinc-specific, but no metal competition experiments were shown to analyze the degree of this specificity.

The zinc-bound structure was determined by soaking the crystals in high zinc acetate (250 mM), which revealed a zinc bound at the expected site. A second zinc forms a crystal contact, perhaps explaining the higher resolution of this structure. Binding the second zinc required flipping of one of the designed histidine ligands and changed the space group to P2(1)2(1)2(1).

The alignment of the fold to the designed fold supports the authors' claims about the potential for their AI method as a useful tool for designing novel metalloenzymes with native-like activities in a streamlined fashion. That the most active design is a helical bundle may not be all that surprising given that more labor-intensive de novo designs from all the way back to the early days of protein design were also helical bundles. Nevertheless, those designs required quite a lot of a priori knowledge not needed for this new method, so it represents a significant step forward.

We thank this reviewer for taking the time to review the manuscript and the crystal structures associated with it.

The discrepancy between the apo and zinc-bound structures and the adventitiously bound zinc at a crystal contact brings up a few questions. Some are easily addressed by including a little more detail or minor edits, others might be better suited for a detailed follow-up study. My opinions about the importance of the comments are added in parenthesis.

1) Are the biophysical analyses (i.e. SEC studies (Fig. S8) and CD studies (Fig. S29)) performed on the apo or metal-bound structure? (Buffer compositions could simply be included in the figure legend.)

We added clarification to Fig. S29 and the SI methods stating that the enzymes are assumed to be in their zinc-bound states. You can find the information about the buffers, preparation, and conditions for the CD and SEC experiments in SI section 4.3.

Briefly, all biophysical analyses were performed in 40 mM HEPES, 50 mM NaCl, pH 8.0 buffer with the exception of the CD experiments, which were dialyzed in 25 mM Tris, 25 mM NaCl, pH 8.0 aqueous buffer to lower the voltage of the instrument measurements at low wavelengths to reduce noise. The enzymes were assumed to purified in their metal-bound state due to the high concentration of zinc in *E. coli* and our use of a strep-tag and immobilized streptactin chromatography, which avoids strong reagents and tags that could chelate the bound zinc. Extra

zinc was supplemented at low concentrations for screening assays and kinetic analyses, but we did not supplement it for CD due to the sensitivity of the instrument voltage to metals and salts which noise the measurements at low wavelengths.

2) How many metals are bound in solution? (editor's purview)

Our experimental data, computational designs, and co-folding predictions (i.e., AF3/Chai-1) suggests that there is only 1 Zn(II) ion strongly bound in the active site for the designed metallohydrolases in solution (i.e., the catalytic Zn(II)). This question was raised by a different reviewer during the first round of peer review regarding ZETA_1, and our full response is provided below. Briefly, the only alternative binding that could occur would be nonspecific surface interactions. However, the binding data and predictions suggest that even if such binding occurred, it would be reasonable to assume independence; therefore it would not affect the Zn(II) binding affinity in the active site and is not relevant. Moreover, the crystal structures of ZETA_2 consistently reveal a single, well-defined Zn(II) binding site, with any additional binding likely reflecting promiscuous surface interactions.

Our original response to the question of whether the triple-H knockout of ZETA_1 could bind two Zn(II) ions:

We believe that there might be some weak promiscuous binding on the surface near our C-terminal streptactin tag that we expressed our designs with, which is predicted with moderate confidence by Chai-1 (Peer Review Figure 1). This promiscuous surface Zn(II) binding is predicted with higher confidence in the triple histidine -> alanine knockout Chai-1 model predicted with 1x Zn(II); in contrast, the Chai-1 model of the wild-type enzyme predicted with 2x Zn(II) lacks confidence in the predictions of both Zn(II) at once, and the predicted models with both Zn(II) ions in the active site do not look physically reasonable (the second Zn(II) lacks proper coordination).

These Chai-1 results also provide us with reasonable evidence to assume that the two Zn(II)-binding sites, if there are two at all, would be independent since they are likely to be in different regions of the protein. This allows us to fit 2 binding sites, with one binding site being set to 330 nM (corresponding to the triple-H mutant Kd for the surface site), and the other active site binding site being set as a fit parameter. These results do not deviate significantly from the currently reported data with only 1 binding site (Peer Review Table 1).

Mutant	Zn(II) Kd (1:1 Fit) (nM)	Zn(II) Kd (2:1 Fit) (nM)
ZETA_1 (A1) Wild Type	41 ± 5	41 ± 6
Triple H->A Knockout	330 ± 30	n.a.
H130A	58 ± 6	57 ± 8
H134A	105 ± 11	108 ± 16
H118A	55 ± 6	54 ± 2
N17A	36 ± 4	36 ± 2
D67A	27 ± 3	27 ± 2

Peer Review Table 1: Active site binding affinity for Zn(II) of ZETA_1 and its knockout mutants for different fit models of Zn(II):protein binding stoichiometry (1:1 and 2:1). Note that the 2:1 fit model assumes an independent, promiscuous surface binding Zn(II) site with a Kd of 330 nM.

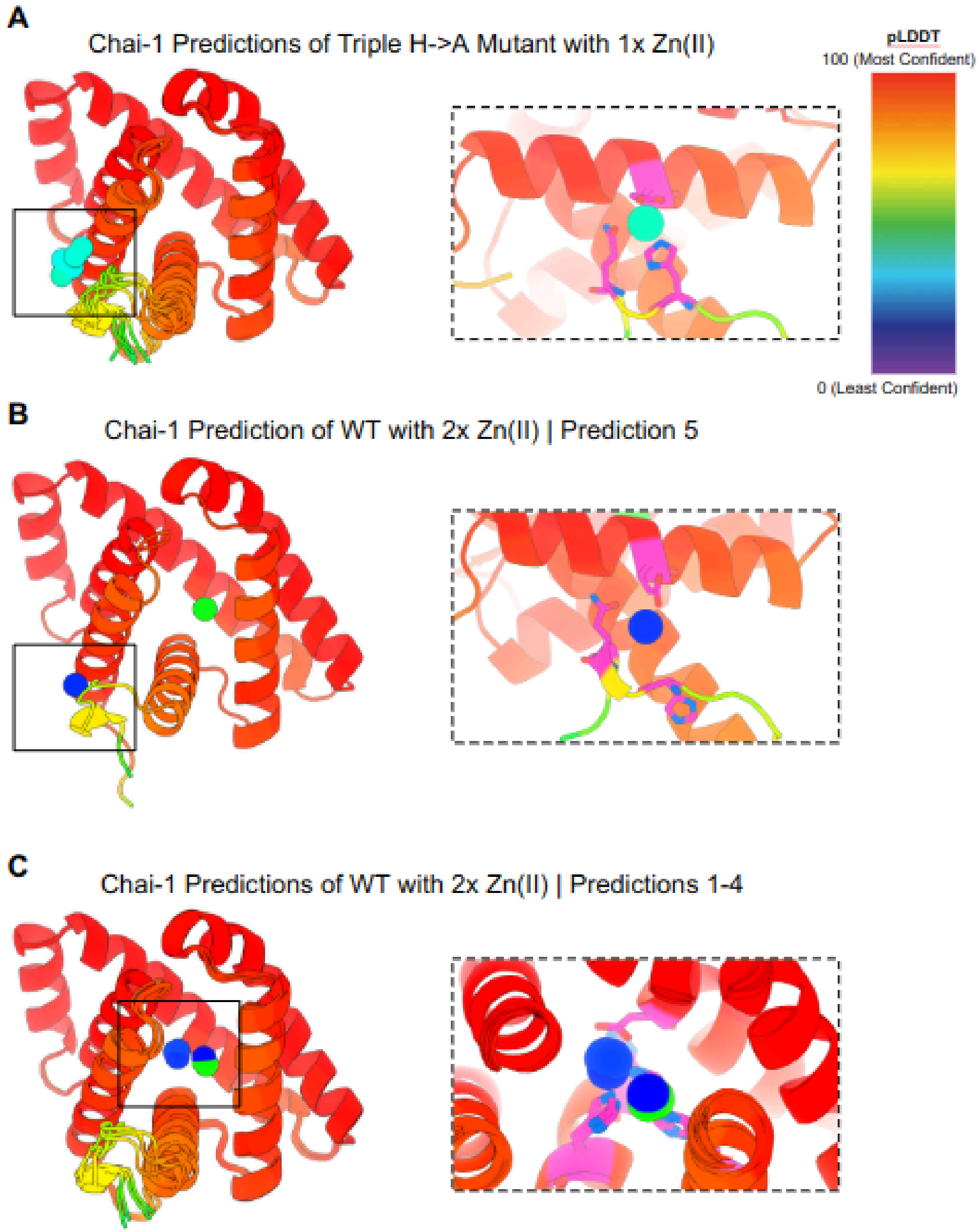

Peer Review Figure 1: Chai-1 predictions of the expressed ZETA_1 (**A**) triple H->A knockout mutant predicted with 1 Zn(II), (**B**) the wild-type predicted with 2 Zn(II) ions, one of which is on the surface near the C-terminal streptactin tag, and (**C**) the wild-type predicted with 2 Zn(II) ions both in the active site of the enzyme. The scaffold and Zn(II) ions are colored by pLDDT (local confidence), the magnified active site residues shown in sticks (right) are just colored pink except for heteroatoms. The pLDDT of the 1x Zn(II) in (A) is approximately 53, moderately confident. The pLDDT of the 2x Zn(II) ions predicted in the wild-type enzyme is very low, the properly positioned one that is coordinated by the His residues is about ~60 but the other ones are predicted with <40 pLDDT; interestingly, predicting with 2x Zn(II) in the wild-type drastically

lowers the confidence of the catalytic Zn(II) than if the structure is predicted with 1x Zn(II). We believe that these results likely suggest that there may be some weak promiscuous Zn(II) binding on the surface of the ZETA_1 enzyme near the flexible C-terminal streptactin tag, which may be enhanced in the triple histidine knockout.

3) How specific for zinc is the designed scaffold? (editor's purview)

We did not investigate the metal-binding specificity in this work. However, it is known that the Zn(II) ion in many zinc-dependent enzymes can be replaced by other metal ions, including cobalt, iron and manganese, often altering efficiency and/or activity. We would expect our proteins to behave similarly but consider such experiments beyond the scope of the current study. Nonetheless, this would certainly be an interesting question for a follow-up study.

4) Is the protein oligomeric state metal-dependent in solution, and does this impact the enzyme activity? (editor's purview)

All of the metallohydrolases were designed as monomers and observed to be uniformly monomeric by SEC with the exception of F7 and ZETA_4 which displayed some minor peaks that could result from oligomerization, although their activities were both orders of magnitude lower than the best enzymes (ZETA_1-3) (Fig. 3B, S8, S28). For F7 and ZETA_4, we only used the fraction corresponding to the monomeric peak after SEC purification for further kinetic analysis. We do not expect any potential oligomerization to be metal-dependent, as the metal-binding residues are confidently predicted to be preorganized; thus, any conformational adjustments required for Zn(II) binding should only occur on a local scale, insufficient to alter the overall protein shape to promote oligomerization. Nonetheless, it could be interesting to explicitly design for metal-dependent oligomerization for other applications in future protein design studies.

- Points 2 and 3 address a larger question about the design process: can off-design possibilities be addressed in the design stage? That is, are there residue variants that could be built that inhibit this other zinc? To address this, the zinc binding and protein oligomerization need further experimental characterization.

Fortunately, we are confident that there are no other Zn(II) ions meaningfully binding our metallohydrolases besides the designed catalytic Zn(II) ion, as addressed in our response to point 2. Additionally, our SEC data shows that our metallohydrolases are monomeric, as designed, addressed in our response to point 4.

Designing against off-design possibilities is absolutely something that can be addressed during the computational design phase. This can be accomplished in one of two ways during the design process: (1) actively prohibiting an unwanted feature during sequence or structure design step, or (2) selecting against an undesirable property during filtering. For example, we employ the first strategy to actively omit cysteine and methionine residues (besides the N-terminus Met) from ever being incorporated during sequence design because cysteines can result in disulfide oligomerization or oxidation which can change the properties of the protein, and extra methionines can result in off-target start codons which only translate part of the protein. If we did have a problem with unintended oligomerization, we could employ the second strategy and use something like AlphaFold Multimer or AF3/Chai-1 cofolding with multiple copies of the design and filter out designs whose oligomer predictions were confident. However, oligomerization was not a problem for us so we did not employ that extra filter.

5) Why is the His33 rotated ever-so slightly so its coordination to the zinc is not quite ideal? (additional clarification in the text might be warranted)

Please note that we provided the incorrectly numbered PDB files for review and that the true numbering should be N-2 to be consistent with the manuscript; thus, His33 will be referred to as His31 to be consistent with the manuscript. We apologize for the confusion.

We are not sure why the His31 coordination to Zn(II) is suboptimal. We suspect this may be an artifact of the tight crystal packing against a neighboring protein subunit, which forces the helix containing H31 to shift slightly from its designed position and have a suboptimal coordination to Zn(II) (Peer Review Figure 2). Additionally, the absence of H110 from the active site, due to its flipped-out orientation, appears to have caused the Zn(II) ion to shift toward that vacant space, which may have further constrained the active-site packing and the coordination geometry. However, this could also reflect some minor inaccuracies with the designed structure.

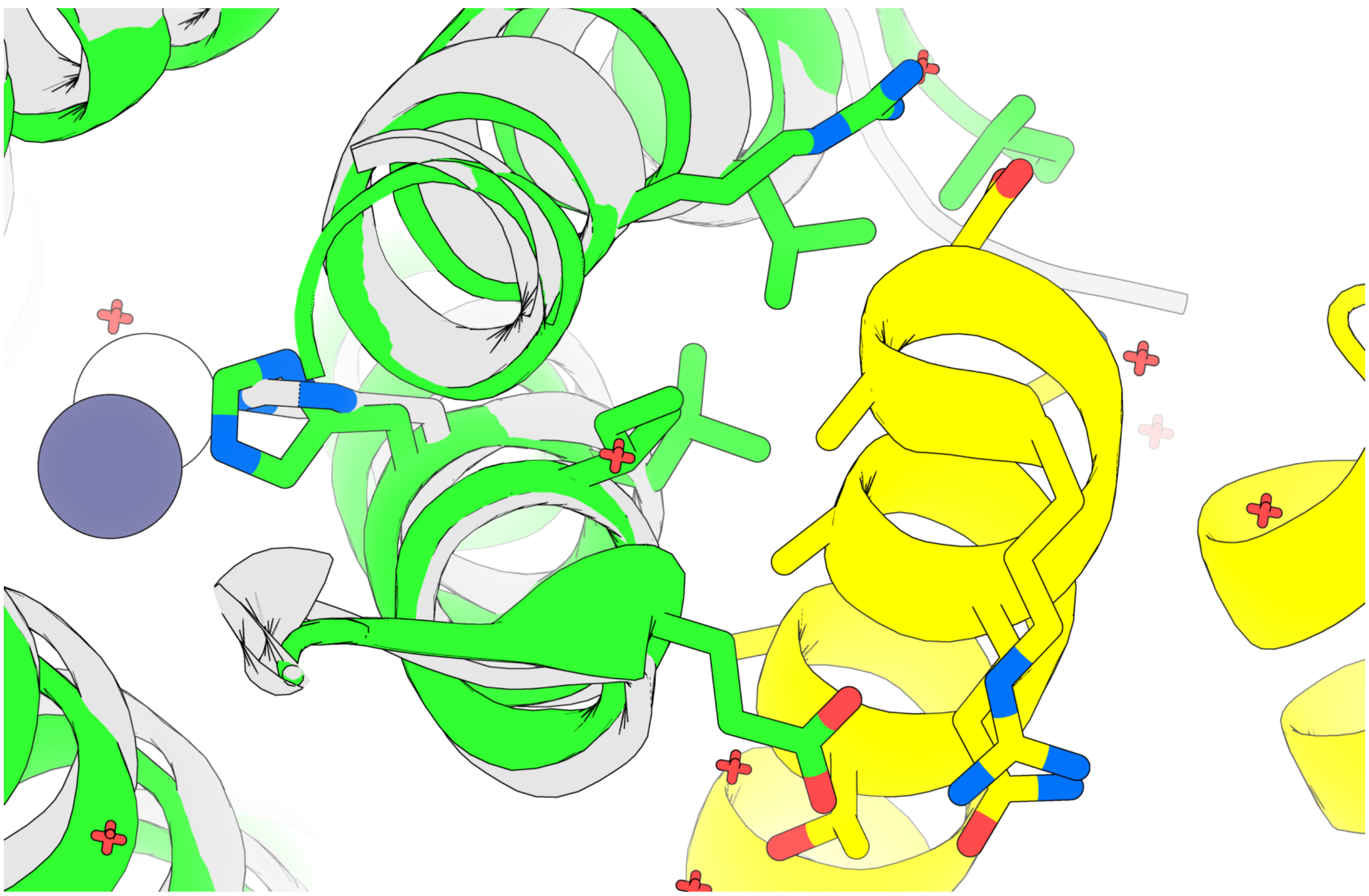

Peer Review Figure 2: Tight crystal packing interface showing the distortion of the helix containing H31 (shown as green sticks) from the design model (shown in grey). Colored structures represent different crystal subunits; the grey Zn(II) denotes the ion observed crystallographically, the white Zn(II) marks the design-model position, and the gray structure represents the design model.

6) Are all the supplemental figures cited? For example, I cannot find Figs S2, S17, or S19. I also do not think they are called in the order presented in the supplemental document (please check carefully)

Thank you for pointing this out, we have updated our text to align with the editor's guidelines of

citing all supplemental figures in order.

7) Fig. 5's legend needs a small edit – panels b and c are called together, then (b) is explained but (c) is not. (minor edits)
"(B,C) Active site zoom-in highlighting the agreement between the experimental and designed catalytic geometry (B) and the shape-complementarity of the designed binding pocket and the transition state model (superimposed in from the design model). Crystal structure shown in grey and design model in color in all panels."

Thank you for catching this error, we have corrected the caption of Figure 5 to refer to (B) and (C) now.
