# Supplementary material for: Computational design of metallohydrolases
Source: Nature. 2025 Dec 3;649(8095):246–53. doi: 10.1038/s41586-025-09746-w (PMC12727532; doi:10.1038/s41586-025-09746-w)
Supplement: Supplementary file 2 [file 41586_2025_9746_MOESM1_ESM.pdf]

nature portfolio

https://doi.org/10.1038/s41586-025-09746-w

Supplementary information

Computational design of metallohydrolases

In the format provided by the
authors and unedited

TABLE OF CONTENTS

TABLE OF CONTENTS 1
1. NOMENCLATURE 3
2. EXTENDED DISCUSSION 4
2.1. Design Campaign 1 Analysis and Discussion 4
2.2. Design Campaign 2 Analysis and Discussion 11
3. GENERAL MATERIALS 15
4. METHODS 17
4.1. Computational Design of Metallohydrolases 17
4.2. Changes in the Second Design Campaign of Metallohydrolases 26
4.3. Experimental Biology & Biochemistry 32
5. SUPPLEMENTAL FIGURES 46
Fig. S1. Detailed flow chart of the design and experimental pipeline for de novo metallohydrolase design in the first design campaign 46
Fig. S2. Theozyme input generation for RFdiffusion2 and the challenge of enumerative catalytic motif scaffolding 47
Fig. S3. Structural panel of the 96 ordered designs campaign spanning 96 unique backbones from the first design campaign 48
Fig. S4. SDS-PAGE gel results for 96 designs expressed and purified in parallel from the first design campaign 49
Fig. S5. Initial metallohydrolase activity screen with the purified designs from the first design campaign 50
Fig. S6. SDS-PAGE gel results and SEC chromatograms of the purified hits from the first design campaign 51
Fig. S7. Electrospray ionization mass spectrometry for A1 (ZETA_1) 52
Fig. S8. Total turnover lower bound experiment for A1 (ZETA_1) and calibration curve 53
Fig. S9. In silico metric distributions for the ordered designs in the first design campaign 54
Fig. S10. Comparison of AlphaFold2 metrics with PLACER metrics and Chai-1 metrics for ordered designs from the first design campaign 55
Fig. S11. Computational and experimental comparison of A1 (ZETA_1) against other sequences identified for that backbone during design 56
Fig. S12. SDS-PAGE gel results and SEC spectra for the purified A1 (ZETA_1) knockout mutants expressed at scale 57
Fig. S13. Kinetic characterization and Chai-1 predicted structures of the A1 (ZETA_1) knockout mutants with the proposed role of residue N17 58
Fig. S14. Zinc-binding affinity experiments for A1 (ZETA_1) and its knockout mutants 59
Fig. S15. Chai-1 prediction distributions with different ligands for the active and inactive designs from the first design campaign 60
Fig. S16. PLACER metrics of ordered designs from each design campaign 61
Fig. S17. Structural panel of the 96 ordered designs spanning 37 unique backbones from the second design campaign 62

Fig. S18. SDS-PAGE gel results for the 96 designs of the second design campaign expressed and purified in parallel.......... 63
Fig. S19. Initial metallohydrolase activity screen with the purified designs for the second design campaign.......... 64
Fig. S20. SDS-PAGE gel results and SEC chromatograms and ESI mass spectrometry plots of ZETA_2-4.......... 64
Fig. S21. Circular dichroism plots for all characterized enzymes across multiple temperatures.......... 66
Fig. S22. AF2/Chai-1/PLACER metric distributions for first design campaign.......... 67
Fig. S23. AF2/Chai-1/PLACER metric distributions for ZETA_1 (A1) variants.......... 68
Fig. S24. AF2/Chai-1/PLACER metric distributions for second design campaign.......... 69
Fig. S25. AF2/Chai-1/PLACER metric distributions for ZETA_2 (H10) variants.......... 70
Fig. S26. Histograms of AF2 metrics from the first design campaign comparing designs with and without RFjoint2 inpainting applied.......... 71
Fig. S27. Uncropped gel images used in manuscript.......... 72
6. SUPPLEMENTAL TABLES.......... 73
Table S1. Electrospray ionization mass spectrometry results for the characterized hits and A1 mutants.......... 73
Table S2. Comparing the catalytic efficiencies of different designed and engineered (metallo)enzymes.......... 74
Table S3. Pocket metric statistics from the first design campaign.......... 76
Table S4. Expressed amino acid sequences for the top 5 characterized designs from the first design campaign.......... 77
Table S5. Expressed amino acid sequences for the top 11 characterized designs from the second design campaign.......... 78
7. SUPPLEMENTAL VIDEOS.......... 79
Video S1. RFdiffusion2 trajectory for ZETA_1 (A1).......... 79
Video S2. PLACER predictions for substrate ensemble of ZETA_1 (A1).......... 79
Video S3. PLACER predictions for catalytic residue ensemble of ZETA_1 (A1).......... 79
Video S4. PLACER predictions for substrate ensemble of the inactive design H8.......... 79
Video S5. PLACER predictions for catalytic residue ensemble of the inactive design H7.... 79
8. SUPPLEMENTAL DATA.......... 80
Data S1. Coordinates for all transition state models calculated by density functional theory.... 80
Data S2. Protein and DNA sequences for all ordered designs.......... 80
Data S3. PDB design models for all ordered designs.......... 80
9. REFERENCES.......... 81

1. NOMENCLATURE

Enzymes

We interchangeably refer to the best enzyme from the first design campaign as A1 *and* zinc metalloesterase 1 (ZETA_1) throughout the supplemental information. The name A1 came from its commercially ordered gene position in a 96-well eBlock (Well ID: A1). However, for the purpose of standardization, we would appreciate it if any future references would use **ZETA_1** as the official name.

The second design campaign featured 11 major hits among 3 completely different scaffolds. After characterizing the kinetics of all 11 hits, we picked the design with the highest catalytic efficiency (k_{cat}/K_M) within each of the 3 scaffold families to represent them. These designs were named ZETA_2, ZETA_3, and ZETA_4 (ordered by catalytic efficiency, where ZETA_2 has the highest). Note that ZETA_2 has the highest catalytic efficiency and rate enhancement of every design in this manuscript, even surpassing ZETA_1.

ZETA_1-4 all have unique scaffolds made from scratch around theozymes with RFdiffusion2. As mentioned above, for the purpose of standardization, we would appreciate it if these enzymes were individually referred to as **ZETA_1**, **ZETA_2**, **ZETA_3**, and **ZETA_4** (or **ZETA_1-4** if referring to all) in any future references.

2. EXTENDED DISCUSSION

Detailed analyses of the design models and associated computational metrics were conducted for each design campaign, with summaries provided in Fig. S22-S25. The following sections discuss key observations from these analyses and present our hypotheses on why the top-performing designs excelled in each campaign. The trends observed in the design models should guide future strategies for metallohydrolase design, while the computational metric patterns provide valuable insights for developing effective filters to enrich and select for active designs.

2.1. Design Campaign 1 Analysis and Discussion

The results of design campaign 1 primarily span Fig. S1 to Fig. S15 and Extended Data Fig. 1 to Fig. 5. Computational metric analysis referenced in this discussion primarily spans Fig. S22 and Fig. S23.

General trends among the top 5 hits

Design model analysis:

We first noted several trends about the theozymes that the design models of the top 5 hits were designed from, which informed the second design campaign and is described more extensively in section 4.2. (1) All of the hits except for F7 came from the class of DFT theozymes using Zn(II) to stabilize the oxyanion, rather than 2 protein hydrogen bonds. (2) The 4 hits using Zn(II) to stabilize the oxyanion were derived from the DFT theozyme of the *S*-enantiomer of the hydroxide attack. (3) The design models of every hit coordinate the Zn(II) with the N_ε atom of the His residues, except B9 which has 2/3 His residues coordinating Zn(II) with their N_ε atom and the other His residue coordinating with its N_δ atom.

We believe the Zn(II)-stabilized theozymes were favored for activity because it was difficult to make quality designs with the other class of theozymes: protein oxyanion holes featuring 2 hydrogen bonds to the oxyanion (Fig. S2). This was especially difficult to do during sequence design since only the histidine residues were fixed during scaffold generation; thus, the protein backbones were not necessarily optimized for the introduction of nearby residues around these theozymes which could make 2 hydrogen bonds to the oxyanion, let alone positioning them in the optimized hydrogen bonding geometry from DFT with sufficient preorganization. Even if the oxyanion hole could be formed, these scaffolds also required the introduction and precise positioning of a general base during sequence design. Satisfying all these constraints during sequence design turned out to be nearly impossible, and thus we only required 1 hydrogen bond to the oxyanion for designs with this class of theozyme to pass; however, this introduces inconsistencies with the DFT model as the geometry of the transition state should change with only 1 hydrogen bond instead of 2. Therefore, it is likely that these designs were modeling a transition state geometry that was not optimal for only a single hydrogen bond stabilizing the oxyanion. In contrast, the Zn(II)-stabilized theozymes only required the introduction of a general base during sequence design, which was difficult but not

impossible. These final designs modeled all the correct interactions present in the DFT calculations such that the design was optimized around a correct model of the reaction transition state. In retrospect, we believe that all catalytically relevant protein groups should have been explicitly modeled from the beginning during RFdiffusion2 such that scaffolds were generated already positioning them with the geometry from quantum chemistry models of the transition state (i.e., 3xHis for Zn(II) coordination, 1xGlu/Asp for the general base, and combinations of residues satisfying 2 hydrogen bonding interactions to the oxyanion).

Regarding the Zn(II)-stabilized oxyanion theozymes, we were unsure why the active designs only came from those modeling the *S*- enantiomer of nucleophilic hydroxide attack. We hypothesize that this outcome could be due to random chance or reflect a scenario in which some designs modeling hydroxide attack from the *S*- enantiomer direction experimentally undergo nucleophilic attack from the opposite face. For instance, evidence suggests that the proposed general base residue in ZETA_1 does not function as initially designed (Fig. 3i). It is plausible instead that the hydroxide attacks from the opposite, more solvent-exposed side, which also coincides with the proposed substrate entrance tunnel.

The additional observation of the mostly all N_ε coordination with Zn(II) for the catalytic histidine residues was unsurprising given this frequently observed geometry in natural metallohydrolases (e.g., PDB: 1AST (pdb_00001ast)). Furthermore, the designed coordination geometry of B9, with 2/3 His residues coordinating with N_ε and the other His residue coordinating with N_δ, is also frequently observed in natural metallohydrolases (e.g., PDB: 2VVB (pdb_00002vvb)). It is much less common to see anything different from these coordination geometries for native metallohydrolases that coordinate Zn(II) with three histidine residues, and thus it was perhaps suboptimal that we still searched this space (e.g., N_δ-N_δ-N_δ, N_δ-N_ε-N_δ, N_ε-N_δ-N_δ). The consistency between our active designs and what is frequently observed in nature with this respect could reflect an energetic advantage related to scaffold geometry and the coordination of Zn(II) with three histidines. Alternatively, this could simply be due to bias from what is structurally represented in the PDB affecting the ML-based methods' performance for designing and predicting with these different coordination modes.

We additionally noted several trends in the global structural features of the top 5 hits. (1) The sequence lengths of the hits were all relatively short compared to the sequence lengths sampled during design and what was ordered. (2) The active hits tended to have a high degree of alpha-helical secondary structure. (3) The histidine residues tended to be on a defined secondary structure. (4) There was a large and obvious entrance tunnel/pocket for the substrate. None of these trends were particularly surprising, although it was interesting that we generated scaffolds between 130-275 amino acids in length but 4/5 hits were <175 amino acids in designed length, with A8 as the exception only being 206 amino acids in designed length.

Computational metric analysis:

Comparison of computational metrics between active and inactive designs from the first design campaign revealed notable shifts in their distributions (Fig. S22).

Analysis of the AlphaFold2 (AF2) predictions and metrics for the apo monomeric protein folds revealed several differences in metric distributions between active and inactive designs (Fig. S22A). (1) The C_α RMSD between the design models and AF2 predictions (rmsd_to_input) was notably lower, on average, for active designs compared to inactive ones. (2) The RMSD of C_α and C_β atoms for the three catalytic histidine residues (cat_HIS_ca_cb) also tended to be slightly lower for active designs, though this difference was less pronounced than the overall C_α RMSD shift. (3) The mean pLDDT scores from AF2 predictions were higher for active designs on average, indicating greater confidence in their structural models. (4) The mean predicted alignment error (PAE) for the apo monomer was lower for active designs, on average, suggesting more consistent domain positioning in the active designs. No significant differences were observed in the distributions of pTM scores or AF2 recycle convergence metrics between active and inactive designs. Ultimately, the noted distribution shifts were not surprising and completely rational. Altogether, they suggest that the active designs had highly confident AF2 predictions that were structurally consistent with the design models; perhaps this suggests that these active designs were more preorganized for catalysis, on average, than the inactive designs and that this was a main failure mode in the inactive designs.

Analysis of the Chai-1 ensemble predictions for the protein, Zn(II), and transition state phosphoanalog (TSA) revealed several notable shifts in metric distributions between active and inactive designs (Fig. S22B). (1) The average RMSD of the TSA between the design model and the five Chai-1 predictions (TSA_lig_rmsd) was consistently lower for active designs, indicating better alignment between the predicted and designed substrate positions. (2) The averaged mean pLDDT scores across the five ensemble predictions were higher, on average, for active designs. This was the case in all predicted components of the ensemble (i.e., protein pLDDT, Zn(II) pLDDT, and TSA pLDDT), although the TSA pLDDT shift was significantly more pronounced. Nonetheless, this reflects greater model confidence in all key components of the catalytic site. (3) The mean predicted TM-score (pTM) and interface pTM (ipTM) of the five Chai-1 ensemble predictions were both elevated in the active designs, on average, with ipTM showing a significantly more pronounced difference, suggesting better-defined interactions in the complexes of the active designs. (4) All averaged PAE scores (protein-Zn(II) PAE, protein-TSA PAE, and Zn(II)-TSA PAE) were lower on average for the active designs than for the inactive designs, indicating higher confidence in the positions of these components with respect to each other. Ultimately, these distribution shifts between the active and inactive designs were also not surprising and completely rational. As with the AF2 metrics, they suggest that the ensemble predictions of the active designs were structurally consistent with the design models, highly confident in the Zn(II) and TSA docking, and highly confident about the interactions between all of these components.

Analysis of the PLACER ensemble predictions for the protein and substrate additionally revealed several shifts in metric distributions between active and inactive designs (Fig. S22C). (1) The average RMSD between the predicted ligand placement in the ensembles and the design model was notably lower in the active designs, on average, than in the inactive designs suggesting a higher degree of substrate preorganization and binding in the active site. (2) The average predicted RMSD (uncertainty) of the pocket residues (u_pocket) was notably lower in

the active designs, on average, than in the inactive designs which further suggests high preorganization in the active site. (3) Although subtle, the distributions of the “pnear” confidence metrics from PLACER are slightly higher, on average, for the active designs suggesting higher local confidence across all the PLACER ensemble predictions. Ultimately, these distribution shifts suggest that PLACER predictions of the substrate in the active site help to assess preorganization and that designs whose ensembles are predicted with high confidence and structural agreement with the design model may tend to be more active.

<u>Hypothesizing why A1 (ZETA_1) was drastically better than the other 4 hits</u>

Design model analysis:

ZETA_1 is the smallest structure out of the 5 hits from the first order (148 amino acids), which is interesting because we already noticed a trend that the hits are relatively small compared to the sequence lengths sampled and ordered (130-275 amino acids). Furthermore, ZETA_1 has the shortest sequence distance between two histidines out of the hits from the first order, as it contains two histidines that are 3 residues apart from each other on an alpha-helix (i.e., HXXXH). In contrast, the catalytic histidines of the other 4 hits are all spaced out further in sequence space and in all cases, each of the three histidines occupies its own unique secondary structure (i.e., no two histidines are on the same alpha-helix/beta-sheet/loop).

Notably, HEXXH is a common and well-known motif for several metalloenzymes, where both histidines are metal-coordinating residues and the motif lies on an alpha-helix. Although the ZETA_1 motif does not contain glutamate, which is frequently a general base in these metalloenzyme motifs, the histidine preorganization in sequence space with the alpha-helical secondary structure may confer optimal entropic benefits to lower the Gibbs free energy of the enzyme-metal complex and the enzyme-TS complex. Additionally, Zn(II) has a flexible coordination geometry because of its unique electronic properties resulting from a completely filled 3d orbital, allowing it to transition from (distorted) tetrahedral geometries to penta/hexacoordinate geometries, such as the case in metallohydrolase reactions; thus, the H(E)XXH motif on alpha-helices may also play a subtle dynamic role that efficiently allows the His-Zn(II) coordination geometries to shift as the Zn(II) goes from tetracoordinate to pentacoordinate in the transition state.

Beyond the HXXXH motif, there is nothing immediately obvious about what makes ZETA_1 a much more competent enzyme than the other 4 hits. It does feature a relatively buried and hydrophobic active site compared to the other hits, but it is not an outlier in these metrics when compared to all of the ordered designs (Table S3). We suspect that ZETA_1 may have some dynamic properties and flexibility because of its peculiar unfolding behavior (Fig. 3c-d), but this is not obvious from the design model.

Computational metric analysis:

The specific metrics for ZETA_1 are shown as the vertical dashed red line among the metric distributions for the active and inactive designs in Fig. S22, allowing its position in the distributions to be clearly identified.

Overall, ZETA_1 consistently ranks among the top performers across all metrics from AF2, Chai-1, and PLACER, suggesting that optimizing multiple design parameters simultaneously is critical for achieving highly active designs. AF2 predictions for ZETA_1 show the lowest RMSD relative to its design model, with rapid convergence across recycling steps, as indicated by a low convergence RMSD. Its predicted structure also ranks among the most confident, with high mean pLDDT scores and low mean PAE. Together, these results suggest that the ZETA_1 scaffold is highly preorganized, adopting the precise geometry it was designed for to coordinate Zn(II) and enable catalysis.

The Chai-1 predictions further corroborate this analysis. Both the transition state analog (TSA) and Zn(II) in ZETA_1 were predicted to dock with exceptional positional agreement to the design model. Confidence in these predictions was consistently high, as reflected by the separate pLDDT scores for the protein, Zn(II), and TSA, along with elevated pTM scores for the complex. Strikingly, the interface pTM ranked among the highest across all designs, suggesting that the interactions between the enzyme, Zn(II), and TSA were optimal. Additionally, the PAEs for the protein, Zn(II), and TSA combinations were low. Collectively, these metrics indicate that the interactions between the protein, Zn(II), and TSA in ZETA_1 closely match the intended design, supporting an optimally preorganized active site that tightly binds the transition state of the 4MU-PA hydrolysis reaction.

Finally, the PLACER metrics for the protein-substrate ensembles further demonstrate strong agreement between the design model and predicted structures within the active site, both for the ligand and surrounding pocket residues. Notably, the confidence of these predictions is substantially higher than the broader design set, particularly in the “pnear” metrics, which capture local structural and interaction confidence across all PLACER ensembles for each design. These elevated “pnear” scores specifically reflect highly confident preorganization of the pocket residues and precise substrate positioning, suggesting that the active site is optimally arranged to bind the substrate in the correct orientation for hydrolysis.

Ultimately, the computational metrics paint a clearer picture as to why ZETA_1 was significantly more active than the other 4 hits. Multiple prediction methods capturing different redundant and nonredundant features about ZETA_1 all arrive at the consensus that it is highly preorganized and matches its designed structure with extreme atomic precision in all aspects. Although the structural reasons for this may not be obvious by eye, it is clear that one must validate their enzymes with a diversity of metrics and methods to capture different aspects of it; highly active enzymes will likely come from designs that optimize nearly every possible metric from these prediction methods.

Hypothesizing why A1 (ZETA_1) was the best out of its retrospectively ordered variants

Design model analysis:

ZETA_1 was found to be the best enzyme out of its 7 retrospectively ordered variants. Nonetheless, variants 6 and 7 were found to be moderately active, variant 5 had minor activity, variant 4 had extremely minor activity, and variants 1-3 were inactive (Fig. S11C). Interestingly, variants 5-7 belong to a different scaffold resample than ZETA_1 and variants 1-4, which all

belong to the same resampled scaffold; variants 5-7 are one amino acid shorter because of a shortened loop region near the 79th residue. Thus, not only is ZETA_1 the most active design, but it is the only design with notable activity for its scaffold resample.

Variant 4 is the closest protein in sequence identity to ZETA_1, sharing 80% (Fig. S11D), and its pocket sequence identity is almost a perfect match. Every residue directly contacting the theozyme in the pocket is exactly the same, and thus it is peculiar why their activities are vastly different. Thus, we hypothesize that variant 4 is significantly less active than ZETA_1 due to differences in its global sequence outside the active site, which likely lead to deviations in its overall fold from the design model and/or disrupt the preorganization and geometry of its catalytic histidine residues.

Upon inspection of the active sites for all variants, we did identify a common feature in the active designs (ZETA_1; variants 4–7): aromatic residues were consistently present at positions 28 and 100 in ZETA_1 and variant 4, or at equivalent positions 28 and 99 in variants 5–7. Likewise, no aromatic amino acids were present at positions 28 and 100 in the inactive variants 1-3, which all contained S28 and L100. These aromatic amino acids contact the aromatic groups in the substrate and are presumably important for binding through pi-pi interactions. Specifically, residue 28 (Y28 in ZETA_1 and variant 4; W28 in variant 5; F28 in variants 6–7) interacts with the 4MU coumarin leaving group of the substrate in a perpendicular edge-to-edge orientation, while residue 99/100 (F100 in ZETA_1 and variant 4; L99 in variant 5; F99 in variants 6–7) engages the phenyl moiety of the substrate in an approximate edge-to-face orientation. We hypothesize that the phenylalanine pi-pi interaction with the phenyl group of the substrate plays an especially critical role in substrate binding, as a similar interaction is observed in the most catalytically efficient serine hydrolase design for 4MU-PA, momi120_103.[1] In the momi120_103 design, the phenylalanine engages the substrate in a face-to-face pi-pi interaction, which increases the contact surface area and enhances substrate affinity, consistent with its remarkably low K_M of 260 nM, compared to the ZETA_1 K_M of 32,000 nM.

Computational metric analysis:

The comparison of ZETA_1 to its 7 variants and the metric distribution of the active variants and inactive variants are shown in Fig. S23.

Somewhat surprisingly, the metrics among ZETA_1 and the variants were quite similar, on average, but there were a few important differences. AF2 predictions show that ZETA_1 and the other two active variants, variants 6-7, had lower RMSDs relative to their design models compared to the inactive variants, on average, with ZETA_1 demonstrating the lowest RMSD. Their AF2 predictions were also slightly more confident, on average, with respect to mean pLDDT and mean PAE. However, we were particularly surprised to see very little differences in the Chai-1 prediction metrics between ZETA_1 and the other variants. The most discriminating metrics for separating ZETA_1 from the less active variants and inactive variants in this analysis came from PLACER. Specifically, the substrate RMSD in the PLACER ensembles was much lower in ZETA_1 than in any other variant. Furthermore, every "pnear" metric was significantly higher for ZETA_1 than any other variant design, indicating higher confidence and convergence

in the distribution of predicted PLACER ensembles. Although it is still wise to choose designs that perform well over many metrics from diverse prediction methods, this analysis would suggest that PLACER may be particularly useful for separating active and less active designs for sequence variants within a scaffold family.

2.2. Design Campaign 2 Analysis and Discussion

The results of design campaign 2 primarily span Fig. S16 to Fig. S21 and Extended Data Fig. 6 to Fig 8. Computational metric analysis referenced in this discussion primarily spans Fig. S24 and Fig. S25.

General trends among the top 11 hits

Design model analysis:

It should first be noted that our screening threshold for identifying active designs was much more stringent than the first design campaign. We identified 11 significantly active designs, which were subsequently characterized (Fig. 4b). However, we believe that this was a highly conservative estimate, as there seem to be many designs with active traces above background, potentially comparable with the four lower-activity hits from the first campaign (Fig. 4b and Fig. S19).

We observed only one notable trend among the theozymes used to generate the design models of the top 11 hits, though this campaign involved considerably less theozyme diversity (see Section 4.2 and Fig. 4a): all the hits originated from theozymes that modeled glutamate, rather than aspartate, as the proposed general base. Surprisingly, we noticed a significant bias toward structures designed with the glutamate general base throughout the entire second design campaign, as the structures with the aspartate general base struggled to pass filters with the same rates. We thought that the additional flexibility of the glutamate may have made it harder to keep positioned during design, but this was not the case. It is also likely that in the mostly helical scaffolds, the aspartate general base struggles to adopt the intended conformation, and rather reorients itself to form a hydrogen bond with the protein backbone. Besides this observation, there was not much variation in the theozymes to note for this design campaign, except for histidine and 4MU ring-flip conformers, which there seemed to be no obvious trend among the 11 major hits. All of the histidines were designed to coordinate Zn(II) with their N_ε atoms, the theozymes all had the Zn(II) oxyanion stabilization, and modeled the *S*-enantiomer of the hydroxide attack.

It is worth mentioning that nearly all designs ordered in the second design campaign contained an HXXXH motif located on an alpha-helix. Of the 11 significantly active hits from the second design campaign, 6 carried this HXXXH motif (ZETA_2 family + ZETA_4), and the other 5 carried a nearly identical HXXXXH motif on a bent alpha-helix (ZETA_3 family). Interestingly, certain designs, such as ZETA_2, even featured the fully canonical HEXXH motif; however, in these cases, the glutamate within the HEXXH sequence is not proposed as the general base, contrary to its typical role in native enzymes. Instead, these designs utilize a different glutamate residue as the intended general base, as the geometry of the HEXXH glutamate residue in these designs appears incompatible with the catalytic function of deprotonating the water molecule polarized by Zn(II).

Computational metric analysis:

Comparison of computational metrics between active and inactive designs from the second design campaign revealed some shifts in their distributions, but less noticeable than the first design campaign (Fig. S24).

Analysis of the AlphaFold2 (AF2) predictions and metrics for the apo monomeric protein folds revealed few differences in metric distributions between active and inactive designs (Fig. S24A). The only notable difference that we identified was that the RMSD of C_α and C_β atoms for the three catalytic histidine residues (cat_HIS_ca_cb) tended to be lower for active designs than the inactive ones. However, the activity threshold used to define "active" designs in this campaign was more stringent, meaning that several less active, but still functional, designs were likely classified as "inactive," potentially skewing the inactive distributions. Despite this, most of the ordered designs, including the active hits, displayed strong AF2 metrics. This suggests that favorable AF2 metrics may serve as a reasonable prerequisite for identifying promising enzyme candidates, though overly stringent filtering based solely on these metrics may not be necessary or even harmful. Furthermore, we hypothesize that AF2 metrics may be useful for selecting between scaffolds, but may lack power for discriminating between active and inactive sequences within the same scaffold; this design campaign only featured 37 unique scaffolds whereas the first design campaign featured 96 unique scaffolds, which could also explain why there were more observed differences in the AF2 metric distributions between the active and inactive designs in the first design campaign. This hypothesis is supported by the analysis of the ZETA_1 variants, which did not demonstrate drastically different AF2 metrics (Fig. S23A).

Analysis of the Chai-1 ensemble predictions for the protein, Zn(II), and transition state phosphoanalog (TSA) revealed some notable differences in metric distributions between the active and inactive designs (Fig. S24B). (1) The average RMSD of the TSA and Zn(II) between the design model and the five Chai-1 predictions was consistently lower for active designs, indicating better alignment between the predicted and designed substrate and Zn(II) positions. (2) The averaged mean pLDDT scores across the five ensemble predictions tended to be higher for active designs. This was especially true for the protein pLDDT, Zn(II) pLDDT, and catalytic residue pLDDT. (3) The mean predicted TM-score (pTM) and interface pTM (ipTM) of the five Chai-1 ensemble predictions were elevated in the active designs, on average, with ipTM showing a much more pronounced difference, suggesting better-defined interactions in the complexes of the active designs. (4) The protein-Zn(II) and Zn(II)-TSA averaged PAE scores were lower on average for the active designs than for the inactive designs, indicating higher confidence in the positions of these components with respect to each other. Ultimately, these highlighted Chai-1 metric distribution shifts from the second design campaign were very similar to those of the first design campaign. These particular metric shifts suggest that the ensemble predictions of the active designs were consistent with the design models and confident about protein-Zn(II)-TSA complex and interactions, which may indicate better preorganization and tighter binding affinity for the transition state.

Analysis of the PLACER ensemble predictions for the protein and substrate additionally revealed several shifts in metric distributions between active and inactive designs that were

similar to this analysis in the first design campaign (Fig. S24C). (1) The average RMSD between the predicted ligand placement across the PLACER ensembles and the design model was lower for active designs, on average, suggesting a higher degree of substrate preorganization and binding accuracy within the active site. (2) The average predicted RMSD (uncertainty) of the pocket residues (u_pocket) was notably lower for active designs, further indicating tighter preorganization of the active site environment. (3) Although more subtle, the distributions of the "pnear" confidence metrics were slightly elevated for active designs, suggesting greater consistency and convergence across predictions. Altogether, these shifts imply that PLACER ensemble predictions provide valuable insight into substrate preorganization, and that designs exhibiting high structural agreement and confidence relative to the design model may be more likely to exhibit catalytic activity.

Hypothesizing why ZETA_2 (H10) was the best enzyme for its scaffold (out of 9 designs)

Design model analysis:

ZETA_2 (H10) emerged as the top-performing enzyme among 9 designs derived from its scaffold within the 96 designs ordered in the second campaign. Four additional designs from this scaffold (F11, H1, F8, F2) were identified as major hits and further characterized (Fig. 4b,d-e). While not classified as major hits, activity screening (Fig. S19) indicated that F7 was also highly active, C8 showed moderate activity, H3 was inactive, and H7 failed to clone or express. Every design came from the same resampled scaffold except for C8, whose resampled scaffold has an extra residue and slightly different loop geometries connecting the alpha-helices.

The active sites of these designs are remarkably similar, but several key features appear to contribute to the exceptional activity observed in ZETA_2 (H10) and the four other characterized designs. (1) A threonine at position 55 is ideally positioned to form a hydrogen bond with the oxyanion intermediate, potentially lowering the transition-state energy beyond the intended stabilization by Zn(II) alone. T55 is conserved among the five most active designs from this scaffold. Conversely, designs C8 and F7, which contain leucine residues (L56 and L55 respectively) incapable of hydrogen bonding, show reduced activity. However, since the inactive designs H3 and H7 also contain T55, this residue alone cannot fully explain the enhanced catalytic performance. (2) ZETA_2 features four surface arginine residues closely surrounding the 4MU leaving group, complemented by additional positively charged residues further from the active site. Although other designs, both active and inactive, also feature arginines in this region, subtle differences in their precise placement and surrounding negatively charged residues may alter the local electrostatic environment. We hypothesize that ZETA_2 optimizes its local net charge and electric field distribution, thereby stabilizing the leaving group and potentially enhancing substrate polarization. (3) A tyrosine residue at position 81 appears to form a face-edge pi-pi interaction with the 4MU moiety of the substrate, while simultaneously accepting a hydrogen bond from H106, which coordinates Zn(II). This dual interaction likely contributes to both substrate binding and preorganizing H106. Y81 is strictly conserved among the five most active designs, whereas the less active C8 and F7 contain H82 and A81, respectively, residues that do not facilitate this critical interaction network.

Beyond these observations, there are no immediately apparent reasons why ZETA_2 (H10) outperforms the other designs from this scaffold. These designs share high sequence identity, particularly within the active site. Interestingly, the active sites across these designs are enriched with aromatic residues and exhibit an intertwined network of interactions, namely hydrogen bonding and pi-pi stacking, that we hypothesize contributes to optimal preorganization for catalysis. The preorganization in ZETA_2 is much more visually obvious to us in the design models than in ZETA_1 or ZETA_3-4.

Computational metric analysis:

The comparison of ZETA_2 to the 8 other ordered designs from its scaffold, along with the metric distributions for the predominantly active and inactive designs, is shown in Fig. S25. As noted previously, many of the other designs are not entirely inactive; however, for the purposes of this analysis, the results were divided into two groups.

As noted with the comparison of ZETA_1 to its variants, the metrics among ZETA_2 and its less active and inactive designs with the same scaffold had similar metrics, on average. AF2 predictions showed that all nine designs from this scaffold exhibited strong metrics with relatively little variation among them. ZETA_2 displayed the highest mean pLDDT and the second lowest mean PAE across the set; however, these differences alone did not clearly explain its superior performance, and there were no notable differences between the distributions of the highly active design distributions and “inactive” design distributions. Analysis of the Chai-1 predictions similarly showed that all designs exhibited excellent metrics with minimal variation across the set. No clear patterns emerged to explain why ZETA_2 outperformed the other designs. In fact, the distributions of most metrics between the highly active and “inactive” designs were largely indistinguishable, and in some cases, the active designs displayed slightly worse metrics, such as higher Zn(II) RMSD, though these values remained exceptionally low overall.

Interestingly, the most discriminating metrics for separating active from inactive variants, and for distinguishing ZETA_2 from the less active designs within its scaffold family, were provided by PLACER. This is consistent with the observations for ZETA_1 and its variants. Specifically, the average RMSD of the pocket residues and the substrate RMSD between the PLACER ensemble predictions and the design models were consistently lower for the active designs. ZETA_2 exhibited the lowest average substrate RMSD, indicating highly accurate and preorganized substrate positioning according to PLACER predictions. Additionally, the predicted RMSD (uncertainty) for both the pocket and catalytic residues was exceptionally low for ZETA_2, and active designs in general had much lower uncertainty in the PLACER predictions than the “inactive” variants. The “pnear” metric distributions were also higher, on average, for the active designs, with ZETA_2 ranking among the top two or three across all these metrics. Collectively, these results suggest that PLACER confidence metrics are valuable for identifying more active designs within a scaffold family.

3. GENERAL MATERIALS

General solvents and buffers

Milli-Q water was obtained in-house using a Q-POD® Ultrapure Water Remote Dispenser from Millipore Sigma. Nuclease-free water was commercially bought from Invitrogen™. Dimethylsulfoxide (DMSO) was obtained from Sigma Aldrich.

All general buffer components (i.e., HEPES, NaCl, Tris-HCl, EDTA, imidazole, dithiothreitol) were bought from Millipore Sigma. All buffers were prepared in Milli-Q water and filtered through a 0.2 μm pore size Nalgene™ Rapid-Flow™ Sterile Disposable Filter Unit with a PES membrane. Further mentions of 'bottle filtration' will refer to this 0.2 μm pore size filter.

Materials for biology

Synthetic linear DNA fragments encoding the genes for each reverse-translated protein design with overhangs for golden gate assembly were ordered in a 96-well eblock® from IDT, shipped dry, and stored at -20 °C. Custom plasmid vectors were ordered from GenScript and stored at -20 °C. Golden gate assembly of the linear gene fragments into the custom plasmid vectors was done using the NEBridge® Golden Gate Assembly Kit (BsaI-HF® v2) from New England Biolabs. Competent BL21(DE3) *E. coli* cells for protein expression and competent NEB 5-alpha *E. coli* cells for plasmid production were commercially bought from New England Biolabs and stored at -80 °C. Super optimal broth with catabolite repression (SOC) media was purchased from New England Biolabs for recovery after heat-shock transformation.

Lysogeny broth (LB) media for *E. coli* inoculation was made from tryptone (Sigma Aldrich; 10 g/L), NaCl (Millipore Sigma; 10 g/L), and Bacto™ Yeast Extract (ThermoFisher Scientific; 5 g/L) dissolved in Milli-Q water. Terrific broth II (TB-II) media for protein production was bought as a solid powder from MP Biomedicals and dissolved at a concentration of 50 g/L in Milli-Q water. All media were prepared under flame, sterilized through bottle filtration, and immediately used or stored at 4 °C before use. TB-II and LB were either autoclaved or sterilized by bottle filtration before use. Kanamycin sulfate from *Streptomyces kanamyceticus* was bought as a powdered bioreagent from Millipore Sigma. Isopropyl β-D-1-thiogalactopyranoside (IPTG, dioxane-free) to induce protein expression was bought from Fisher Scientific. Agar plates with kanamycin were made in-house for colony isolation. Glycerol (≥99.0% grade) for glycerol stocks was bought from Sigma Aldrich.

Axygen® 96-well clear round bottom 2 mL polypropylene deep well plates for cultures are bought from Corning (P-DW-20-C-S). Breathe-Easier® breathable films for culture plates were bought from Andwin Scientific. Axygen® aluminum sealing films for general sealing purposes were bought from Corning (PCR-AS-600).

Materials for protein purification

Lysozyme, DNase I, and Polymyxin B sulfate used in *E. coli* cell lysis were bought from Sigma Aldrich (L6876, DN255G, P0972), respectively. Pierce® protease inhibitor tablets were bought from Thermo Scientific™. Filter polypropylene microplates with 96 wells (800 μL/well),

long drip spouts, and 25 μm polyethylene frit for small-scale affinity chromatography were purchased from Agilent. Econo-Pac® 25 mL gravity flow-through plastic chromatography columns for low-throughput affinity chromatography were purchased from BioRad. Econo-Column® 50 mL gravity flow-through glass chromatography columns for low-throughput affinity chromatography were purchased from BioRad. Ni-NTA Superflow® nickel resin for immobilized-metal affinity chromatography was purchased from Qiagen. Strep-Tactin® resin for affinity chromatography was purchased from IBA Lifesciences. Desthiobiotin used in protein elution was purchased from IBA Lifesciences. Strep-Tactin® regeneration buffer containing HABA (10x concentration) was also purchased from IBA Lifesciences. Amicon® 3 kDa and 10 kDa MWCO centrifugal concentrators for protein spin-concentration were purchased from Millipore Sigma. Size exclusion chromatography was performed on an ÄKTA pure™ chromatography system from Cytiva using a Superdex 200 Increase 10/300 GL column also purchased from Cytiva.

Materials for gel electrophoresis

SDS-PAGE gel electrophoresis was performed with 15- or 26-well precast AnykD gels purchased from Bio-Rad. Tris, glycine, and sodium dodecyl sulfate were obtained from Sigma Aldrich. Laemmli buffer was purchased from Bio-Rad. Precision Plus Protein™ Dual Xtra Prestained Protein Standards was purchased from Bio-Rad to serve as a ladder of protein molecular weight standards in gels. Electrophoresis was performed in either a Mini-PROTEAN Tetra Cell (15-well gel) or Criterion™ Cell (26-well gel), and powered by a PowerPac basic unit, all purchased from Bio-Rad. Gel staining was performed using an eStain L1 protein staining device purchased from GenScript with the eStain L1C Staining Kit also purchased from GenScript. Gels were imaged on a ChemiDoc XRS+ system purchased from Bio-Rad.

Materials for enzyme characterization

4-Methylumbelliferyl phenylacetate (4MU-PA, commercially known as phenyl-acetic acid 4-methyl-2-oxo-2H-chromen-7-yl ester) for the first round of activity screening was acquired from Millipore Sigma. 4MU-PA for the second round of activity screening and all kinetics and characterization experiments was custom synthesized by WuXi. 4-Methylumbelliferone (4MU) for making product concentration standards was purchased as a solid from Millipore Sigma. Full area 96-well flat bottom assay plates with a non-binding surface made from black polystyrene, used in the 1st round of screening measurements, were purchased from Corning. Half area 96-well flat bottom assay plates with a non-binding surface made from black polystyrene, used in all other experiments, were purchased from Corning. Fluorescence measurements were performed with The BioTek Synergy Neo2 Hybrid Multimode Reader RUO.

Zinc sulfate was purchased from Sigma Aldrich. 1,10-Phenanthroline for zinc chelation from the enzymes was purchased from Sigma Aldrich. Slide-A-Lyzer® 3.5 kDa MWCO dialysis cassettes were purchased from ThermoFisher Scientific. Mag-Fura-2, tetrapotassium salt was purchased from ThermoFisher Scientific for experiments determining zinc binding affinity. Quartz spectrophotometer cells with a pathlength of 10 mm were purchased from Starna Cells, Inc. UV-visible absorbance measurements were performed using a JASCO V-750 Double Beam UV-Visible Spectrophotometer. Quartz cuvettes (110 - Macro Cells) with a 1 mm pathlength for

circular dichroism were purchased from Hellma USA Inc. Circular dichroism measurements were performed using a JASCO J-1500 Circular Dichroism Spectrophotometer.

4. METHODS

4.1. Computational Design of Metallohydrolases

Design pipeline overview

We chose to design a zinc metallohydrolase for a fluorogenic ester, 4-methylumbelliferyl phenylacetate (4MU-PA), as a model reaction that would allow for sensitive screening and kinetic characterization of active enzymes and direct benchmarking with previous computationally designed zinc esterases (Fig. 1a). Rational theozymes for the rate-limiting hydroxide attack transition state in the proposed hydrolysis reaction were constructed and optimized using density functional theory. We modeled the nucleophilic attack from either side of the substrate in addition to stabilizing the oxyanion with either two external hydrogen bonds or via the zinc ion alone before creating additional low energy rotamers of the substrate to model different modes of binding (Fig. S2). Note that each theozyme contained three histidines coordinated to the zinc ion and a carboxylic acid to represent a protonated general base that deprotonated the attacking water molecule.

Protein scaffolds were generated around the designed theozymes using RFdiffusion2 with the catalytic histidine imidazoles fixed in place during generation while allowing for delta or epsilon nitrogen coordination with the zinc (Extended Data Fig. 1 and Extended Data Fig. 2a-b). The general base and oxyanion stabilizing hydrogen bonds were left to be found during later sequence generation to explore additional backbone and sequence space alike due to the flexibility of residues that can accomplish these tasks. Competent scaffolds were resampled with RFjoint2 inpainting to modify loop structures and lengths outside of the active site to generate extra diversity for each scaffold (Extended Data Fig. 2c). ProteinMPNN was used to generate global, foldable sequences for each scaffold which were verified by AlphaFold2 (Extended Data Fig. 2d). Successfully folded designs then underwent iterative active site sequence design and constrained fast relaxing (i.e., energy minimization) with LigandMPNN and Rosetta, respectively (Extended Data Fig. 2e). First, the primary sphere of residues directly interacting with the ligand (< 5Å) was redesigned before the scaffold was gently relaxed and the sidechains were repacked. Residues that were identified to make hydrogen bonds to the substrate and general bases within 3Å of the theozyme hydroxide position were kept. The process was then repeated 5 times with the allowed scaffold movement during relaxation being increasingly constrained with each cycle. Finally, the secondary sphere of residues responsible for interacting with the primary sphere residues to preorganize the active site (5Å < X < 8Å) was redesigned with LigandMPNN. The original catalytic histidines were fixed for the entirety of sequence design.

Several hundred thousand resulting design sequences across hundreds of unique scaffolds were then filtered for the presence of a general base and hydrogen bonds to the oxyanion hole for the theozymes modeled with them. Successfully filtered design sequences were then passed through AlphaFold2 to examine the predicted structure confidence and

agreement with the designed structure. Structurally verified designs were then scored by PLACER for substrate and catalytic sidechain preorganization and agreement with the designed active sites. Rosetta was used to further score the designs on additional metrics and a custom-made, weighted function combining the relevant metrics from PLACER and Rosetta was used to identify the best sequence for every remaining unique scaffold.

Theozyme construction

The initial theozymes modeling the rate-limiting nucleophilic zinc-hydroxide attack of the 4MU-PA ester were manually constructed in a 3D chemical modeling software. Each theozyme consisted of (1) a single zinc(II) ion, (2) three imidazole rings representing histidine sidechains coordinating to the zinc ion, (3) the 4MU-PA ester substrate, (4) a hydroxide ion coordinated to the zinc ion representing the deprotonated nucleophilic water molecule, and (5) an acetic acid group interacting with the hydroxyl group, representing a protonated catalytic general base (e.g., aspartate or glutamate). Furthermore, we considered two modes for oxyanion stabilization: (1) Zn(II)-O interaction, and (2) external hydrogen bond oxyanion hole (Fig. S2C-E). The Zn(II)-O oxyanion stabilization uses the zinc ion to stabilize the forming oxyanion in addition to stabilizing the nucleophilic hydroxide, requiring no extra elements to be modeled. On the contrary, the external hydrogen bond oxyanion stabilization has the oxyanion facing away from the zinc and uses two additional H-bonded methanol molecules to mimic a potential oxyanion hole. These two methanol molecules represent a generic form of an oxyanion hole and help locate the transition state geometry more akin to what it would look like in the presence of an oxyanion hole by polar groups in an enzyme active site. Each of these two classes of theozymes had the nucleophilic attack modeled from each side of the planar 4MU-PA ester bond before forming the chiral tetrahedral intermediate (i.e., *R* or *S* attack, Fig. S2A-B). Thus, four unique theozymes were modeled in total as transition states.

All transition state optimization calculations were performed using Gaussian 16 software.[48] Structural optimizations and frequency calculations were performed with the B3LYP-D3 method along with the 6-31G(d) basis set and the SDD ECP on the Zn(II) atom. D3 dispersion correction was applied using the Becke-Johnson damping function.[49] Solvent effects of water were included using the CPCM solvation model during geometry optimization. Frequency calculations were performed to confirm whether the structure is a minimum or a transition state. Intrinsic reaction coordinate (IRC) analysis was used to confirm that the obtained transition states connect the correct minima.

Conformational flexibility of the transition states was sampled based on rotatable bonds in the 4MU-PA substrate (Fig. S2C-F). The conformers were sampled as frozen coordinates with no geometry optimization, in order not to affect the transition state geometries. Energies of the conformers were evaluated using the GFN2-XTB semiempirical QM method.[50] This procedure was performed using a Python script *frozen-conf-xtb*, available on GitHub.[51] We generated 10 conformers for each transition state, all within 5 kcal/mol of each other, and with a minimum difference between conformers being at least 20 degrees for at least one rotatable bond. These conformers do not necessarily represent local minima in the conformational energy surface but rather capture structural diversity among low energy conformers. Depending on the specific

protein environment each of these conformers may be more or less preferred, and furthermore, Rosetta minimization is capable of adjusting these rotatable bonds within a small range.

We generated unique Rosetta ligand params files from each of the transition state geometries. Each params file defines the ligand, containing the 4MU-PA substrate atoms, the Zn(II) atom, and the hydroxyl group. Rosetta ligand params file was created from the produced conformers by first converting the conformer multiXYZ files to mol2 files using Open Babel, and thereafter converting the mol2 file to params using the `$ROSETTA_MAIN/source/scripts/python/public/molfile_to_params.py` script provided in Rosetta.

<u>Theozyme conformer generation & enumeration of histidine coordination modes</u>

We used the obtained transition state geometries to construct RosettaMatch constraint files that define the six degrees of freedom between the ligand and the catalytic sidechains. For example, the interaction with histidine is defined by the ZN1-NE bond, O1-ZN1-NE angle, ZN1-NE-C angle, C2-O1-ZN1-NE torsion, O1-ZN1-NE-C torsion, and ZN1-NE-C-ND torsion. Note that 'ZN1' refers to the atom name of zinc in the structure in Extended Data Fig. 1, not zinc in oxidation state 1. N_δ and N_ε coordination was sampled by defining the histidine root atom (NE or ND) using the atom type NHis, which allows either of the not-protonated nitrogen atoms in either of the tautomers to be sampled. Lastly, sampling the flip of the histidine ring was enabled by setting the rotation periodicity of the O1-ZN1-NE-C torsion to 180 degrees (Extended Data Fig. 1).

We used the constraint files to generate input motifs for RFdiffusion2. We used the Invrotzyme tool[52] to generate all samples of the different histidine coordination modes and flipped states. We only sampled allowed histidine placements, as defined by the constraint file, while ignoring all residue inverse rotamers (backbone position and sidechain χ_1 and χ_2 angles). The motifs were generated by running a command such as:

```
invrotzyme.py --cstfile theozyme.cst --params LIG.params --tip_atom …
```

<u>Backbone generation and resampling</u>

We used RFdiffusion2 to generate backbones to host each of the unique combinations of histidine placements, together with the hydrolysis reaction transition state. For simplicity, we omitted the general base at this stage and left its placement to the subsequent sequence design step. RFdiffusion2 was run by using the following command:

```
{RFdiffusion2}/run_inference.py --config-name config.yaml --config-path ./
```

Where the configuration file `config.yaml` specifies the motif parameters and other diffusion run settings. Here, `contig_atoms` specifies the fixed atoms in each input residue, with A1, B2, and C3 being the three individual histidines; PSZ is the name of the input ligand; `input_pdb` provides a path to a PDB file containing the ligand and the three histidines.

config.yaml

```yaml
defaults:
  - base

diffuser:
  T: 75

guidepost_bonds: True

inference:
  align_motif: False
  num_designs: 2
  output_prefix: /path/to/RFd2_output/zn_hydrolase_PSZ_H_H_H_1_64_1_FM_model50
  input_pdb: /path/to/theozymes/PSZ_H_H_H_1_64.pdb
  final_step: 1
  ligand: PSZ
  contig_as_guidepost: True
  remove_guideposts_from_output: True
  infer_guidepost_positions: True
  guidepost_xyz_as_design: True
  recenter_xt: True
  recenter_xt_dropt: 3

contigmap:
  contigs: ['130-275,A1-1,B2-2,C3-3']
  length: '130-275'
  contig_atoms: '{"A1":"NE2,CD2,CG,CB,ND1,CE1", "B2":"NE2,CD2,CG,CB,ND1,CE1",
                "C3":"NE2,CD2,CG,CB,ND1,CE1"}'
```

From the generated protein backbones we selected globular proteins with desirable binding pocket features based on metrics describing ligand burial, substrate exposedness, lack of clashes between the ligand and protein backbone, loop content, distance of termini from the ligand, radius of gyration, and the length of the longest helix.

We further diversified identified protein backbones with RoseTTAFold joint inpainting (RFjoint2)[53] by re-generating loop regions of the diffused backbones with variable length loop substitutions. The loop regions were selected based on geometric distance from the ligand heavy atoms and the motif residue. Sequence design was enabled on all non-motif positions during the inpainting process. For each input scaffold from RFdiffusion2, at least 4 diversified backbones were generated with this method. In summary, the purpose of RFjoint2 was to diversify and idealize small regions distant from the active site, namely loops, by sampling different sequence lengths and geometries. This method is simple, relatively inexpensive, and we found that it improves AlphaFold2 passing rates when used to complement RFdiffusion2 (Fig. S26). Note that the modified (inpainted) scaffolds from RFjoint2 were combined with the non-inpainted parent scaffolds from RFdiffusion2 for sequence design, such as to supplement them rather than replace them (e.g., 1000 parent scaffolds + 4x1000 inpainted variations = 5000 scaffolds for sequence design).

Scaffold sequence design

We reasoned that due to the possible flexibility and sequence diversity in the enzyme active site, it would be beneficial to first identify sequences that are more likely to fold correctly, and only then focus on designing the protein-ligand interactions. Therefore, we first used ProteinMPNN[34] to generate amino acid sequences optimal for each of the selected backbones (both inpainted and non-inpainted). We generated 20 sequences for each backbone while keeping the catalytic histidines fixed. Three sampling temperatures (T = 0.1, 0.3, 0.6) were used, with 10 sequences generated for T = 0.1, 5 sequences generated for T = 0.3, and 5 sequences generated for T = 0.6. This was performed while omitting the introduction of cysteine and methionine residues (--omit_AA CM) and down-weighting the introduction of alanine residues (--bias_AA A: -0.75). Generated sequences were analyzed using single-sequence AlphaFold2 prediction (monomer pTM model 4 with 6 recycles) to identify the sequences that do fold into the desired shape. Successful sequences were first selected based on AF2 metrics of plDDT ≥ 60, C_α RMSD ≤ 3.0 Å, PAE ≤ 15, pTM ≥ 0.5, and recycle convergence ≤ 1.5 Å. Note that these metrics were liberal at this step to allow a greater diversity of scaffolds to pass, especially larger ones. We reasoned that this was rational because they were going to undergo further sequence redesign and structural refinement with FastMPNNdesign, which would also reposition the catalytic residues back into their ideal geometry or otherwise get filtered out.

The sequences that passed these AF2 filters were then further processed and filtered for their catalytic histidine positional agreement with the design models. Selected AF2-predicted models were superimposed to the corresponding design models to calculate the Euclidean distances between specific pairs of atoms on matching catalytic residues (e.g., the distance between AF2 H130 C_α - design model H130 C_α). We calculated RMSDs from these measured distances for provided sets of atoms in each catalytic residue (e.g., RMSD of H130 C_α + C_β) before calculating the RMS between these RMSDs for all the catalytic residues of one type (e.g., RMS of C_α + C_β RMSDs for H118, H130, and H134). An example command calculating the backbone atom RMSD (`bb_rmsd`), backbone + C_β RMSD (`cb_bb_rmsd`), imidazole ring RMSD (`imidazole_rmsd`), and C_α + C_β RMSD (`ca_cb_rmsd`) for each matching catalytic histidine is given below:

```
sidechain_rmsd_and_info_af2_matching_res.py --scorefile scores.sc --ref_pdbs_dir
af2_inputs/ --params LIG.params --output_file updated_scores.sc
--atom_groups '{"HIS": [{"atoms": ["N", "CA", "C", "O"], "label": "bb_rmsd"},
{"atoms": ["N", "CA", "C", "O", "CB"], "label": "cb_bb_rmsd"}, {"atoms": ["CG", "CE1",
"ND1", "NE2", "CD2"], "label": "imidazole_rmsd"}, {"atoms": ["CA", "CB"], "label":
"ca_cb_rmsd"}, {"atoms": ["ND1", "NE2"], "label": "imid_sc_nitrogen_rmsd"}]}'
```

After processing with the above script, the sequences were required to pass at least one additional filter of catalytic histidine backbone (or +C_β) (`bb_rmsd` or `cb_bb_rmsd`) RMS of RMSDs ≤ 2.0 Å, catalytic histidine C_α + C_β (`ca_cb_rmsd`) ≤ 2.0 Å, catalytic histidine imidazole (`imidazole_rmsd`) RMS of RMSDs ≤ 3.0 Å, or catalytic histidine imidazole tip nitrogens (`imid_sc_nitrogen_rmsd`) RMS of RMSDs ≤ 3.0 Å.

Iterative active site sequence design with FastMPNNdesign

To optimize the amino acid sequence around the substrate and the catalytic residues we used a protocol of iteratively performing ligandMPNN[36] sequence generation, Rosetta sidechain packing, and backbone minimization, with an implementation analogous to Rosetta FastDesign[54], hereby referred to as FastMPNNdesign. The designable positions were selected based on distance from the ligand (substrate, Zn(II), OH) heavy atoms; residues with C_α atom within 8 Å from any ligand heavy atom were considered for design. Geometric constraints were applied to the Zn(II)-HIS interactions during repacking and minimization using the Rosetta AddOrRemoveMatchCsts mover. To facilitate the introduction of a general base near the Zn(II)-bound hydroxide, we identified backbone positions within 5.0 Å from the hydroxyl proton, or 7.0 Å if the distance between the proton and C_β was less than that of C_α, and applied an increased bias (+4.0) towards GLU, ASP, and HIS at those positions during ligandMPNN sequence generation.

Analysis and filtering of designed sequences

Designs were selected based on metrics describing protein-ligand contacts (Rosetta ddG, contact molecular surface, SASA, presence of a general base, H-bonds to/from acceptors/donors). An additional round of ligandMPNN sequence design was performed at the positions flanking the binding pocket, with only those in the window of 9-13 Å from the ligand enabled for redesign. For each successful design, we generated 10 additional sequences at temperatures 0.1 and 0.2, with 5 sequences coming from each temperature.

The structures of the resulting sequences were predicted using AlphaFold2 (monomer pTM model 4 with 6 recycles) and filtered with AF2 metric cutoffs set at plDDT ≥ 72.5, C_α RMSD ≤ 1.75 Å, PAE ≤ 7.25, pTM ≥ 0.725, and recycle convergence ≤ 0.75 Å. The passing structures were further processed and filtered for their catalytic histidine positional agreement with the design models using the `sidechain_rmsd_and_info_af2_matching_res.py` script as described above. After processing with the script, the sequences were required to pass at least one filter of catalytic histidine backbone (or +C_β) (`bb_rmsd` or `cb_bb_rmsd`) RMS of RMSDs ≤ 0.85 Å, catalytic histidine C_α + C_β (`ca_cb_rmsd`) ≤ 0.85 Å, catalytic histidine imidazole (`imidazole_rmsd`) RMS of RMSDs ≤ 1.25 Å, or catalytic histidine imidazole tip nitrogens (`imid_sc_nitrogen_rmsd`) RMS of RMSDs ≤ 1.25 Å.

The remaining sequences then had their apo-AF2 predicted structures relaxed with Rosetta FastRelax,[37] before calculating the spatial aggregation propensity (SAP) score, net charge, and hydrophobic exposure SASA of the relaxed structures. The relaxed sequence designs were then filtered for their 'expressibility' where they were first required to have SAP score ≤ 75 or the net charge excluding histidines ≥ 20 and hydrophobic exposure SASA ≤ 1500. The passing sequences were then clustered by their original design structure and sequences containing repeating amino acids occurring 5 times or more in a row and/or hydrophobic residues in the set AVILMFYWP occurring 7 times or more in a row were removed. For each cluster, at least one sequence was kept by adding some leniency to the aforementioned selection if all sequences were flagged by keeping the sequence with the highest absolute AF2 pTM to AF2 C_α RMSD ratio.

Enzyme sidechain ensemble analysis with PLACER

The AF2-predicted models of selected designs were superimposed to their corresponding design models, the ligand placed into the AF2 model, and the resulting holo-structures relaxed with Rosetta FastRelax. Subsequent PLACER input files were prepared either by (1) removing the zinc and hydroxide, or (2) removing the hydroxide and substrate. Thus, PLACER ensemble predictions were performed on the substrate-enzyme and zinc-enzyme complexes for each design. The generated 40 PLACER models were analyzed for the following metrics: average substrate RMSD, average histidine RMSD, average histidine pRMSD, average pocket residue RMSD and pRMSD, and worst pocket residue pRMSD. Furthermore, we analyzed the 5 highest pLDDT (1D confidence) frames for substrate RMSD and average plDDT.

Filtering and analysis after PLACER

Dataframes containing scores from FastMPNNdesign, AF2, Rosetta, and PLACER were merged based on design identity for the few thousand remaining designs spanning approximately 130 unique RFdiffusion2 scaffolds. First, one simple random design sequence for every candidate RFdiffusion2 scaffold was manually screened to exclude implausible scaffolds (e.g., completely buried substrate, buried coumarin, and exposed phenylacetate). Thereafter, we used a broad combination of redundant and nonredundant *in silico* metrics to rank and identify the best design(s) for each remaining scaffold. This approach was taken as opposed to using strict cutoffs due to the lack of known standalone predictive metrics for metallohydrolase activity; additionally, we wanted to maximize structural diversity in the final set of 96 designs as opposed to sequence sampling, hence the clustering by RFdiffusion2 scaffold. Thus, we sought to explore diverse ranges for each metric in our final order while also capturing designs that were hypothesized to be most well-balanced.

To this end, the remaining designs were ultimately clustered by the parent RFdiffusion2 scaffold. After clustering, a selection of AF2, PLACER, and Rosetta metrics hypothesized to be potentially relevant indicators of preorganization, active site configuration, and predicted structure confidence were normalized and used for a custom-weighted scoring function to score and rank designs within each cluster. Note that the metrics were split accordingly before normalization into groups where higher metric values are preferred (e.g., AF2 plDDT) or lower metric values are preferred (e.g., AF2 C_α RMSD). The chosen weights were an arbitrary initial guess that were meant to roughly balance the cumulative influence of grouped metrics from AF2, PLACER, and Rosetta. The ‘composite score’ calculated as the output of the scoring function was simply the weighted sum of all normalized metrics preferring lower values, plus the weighted sum of inverse-normalized metrics preferring higher values. Thus, the lowest composite score corresponded to the best-ranked design.

By this logic, the 3 designs with the lowest composite scores were selected from each RFdiffusion2 scaffold cluster (for clusters with 3 or fewer candidate sequences, all were retained), applying the metrics and weights shown below. All other designs were filtered out. Following this filtering step there were only 3 or fewer sequences left for each candidate scaffold, leaving approximately 260 sequences spanning 119 RFdiffusion2 scaffolds. The

optimal sequence for each RFdiffusion2 scaffold cluster was chosen as the one with the lowest AF2 C_α RMSD from its design. All other sequences were filtered out, leaving a single sequence per scaffold. Only scaffolds with top-ranked sequences among the 96 lowest AF2 C_α RMSD values were subsequently ordered.

```
## Columns to Normalize and Invert (i.e., higher value = better) ##
columns_to_normalize_and_invert = [
'pTMscore_af2_out','zinc_plddt_pde_top5','zinc_plddt_top5','zinc_plddt_pde_pnear',
'zinc_plddt_pnear','subst_plddt_top5','subst_plddt_pde_top5',
'subst_frac_good_kabsch', 'subst_plddt_pnear', 'subst_plddt_pde_pnear',
'O2_hbond_fastmpnn','total_hbonds', 'O2_hbond', 'O3_hbond', 'O5_hbond',
'electrostatic_complementarity_p', 'electrostatic_complementarity_s',
'hbonds_to_metal_binding_residues']
...
normalized_df[col] = 1 - (input_df[col] - min_val) / (max_val - min_val)

## Columns to Normalize without Inversion (i.e., lower value = better) ##
columns_to_normalize = [
'rmsd_to_input_af2_out', 'zinc_rmsd_ligand', 'zinc_rmsd_std_cat_rms',
'subst_rmsd_ligand', 'subst_u_cat_rms', 'zinc_rmsd_std_ligand',
'zinc_rmsd_pde_top5', 'zinc_rmsd_top5', 'zinc_rmsd_cat_rms', 'zinc_u_cat_rms',
'subst_rmsd_std_ligand', 'subst_rmsd_top5', 'subst_rmsd_pde_top5',
'subst_rmsd_cat_rms', 'subst_rmsd_std_cat_rms', 'SAP_score', 'violations',
'all_cst_fastmpnn', 'cat_HIS_ca_cb_ONLY_rms_of_rmsds_af2_out',
'cat_HIS_imidazole_rms_of_rmsds_af2_out',
'cat_HIS_imidazole_tip_Ns_rms_of_rmsds_af2_out', 'ligand_rmsd_post_holo_relax',
'Ca_rmsd_after_fastrelax', 'longest_cont_apolar_seg',
'hydrophobic_exposure_sasa_in_design', 'fa_elec_fastmpnn', 'fa_intra_elec_fastmpnn',
'hbond_sc_fastmpnn', 'total_score_fastmpnn']
...
normalized_df[col] = (input_df[col] - min_val) / (max_val - min_val)
```

```
## Composite Score Calculation ##
# Each cumulative family of metrics (i.e., Rosetta, PLACER, and AF2) is
# approximately equal.

weights = {
    ## AF2 Metrics ##
    'rmsd_to_input_af2_out': 0.125,
    'pTMscore_af2_out': 0.125,
    'cat_HIS_ca_cb_ONLY_rms_of_rmsds_af2_out': 0.0625,
    'cat_HIS_imidazole_rms_of_rmsds_af2_out': 0.0625,
    ## Zinc-Enzyme PLACER Ensemble Metrics ##
    'zinc_rmsd_ligand': 0.05,
    'zinc_rmsd_std_ligand': 0.01,
    'zinc_plddt_pde_top5': 0.01,
    'zinc_rmsd_pde_top5': 0.01,
    'zinc_rmsd_top5': 0.01,
    'zinc_plddt_top5': 0.01,
    'zinc_rmsd_cat_rms': 0.01,
    'zinc_rmsd_std_cat_rms': 0.01,
    'zinc_u_cat_rms': 0.01,
    'zinc_plddt_pde_pnear': 0.01,
    'zinc_plddt_pnear': 0.01,
    ## Substrate-Enzyme PLACER Ensemble Metrics ##
    'subst_rmsd_ligand': 0.05,
    'subst_rmsd_std_ligand': 0.01,
    'subst_rmsd_top5': 0.01,
    'subst_plddt_top5': 0.01,
    'subst_rmsd_pde_top5': 0.01,
```

```
    'subst_plddt_pde_top5': 0.01,
    'subst_rmsd_cat_rms': 0.01,
    'subst_rmsd_std_cat_rms': 0.01,
    'subst_frac_good_kabsch': 0.01,
    'subst_plddt_pnear': 0.01,
    'subst_plddt_pde_pnear': 0.01,
    ## Rosetta Metrics Dealing with Expressibility & Active Site Features ##
    'O2_hbond_fastmpnn': 0.05,
    'SAP_score': 0.05,
    'violations': 0.05,
    'all_cst_fastmpnn': 0.05,
    'longest_cont_apolar_seg': 0.025,
    'hydrophobic_exposure_sasa_in_design': 0.025,
    ## Rosetta Metrics from FastMPNNdesign ##
    'ligand_rmsd_post_holo_relax': 0.05,
    'Ca_rmsd_after_fastrelax': 0.05,
    'O2_hbond': 0.025,
    'O3_hbond': 0.025,
    'O5_hbond': 0.015,
    'electrostatic_complementarity_p': 0.015,
    'electrostatic_complementarity_s': 0.015,
    'hbonds_to_metal_binding_residues': 0.015,
    'fa_elec_fastmpnn': 0.01,
    'fa_intra_elec_fastmpnn': 0.01,
    'hbond_sc_fastmpnn': 0.01,
    'total_score_fastmpnn': 0.01
}

## Calculate the Composite Score ##
normalized_df['composite_score'] = sum(normalized_df[col] * weight for col, weight
in weights.items())
...
### FILTERING TO 3 FILES PER GROUP IF MORE THAN 3 ###
filtered_groups = []
for group, data in pre_filtered_df.groupby('flow_matching_group'):
    if len(data) > 3:
        data = data.nsmallest(3, 'composite_score')
```

Structural similarity search of the PDB and AFDB (1st Design Campaign)

To evaluate the structural novelty of the A1 (ZETA_1) design, we employed FoldSeek,[55] a tool that enables rapid and accurate structure searches. We compared the A1 design against the PDB100, a clustered subset of the Protein Data Bank containing entries deposited before January 2024, and the AFDB50,[56] a clustered database with 50% sequence identity (version 4, encompassing 54 million structures). We reported the TM-score for the top match (Extended Data Fig. 4a-b). The top hit against PDB100 was 8GYN, which corresponds to the Tumor Necrosis Factor Alpha-Induced Protein 8-Like Protein 1 in zebrafish. In contrast, the top hit against AFDB50 was A0A176QCW7, an unknown protein predicted to function as a biotin transporter.

4.2. Changes in the Second Design Campaign of Metallohydrolases

Note: This is referred to as the “2nd/second design campaign” and “round 2” interchangeably in the main text and supplemental information.

Observations and trends from the first design campaign

We made several observations from the design structures of the hits from the first design campaign which informed our refined strategy for the second design campaign. We describe most of these in section 2.1, but we note the especially relevant observations again below:

(1) All of the hits except for F7 came from the class of DFT theozymes using Zn(II) to stabilize the oxyanion, rather than 2 protein hydrogen bonds. Perhaps this was because it was difficult to precisely position 2 protein hydrogen bonds, either from sidechains and/or backbones, to the substrate oxyanion hole during sequence design after the scaffold had already been generated. Even in the case of F7, the design model only predicts 1 hydrogen bond from a serine residue to the oxyanion, not 2 as intended by the DFT theozyme. We allowed these cases, like F7, to pass since it was extremely rare to get a well-behaved design with 2 hydrogen bonds in place stabilizing the oxyanion.

(2) Excluding F7, the 4 hits using Zn(II) to stabilize the oxyanion were derived from the DFT theozyme of the *S*- enantiomer of the hydroxide attack. That is, the design models of A1 (ZETA_1), A8, B9, and C4 all contain the *S*- enantiomer of the hydroxide attack. F7 models the *R*- enantiomer of the hydroxide attack, but note that it was designed for a different mode of oxyanion stabilization.

(3) The design models of every hit coordinate the Zn(II) with the N_ε atom of the His residues, except B9 which has 2/3 His residues coordinating Zn(II) with their N_ε atom and the other His residue coordinating with its N_δ atom. The all-N_ε atom coordination and 2/3 N_ε atom coordination geometries for zinc hydrolases with three Zn(II)-coordinating His residues are very common (e.g., PDB: 1AST (pdb_00001ast); 2VVB (pdb_00002vvb)).

(4) The scaffold for A1 (ZETA_1) was generated around its theozyme with the exact conformation from DFT (i.e., NOT from one of the extra conformers rapidly generated from the original DFT model using GFN2-XTB semiempirical QM). However, during FastMPNNdesign, Rosetta chose a different conformer with the 4MU flipped approximately 180 degrees. Nonetheless, this is almost an equivalent geometry in terms of the interactions that were optimized during DFT such as pi-stacking with the His residues. The models of the other hits also had the substrate approximately in the geometry that was optimized out of DFT, rather than one of the more drastically-different conformers. We interpreted this to suggest that designing around the exact DFT theozyme, instead of a different conformer of the DFT theozyme, is optimal and that any conformational sampling should be done at the DFT step (i.e., creating different conformer input models for DFT). This better ensures that the energy is minimized for each theozyme complex of the different conformers (i.e., interactions are optimized).

(5) The position of the proposed general base in the design models of the hits was suboptimal in comparison to how it was positioned during DFT or in comparison to native enzymes like astacin, thermolysin, or carboxypeptidase. Additionally, we experimentally confirmed that the general base was dysfunctional in A1 (ZETA_1) after knocking it out to alanine and measuring the kinetics, which were not significantly different from the wild-type (Fig. 3i). Although we modeled the general base during DFT, we did not include it in the motif during RFdiffusion2, reasoning that Glu/Asp/His could accomplish this function and thus it could be found during sequence design. This suggested to us that this strategy was not ideal and that the position of the general base was potentially more delicate than expected.

(6) The sequence lengths of the hits were all relatively short compared to what was sampled during design. We generated scaffolds that were between 130 amino acids and 275 amino acids in length, but 4/5 of the hits were designed to be <175 amino acids in length (A8 was only 206 amino acids). Notably, A1 (ZETA_1) was the shortest enzyme of the hits, only 148 residues in sequence length. Many of the methods may perform better with smaller proteins which could have biased the set of proteins that survived the design pipeline to be smaller on average. Nonetheless, the ideal catalyst should generally be as small as possible to optimize material efficiency, assuming the desired catalytic properties of the catalyst stay the same.

Theozyme preparation for the second design campaign using only new DFT models

We used the observations stated above to narrow our theozyme search in a second round of DFT and enhance our design pipeline for the second design campaign. Accordingly, we restricted our new DFT transition state search to: (1) theozymes in which the oxyanion is stabilized by Zn(II); (2) the *S*-enantiomer of the hydroxide attack; and (3) histidine sidechains explicitly coordinating Zn(II) through their N_ε atom, thus adding C_β to each histidine in the DFT input models (Fig. 4a). We built an DFT input theozyme of this description using a 3D chemical modeling software, with the A1 (ZETA_1) design model active site as reference, and performed transition state optimization as described in section 4.1.

Additionally, we wanted to perform any conformational sampling at the DFT step, where unique conformers could be optimized and verified to be a valid transition state, especially since we were working with only one specific theozyme. We modeled every combination of 180° flips for (1) each histidine sidechain, (2) the 4MU leaving group in the substrate, and (3) the carboxylate general base (see annotated arrows on Fig. 4a) and performed transition state optimization for each model. In total, 32/32 optimized transition state geometries were successfully obtained from DFT for the combinations of conformational samples. These optimized transition state geometries from DFT were directly turned into pdb files. Furthermore, the general base was represented as either glutamate or aspartate. This amounted to 64 pdb theozymes which served as the inputs to RFdiffusion2.

Generating scaffolds with an updated version of RFdiffusion2

The updated version of RFdiffusion2 can optionally intake point coordinates which direct the model on where it should aim to generate the scaffold's center of mass. These coordinates are specified as an 'ORI token' in the theozyme input pdbs, a unique line with a format such as:

```
HETATM   80  ORI ORI X  10       X_CORD  Y_CORD  Z_CORD  1.00  1.00           X
```

Where `X_CORD`, `Y_CORD`, and `Z_CORD` specify the point coordinate (x,y,z) of the ORI token directing RFdiffusion2 on where the generated protein center of mass should be approximately located. The advantage of this strategy is that a user can place ORI tokens around parts of the substrate or theozyme which they believe should be more surrounded by protein, effectively controlling which part of the theozyme should be more buried (and conversely, which part of the theozyme should be more solvent-exposed). We suspected that it might be important for the 4MU leaving group to be more solvent exposed, suggesting that the ORI token be placed around the PA part of the substrate, but we decided not to fully commit to that strategy in favor of one that would yield a greater diversity of scaffold designs.

To this end, we placed a central ORI token near the ester of the substrate and used a simple script to generate a cloud of 71 additional ORI token coordinates in a 4.5 Å radius around the central point, totaling 72 ORI tokens. The ORI tokens were approximately homogeneously distributed throughout the sphere, not just on the surface. The result was a wide diversity of ORI tokens which would specify many points for generating protein centers of mass with respect to the theozyme, resulting in many different binding orientations. This ended up being the case for ZETA_2 and ZETA_3, which had ORI tokens on opposite sides of the substrate (Extended Data Fig. 7).

We performed small-scale RFdiffusion2 testing of these 72 ORI token positions with a single theozyme and manually checked the outputs. We decided to remove 8 of the ORI tokens, although in retrospect we were not convinced that this step was worth the computational effort. This left us with a final set of 64 ORI tokens. This set of filtered ORI tokens was inserted the 64 theozyme pdbs; each theozyme pdb file was replicated for every ORI token, where a single ORI token was inserted into each copy, generating the complete set of combinations between the 64 theozyme pdbs and all ORI tokens. These served as the final inputs to RFdiffusion2. Specifically, there were 64 theozymes (2^5 flip conformer combinations x 2 possible general bases (Glu/Asp)) x 64 filtered ORI tokens = 4096 unique combinations as inputs to RFdiffusion2.

RFdiffusion2 was set up and executed as described in section 4.1, with some minor changes to the yaml file (example shown below). Notably, we specified the functional group for the general base as part of the motif in this second design campaign, instead of leaving the general base to be found during sequence design. We hypothesized that this would better guarantee its precise positioning since it was being fixed in its DFT geometry from the start of the pipeline. Additionally, we reduced the possible scaffold length range from 130–275 to 110–190 amino acids. Specifically, for each of the 4096 RFdiffusion2 inputs, commands were created such that exactly 2 structures would be produced: 1 structure with a length between 110-150 amino acids and 1 structure with a length between 150-190 amino acids. Thus, a total of ~8000 unique scaffolds were generated.

config.yaml (2nd Design Campaign with Updated RFdiffusion2)


```yaml
defaults:
  - aa_v2

diffuser:
  T: 100
  aa_decode_steps: 40

guidepost_bonds: True

inference:
  align_motif: False
  num_designs: 1
  output_prefix: /path/to/RFd2_output/theozyme_X_GLU_lig_SZA_ORI_Y__model_RFD140
  input_pdb: /path/to/theozymes/theozyme_X_GLU_lig_SZA_ORI_Y.pdb
  final_step: 1
  ligand: SZA
  contig_as_guidepost: True
  idealize_sidechain_outputs: True
  remove_guideposts_from_output: True
  infer_guidepost_positions: True
  guidepost_xyz_as_design: True
  center_motif: True
  recenter_xt: False
  update_seq_t: False
  write_trb: True

contigmap:
  contigs: ['110-150,A1-1,B2-2,C3-3,D4-4']
  length: '110-150'
  contig_atoms: '{"A1":"NE2,CD2,CG,CB,ND1,CE1", "B2":"NE2,CD2,CG,CB,ND1,CE1",
                 "C3":"NE2,CD2,CG,CB,ND1,CE1", "D4":"OE1,OE2,CG,CD"}'
```


Filtering with Chai-1 predictions of the protein-Zn(II)-substrate phosphoanalog

We had the advantage of incorporating Chai-1 as a computational filter in our second design campaign, encouraged by the remarkable agreement between the A1 (ZETA_1) design model and the predicted Chai-1 ensembles, which also predicted the D67 and H130 Zn(II) binding competition. However, we had concerns about using Chai-1 to predict the protein-Zn(II)-substrate ensemble and filtering on this basis: this strategy could enrich design selection for tight substrate binders, which are not necessarily good enzymes. Enzymes perform catalysis through binding their reaction transition state(s) tighter than the substrate or product(s).[57] Therefore, it would be ideal to use Chai-1 to predict ensembles of the protein-Zn(II)- and something that resembles the transition state of the reaction, then filtering on this basis. However, we were also concerned about using Chai-1 to predict the protein-Zn(II)-tetrahedral carbon intermediate ensemble since it will have never seen an ester tetrahedral during training because it is an unstable, brief intermediate and therefore non-crystallizable.[58]

Thus, we decided that the ideal Chai-1 ligand prediction would (1) be nearly identical to our substrate, (2) resemble the transition state geometry and charge, and (3) be well-represented in its training data, especially in the context of Zn(II). Indeed, crystallographers and enzymologists previously solved problems (1) and (2) when trying to capture

metallohydrolase structures in their catalytic geometry[24] and measure the binding affinity of the transition state: they used phosphate transition state analogs (phosphoanalogs) of the ester or amide substrate, such as phosphinates.[59,60] Phosphates are tetrahedral, and phosphodiesters specifically, typically have a net negative charge of -1 around neutral pH, closely mimicking the geometry and charge of the transition state of a hydrolysis reaction in a metallohydrolase active site. Furthermore, there are many crystal structures of zinc hydrolases bound to these transition state analogs (e.g., PDB: 1TLP (pdb_00001tlp), 1QJI (pdb_00001qji), 5OD1 (pdb_00005od1), 2TMN (pdb_00002tmn)) because of their resemblance of the transition state, making them potent inhibitors,[59,60] solving problem (3). With this information, we made Chai-1 predictions of the protein-Zn(II)-phosphoanalog of 4MU-PA (i.e., replacing the ester carbon with phosphorus) and filtered the designs from these predictions. An example of a Chai-1 command used is shown below:

```
path/to/chai/run_chai.sh --name designX_ZN_TSA__5_pred --protein "['DESIGNSEQ']"
    --ligand "['[Zn+2]', 'Cc2cc(=O)oc3cc(OP(=O)([O-])Cc1ccccc1)ccc23']"
  --output_dir /path/to/out/chai_output_dir --export_arrays --export_mode json
```

Predictions and filtering was performed in two broad iterations: (1) Chai-1 predictions of the protein-Zn(II)-phosphoanalog of 4MU-PA without additional N-terminal or C-terminal tags followed by filtering, and (2) Chai-1 predictions of the protein-Zn(II)-phosphoanalog of 4MU-PA with an added MSG N-terminal sequence and added GSAWSHPQFEK C-terminal sequence, representing what the designs would be expressed as, followed by filtering. The second step was performed due to concerns about the N- or C-terminus being too close to the active site in some designs. After each step of predictions, filtering was also done in two iterations: (1) general metric cutoffs that every design had to pass with no exceptions and (2) more extreme metric cutoffs that every design had to pass unless every design from a unique scaffold family was filtered out, in which case only the best designs from that family would be kept (2-3 maximum).

Although the exact filtering cutoffs differed for the steps described above, we always filtered on the same metrics. Described herein are the metrics and the specific cutoffs for the final step of filtering. First, we filtered for the positional agreement between the Chai-1 predictions and the design model: C_α RMSD ≤ 1.25 Å, catalytic residue RMSD ≤ 1.5 Å, substrate RMSD ≤ 4.0 Å, substrate “core” atoms (ester + hydroxide) RMSD ≤ 2.0 Å, and Zn(II) RMSD ≤ 1.25 Å. Note that Chai-1 predicts 5 ensembles by default; we filtered such that 4/5 (80%) of the Chai-1 predictions had to pass all of these filters. Additionally, we filtered on the Chai-1 confidence metrics for its predictions: average protein atom (chainA) pLDDT ≥ 80, average substrate atom (chainB) pLDDT ≥ 85, Zn(II) (chainC) pLDDT ≥ 85, catalytic residue atom pLDDT ≥ 85, substrate “core” atoms (ester + hydroxide) pLDDT ≥ 80, ipTM ≥ 0.7, pTM ≥ 0.875, protein-substrate average PAE (chainA_chainB_pae_avg) ≤ 6, protein-Zn(II) average PAE (chainA_chainC_pae_avg) ≤ 6, and finally substrate-Zn(II) average PAE (chainB_chainC_pae_avg) ≤ 6. Again, note that Chai-1 predicts 5 ensembles by default and we required 4/5 (80%) of the Chai-1 predictions had to pass all of these filters. At this step, if all designs were filtered out for a scaffold family, we kept the designs with the lowest substrate

RMSD and catalytic residue RMSD in that scaffold family (2 maximum). This left us with designs spanning 37 unique scaffolds.

Structural similarity search of the PDB and AFDB (2nd Design Campaign)

To evaluate the structural novelty of the ZETA_2-4 designs, we employed FoldSeek,[55] as was done in the first design campaign for ZETA_1. Likewise, we compared these designs against the PDB100, a clustered subset of the Protein Data Bank containing entries deposited before January 2024, and the AFDB50,[56] a clustered database with 50% sequence identity (version 4, encompassing 54 million structures). We reported the TM-score for the top match and displayed the structural alignment in Extended Data Fig. 4c-h.

The top hit for ZETA_2 against the PDB100 was 2V6Y, which corresponds to the structure of the microtubule interacting and transport domain from a *S. solfataricus* Vps4-like ATPase, a protein that plays a key role in membrane remodeling processes, including vesicle trafficking and membrane scission, through the disassembly of ESCRT-III complexes. The top hit for ZETA_2 against the AFDB50 was A0A558CL52, which corresponds to an uncharacterized protein in the organism *Amycolatopsis rhizosphaerae*.

The top hit for ZETA_3 against the PDB100 was 5FUC, which corresponds to the structure of a junctional epitope antibody (VHH6) that simultaneously binds human interleukin-6 (IL-6) and its receptor subunit gp80, stabilizing their complex and modulating inflammatory signaling pathways. The top hit for ZETA_3 against the AFDB50 was A0A1B7XEX0, which corresponds to a GntR C-terminal domain-containing protein in the organism *Halodesulfovibrio spirochaetisodalis*.

The top hit for ZETA_4 against the PDB100 was 6Y06, which corresponds to the structure of Moevan, a de novo designed granulopoietic protein engineered through topological rescaffolding. Moevan was developed to bind the granulocyte colony-stimulating factor (G-CSF) receptor and promote granulocyte differentiation, despite lacking sequence similarity to native cytokines. The top hit for ZETA_4 against the AFDB50 was A0A353XWA2, which corresponds to a major facilitator superfamily transporter protein from a *Gammaproteobacteria* bacterium.

4.3. Experimental Biology & Biochemistry

Cloning and transformation

Genes encoding the designed proteins with overhangs for Golden Gate assembly were ordered from IDT as dry eBlocks™. The DNA sequences were codon-optimized for increased *E. coli* expression, and ordered as follows:

```
(5') ATACTACGGTCTCAAGGA-<design>-GGTTCCCGAGACCGTAATGC (3')
```

The DNA fragments were cloned into a custom vector (pDTstrep1; 5684 base pairs) encoding a kanamycin-resistance gene for positive selection, a T7 promoter, and a C-terminal Strep-tag®, following a published protocol.[61] The final expressed proteins were obtained as `MSG-design-GSA-WSHPQFEK`, where WSHPQFEK is the C-terminal Strep-tag® sequence for Strep-Tactin® affinity chromatography purification.

Before golden gate assembly, 20 µL of nuclease-free water was added to each well of the dry eBlock before the plate was covered with an aluminum seal, vortexed, and centrifuged (1000g x 30s) to suspend the linear DNA fragments at a concentration of 10 ng/µL (approximately 23 nM for the largest fragments of 700 bp). Golden gate assembly was performed using the NEBridge® Golden Gate Assembly Kit (BsaI-HF® v2) containing (1) a solution of BsaI type IIS restriction enzyme and T4 DNA ligase, and (2) a T4 ligase buffer with 1 mM ATP. The reactions were set up at a 5 µL scale such that DNA fragment concentration was at least 8.8 nM for the largest DNA fragments (700 bp) and the cloning vector concentration was 4.4 nM. First, a master mix of the cloning reaction components (GG assembly kit, T4 buffer, cloning vector, and ddH_2O) was prepared. For each 5 µL reaction, the master mix consisted of 1.8 µL ddH2O, 0.5 µL T4 buffer, 0.25 µL GG assembly kit, and 0.54 µL cloning vector (from 41 nM stock), mixed in the given order. The master mix was prepared for 96 reactions in a cooled microcentrifuge tube and then spread out in 3.1 µL aliquots to a cooled PCR plate. Lastly, 1.9 µL solution of the DNA fragment (~23 nM stock in case of 700 bp fragment) was added to each well and carefully mixed with the pipette tip. The plate was sealed, and incubated in a thermocycler (37 °C) for 20 minutes, followed by a 5 minute incubation at 60 °C to heat-inactivate the enzymes. The resulting plasmid products of Golden Gate assembly were then used for transformation; excess reaction mixture was stored at -20 °C for future use.

Bacterial transformation was performed over ice using competent BL21(DE3) *E. coli* cells. A new PCR plate was set into a cooled metal block on ice and 2.0 µL of the Golden Gate plasmid products from the previous steps were transferred into their respective wells with a multichannel pipette. This PCR plate was briefly sealed and centrifuged (1000g x 30s) to get the plasmids at the bottom of each well. Next, tubes of competent *E. coli* stored at -80 °C were allowed to thaw and swiftly added in 7 µL aliquots to each well with a repeater pipette. The PCR plate was rapidly centrifuged (100g x 5-10s) to ensure sufficient mixing of the cells and plasmids at the bottom of each well and the plate was returned to the metal cooling block on ice for 30 minutes. Thereafter, the plate was transferred to a 42 °C metal block for 17~20s to heat shock the *E. coli* and returned to ice for 2 minutes. 100 µL of pre-warmed SOC recovery media was

added into each well of the PCR plate and a breathable film was used to cover the plate. The plate was incubated in a microplate shaker (37 °C, 1200 rpm) for 1 hour. 900 µL aliquots of sterile LB media with kanamycin (50 µg/mL) were added to each well in a 2 mL deep round-well incubation plate under flame, followed by 100 µL of the transformed *E. coli* in SOC media. The deep round-well plate was covered with a breathable film and incubated in a microplate shaker (37 °C, 1200 rpm) for 16-18 hours.

Successfully cloned designs had opaque wells while unsuccessfully cloned designs had transparent wells the following day. Glycerol stocks were made for every clone by mixing 125 µL of 50% v/v glycerol in Milli-Q water with 125 µL of the overnight culture in a new PCR plate. The PCR plate was covered with an aluminum seal and stored at -80 °C. Note that the obtained clones are not sequence-verified at this stage and could be polyclonal or even wrong due to mistakes during DNA synthesis. After preparing the glycerol stocks, the remaining cultures were used for expression, as described below.

<u>Small-scale protein expression with IPTG</u>

Proteins were expressed at a 4x1 mL culture scale in sterile terrific broth II (TB-II) media supplemented with 50 µg/mL of kanamycin. Four 2 mL deep round-well plates were loaded with 1 mL of the TB-II+kanamycin media under flame. 150 µL of the overnight cultures in LB media were transferred into each respective well for each of the four plates. These plates were covered with breathable film and incubated in a microplate shaker (37 °C, 1200 rpm) for 90 minutes to promote sufficient cell growth. Thereafter, 11.5 µL of 100 mM IPTG (isopropyl β-D-1-thiogalactopyranoside) was added into every well of the four plates (1 mM final [IPTG]) under flame to induce expression. The plates were resealed with the breathable films and incubated in the microplate shaker (37 °C, 1200 rpm) for 2 hours before being transferred to a room temperature microplate shaker (25 °C, 1200 rpm) to incubate for an additional 20-24 hours. The following day, the plates were centrifuged (4,198 g, 8 min, 25 °C) to collect the cell pellets. The resulting plates containing the cell pellets were covered with an aluminum seal and stored at -80 °C until purification.

<u>Protein purification for screening</u>

The frozen cell pellets were thawed and lysed, and the expressed protein was purified by affinity chromatography with Strep-Tactin® resin following an adapted published procedure.[62] First, three buffers were prepared with Milli-Q water as follows: **W1 buffer** (100 mM Tris-HCl, 150 mM NaCl, 1 mM EDTA, pH 8.0), **W2 buffer** (100 mM Tris-HCl, 150 mM NaCl, pH 8.0), and **E buffer** (40 mM HEPES, 50 mM NaCl, 2.5 mM desthiobiotin, pH 8.0). Additionally, a **lysis buffer** was made from the W2 buffer where it was supplemented with 1 mg/mL lysozyme, 0.5 mg/mL polymyxin B sulfate, 10 µg/mL of DNAse I, and 1 Pierce® protease inhibitor tablet per 50 mL of W2 buffer. All buffers were stirred until the reagents were completely dissolved, and sterile-filtered through a 0.2 µm pore size bottle-filter.

Four plates of cell pellets (i.e., 4 mL of culture for each design) were simultaneously thawed and 600 µL of **lysis buffer** was added to each well in one plate and transferred to the other three plates to combine all of the same-design pelleted cultures in the fourth plate (e.g.,

A1 plate 1 → A1 plate 2 → A1 plate 3 → A1 plate 4). Cells were suspended by repeatedly pipetting the suspension in and out of the tip. The final plate containing the combined cultures in lysis buffer was covered with an aluminum seal and placed in a shaking microplate incubator at 37 °C, 1200 rpm for 60 minutes and then transferred to a 60 °C metal bead bath for 20 minutes. The plate was then centrifuged (4,198 g, x 30 minutes; 14 °C) to pellet the insoluble lysate aggregates before column purification.

Affinity chromatography was performed by adding 200 µL of a 50% Strep-Tactin® resin suspension to each well in a 96-well 25 µm fritted plate such that 1 CV = 100 µL of resin. The resin was equilibrated with 500 µL of **W1** buffer 3 times (3 x 5 CV of W1 buffer) while using a vacuum manifold to pull the liquid through the plate. The bottom spouts of the fritted plate were sealed with parafilm, and 600 µL of the soluble lysate supernatant was gently added to each well in the fritted plate. The fritted plate was covered with an aluminum seal and inverted several times to resuspend the resin. The resuspended resin was allowed to settle at room temperature for 15 minutes before the parafilm and aluminum seal were removed. The protein-loaded resin was washed 2 times with 500 µL of **W1** buffer (2 x 5 CV of W1 buffer) and 1 time with 500 µL of EDTA-free **W2** buffer (1 x 5 CV of W2 buffer) while using a vacuum manifold to pull the liquid through. Finally, the bound protein in each column was eluted into a 2 mL deep 96 well plate by adding 200-300 µL of **E** buffer (1 x 2-3 CV of E buffer) and eluting by centrifugation (1,500 g, 2 min). The eluted protein plate was covered with an aluminum seal and stored at 4 °C until further use. The eluted protein solutions were analyzed by SDS-PAGE to determine the presence of soluble protein. The results of the SDS-PAGE analysis are presented in Fig. S3. We found that 89/96 designs yielded soluble protein band(s). For some proteins, multiple bands were present, possibly because of the presence of multiple species in the obtained eBlocks™ DNA fragments.

The used Strep-Tactin® resin was recovered by first washing each well in the plate with 100-200 µL of E buffer (1 x 1-2 CV of E buffer), 500 µL of W1 buffer (1 x 5 CV of W1 buffer), and 300 µL of W2 buffer (1 x 3 CV of W2 buffer). Finally, the resin was regenerated by adding 3x500 µL of regeneration buffer (purchased as 10x concentrated and diluted in Milli-Q water) (3 x 15 CV of regeneration buffer) until a dark red color was homogeneous through the resin. Note that the resin was resuspended several times via pipetting during the cleaning and regeneration steps to remove aggregates and ensure homogeneity. The regenerated resin wells in the fritted plate were stored under 40-60% v/v ethanol and water to prevent bacterial growth; the plate was covered with an aluminum seal on the top and parafilm on the bottom spouts before being stored at 4 °C until further use.

Screening fluorescence assay (1st round of 96 designs)

The activity screening assay was performed in a 96-well (full-area) fluorescence plate to screen for the hydrolysis of the fluorogenic ester 4MU-PA (Sigma), monitored by a plate-reader using an excitation/emission wavelength of 365 nm and 445 nm, respectively. Briefly, each reaction was run at 30 °C using 6 µL of eluted enzyme containing 200 µM of supplemented zinc sulfate and 54 µL of a substrate solution composed of 10 µM of 4MU-PA in reaction buffer (40 mM HEPES, 50 mM NaCl, pH 8.0) with 5.0% v/v DMSO. Thus, the final reaction in each well

contained the eluted enzyme (1/10th the eluted concentration), 20 μM of zinc sulfate, 9 μM of 4MU-PA, and approximately 4.5% v/v DMSO in the reaction buffer.

In a new PCR plate, 48 μL of eluted protein and 2 μL of the 5 mM zinc sulfate solution were combined (final [Zn(II)] = 200 μM). The plate was covered with an aluminum seal, briefly centrifuged (1,000g, 15 s), and incubated at 37 °C for 15 minutes. Afterward, 6 μL of the enzyme-zinc solution from each well was transferred into its corresponding well in a 96-well (full area) fluorescence reaction plate. A stock solution of 100 mM 4MU-PA (Sigma) in DMSO was prepared and used to make a 10 μM working solution of the substrate in reaction buffer with 5.0% DMSO v/v. 54 μL of the substrate working solution was quickly added to every well in the reaction plate using a multichannel pipette. The reaction plate was placed into the plate reader, shaken for 3s at 282 cpm, and fluorescent progress curves monitored for one hour at 30 °C. Note that the enzyme concentration was not standardized in the screening assay, as this was only used to identify hits to characterize in more detail.

Screening fluorescence assay (2nd round of 96 designs)

The activity screening assay was performed in a 96-well (half-area) fluorescence plate to screen for the hydrolysis of the fluorogenic ester 4MU-PA (WuXi), monitored by a plate-reader using an excitation/emission wavelength of 365 nm and 445 nm, respectively. Briefly, each reaction was run at 25 °C using 10 μL of eluted enzyme containing 100 μM of supplemented zinc sulfate and 90 μL of a substrate solution composed of 111 μM of 4MU-PA in reaction buffer (40 mM HEPES, 50 mM NaCl, pH 8.0) with 5.55% v/v DMSO. Thus, the final reaction in each well contained the eluted enzyme (1/10th the eluted concentration), 20 μM of zinc sulfate, 100 μM of 4MU-PA, and approximately 5% v/v DMSO in the reaction buffer.

In a new PCR plate, 49 μL of eluted protein and 1 μL of the 5 mM zinc sulfate solution were combined (final [Zn(II)] = 100 μM). The plate was covered with an aluminum seal, briefly centrifuged (1,000g, 15 s), and incubated at room temperature for 15 minutes. Afterward, 10 μL of the enzyme-zinc solution from each well was transferred into its corresponding well in a 96-well (half area) fluorescence reaction plate. A stock solution of 100 mM 4MU-PA in DMSO was prepared and used to make a 111 μM working solution of the substrate in reaction buffer with 5.55% DMSO v/v. 90 μL of the substrate working solution was quickly added to every well in the reaction plate using a multichannel pipette. The reaction plate was placed into the plate reader, shaken for 3s at 282 cpm, and fluorescent progress curves monitored for one hour at 25 °C. Note that the enzyme concentration was not standardized in the screening assay, as this was only used to identify hits to characterize in more detail.

Isolation and sequence-verification of colonies

Identified screening hits and designs of interest were characterized in greater detail and verified through DNA sequencing and electrospray ionization mass spectrometry (ESI-MS; mass spectrometry methods below). Single clones of identified hits were obtained by the following procedure. A stab from the corresponding polyclonal glycerol stock was diluted into 200 μL of sterile water, and 2 μL of this was further diluted into another 200 μL of water. 20 μL of the diluted cell suspension was spread on an LB-agar plate supplemented with kanamycin, and

grown at 37 °C overnight. The following day, 1-3 of the colonies were picked by careful swabbing with a pipette tip and transferred to individual PCR tubes containing 100 µL Milli-Q water. Then, 94 µL of each isolated colony in water was used to inoculate a 1 mL LB culture containing 50 µg/mL of kanamycin for overnight growth and the rest of the isolated colony in water was used for DNA sequencing (described below). The overnight LB+kanamycin cultures were incubated at 37 °C and 225 rpm until the following day when they were visibly opaque (~16-20 hours of growth). Glycerol stocks were made for each of the isolated clones that had verified DNA sequences containing the gene of interest. In the case of a single design having 2+ isolated clones that were DNA-sequence verified, one was chosen at random for glycerol stocks and future experiments. Note that all of these steps were performed using sterile technique under flame.

The DNA sequencing of isolated colonies was performed using Sanger sequencing. ColonyPCR with T7 and T7-term primers was performed to amplify the expressing region of the DNA contained in the isolated cells. The PCR reaction mixture comprised 2 µL of the single colony suspended in nuclease-free water, 1 µL of 10 µM T7 and T7 terminal primers designed to flank the target sequence, 12.5 µL of GoTaq Green 2X Master Mix (which includes Taq DNA polymerase and the accompanying buffer), and 8.5 µL of additional nuclease-free water. The thermal cycling conditions were as follows: an initial lysis and denaturation step at 95°C for 3 minutes, followed by 31 cycles of denaturation (95 °C, 30 s), annealing (58 °C, 30 s), and extension (72 °C, 1 min), culminating in a final extension step at 72 °C for 5 minutes. The success of the PCR reactions was verified by DNA gel electrophoresis using 1.2% agar/TAE gel with SybrSafe dye. Subsequently, 10 µL of unpurified PCR products were sent for sequencing at Genewiz/Azenta, and the results were compared against the design sequences.

Large-scale protein expression with IPTG

DNA sequence-verified, single colony glycerol stocks were used to inoculate 5 mL overnight cultures in LB media with 50 µg/mL of kanamycin that were incubated at 37 °C and 225 rpm. After 16-20 hours, the 5 mL cultures were added to 50 mL of TBII+kanamycin media in 250 mL baffled Erlenmeyer culture flasks, and incubated for 90 minutes at 37 °C and 225 rpm. After the time elapsed, 55 µL of 1 M IPTG solution was added to each of the 55 mL cultures to induce expression. The flasks were incubated at 37 °C and 225 rpm for an additional 2 hours before the temperature was lowered to 25 °C where the flasks were incubated for 20-24 hours to promote sufficient expression. The following day, the cultures were transferred into 50 mL falcon tubes, and cell pellets were collected by centrifugation (4,198g, 8 min, 25 °C). The cell pellets were stored at -80 °C until purification. Note that all steps involving inoculation, media transfers, and IPTG additions were performed with sterile technique under flame.

Protein purification for characterization

The frozen cell pellets were thawed, lysed, and purified by (1) affinity chromatography with Strep-Tactin® resin and (2) size-exclusion chromatography. First, three buffers were prepared with Milli-Q water as follows: **W1 buffer** (100 mM Tris-HCl, 150 mM NaCl, 1 mM EDTA, pH 8.0), **W2 buffer** (100 mM Tris-HCl, 150 mM NaCl, pH 8.0), and **E buffer** (40 mM HEPES, 50 mM NaCl, 2.5 mM desthiobiotin, pH 8.0). Additionally, a **lysis buffer** was made from

the W2 buffer where it was supplemented with 100 µg/mL lysozyme, 10 µg/mL of DNAse I, and 1 Pierce protease inhibitor tablet per 50 mL of W2 buffer.

The thawed cell pellets were resuspended in 20 mL of lysis buffer and mechanically lysed by ultrasonication (IBA Lifesciences) at 80% amplitude with 10s on/off pulse cycles for 5 minutes of total “on” time. Note that this was all performed in an ice bath to prevent significant heating of the lysate. The lysates were then centrifuged (4,198 g, 30 min, 4 °C) to collect the supernatant.

Plastic 25 mL gravity flow-through chromatography columns were prepared with 6-10 mL of new Strep-Tactin® resin in a 50% suspension such that 1 CV = 3-5 mL of resin. The columns were equilibrated 2 times with 2.5 CV of W1 buffer followed by 1 addition of 1 CV of W2 buffer. Note that this step and the following steps for affinity chromatography were carried out in a 4 °C room using only gravity flow-through. Once the columns were equilibrated, the lysate supernatant was gently added over the columns and allowed to completely flow through. The columns were then washed 3 times with 1 CV of W1 buffer, 2 times with 1 CV of W2 buffer, and 1 time with 0.5 CV of E buffer. Next, the bound protein in each column was eluted into a falcon tube with 5 additions of 0.5 CV of E buffer. The collected elutants were set aside for further purification by size-exclusion chromatography and the columns were washed 1 time with 2 CV of E buffer before being regenerated by 3 additions of 5 CV of regeneration buffer (obtained as 10x concentrated and diluted in Milli-Q water). Note that the resin was resuspended several times during regeneration so that the columns had a homogenous bright red color after regeneration; the regenerated columns were overlaid with 40-60% v/v ethanol and water to prevent bacterial growth, capped at both ends, and stored at 4 °C for future use.

The collected protein eluants were spin-concentrated to a final volume of 1.0~1.1 mL using a 3 kDa MWCO centrifugal concentrator. The concentrated elutants were then purified by size-exclusion chromatography on an ÄKTA pure™ chromatography system using a Superdex 200 Increase 10/300 GL column with a running buffer of 40 mM HEPES, 50 mM NaCl, pH 8.0. The resulting monomeric fractions of the purified samples were collected and immediately used in downstream experiments. Protein molecular weight was confirmed with electrospray ionization mass spectrometry.

<u>Kinetic analysis</u>

The Michaelis-Menten kinetics of each purified design of interest were determined by incubating the protein samples with zinc sulfate and the fluorogenic substrate 4MU-PA (WuXi) while monitoring the fluorescent signal of the product formation at 25 °C with an excitation/emission wavelength 365 nm and 445 nm, respectively. Before the kinetics, the purified protein samples were diluted to 10x of their desired concentration in the reaction using **reaction buffer** (40 mM HEPES, 50 mM NaCl, pH 8.0). Additionally, zinc sulfate dissolved in Milli-Q water was added to each of these 10x concentrated purified protein stocks to reach a desired [zinc]:[enzyme] ratio. Note that the numeric details for the concentrations of protein and zinc are described in the final paragraph. The samples containing protein and zinc sulfate at 10x the reaction concentration were incubated at 25 °C for 15 minutes. Additionally, stock solutions

for the 4MU-PA substrate (WuXi) and 4MU fluorescent product (Sigma) were made by dissolving each of the solids in DMSO.

Two sets of serial dilutions of the 4MU product (1.4 - 45 μM and 0.9 - 30 μM) were made in 95% reaction buffer and 5% DMSO to construct a fluorescence calibration curve, where each point was measured at 25 °C in a 96-well half area plate using 100 μL. Every point was measured in triplicate and averaged over 4 minutes of measurements. The calibration curve was fitted with a linear regression equation to convert fluorescence into the product concentration of 4MU. New calibration curves were made before every series of experiments.

After constructing a standard curve, serial dilutions of the 4MU-PA substrate (1.1 - 144 μM reaction concentration) were made in 94.5% v/v reaction buffer and 5.5% v/v DMSO at concentrations 1.11x the desired concentration in the reaction to account for a 0.9x dilution with the 10x concentrated protein-zinc sample. Kinetic reactions were performed by mixing 10 μL of the 10x concentrated protein-zinc sample with 90 μL of the prepared substrate solution in a 96-well half area plate. The reactions were allowed to shake for 3s at 282 cpm immediately after mixing the enzyme and substrate. Each reaction was monitored for 4 minutes at 25 °C and all substrate concentration points were measured in triplicate. Note that the DMSO was 5.0% v/v in the final reaction mixture. The resulting curves were analyzed by first subtracting the fluorescence of the background reaction at each substrate concentration point for the matching zinc concentration in the reaction buffer. The resulting fluorescence curve was manually set to start at y=0 fluorescence units such that a linear regression (y = mx + b) could be calculated for the linear initial velocity with b=0 (i.e., only the slope, m, needed to be determined). The calculated slopes for the initial velocities at each substrate concentration point were transformed to be expressed in terms of [4MU product]/[enzyme] using the calibration curve and known enzyme concentration. The steady-state parameters k_{cat} and K_M were determined by nonlinear regression fitting all data points to the Michaelis-Menten equation: $v_o/[E]_o = k_{cat}[S]/(K_M + [S])$, where v_o denotes the initial velocity, $[E]_o$ the enzyme concentration, and [S] the substrate concentration. Standard deviations were calculated from the standard error of the fit considering each triplicate value as an individual data point.

To determine the uncatalyzed reaction rate constant (k_{uncat}), background hydrolysis reactions were carried out without any enzyme at 25 °C in the reaction buffer, initiated by 4MU-PA substrate (1.1 - 72 μM). Each reaction was performed in triplicate. The uncatalyzed rate constant was determined from the initial rates of these reactions by fitting all data points to the equation $v_0 = k_{uncat}[S]$ using linear regression. We measured $k_{uncat} = 0.000021 \pm 0.000001\ s^{-1}$ for 4MU-PA in the reaction conditions (40 mM HEPES, 50 mM NaCl, pH 8.0, 5% v/v DMSO).

From the first design campaign, the kinetics for A1 (ZETA_1) were carried out at $[E]_o$ = 100 nM, 40:1 [Zn(II)]:$[E]_o$ (4 μM zinc sulfate in reaction), and 1.1 - 72 μM 4MU-PA substrate. The kinetics for the active A1 mutants (D67A, H130A, and N17A) were carried out at $[E]_o$ = 100 nM (D67A) or $[E]_o$ = 500 nM (H130A and N17A), 40:1 [Zn(II)]:$[E]_o$, and 1.1 - 72 μM 4MU-PA substrate to be consistent with the measurement of the wild-type A1. The kinetics for C4 were carried out at $[E]_o$ = 3 μM, 10:3 [Zn(II)]:$[E]_o$ (10 μM zinc sulfate in reaction) due to precipitation of

this design above this zinc concentration, and 1.1 - 144 μM 4MU-PA substrate. The kinetics for all of the other characterized hits (A8, B9, F7) were carried out at $[E]_o$ = 2 μM, 10:1 $[Zn(II)]:[E]_o$ (20 μM zinc sulfate in reaction), and 1.1 - 144 μM 4MU-PA substrate. Experiments were performed in biological duplicates, with the results being in close agreement. We herein report representative results for each design/variant.

From the second design campaign, the kinetics for ZETA_2 (well position H10) and ZETA_3 (well position H6) were carried out at $[E]_o$ = 100 nM, 10:1 $[Zn(II)]:[E]_o$ (1 μM zinc sulfate in reaction), and 1.1 - 72 μM 4MU-PA substrate. The kinetics for ZETA_4 (well position C5) were carried out at $[E]_o$ = 500 nM, 10:1 $[Zn(II)]:[E]_o$ (5 μM zinc sulfate in reaction), and 1.1 - 72 μM 4MU-PA substrate. Experiments were performed in biological duplicates, with the results being in close agreement. We herein report representative results for each design/variant.

Total turnover number (lower bound estimation)

The lower bound for the total turnover number of A1 (ZETA_1) was estimated using an activity assay monitoring the fluorescent signal generated by the hydrolyzed coumarin product, which was converted into product concentration by a standard curve and taken as a ratio over the enzyme concentration to yield turnover number. The 4MU-PA substrate was loaded at a concentration just below its precipitation limit under the experimental conditions and the enzyme concentration was set as low as possible, while still achieving a high rate above background, to maximize the number of possible turnover events. Purified A1 enzyme was prepared to a stock concentration of 1.0 μM in reaction buffer (40 mM HEPES, 50 mM NaCl, pH 8.0) and supplemented with 40 μM zinc sulfate dissolved in Milli-Q water. Additionally, a stock solution of 4MU product was made in anhydrous DMSO and subsequently used to make serial dilutions of 4MU (4.7 μM - 150 μM) in 95% v/v reaction buffer and 5% v/v DMSO for standard curves. The 4MU-PA substrate was also dissolved in anhydrous DMSO to make a stock solution; this was used to prepare an aqueous solution of 4MU-PA substrate (150 μM reaction concentration) in 94.5% v/v reaction buffer and 5.5% v/v DMSO at concentrations 1.11x the desired concentration in the reaction to account for a 0.9x dilution with the 10x concentrated A1-zinc stock.

After these solutions were made, 100 μL of the 4MU product standards were added to a 96-well half area plate. Reactions were then prepared in the plate by mixing 10 μL of the A1-zinc stock with 90 μL of the prepared substrate solution such that the final A1 concentration was 100 nM, the final zinc sulfate concentration was 4 μM, and the final substrate concentration was 150 μM. Additionally, note that the DMSO was 5.0% v/v in the final reaction mixture. The non-enzymatic background reaction in aqueous reaction buffer with 5.0% v/v DMSO and 4 μM zinc sulfate was also prepared in this plate. After the plate of standards, backgrounds, and reactions on a 100 μL scale was prepared, the plate was immediately covered with a transparent optical film, to prevent evaporation over several hours, and allowed to shake for 5s at 282 cpm. The fluorescent signal of each well in the plate was monitored for 3 hours at 25 °C using an excitation/emission wavelength of 365 nm and 445 nm, respectively. All standards, background reactions, and enzyme-catalyzed reactions were measured in triplicate.

The fluorescence progress curves corresponding to product generation for the background and enzyme-catalyzed reactions were averaged and plotted with the averaged fluorescence signal lines corresponding to the calibration standards of 4MU (Fig. S8A). The calibration curve was constructed by measuring the fluorescence signal of 4MU at each set concentration by averaging the signals during the course of the reaction monitoring experiment, in triplicate (Fig. S8B). A nonlinear calibration curve was observed >75 μM product, which we found to be independent of instrument gain (i.e., the calibration curve always had the same shape), likely due to the common phenomenon of fluorescence self-quenching at high fluorophore concentrations.[63–65] Note that all other experiments only required measuring relatively low fluorescence (i.e., in the linear range) and thus all used linear calibration curves. To overcome this experiment-specific challenge, we used previously reported nonlinear calibration curve fitting methods for interpolation, specifically higher-order polynomial fitting.[66–70] After converting the fluorescence signal into [4MU] using a 4th-order polynomial fit, the y-axis was transformed into units of 4MU product concentration (Fig. S8C). This allowed for the background reaction to be directly subtracted from the enzyme-catalyzed reaction so that any product generated could be attributed to enzymatic catalysis. Turnover number was then calculated based on the ratio of [product]:[enzyme] for the background-subtracted reaction using 100 nM of enzyme to produce the final plot (Fig. 3e).

It should be noted that the conclusion from this experiment (i.e., A1 achieving >1000 turnovers) is not affected by disregarding the interpolation using the nonlinear fit and exclusively considering the discrete fluorescence-[4MU] relationship of the calibration samples (Fig. 8A). By the end of the A1-catalyzed reaction, the fluorescence signal has reached a known value corresponding to 150 μM 4MU, and the background reaction has reached a known value corresponding to 38 μM 4MU. Converting this difference into enzyme turnover number ultimately yields a conservative lower bound estimate of the total turnover number since all of the substrate was hydrolyzed and it was not possible to further enhance the [product]:[enzyme] ratio; we observed limited capacity to raise the initial substrate concentration without precipitation or to reduce the enzyme concentration without substantially lowering the activity-to-background ratio. Thus, A1 (ZETA_1) may catalyze more turnovers.

Preparing the apo-enzyme by zinc chelation, and the zinc readdition experiment

The chelation and removal of zinc from the purified A1 (ZETA_1) protein and its mutants were accomplished by dialyzing the sample in a chelation buffer containing 1,10-phenanthroline, a potent zinc chelator, based on a previous protocol.[71] Briefly, 180 mg of 1,10-phenanthroline was dissolved in 500 mL of reaction buffer (40 mM HEPES, 50 mM NaCl, pH 8.0), reaching a final concentration of 2 mM in the **chelation buffer** (40 mM HEPES, 50 mM NaCl, 2 mM 1,10-phenanthroline, pH 8.0). Note that the buffer required heating and vigorous stirring over a stir plate to fully dissolve the 1,10-phenanthroline; the chelation buffer was cooled to 4 °C before dialysis. Wild-type A1 and its mutants were individually loaded into 3.5 kDa MWCO dialysis cassettes and dialyzed against chelation buffer at 4 °C in a beaker with a volume exceeding 400x the sample volume. The beaker had gentle stirring and the dialysis was allowed to take place for 12-24 hours before the dialyzed chelation buffer was exchanged for fresh chelation buffer where the dialysis was allowed to continue for an additional 12-24 hours. After this point,

the samples were completely removed of zinc. Part of the wild-type apo-A1 sample was directly used in the activity assay and zinc readdition experiment, while the apo-mutants and leftover wild-type apo-A1 sample were redialyzed in reaction buffer to remove the 1,10-phenanthroline for accurate zinc binding affinity characterization (described in a separate methods section below).

The concentration of the wild-type apo-A1 sample in chelation buffer was measured in triplicate (70 μM) and diluted in chelation buffer to a stock concentration of 1.0 μM before the activity assay, which was performed at 100 nM apo-A1 concentration (containing ~200 μM 1,10-phenanthroline); wild-type A1 was also prepared at a stock concentration of 1.0 μM and supplemented with 40 μM of zinc sulfate dissolved in Milli-Q water as a control. Additionally, a stock solution of 4MU-PA (WuXi) was prepared in anhydrous DMSO and subsequently used to make an aqueous solution of 4MU-PA substrate (1.8 μM reaction concentration) in 94.5% v/v reaction buffer and 5.5% v/v DMSO at concentrations 1.11x the desired concentration in the reaction to account for a 0.9x dilution with the 10x concentrated apo-A1 stock. Kinetic reactions were performed by mixing 10 μL of the 10x concentrated apo-A1 stock sample with 90 μL of the prepared substrate solution in a 96-well half area plate where the fluorescent signal of the generated product was monitored at an excitation/emission wavelength of 365 nm and 445 nm, respectively. The reactions were shaken for 3s at 282 cpm immediately after mixing the enzyme and substrate. Each reaction was monitored for 30 minutes at 25 °C and all substrate concentration points were measured in triplicate. Note that the DMSO was 5.0% v/v in the final reaction mixture. The background reactions without added enzyme were also recorded for 4MU-PA hydrolysis in the presence of 4 μM zinc sulfate and without zinc sulfate to match the conditions of the wild-type A1 reaction and apo-A1 reaction, respectively.

After the reactions proceeded for 30 minutes, the monitoring was briefly stopped for 2 minutes and 1 μL of 10 mM zinc sulfate dissolved in Milli-Q water was added to the apo-A1 and zinc-absent background reactions, achieving a final zinc sulfate reaction concentration of approximately 100 μM. The reactions were again shaken for 3s at 282 cpm before they were monitored for an additional 30 minutes at 25 °C. Note that a large excess of zinc sulfate was added to the apo-A1 reaction compared to the wild-type A1 reaction because the apo-A1 reaction still had a considerable amount of 1,10-phenanthroline, approximately 200 μM after the 10-fold dilution in the reaction mixture. Thus, it was necessary to add enough zinc to overcome the 1,10-phenanthroline binding, which typically occurs at a 2:1 and 3:1 stoichiometric ratio since zinc has 6 possible coordination sites, so that there was enough zinc to bind in the apo-A1 enzymes.[72] However, it should be noted that there is a possibility that 1,10-phenanthroline was binding zinc in the active site and inhibiting the holo-A1 (i.e., zinc-bound) structure such that the readdition of excess free zinc simply out-competed the holo-A1 zinc for 1,10-phenanthroline binding; therefore, this would stop the inhibition of the holo-A1, as this phenomenon has been reported for some native metalloenzymes.[72–74] Nonetheless, the outcome of demonstrating the zinc dependence of A1 (ZETA_1) remains the same.

Zinc binding affinity of A1 (ZETA_1) and its mutants

The Zn(II) ion binding of the design A1 (ZETA_1) and its mutants was evaluated using a competition binding assay with the ratiometric dye Mag-Fura-2 as reporter and competitor.[75] The significant change in absorbance ratio at 323/342 nm (absorbance maximum of metal-bound state to isosbestic point) was used for this analysis. First, the dissociation constant of the dye (K_D(Mag-Fura-2, Zn(II)) = 12 ± 2 nM) was determined in competition with nitrilotriacetic acid (NTA); it was measured that K_D(NTA, Zn(II)) = 830 pM under the experimental conditions of I = 0.05 M, pH 8.0, and 25 °C. Afterward, the dissociation constants for the proteins were determined in competition with Mag-Fura-2. For each measurement, the proteins, Mag-Fura-2, and/or NTA were each utilized at a 10 μM concentration in a 10 μL buffered solution with varying Zn(II) ion concentrations. The samples were incubated for 3 days in the dark at 25 °C to ensure binding equilibrium. Duplicate measurements were performed for each experiment.

Circular dichroism (CD) spectroscopy

To perform CD spectroscopy, it was necessary to exchange from the HEPES-based buffer to a low-salt, Tris-based buffer for accurate measurement at low wavelengths; otherwise, the machine voltage would jump too high and noise the measurements at these wavelengths. To exchange buffers, the enzymes were dialyzed overnight at 4 °C in 25 mM Tris, 25 mM NaCl, pH 8.0 aqueous buffer (**CD buffer**).

CD spectra were recorded for purified A1 (ZETA_1) enzyme (28 μM) in aqueous **CD buffer** (25 mM Tris, 25 mM NaCl, pH 8.0) using a JASCO J-1500 CD Spectrophotometer and a 1-mm pathlength cuvette. Initial spectra were collected at 25 °C from 200-250 nm with a bandwidth of 0.1 nm. Thermal denaturation was performed by heating the sample from 25 °C to 95 °C at a rate of 1 °C min^{-1}, with data points recorded every 1 °C at 222 nm (bandwidth 0.1 nm), using a 5 s averaging time per 0.1 °C increment. Full spectra (200-250 nm, bandwidth 0.1 nm) were recorded at 10 °C intervals during heating from 25-95 °C and at 95 °C. After heating, the sample was cooled back to 25 °C over approximately one hour, and final spectra were collected using the same parameters.

Additionally, CD spectra were recorded for the other 4 hits from the first design campaign (A8, B9, C4, and F7) and the best designs from each of the 3 unique scaffolds from the second design campaign (ZETA_2, ZETA_3, and ZETA_4). All designs were dialyzed in the aqueous **CD buffer** as given above. Spectra were collected at 25 °C from 200-250 nm with a bandwidth of 0.1 nm. The concentrations of enzyme used in each experiment were as follows: A8 = 19 μM, B9 = 22 μM, C4 = 26 μM, F7 = 28 μM, ZETA_2 = 33 μM, ZETA_3 = 39 μM, and ZETA_4 = 17 μM. All enzymes were assumed to be purified and analyzed in the zinc-bound state.

All CD measurements were initially recorded as ellipticity (θ) in millidegrees (mdeg). These values were converted to Mean Residue Ellipticity (MRE), expressed in units of $deg \cdot cm^2 \cdot dmol^{-1}$, to allow standardized comparison of secondary structures. MRE was calculated using the following equation: $MRE = \theta / (\ell \cdot [P] \cdot N_{residues})$ where θ is the measured ellipticity in mdeg, ℓ is the pathlength in mm, [P] is the protein concentration in M, and $N_{residues}$ is the length of the protein sequence.

SDS-PAGE gel electrophoresis and staining

SDS-PAGE gel electrophoresis was performed with 15- or 26-well precast BioRad AnykD gels. SDS-PAGE samples were prepared by mixing equal volumes of the desired protein sample and 2x-concentrated Laemmli buffer for denaturation. The prepared SDS-PAGE samples were then loaded into the gel in 6 µL aliquots and electrophoresis was performed in SDS-PAGE buffer (25 mM Tris, 192 mM Glycine, and 0.1% (w/v) sodium dodecyl sulfate) at 180 V for 28 minutes until the lowest bands traveled sufficiently through the gel. Note that ladders (Dual Xtra) were also loaded into the gels for reference. After electrophoresis, the gels were removed from their cassette, rinsed with water, and stained for 9 minutes using the eStain L1C protein staining kit. The gels were again rinsed in water and immediately imaged.

Electrospray ionization (ESI) mass spectrometry

MS data for the identified hits and A1 (ZETA_1) mutants were acquired on an Agilent 1200series LC G6230B TOF LC-MS with an AdvanceBio RP-Desalting column (A: H2O with 0.1% Formic Acid, B: Acetonitrile with 0.1% Formic Acid). Final protein concentrations were adjusted to 1-2 mg/mL in 40 mM HEPES, 50 mM NaCl, pH 8.0. Subsequent data deconvolution was performed in Bioconfirm using a total entropy algorithm. All data are presented in Table S1.

Protein purification and preparation for crystallography

Prior to crystallography, the original eBlock DNA fragments corresponding to the designs intended for crystallography were cloned into a different custom vector (FP0017; 5725 base pairs) encoding a kanamycin-resistance gene for positive selection, a T7 promoter, and a N-terminal His-tag followed by a TEV-cleavage site, using a published protocol described in a previous section.[61] The resulting plasmids were used to transform *E. coli*, from which isolated monoclonal colonies were obtained, sequence-verified, and preserved as glycerol stocks, as detailed in previous sections. The proteins were then expressed at scale with the same procedure as before. The expressed proteins were obtained as `MS-HHHHHH-GSGSGSGSWG-ENLYFQS-G-design-GS` where HHHHHH is the His-tag for immobilized metal affinity chromatography (IMAC) purification, and ENLYFQS is the TEV recognition site which cleaves the amide bond between QS.

The frozen cell pellets containing the expressed protein were thawed, lysed, and purified by (1) IMAC with Ni(II) resin and (2) size-exclusion chromatography. First, two buffers were prepared with Milli-Q water as follows: **IMAC wash buffer** (40 mM HEPES, 50 mM NaCl, 30 mM imidazole, pH 8.0), and **IMAC elution buffer** (40 mM HEPES, 50 mM NaCl, 300 mM imidazole, pH 8.0). Additionally, a **lysis buffer** was made from the IMAC wash buffer where it was supplemented with 100 µg/mL lysozyme, 10 µg/mL of DNAse I, and 1 Pierce protease inhibitor tablet per 50 mL of buffer. The cells were then lysed as described in a previous section to yield 20 mL of lysate for each cell pellet derived from 50 mL of culture.

Glass 50 mL gravity flow-through chromatography columns were prepared with 2-4 mL of fresh Ni-NTA Superflow® resin in a 50% suspension such that 1 CV = 1-2 mL of resin. The columns were equilibrated 1 time with Milli-Q water, then 2 times with 5 CV of IMAC wash buffer.

Note that this step and the following steps for affinity chromatography were carried out in a 4 °C room using only gravity flow-through. Once the columns were equilibrated, the 20-40 mL of lysate supernatant was gently added over the columns and allowed to completely flow through. The columns were then washed 5 times with 3 CV of IMAC wash buffer, and 1 time with 0.6 CV of IMAC elution buffer. Next, the bound protein in each column was eluted into a falcon tube with 2 additions of 1 CV of IMAC elution buffer followed by 1 final addition of 0.5 CV of IMAC elution buffer. The collected elutants were set aside for further purification by size-exclusion chromatography and the columns were washed 2 times with 5 CV of IMAC elution buffer and 2 times with 5 CV of Milli-Q water. The resin was then resuspended in 40-60% v/v ethanol and water to prevent bacterial growth and stored for later recharging.

The collected protein eluants were spin-concentrated to a final volume of 1.0~1.1 mL using a 3 kDa MWCO centrifugal concentrator. Purified hyperTEV[76] protein containing a His-tag, expressed and purified in-house, was then supplemented into the resulting concentrated eluants at a 1:20 concentration ratio (hyperTEV : protein). The concentrated eluants containing the purified protein and hyperTEV were then added to a 3.5 kDa MWCO dialysis cassette and dialyzed overnight in 40 mM HEPES, 50 mM NaCl, 1 mM DTT, pH 8.0 **hyperTEV reaction buffer** to reduce the imidazole concentration and add DTT, which is important for the hyperTEV activity. The dialysis buffer was swapped the next day for fresh hyperTEV reaction buffer, and the reactions were allowed to proceed another 6-24 hours. This reaction yielded two protein fragments, `MS-HHHHHH-GSGSGSGSWG-ENLYFQ` and `S-G-design-GS`, alongside the His-tagged hyperTEV.

IMAC purification following a simplified protocol from the one described above was performed to purify out the cleaved N-terminus and hyperTEV, and the flow-through containing the `S-G-design-GS` was collected. Briefly, IMAC columns were prepped and washed by the prior protocol, the hyperTEV-protein reaction mixture was added on top of the resin, the flow-through was collected, and the resin was washed with 2-4 CV of IMAC wash buffer which was also collected. The column was then flushed with IMAC elution buffer to discard the bound hyperTEV and His-tagged N-terminal fragment, and the resin was stored for later recharging. The collected protein flow-through was spin-concentrated to a final volume of 1.0~1.1 mL using a 3 kDa MWCO centrifugal concentrator. The concentrated protein flow-through was then purified by size-exclusion chromatography on an ÄKTA pure™ chromatography system using a Superdex 200 Increase 10/300 GL column with a running buffer of 40 mM HEPES, 50 mM NaCl, pH 8.0.

The resulting monomeric fractions of the purified samples were collected and diluted in Milli-Q water containing 50 mM NaCl to reduce the HEPES concentration to 10 mM, such that the buffer became 10 mM HEPES, 50 mM NaCl, pH 8.0. The proteins were then concentrated in this buffer to the desired concentration for crystallography (~20-65 mg/mL). As mentioned above, the final proteins for crystallography were of the sequence `SG-design-GS`.

X-ray crystallography

We attempted to solve structures for both ZETA_1 and ZETA_2, but only ZETA_2 successfully crystallized and diffracted. Both proteins were incredibly soluble (observed >65

mg/mL) in the 10 mM HEPES, 50 mM NaCl, pH 8.0 **initial buffer** which they were concentrated in to submit for crystal condition screening. However, zinc sulfate was observed to precipitate out in the initial buffer conditions at concentrations above approximately ~300-400 μM; therefore, we were unable to raise the zinc sulfate concentration to match the concentrated protein stoichiometry when submitting samples to crystal condition screening, resulting in protein crystals in their apo-state.

Crystallization experiments were conducted using the sitting drop vapor diffusion method. Initial crystallization trials were set up in 200 nL drops using the 96-well plate format at 20 °C. Crystallization plates were set up using a Mosquito LCP from SPT Labtech, then imaged using UVEX microscopes and UVEX PS-256 from JAN Scientific. Diffraction quality crystals for ZETA_2 formed in 0.2 M $MgCl_2$, 0.1 M Tris pH 8.5, and 30% (w/v) PEG4000 at 50 mg/mL protein concentration. To get the Zn(II)-bound structure of ZETA_2, crystals from this condition were soaked in mother liquor + 250 mM zinc acetate for 3 days before freezing in liquid nitrogen for synchrotron data collection.

Diffraction data was collected at the Advanced Photon Source beamline NE-CAT 24ID-E at wavelength of 0.97920 Å. X-ray intensities and data reduction were evaluated and integrated using XDS[77] and merged/scaled using Pointless/Aimless in the CCP4 program suite[78]. Structure determination and refinement starting phases were obtained by molecular replacement using Phaser[79] using the designed model for the structures. Following molecular replacement, the models were improved using Phenix[80]/Refmac[78], and using simulated annealing. Structures were refined in Phenix[80]/Refmac[78]. Model building was performed using COOT[81]. The final model was evaluated using MolProbity[82]. Data collection and refinement statistics are recorded in Extended Data Table 2. Data deposition, atomic coordinates, and structure factors reported in this paper have been deposited in the Protein Data Bank (PDB, http://www.rcsb.org/) with PDB accession codes 9PYJ (apo-ZETA_2) and 9PYL (holo-ZETA_2).

Statistics

All bar graphs presented in this manuscript show the bar height as the mean and the error bars as 1 standard deviation. All box plots presented in this manuscript show the box extending from the first quartile (Q1) to the third quartile (Q3) of the data, with a line at the median (Q2); the whiskers extend from the box to the farthest data point lying within 1.5x the interquartile range (IQR) from the box. All individual points plotted on curves represent the mean value with error bars representing 1 standard deviation. All curves derived from fluorescent data with respect to time display the mean value as the central line for a given time with the shadow representing 1 standard deviation in either direction. There was a significance test performed in Fig. S16 which employed a two-sided Wilcoxon rank-sum test, a nonparametric statistical test. The results and analysis of this test are described in the corresponding figure caption.

5. SUPPLEMENTAL FIGURES

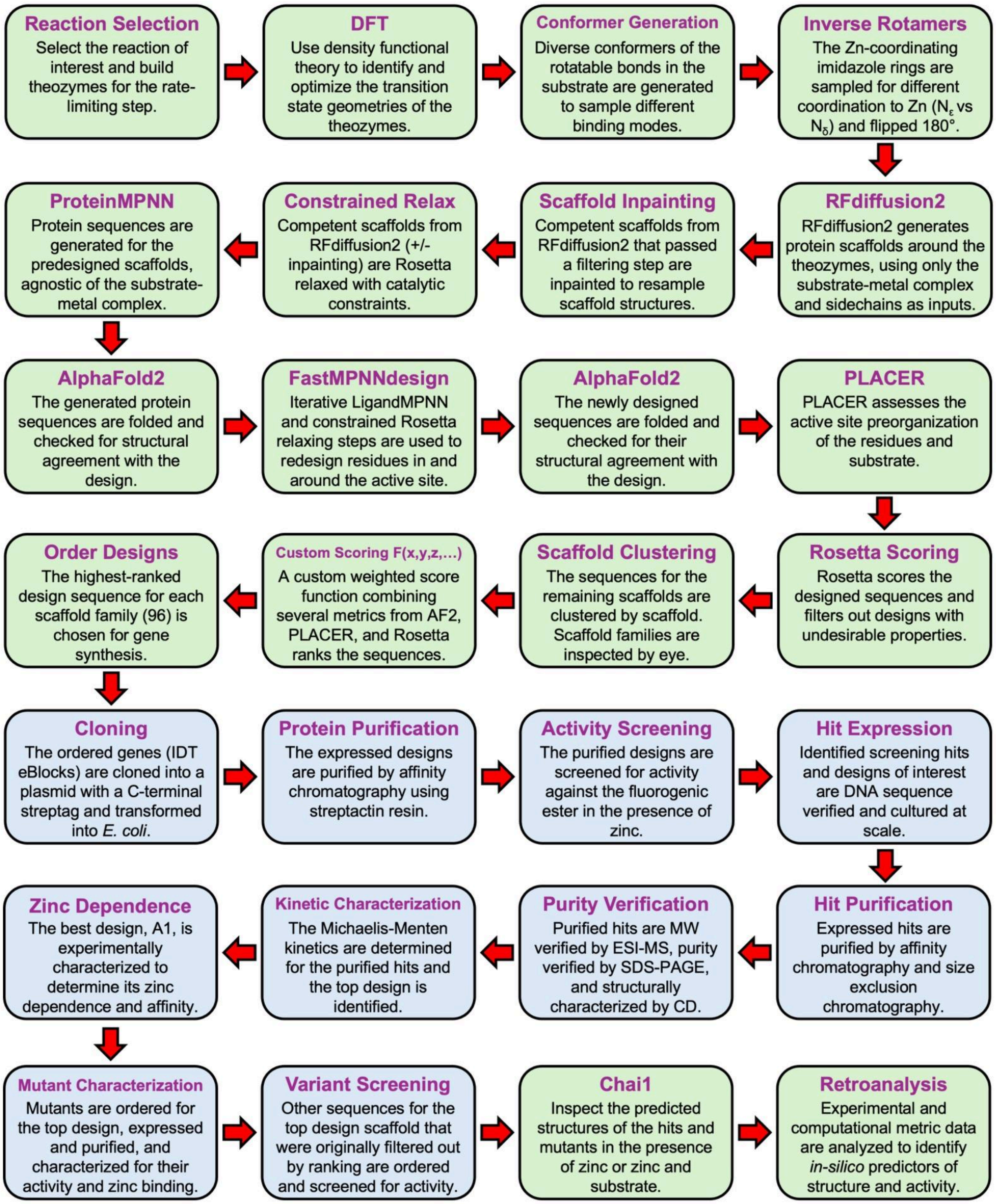

Fig. S1. Detailed flow chart of the design and experimental pipeline for the computational design of de novo metallohydrolases (first design campaign). The green color represents steps done *in silico* while the blue color represents experimental steps done in the wet lab.

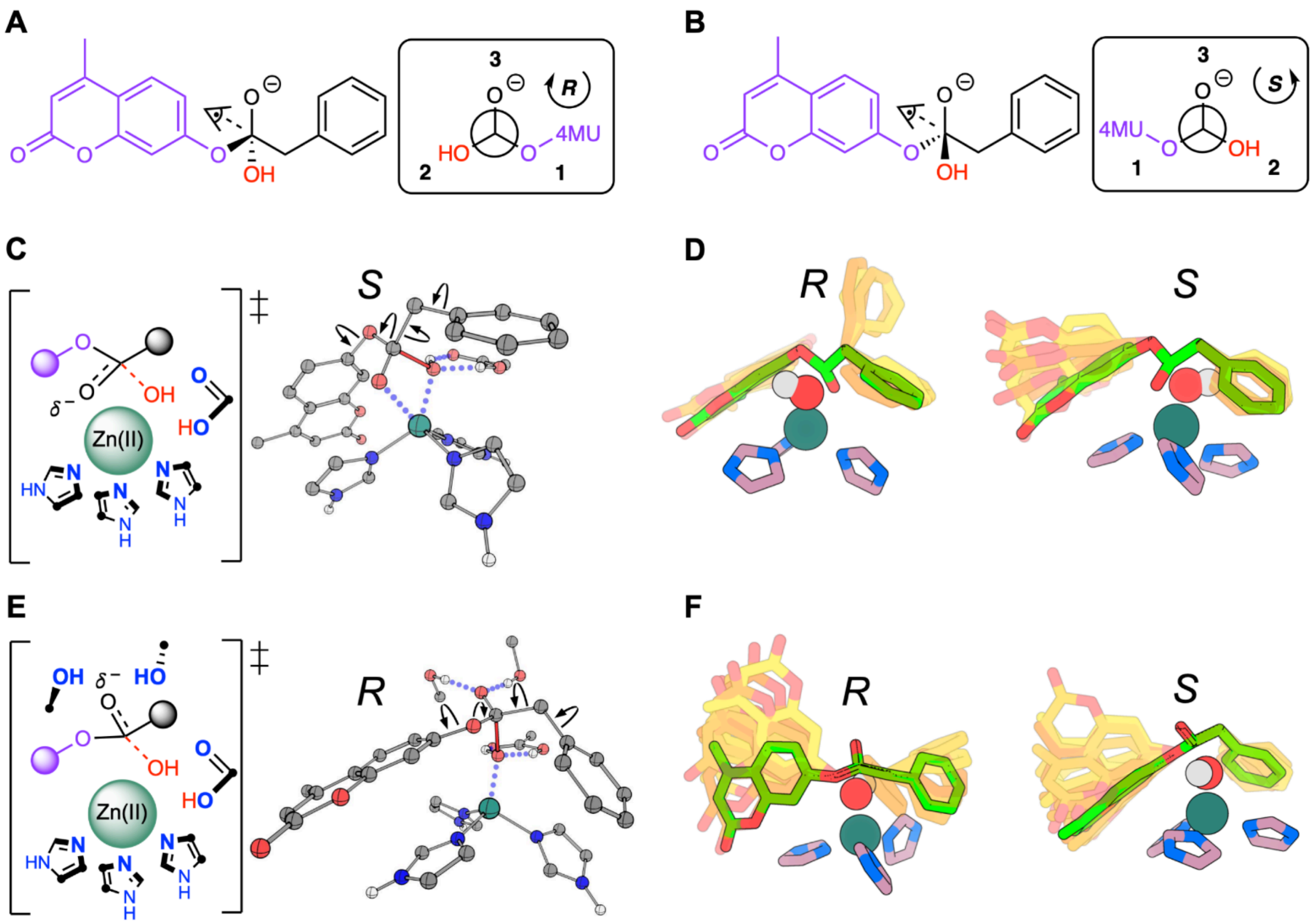

Fig. S2. Theozyme inception and transition state optimization with density functional theory followed by substrate conformer generation. (**A**,**B**) Tetrahedral intermediate enantiomers. (**C**,**E**) Example theozymes for zinc-hydroxide nucleophilic attack of the 4MU-PA ester modeling (**C**) oxyanion stabilization via zinc or (**E**) oxyanion stabilization via protein hydrogen bonds. 2D representation (left) and 3D DFT model (right). Arrows on the 3D model represent sampled conformational flexibility. (**D**,**F**) Substrate conformers (yellow spectrum) from each DFT model (green). Note that the general base, and the oxyanion hole in the case of the (E,F), were not modeled during the scaffold generation with RFdiffusion2; these were left to be found during sequence design due to the flexibility of residues that can accomplish those tasks.

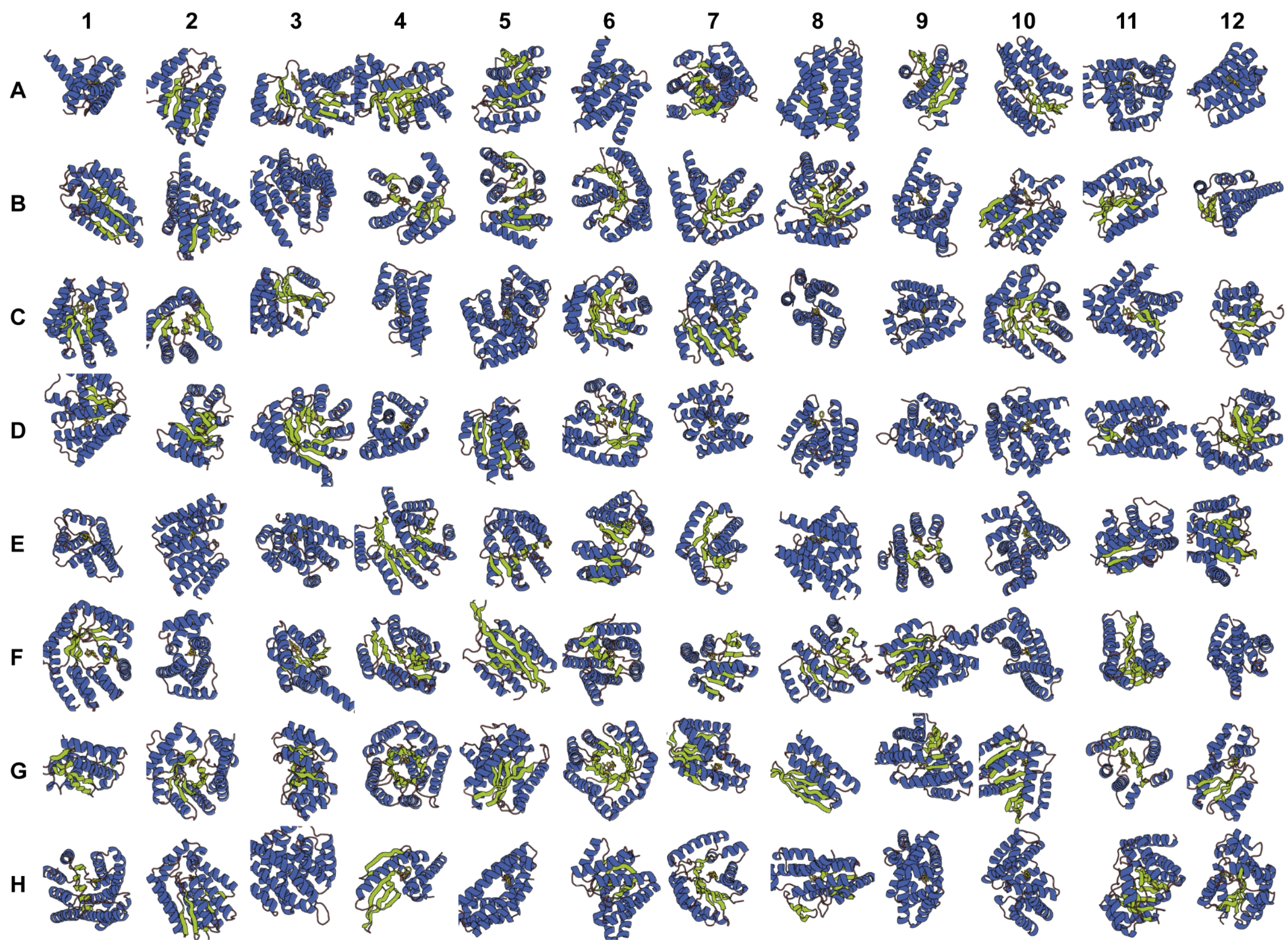

Fig. S3. Structural panel of the 96 ordered designs spanning 96 unique backbones from the first design campaign. Each structure corresponds to the well it was ordered in based on its *in silico* ranking (A1 = best ranking; H12 = worst ranking). The secondary structures are colored (green = beta-sheets; blue = alpha-helices). Structures range in length from 140 - 275 amino acids and have a range of diverse folds.

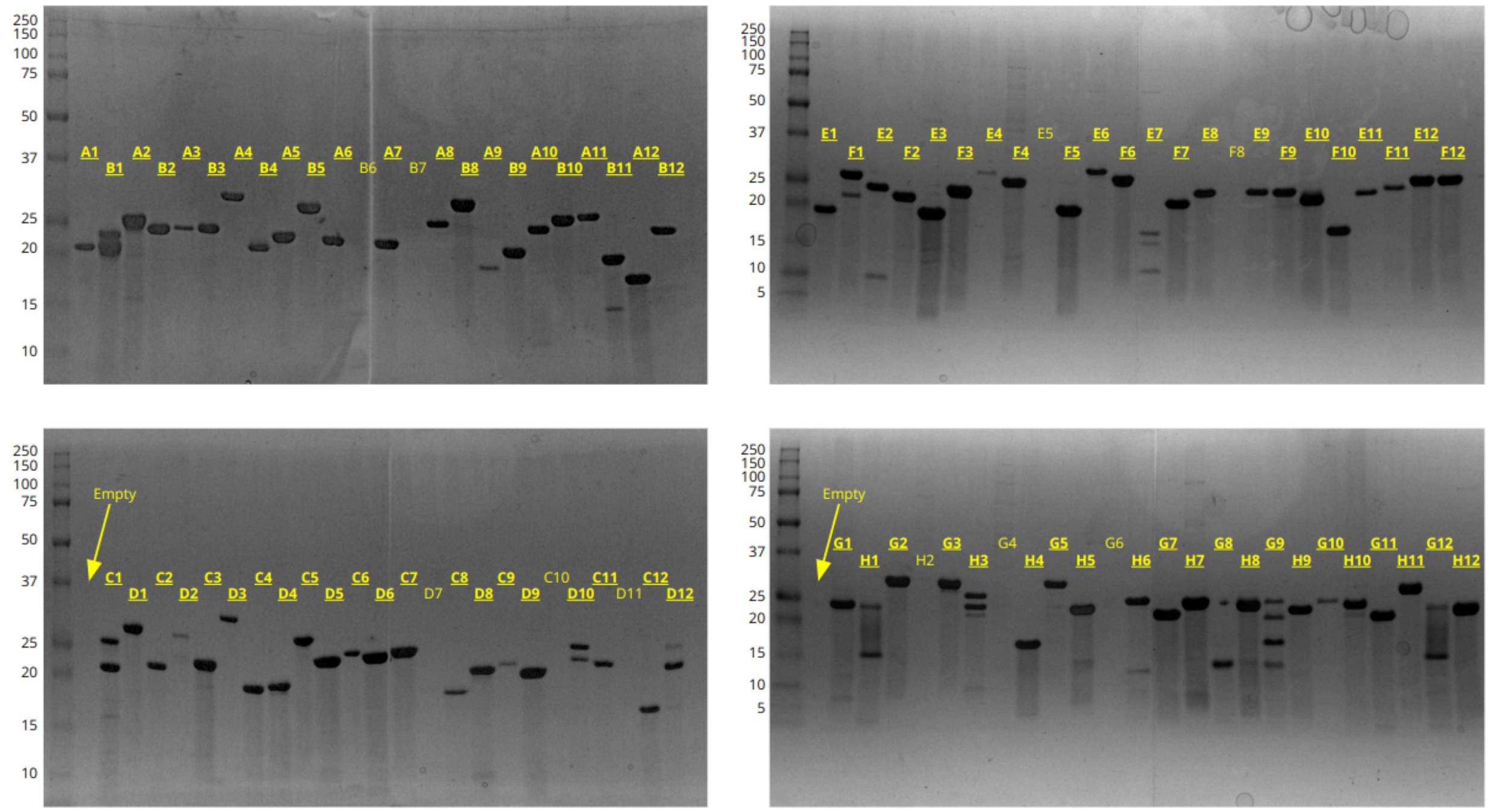

Fig. S4. SDS-PAGE gel results for 96 designs from the first design campaign expressed in *E. coli* and purified by using the Strep-tag affinity chromatography. Designs that had any visible sign of a band were counted as an expressed design (bolded and underlined text). Note that the ladder units are in kDa and that the bottom two gels had an empty spacer well. 86/96 designs show at least one band. For gel source data, see Fig. S27.

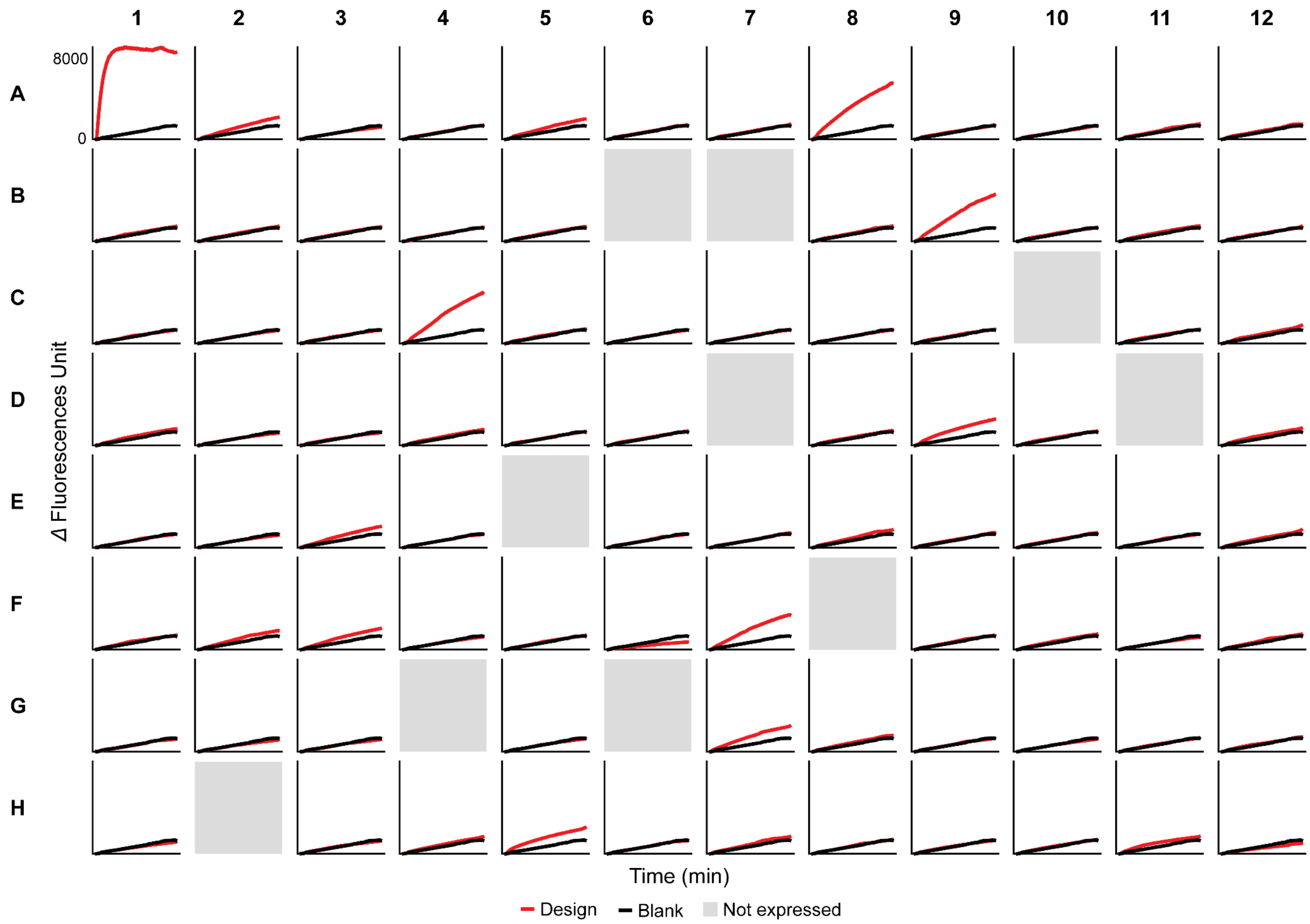

Fig. S5. Initial metallohydrolase activity screen with the purified designs from the first design campaign. The hydrolysis reactions of 9 μM 4MU-PA were monitored by the fluorescence generated from the product over 1 hour. The red progress curves display the reaction in the presence of a purified enzyme (+20 μM added zinc sulfate in the reaction) and the black progress curve overlaid on every plot displays the background reaction in the presence of 20 μM zinc sulfate for comparison. Note that the position of each graph corresponds to its design well position (i.e., top left is design A1) and that the grayed-out plots correspond to design wells that did not express as judged by SDS-PAGE (Fig. S4).

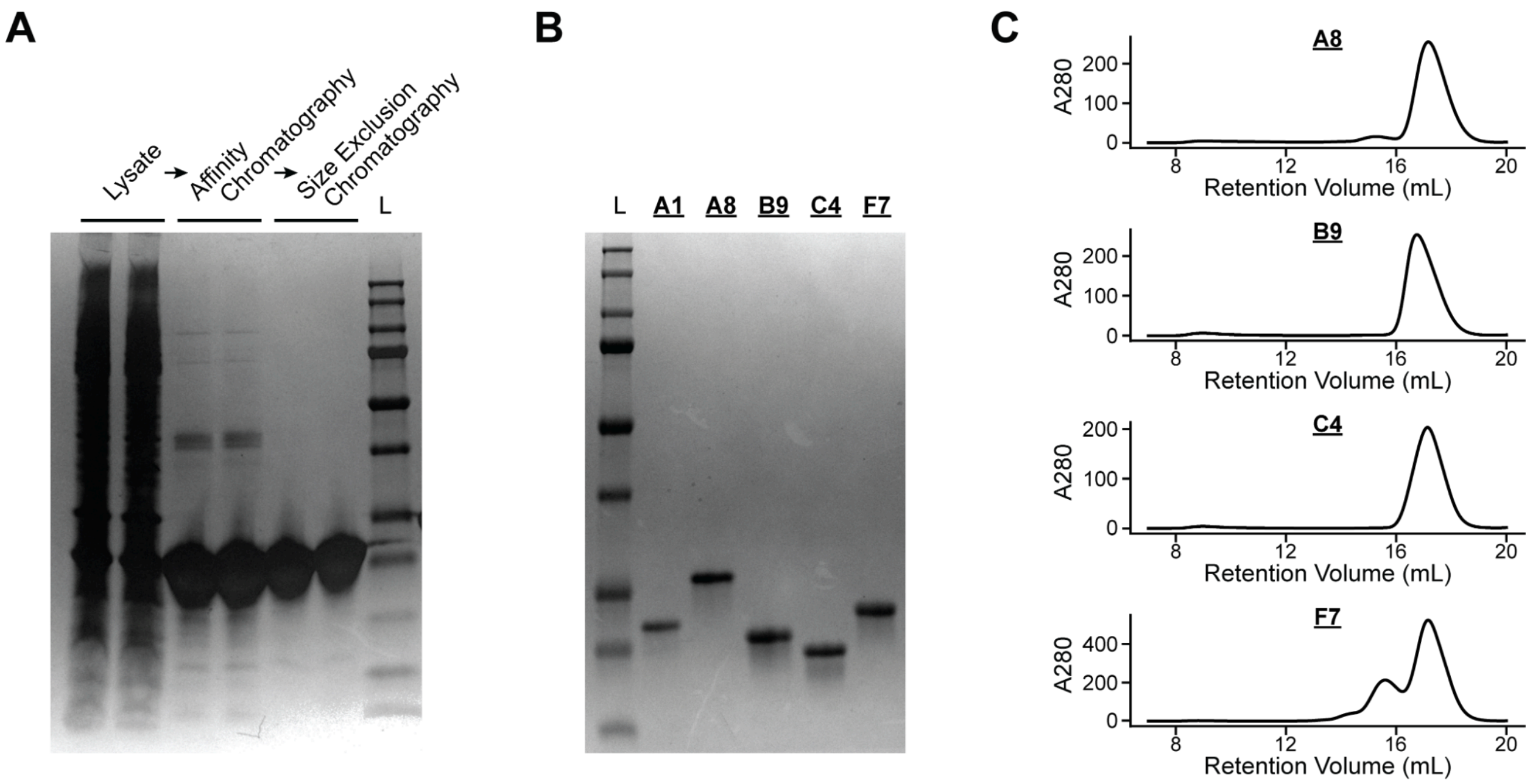

Fig. S6. SDS-PAGE gel results and SEC spectra for the purified hits expressed at scale from the first design campaign. (**A**) Purposefully-overloaded SDS-PAGE gel for the A1 (ZETA_1) design in lysate (left), after affinity chromatography (middle), and after affinity chromatography and size exclusion chromatography (right) with a ladder (far right). Duplicate A1 samples were loaded at each stage. This gel conveys that even with significant protein loading, which should increase the visibility of any contaminants, we are unable to see contaminants after affinity chromatography and size exclusion chromatography, validating the purification protocol and testifying to the high purity of A1 used in further experiments. (**B**) SDS-PAGE gel for the 5 screening hits purified by affinity chromatography and size exclusion chromatography. Note that no contaminant bands are visible and all of the bands are around their expected mass as judged by the ladder position. (**C**) Size exclusion chromatography A280 traces during elution. Most proteins obtain a clean monomeric peak except F7, which appears to have a slight tendency to oligomerize. Only the fraction corresponding to the highest peak in the monomeric state was used for each design. The size exclusion chromatography A280 trace of A1 can be found in the main text (Fig. 3c). For gel source data, see Fig. S27.

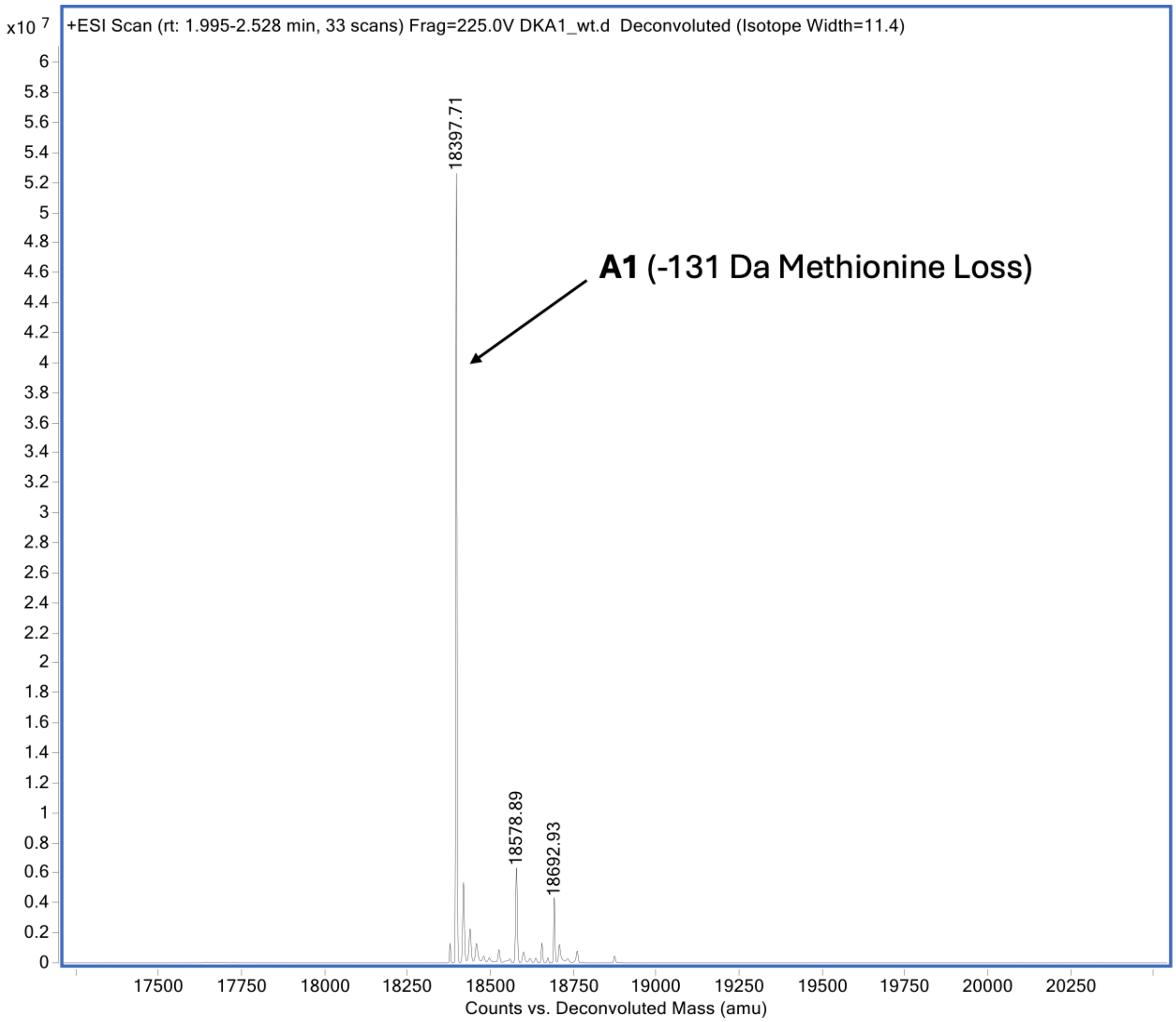

Fig. S7. Electrospray ionization mass spectrometry for A1 (ZETA_1). The expected molecular weight is 18528 Da. The highest peak is approximately 18397 Da, -131 Da from A1 corresponding to the loss of N-terminal methionine (-131 Da). All ESI-MS values are given in Table S1.

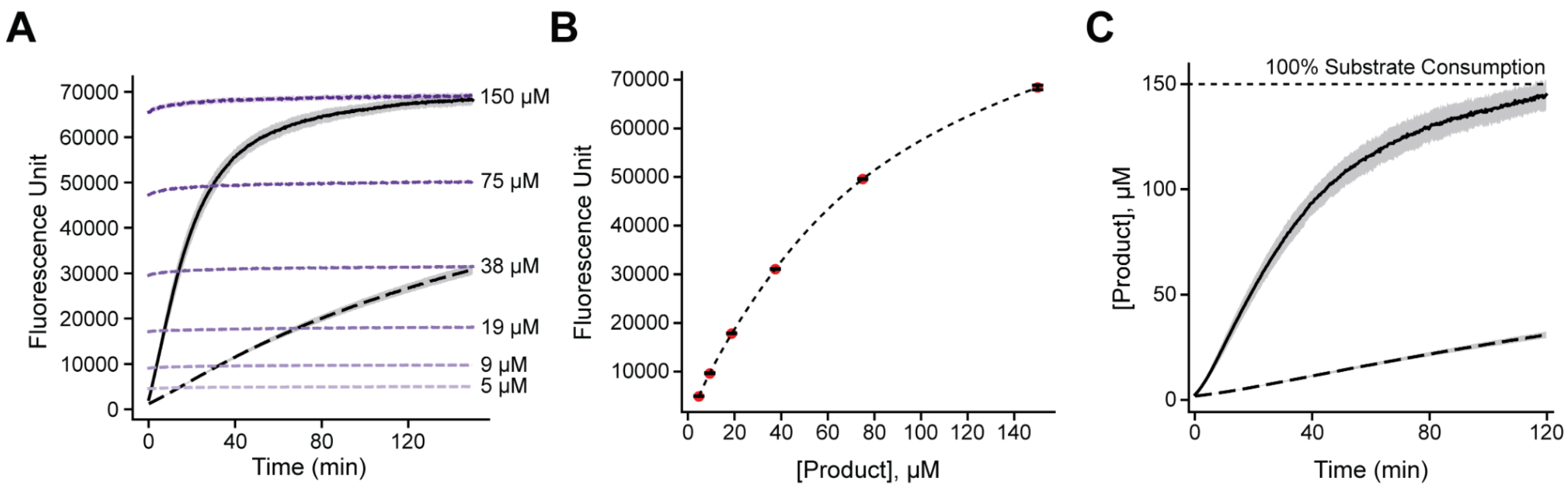

Fig. S8. Total turnover lower bound experiment for A1 (ZETA_1) and calibration curve. (**A**) Fluorescent progress curves for the A1-catalyzed reaction (solid black curve), background reaction with zinc (dotted black curve), and 4MU product calibration standards at labeled concentrations (rainbow lines). (**B**) Calibration curve from the 4MU product standards for the turnover experiments with a fitted 4th order polynomial regression curve for interpolation. Note that the calibration curves used for all other experiments were in the linear range and fitted with linear regression; this experiment was an exception because it required measurements of high fluorescence corresponding to highly concentrated 4MU product, which displays nonlinear behavior likely due to fluorescence self-quenching at high concentrations. The interpolation does not affect the final results of this experiment (i.e., A1 catalyzes >1000 turnovers). (**C**) A1-catalyzed reaction and background reaction with 4 μM zinc plotted in terms of product concentration after using the calibration curve to convert the fluorescence signal into product concentration. The A1 enzyme was present at 100 nM and all reactions used 150 μM of 4MU-PA substrate; enzyme turnover number is calculated by the ratio of [product]:[enzyme] after subtracting off the background reaction.

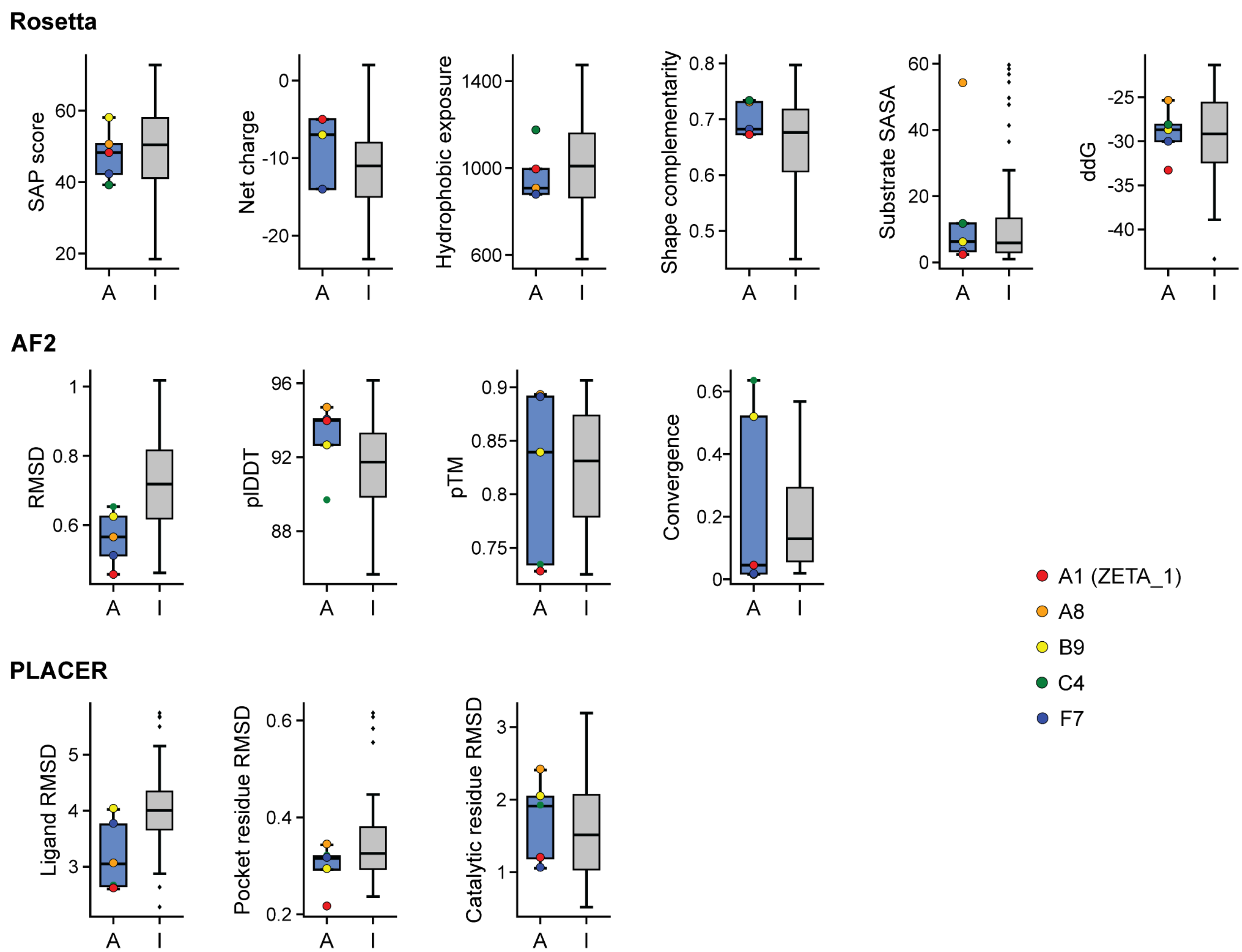

Fig. S9. *In silico* metric distributions for the ordered designs in the first design campaign. 'A' represents the computational metric distribution for the 5 'Active' designs (A1, A8, B9, C4, F7) and 'I' represents the computational metric distribution for the other 91 'Inactive' designs, including the 10 designs that did not express. These plots compare the distributions for common and insightful metrics from Rosetta, AlphaFold2, and PLACER between the 5 active and 91 inactive designs. The box plot shows the five-quartile summary and the identified outliers are shown as small black dots for the inactive designs. The data points for the 5 active designs are shown as colored dots in each metric with the corresponding color legend shown on the right. Note that Fig. S22 is a greater extension of this analysis, including more metrics and Chai-1 metrics.

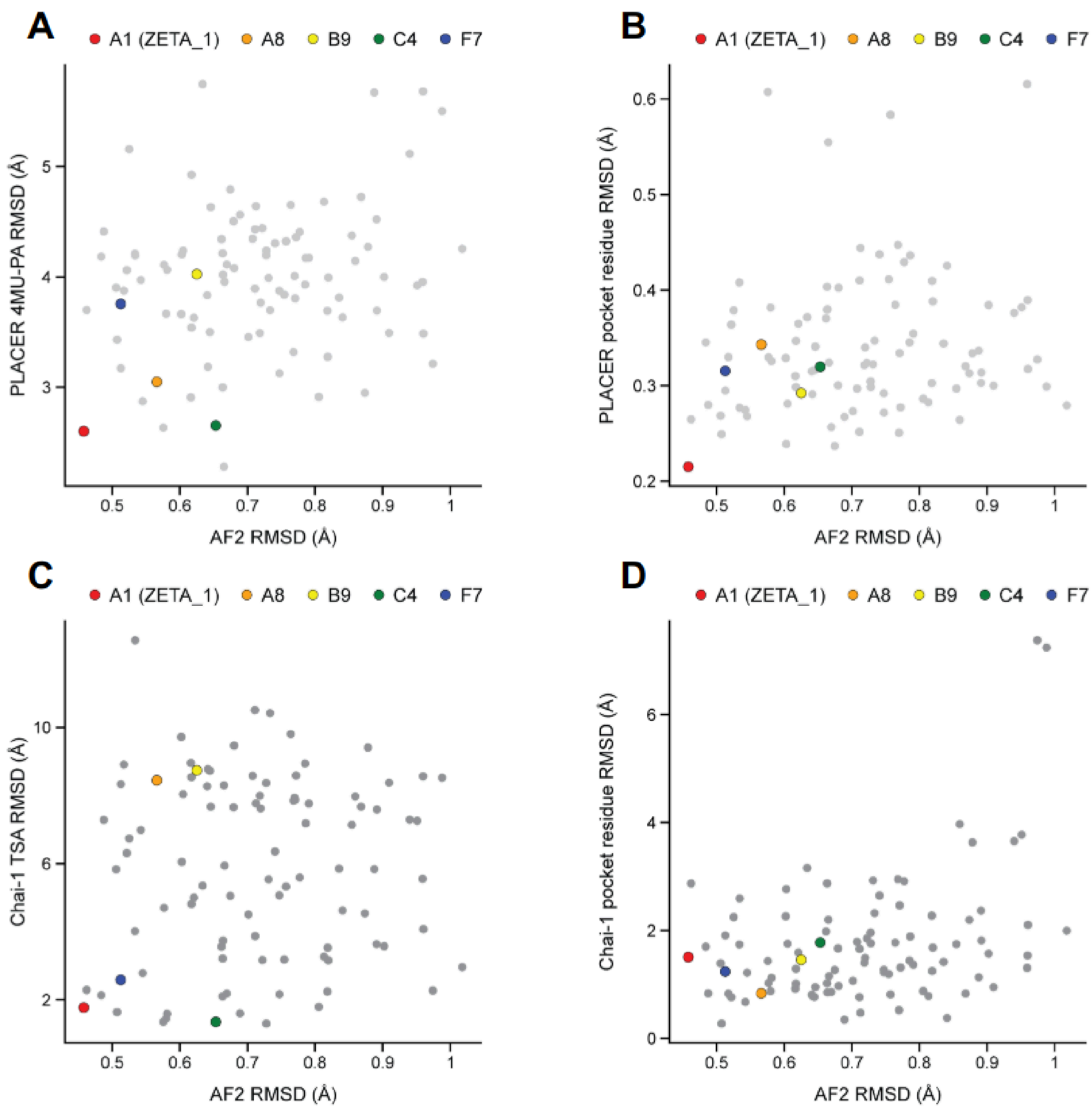

Fig. S10. Comparison of AlphaFold2 metrics with PLACER metrics and Chai-1 for ordered designs (1st design campaign). (**A**) Average substrate RMSD between the design model and the predicted PLACER ensembles (PLACER 4MU-PA RMSD) vs the global C_α RMSD between the design model and the AlphaFold2 prediction (AF2 RMSD). (**B**) Average active site residue RMSD between the design model and the predicted PLACER ensembles (PLACER pocket residue RMSD) vs the AF2 RMSD. (**C**) Average transition state phosphoanalog (TSA) RMSD between the design model and the predicted Chai-1 ensembles with Zn(II) and the TSA vs the AF2 RMSD; the TSA is a good way to predict with something that mimics the transition state (see Fig. S15). (**D**) Average protein pocket residue RMSD between the design model and the predicted Chai-1 ensembles with Zn(II) and the TSA vs the AF2 RMSD. Note that the bottom left corner is ideal in all plots, indicating high agreement between the design and predictions. Interestingly, A1 (ZETA_1) is the closest to the bottom left in 3/4 of these plots, suggesting that high agreement between AlphaFold2/PLACER/Chai-1 may be useful for predicting activity.

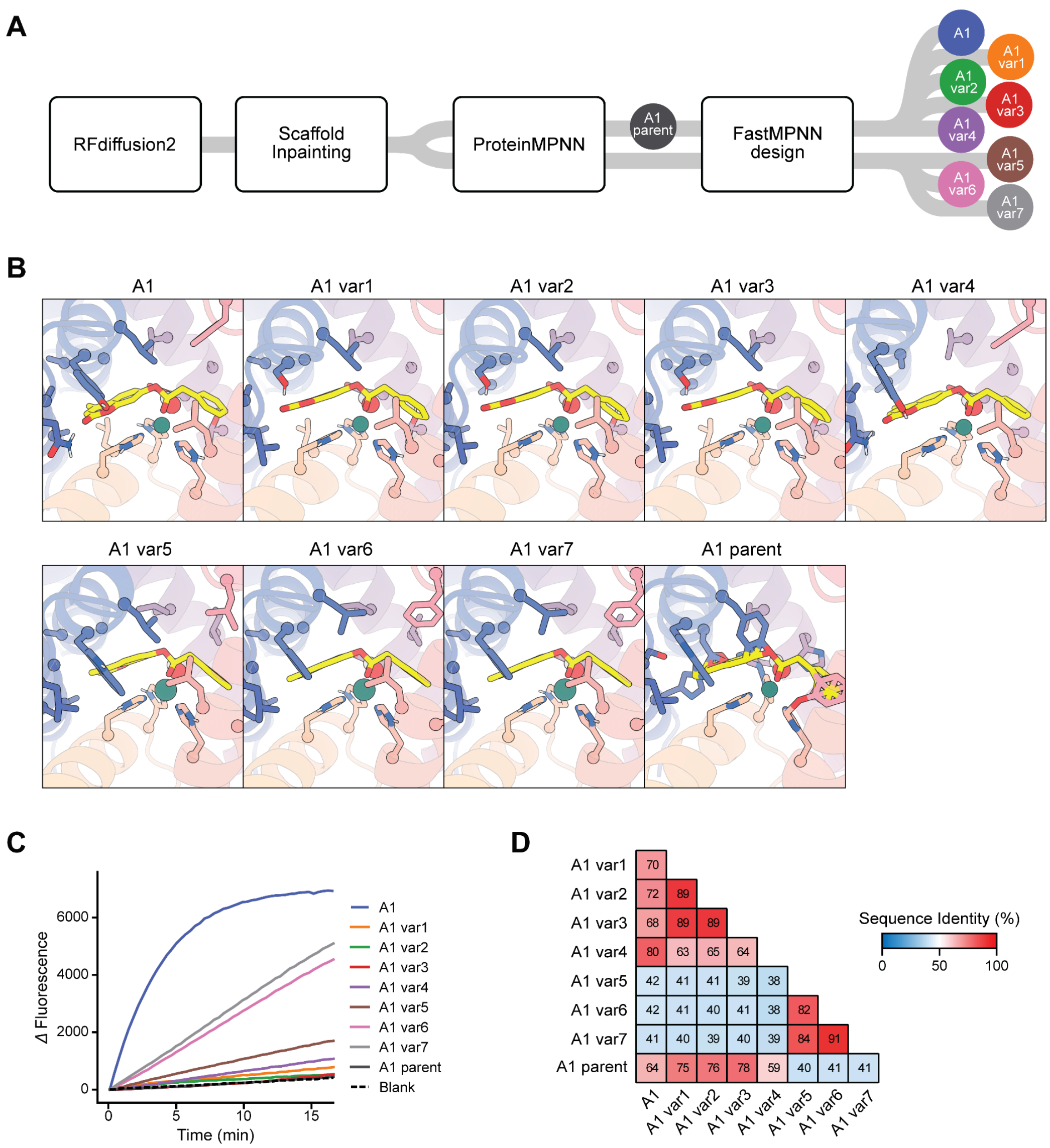

	A1	A1 var1	A1 var2	A1 var3	A1 var4	A1 var5	A1 var6	A1 var7
A1 var1	70							
A1 var2	72	89						
A1 var3	68	89	89					
A1 var4	80	63	65	64				
A1 var5	42	41	41	39	38			
A1 var6	42	41	40	41	38	82		
A1 var7	41	40	39	40	39	84	91	
A1 parent	64	75	76	78	59	40	41	41

Fig. S11. Computational and experimental comparison of A1 (ZETA_1) against other sequences identified for that backbone during design. (**A**) High-level computational pipeline overview visualizing the conception of A1, the parent A1, and 7 variants. Note that all variants came from the same RFdiffusion2 scaffold but variants 5-7 came from a separate resample. (**B**) Comparison of the active sites between A1 and its 7 sequence variants that made it to the final stages of computational filtering. The “A1 parent” is the originally folded sequence from ProteinMPNN, which is agnostic of ligands and metals, before the active site optimization step with iterative LigandMPNN and Rosetta design, hence the clashes. (**C**) Ester hydrolase activity assay comparing A1 with its 7 variants and parent sequence demonstrating that A1 has superior activity. This provides confidence in our *in silico* filtering method, which was able to successfully identify the best sequence for the A1 backbone for the 96-design order with 96 unique backbones (1 sequence / scaffold). (**D**) Heatmap comparing the sequence similarity between A1, the A1 parent, and all of the A1 variants. Shades of blue represent lower sequence identity (<50%) between two designs and shades of red represent higher sequence identity (>50%) between two designs. Variant 4 is the most similar in sequence to A1 but still only shares 80% sequence identity, showing that this pipeline explores a highly diverse sequence space within each scaffold during design.

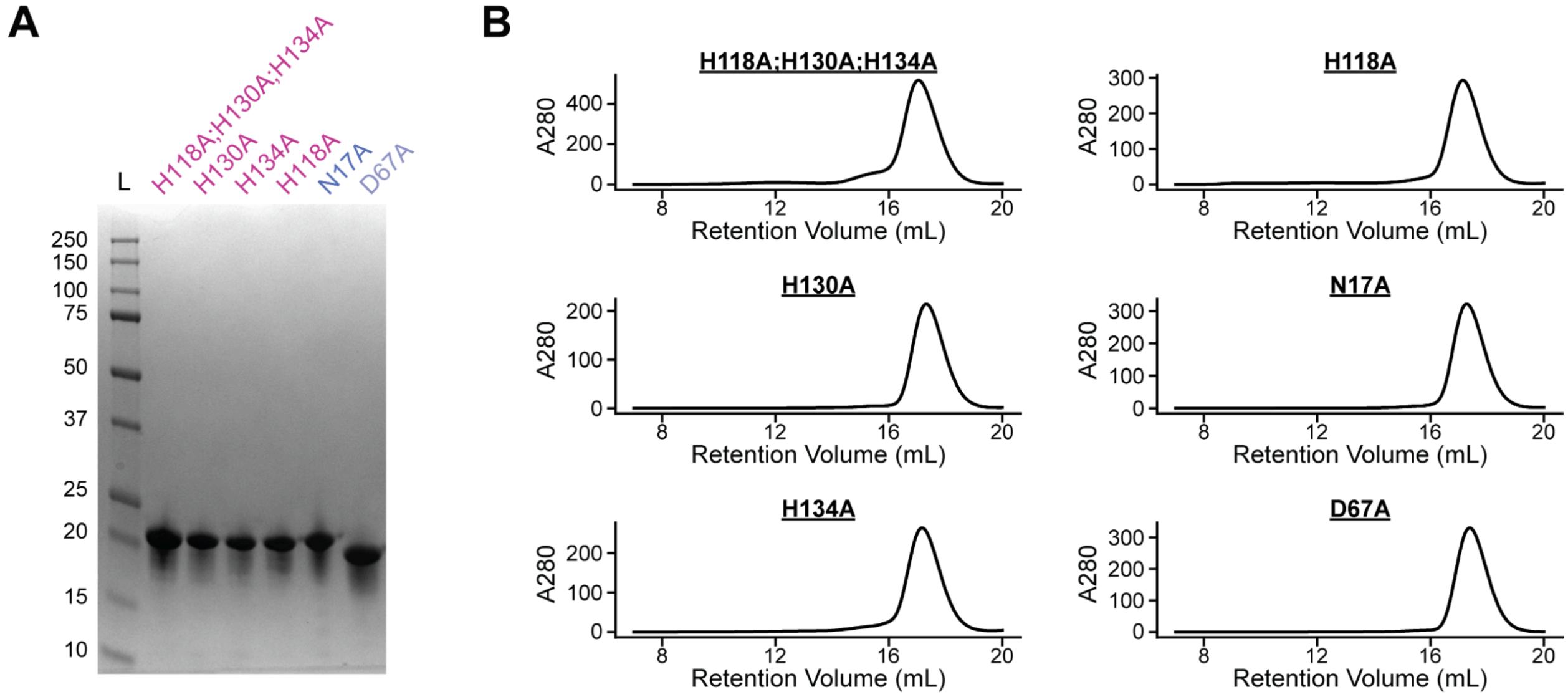

Fig. S12. SDS-PAGE gel results and SEC spectra for the purified A1 (ZETA_1) knockout mutants expressed at scale. (**A**) SDS-PAGE gel for the six A1 mutants purified by affinity chromatography and size exclusion chromatography, respectively. Note that no contaminant bands are visible and all of the bands are around their expected mass as judged by the ladder position. (**B**) Size exclusion chromatography A280 traces during elution. All of the mutants demonstrate a clean monomeric peak. Only the fraction corresponding to the highest peak in the monomeric state was used for each design. For gel source data, see Fig. S27.

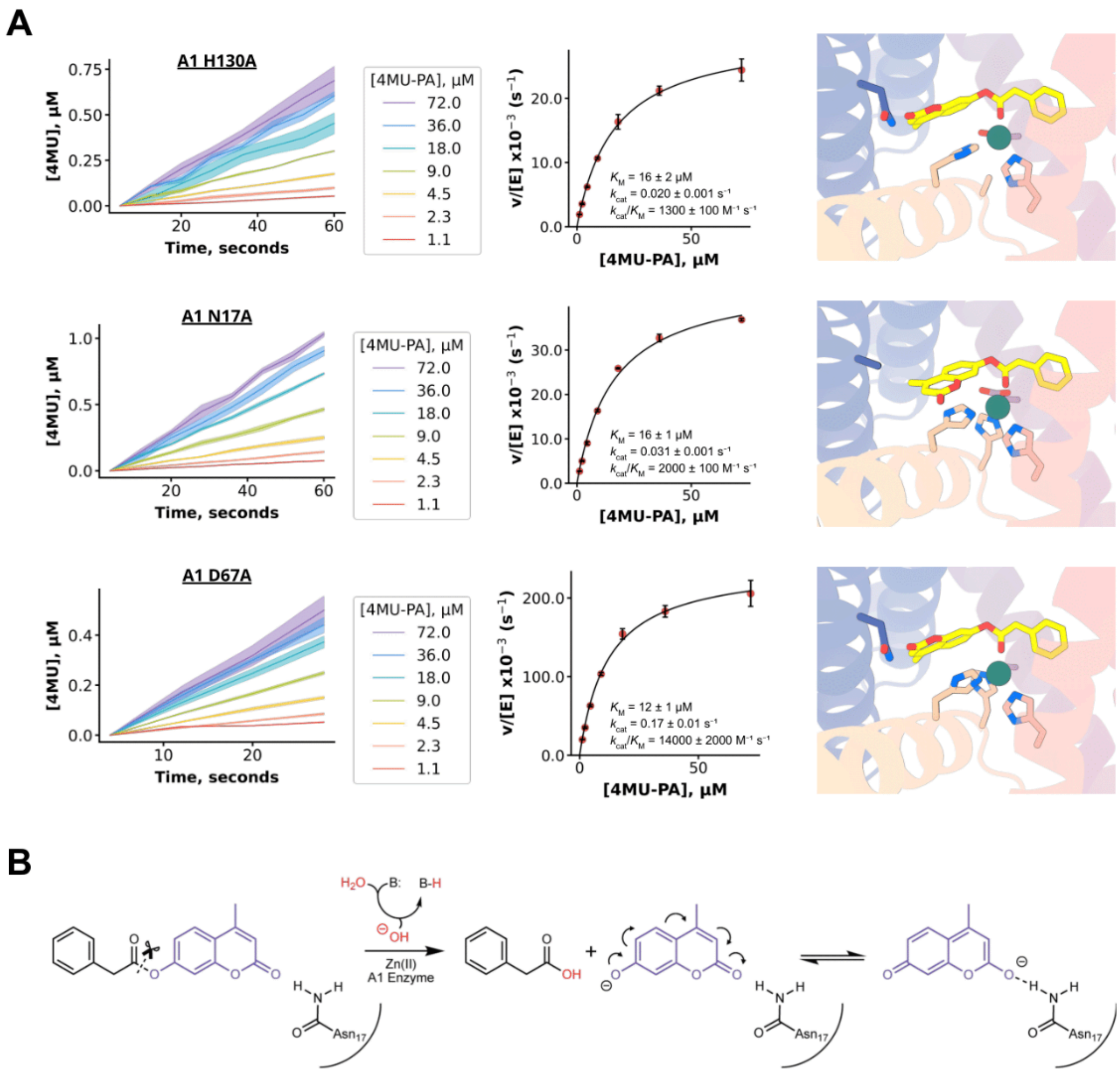

Fig. S13. Kinetic characterization and Chai-1[13] predicted structures of the A1 (ZETA_1) knockout mutants with the proposed role of residue N17. (**A**) The progress curves for the measured initial velocities are shown on the left. Michaelis-Menten plots from these data are shown in the middle. The Chai-1 predicted structures and predicted complexes of the protein, zinc, and substrate are shown on the right for each mutant. Chai-1 predicts the zinc and substrate in almost the exact same position and geometry as the A1 design model for each of the active mutants. Additionally, Chai-1 predicts the D67 coordination to zinc in the H130A mutant as hypothesized. Interestingly, the N17A substrate docking position by Chai-1 reveals a slightly shifted coumarin (4MU) conformation, suggesting N17 likely plays a role in positioning the coumarin and possibly a role in stabilizing the coumarin leaving group. (**B**) Proposed stabilization of the coumarin leaving group through resonance and H-bonding to N17.

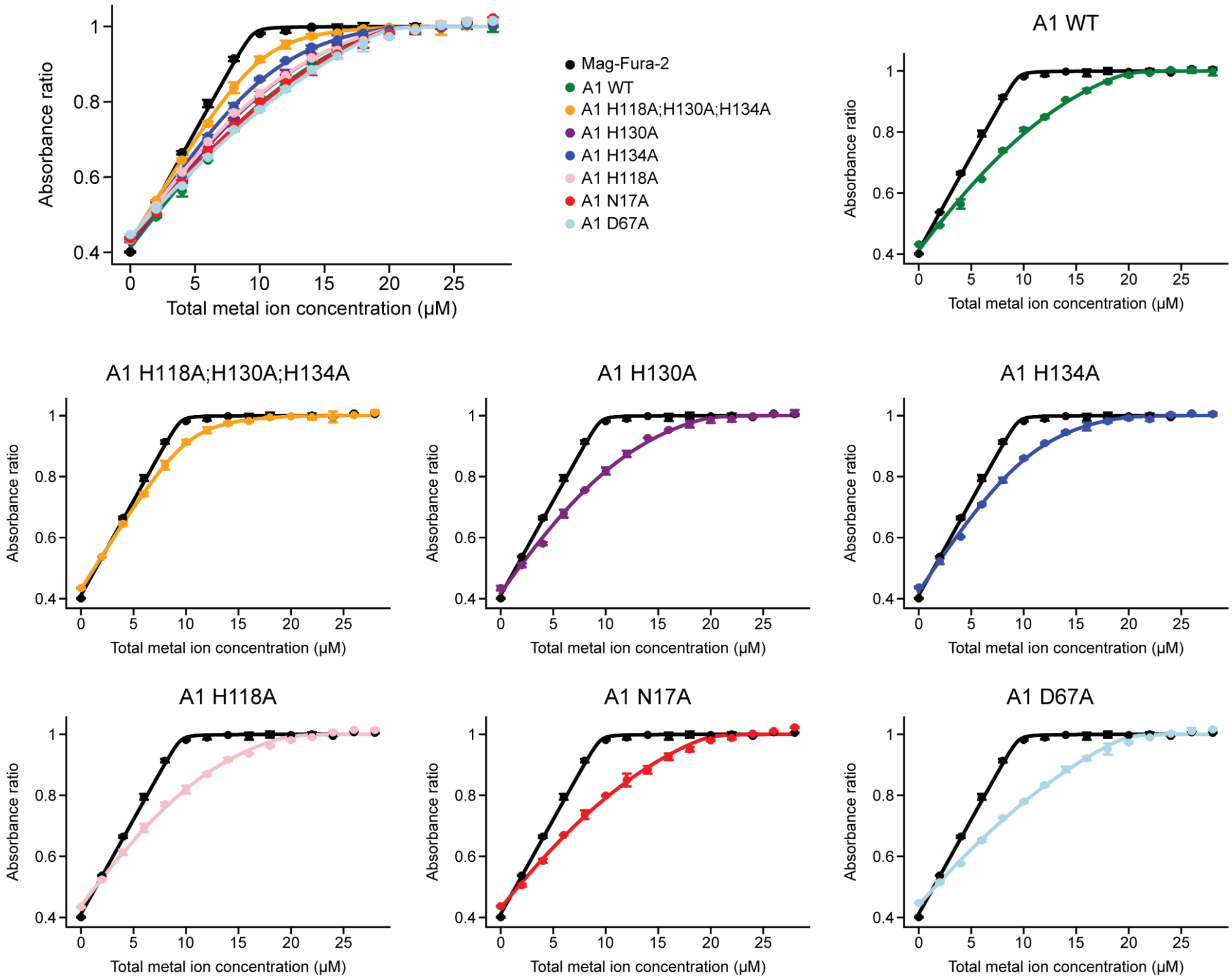

Fig. S14. Zinc-binding affinity experiments for A1 (ZETA_1) and its knockout mutants. The plots display the zinc-binding profiles of the wild-type A1 enzyme and its mutants. As the total zinc ion concentration increases, the absorbance ratio plateaus reflecting saturated zinc binding.

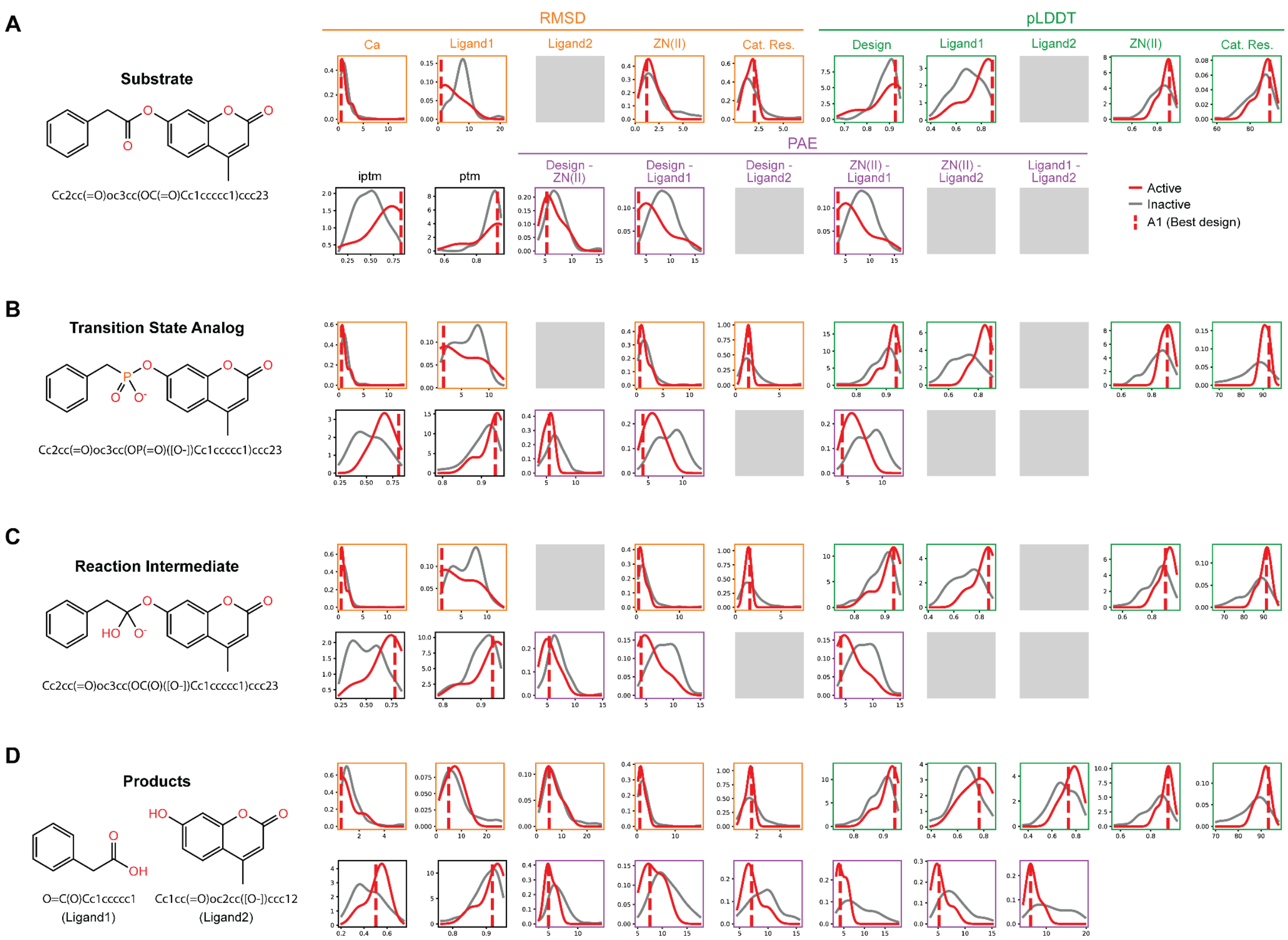

Fig. S15. Chai-1 prediction distributions with different ligands for the active and inactive designs from the first design campaign. Averaged metrics across five Chai-1 prediction ensembles of the designed protein sequence, one Zn(II) ion, and either (**A**) the substrate, (**B**) the transition state phosphoanalog, (**C**) the tetrahedral reaction intermediate, or (**D**) the two products. The ligand strings are provided for each small molecule used in the prediction. The black line represents the metric distribution for the inactive designs from the first design campaign (91 designs), the red line represents the metric distribution for the active designs from the first design campaign (5 designs), and the dashed red vertical line represents the metric value for ZETA_1 (A1). The metrics are labeled in (A) and organized the same way in the rest figure. There are five main classes of metrics evaluated: RMSD between the design model and predicted model (orange border; lower is better), pLDDT of the predicted model (green border; higher is better), pTM of the prediction and interface pTM (ipTM) of the prediction (black borders; higher is better), and PAE of the prediction (purple border; lower is better). Note that (D) has 2 small molecules corresponding to the two products from the hydrolyzed substrate, these positions are grayed out in (A-C). We believe that predicting with the transition state analog is the most rational decision (see section 4.2). This informed the second design campaign filtering, which actively used Chai-1 for filtering rather than retrospective analysis.

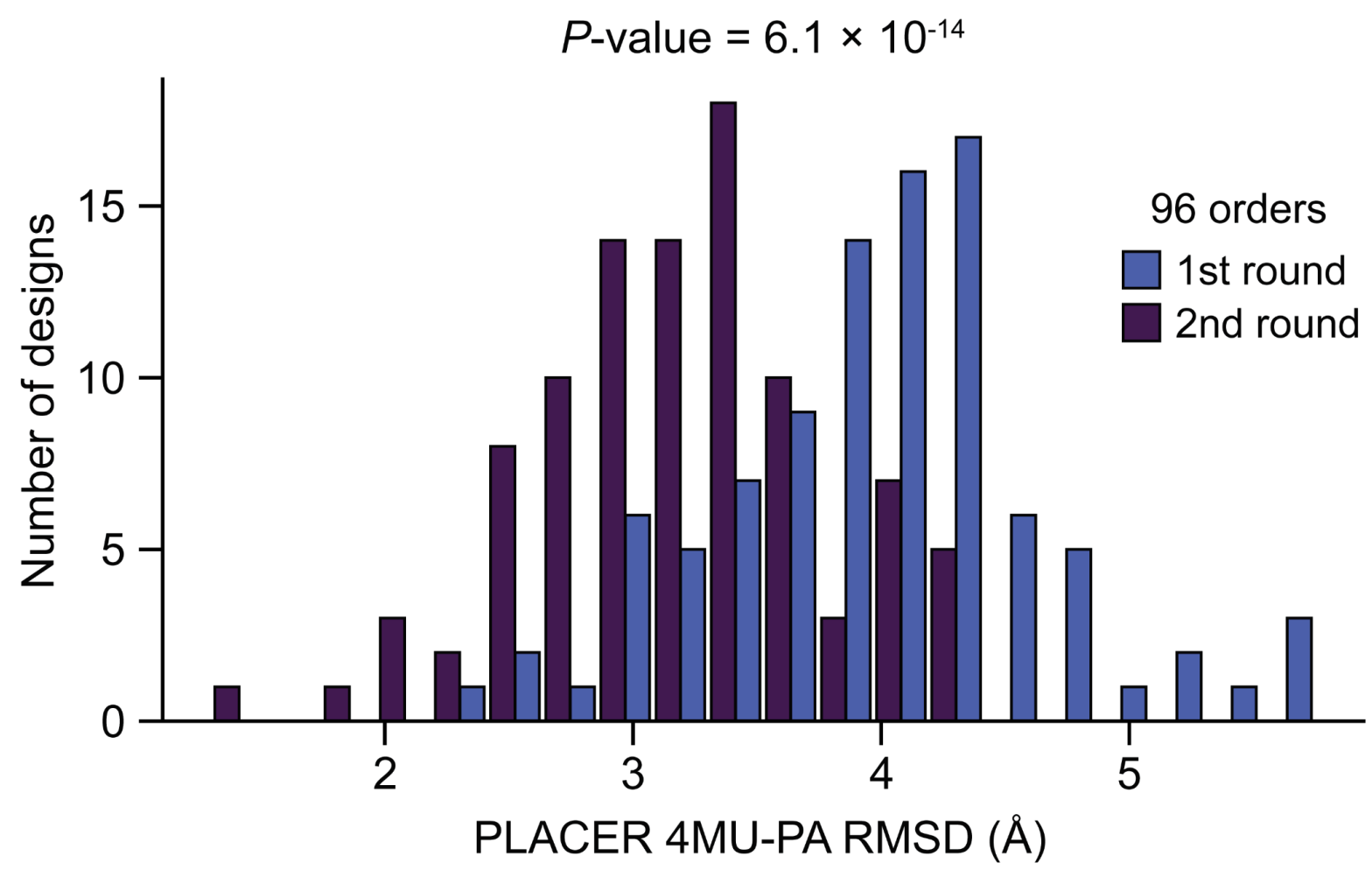

Fig. S16. PLACER metrics of ordered designs from both design campaigns. Histogram comparing the distribution of average substrate RMSD between the design model and the predicted PLACER ensembles (PLACER 4MU-PA RMSD) for the 96 designs ordered from the 1st design campaign (blue) and the 96 designs ordered from the 2nd design campaign (purple). The significance was measured using a two-sided Wilcoxon rank-sum test, a nonparametric statistical test. The effect size was negative (rank-biserial correlation = -0.63), indicating that PLACER metrics from the second round tended to be lower than those from the first round. The 2nd design campaign featured designs whose substrate positions were predicted by PLACER to be in higher agreement with their design model (lower RMSD), on average.

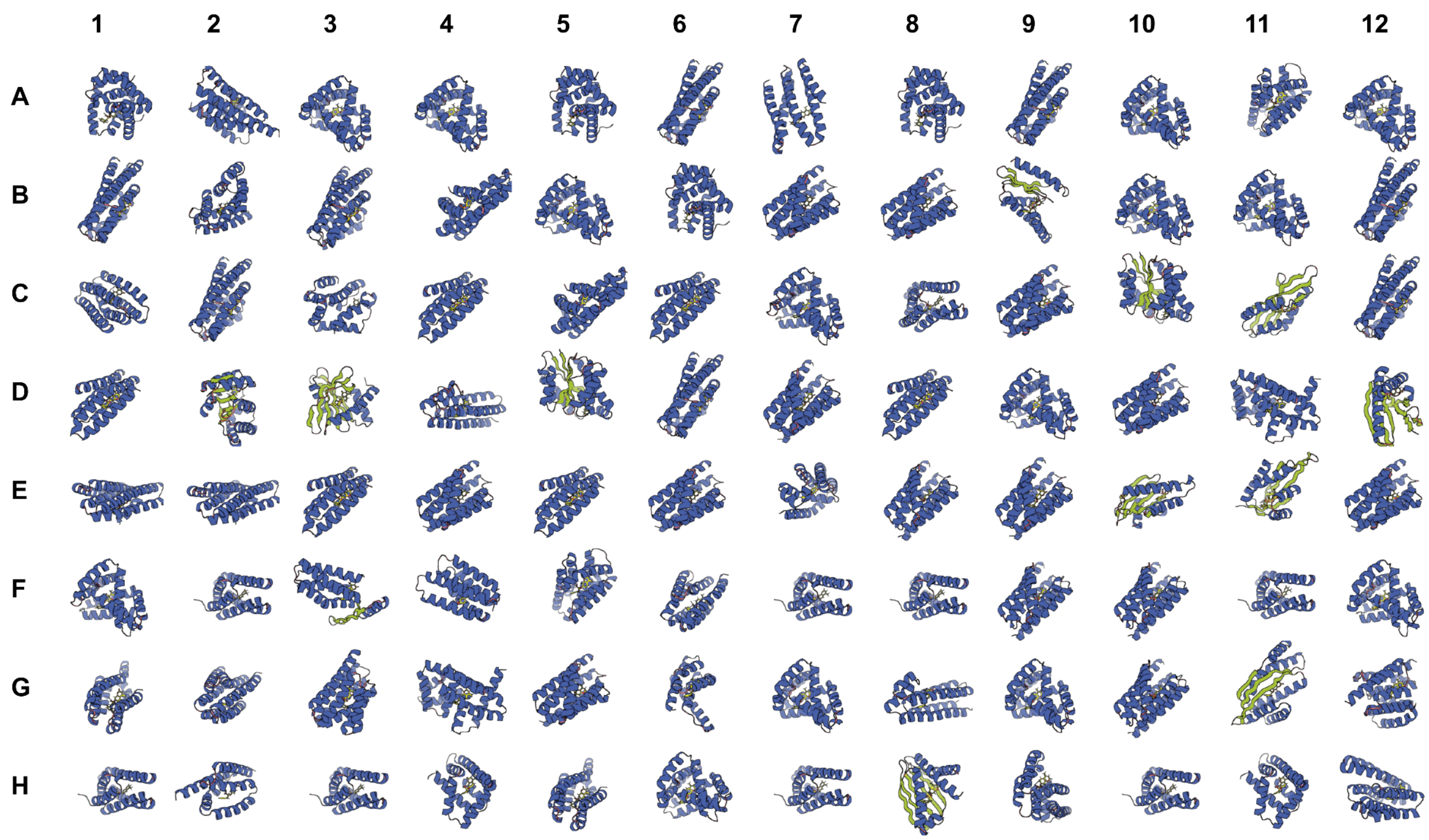

Fig. S17. Structural panel of the 96 ordered designs spanning 37 unique backbones from the second design campaign. The secondary structures are colored (green = beta-sheets; blue = alpha-helices). Structures range in length and have a range of diverse folds. Scaffolds were generated in the length range of 110 - 190 amino acids.

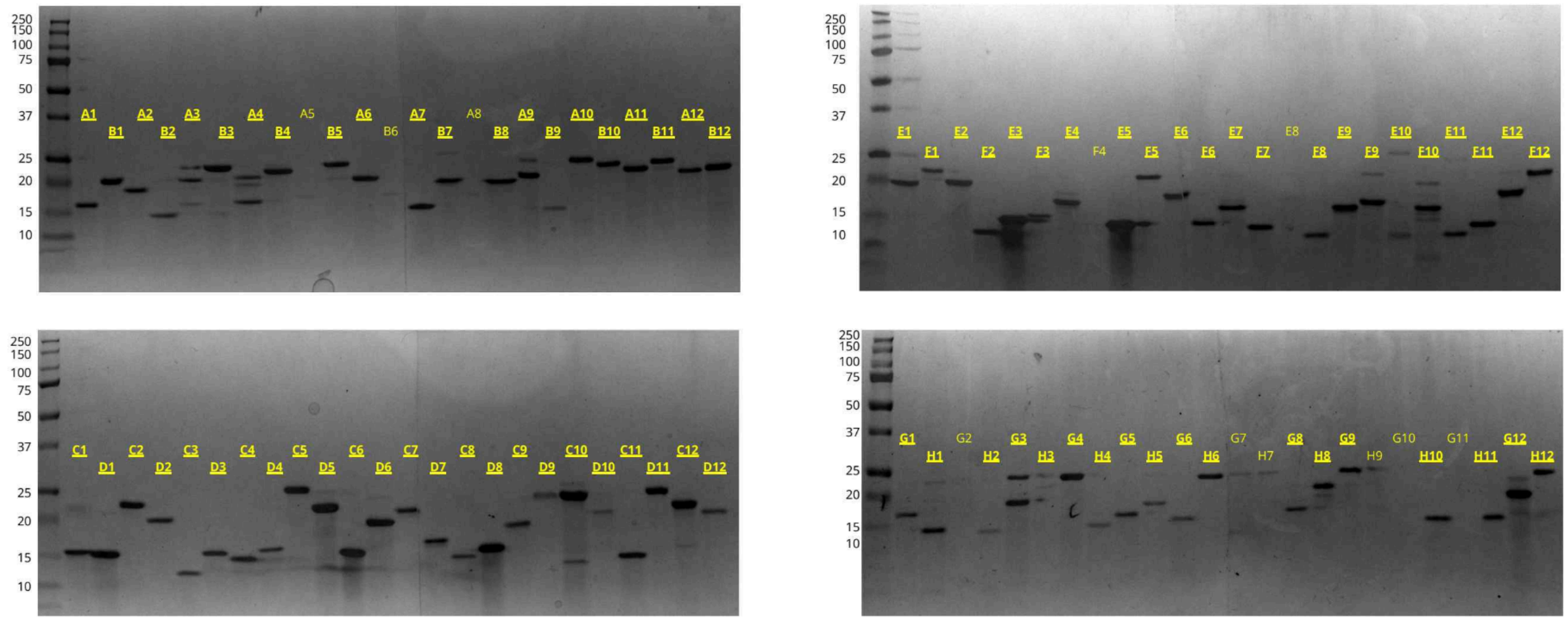

Fig. S18. SDS-PAGE gel results for the 96 designs of the second round campaign expressed in *E. coli* and purified in parallel using Strep-tag affinity chromatography. Designs that had any visible sign of a band were counted as an expressed design (bolded and underlined text). Note that the ladder units are in kDa and that the bottom two gels had an empty spacer well. 85/96 designs show at least one band. For gel source data, see Fig. S27.

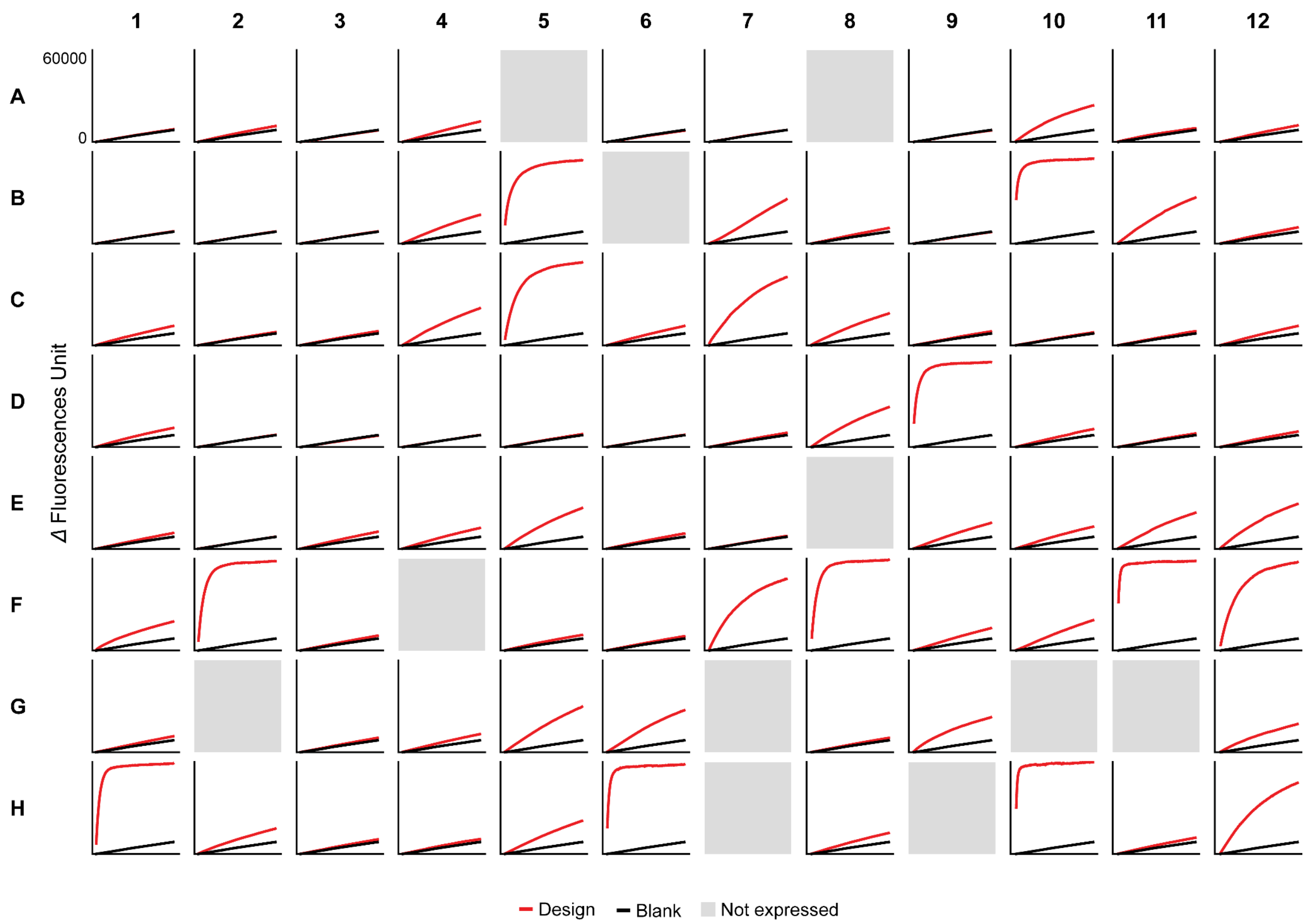

Fig. S19. Initial metallohydrolase activity screen with the Strep-tag affinity chromatography purified designs for the second design campaign. The hydrolysis reactions of 100 μM 4MU-PA were monitored by the fluorescence generated from the product over 1 hour. The red progress curves display the reaction in the presence of a purified enzyme (+10 μM added zinc sulfate in the reaction) and the black progress curve overlaid on every plot displays the background reaction in the presence of 10 μM zinc sulfate for comparison. Note that the position of each graph corresponds to its design well position (i.e., bottom right is design H12) and that the grayed-out plots correspond to design wells that did not express as judged by SDS-PAGE (Fig. S18).

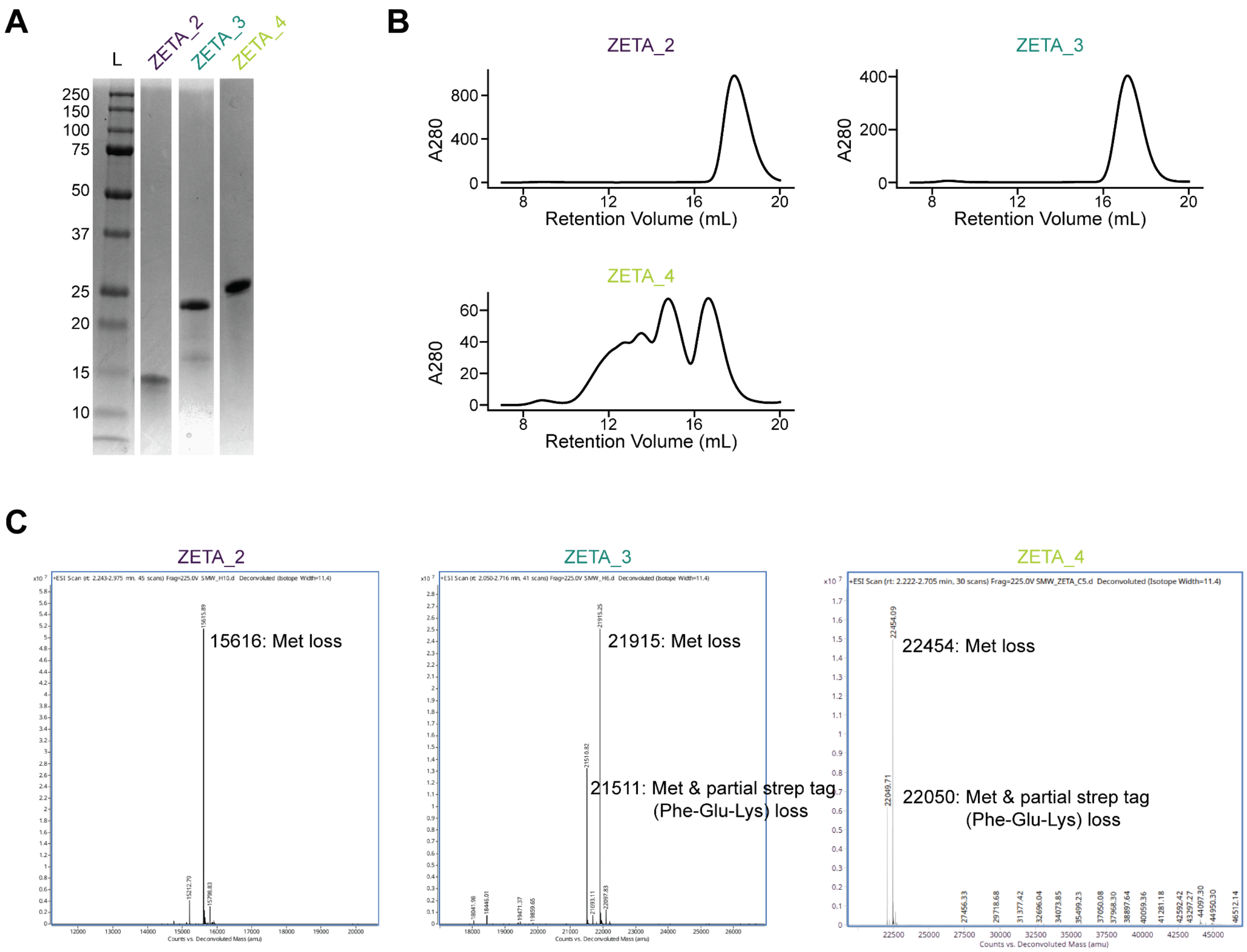

Fig. S20. SDS-PAGE gel results and SEC chromatograms and ESI mass spectrometry plots of ZETA_2-4. (**A**) SDS-PAGE gel for ZETA_2-4 purified by Strep-tag affinity chromatography followed by size exclusion chromatography. All of the bands are around their expected mass as judged by the ladder position. (**B**) Size-exclusion chromatography (SEC) A280 traces during elution using the Superdex 200 Increase 10/300 GL column. ZETA_2 and ZETA_3 obtain clean, monomeric peaks but ZETA_4 appears to have a tendency to dimerize or oligomerize. Only the fraction corresponding to the highest peak in the monomeric state was used for each design. (**C**) Electrospray ionization (ESI) mass spectrometry (MS) for ZETA_2-4. Their observed molecular weights are labeled and identified. N-terminal Met loss (-131 Da) was observed in all cases as the major peak. Interestingly, ZETA_3 and ZETA_4 also had major peaks corresponding to N-terminal Met loss + C-terminal Phe-Glu-Lys loss from the Strep-tag (-131 + -404 = -535 Da loss). Although not formally investigated or shown, we observed the -535 Da loss peak become larger if we let ZETA_3-4 sit for longer after purification before ESI-MS; given the designed hydrolytic function for 4MU-PA, which contains a -CH2-phenyl group like that of Phe (adjacent to where the peptide cleavage happens), we suspect that ZETA_3-4 may have some promiscuous protease activity, specifically for Phe-adjacent amides. All ESI-MS values are given in Table S1 with the expected masses. For gel source data, see Fig. S27.

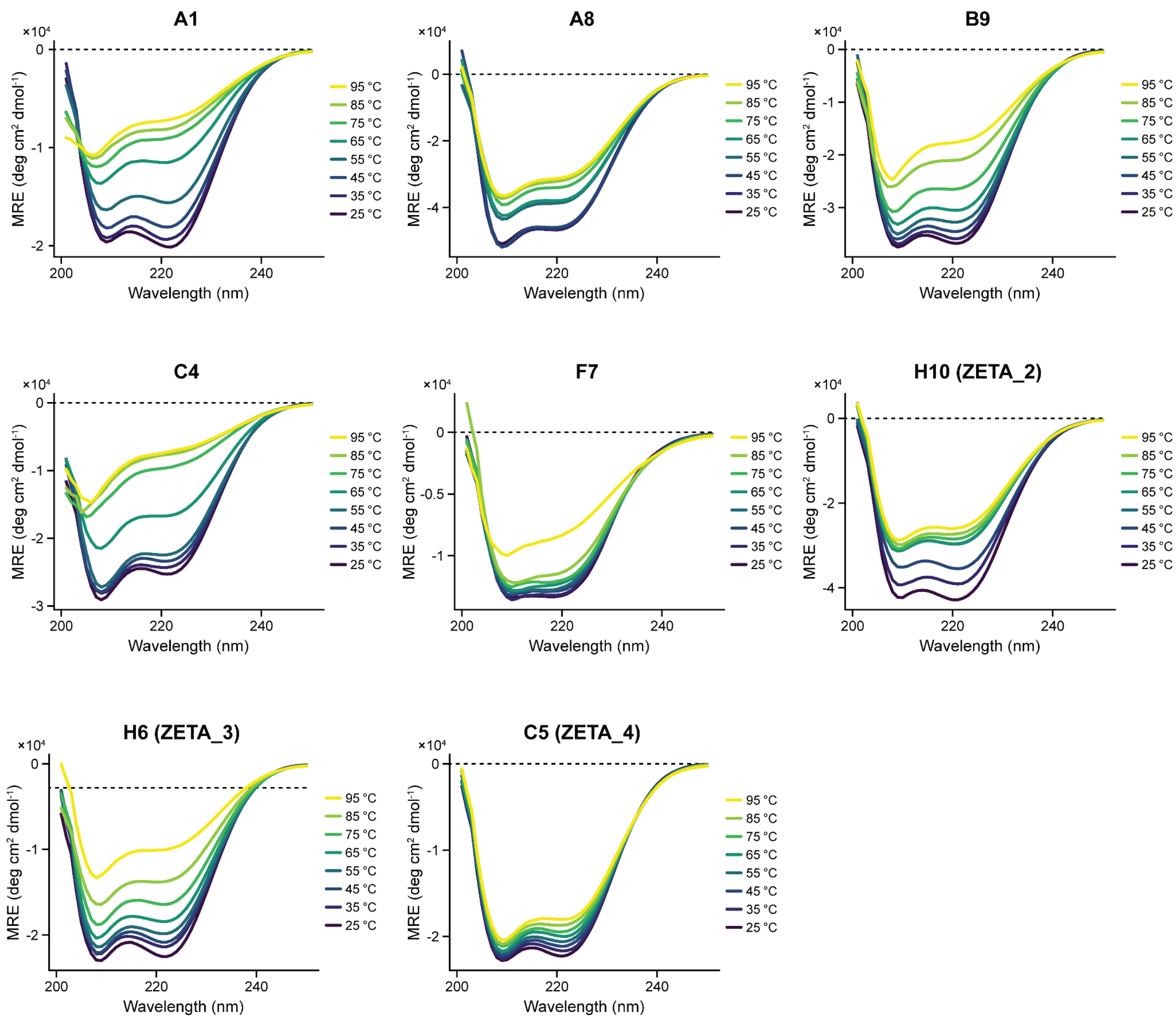

Fig. S21. Circular dichroism spectra for all characterized enzymes at increasing temperatures. A1 (ZETA_1), A8, B9, C4, and F7 are from the first design campaign. ZETA_2-4 are from the second design campaign. The CD spectra were recorded in 10 °C increments from 25 °C to 95 °C. In all cases, the CD spectra are consistent with the expected secondary structure from the design models, which is alpha-helical in most cases. The enzymes were assumed to be purified and analyzed in their zinc-bound states.

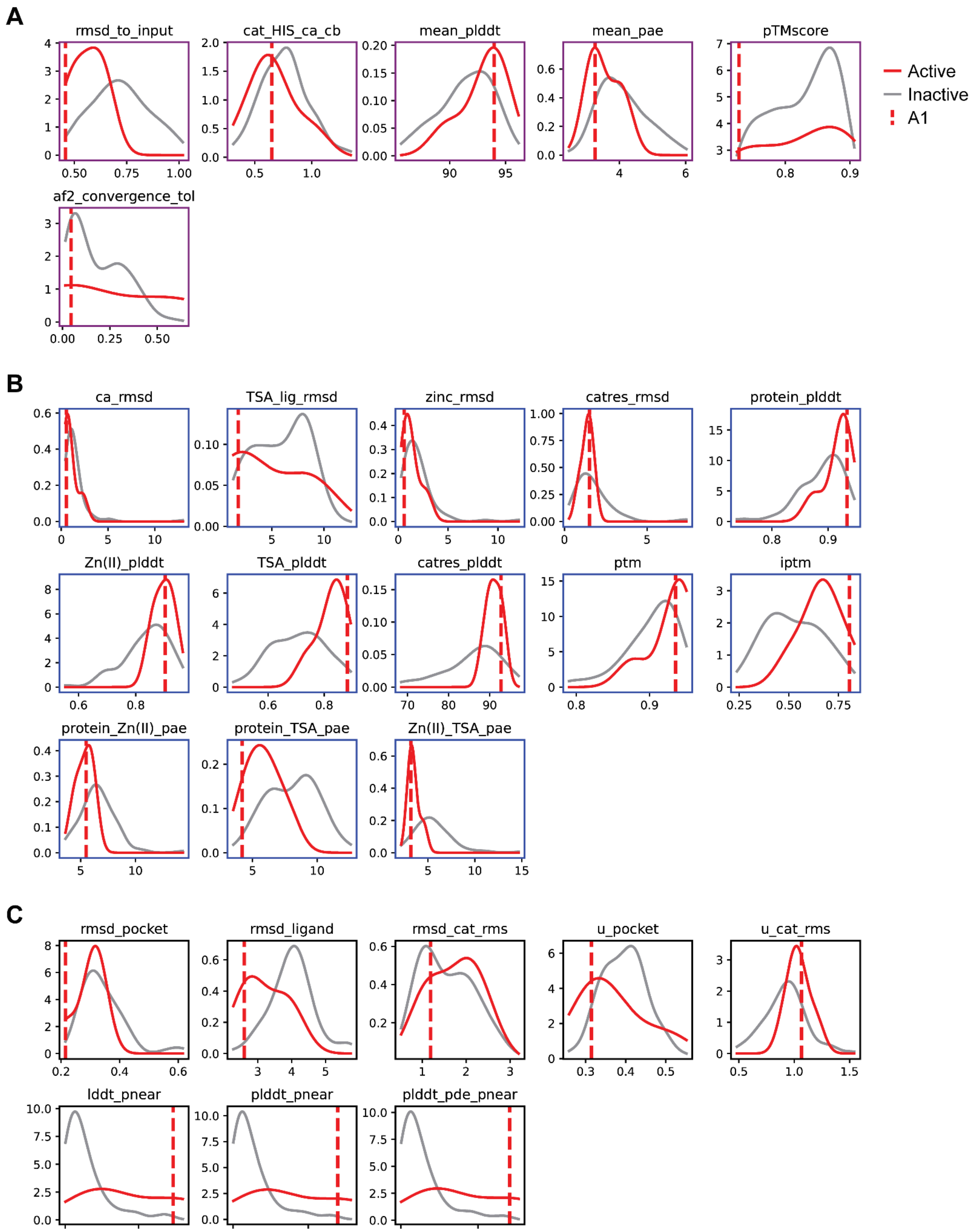

Fig. S22. AF2/Chai-1/PLACER metric distributions for first design campaign. (**A**) AlphaFold2, (**B**) Chai-1, and (**C**) PLACER metric distributions. Gray line is the distribution for the 91 inactive designs. Red solid line is the distribution for the 5 active designs. Red dashed line is ZETA_1.

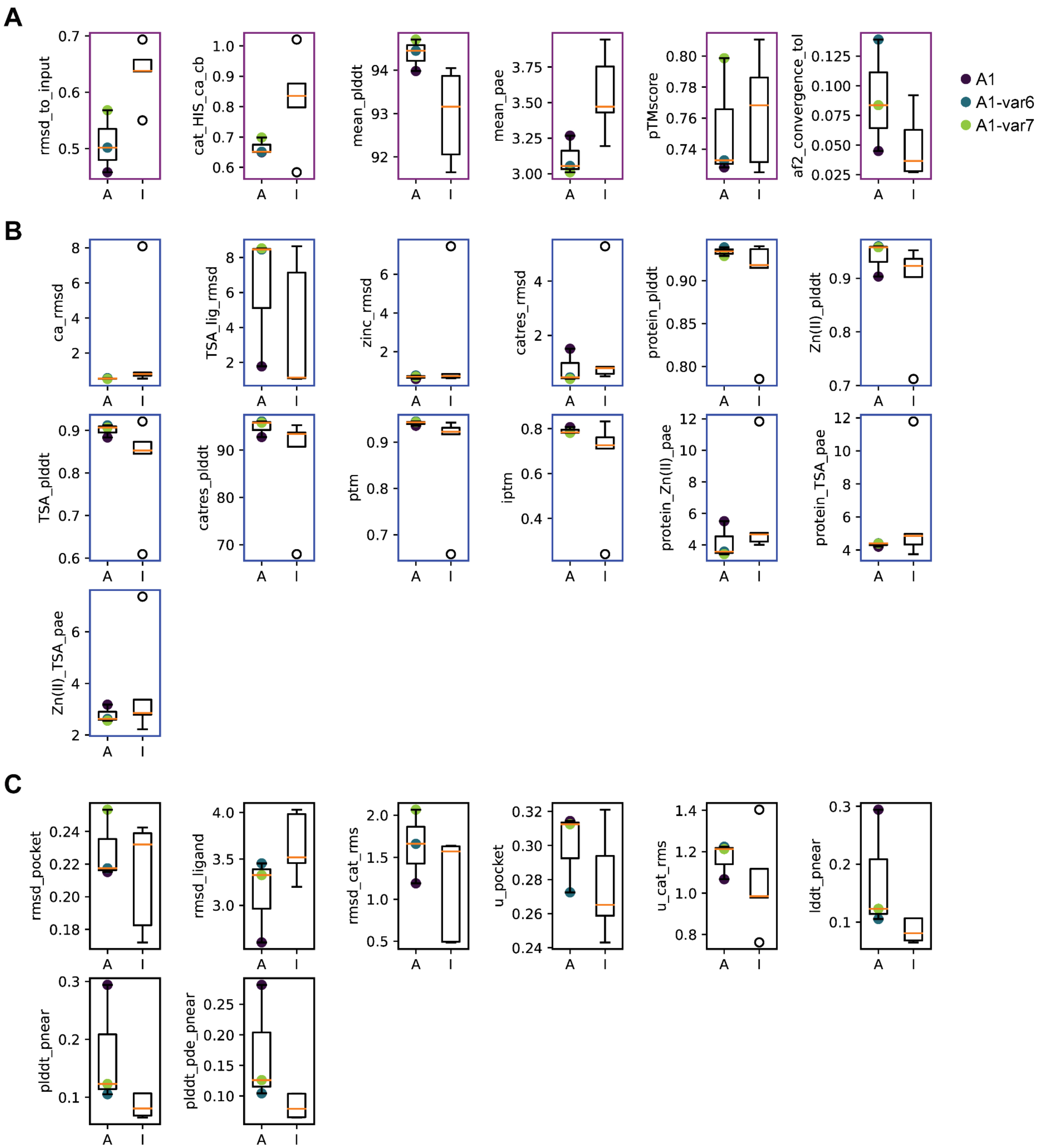

Fig. S23. AF2/Chai-1/PLACER metric distributions for ZETA_1 (A1) variants. (**A**) AlphaFold2, (**B**) Chai-1, and (**C**) PLACER metric distributions. The 7 variants + ZETA_1 are categorized as A = active or I = inactive for the box plots. Only variants 6 and 7 were additionally considered active in this analysis, although it should be noted that variants 4 and 5 have minor activity. In the active box plot, ZETA_1 is colored purple, variant 6 is colored blue, and variant 7 is colored lime green.

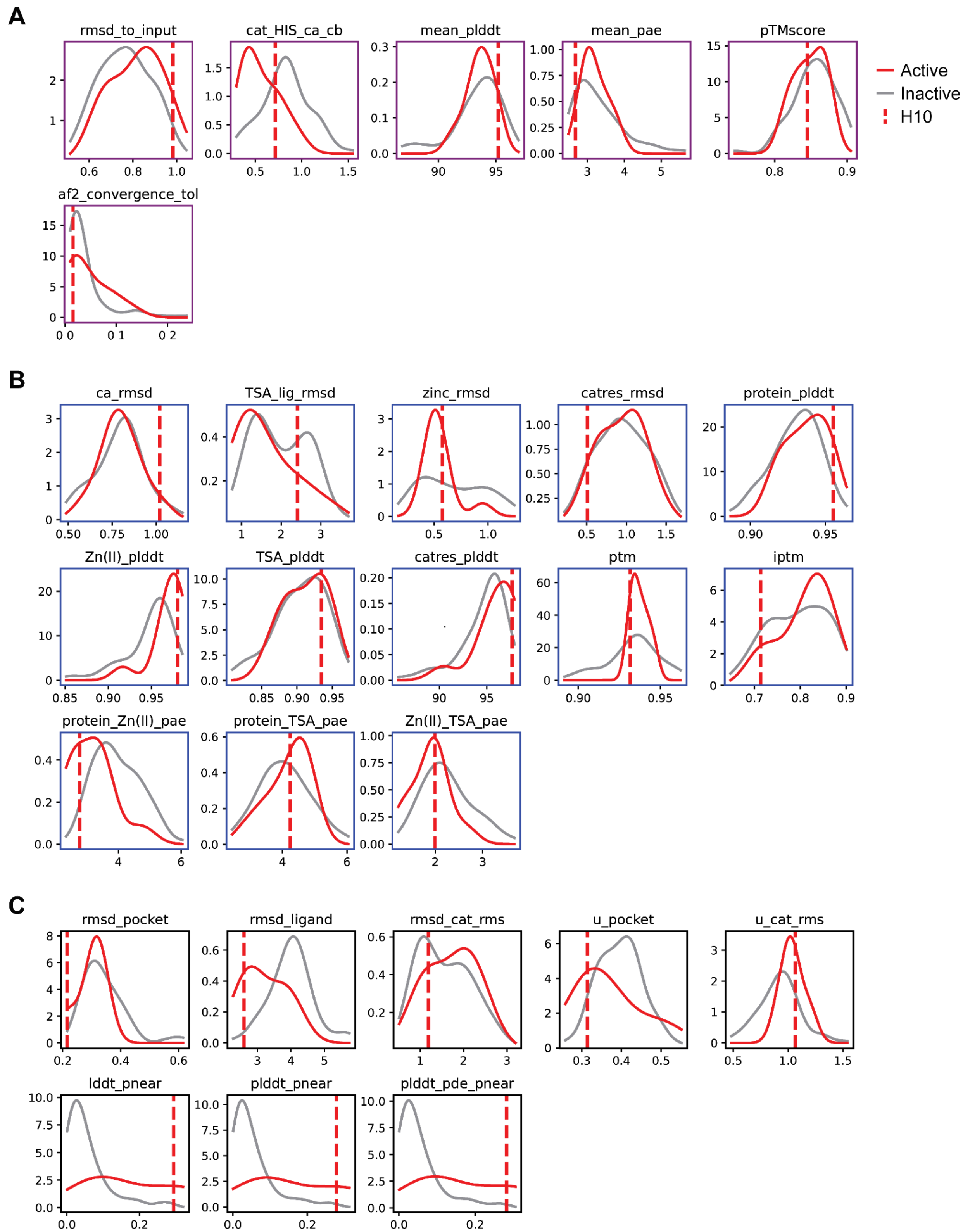

Fig. S24. AF2/Chai-1/PLACER metric distributions for second design campaign. (**A**) AlphaFold2, (**B**) Chai-1, and (**C**) PLACER metric distributions. Gray line is the distribution for the 85 inactive designs. Red solid line is the distribution for the 11 highly active designs spanning 3 unique scaffolds. Red dashed line is ZETA_2, the best enzyme out of both design campaigns.

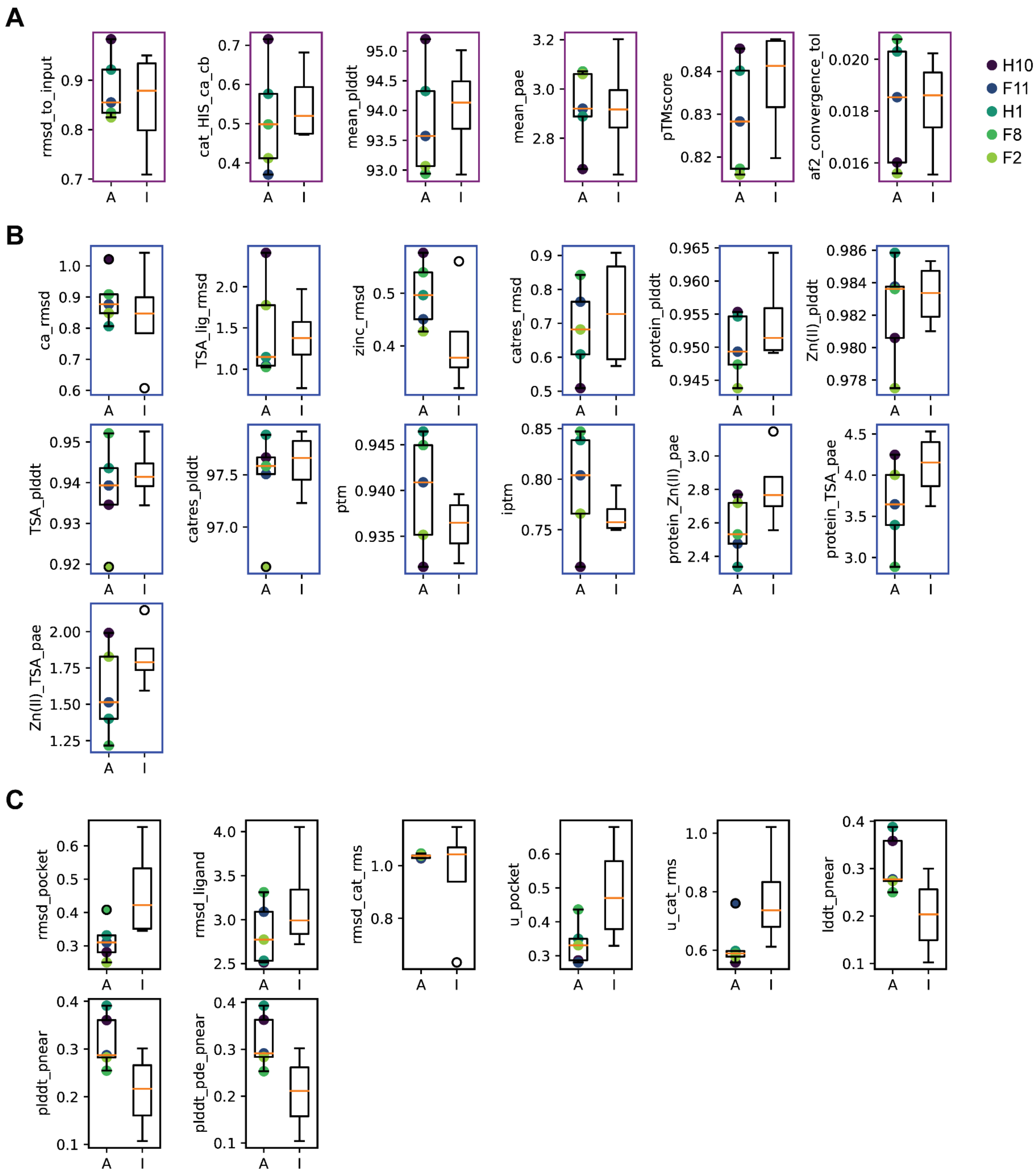

Fig. S25. AF2/Chai-1/PLACER metric distributions for ZETA_2 (H10) variants. There were 8 other designs (called variants here) from the ZETA_2 scaffold in the second design campaign order of 96 designs (9 total). (**A**) AlphaFold2, (**B**) Chai-1, and (**C**) PLACER metric distributions. The 8 variants + ZETA_2 are categorized as A = active or I = inactive for the box plots. Only H10 (ZETA_2), F11, H1, F8, and F2 were considered active in this analysis, although it should be noted that F7 and C8 also had moderate activity in the initial screen. In the active box plot, The dot coloring of the active designs is shown in the upper right corner.

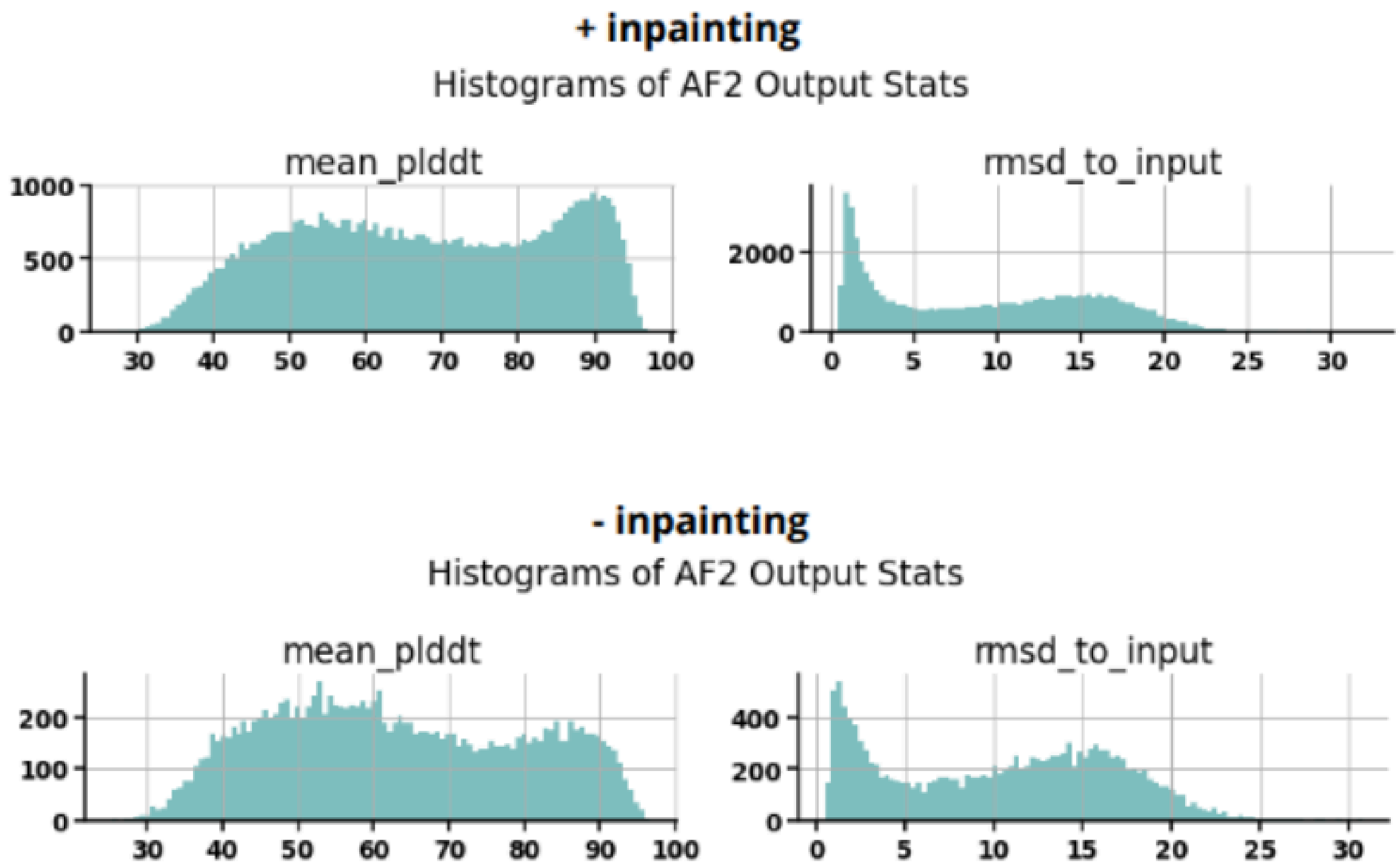

Fig. S26. Histograms of AF2 metrics from the first design campaign comparing designs with and without RFjoint2 inpainting applied. Inpainting with RFjoint2 locally diversifies scaffolds, mostly modifying loop structures and lengths (see section 4.1). The top graphs are the histograms of the mean AF2 pLDDT (left) and the AF2 prediction RMSD to the design (right) for designs from the first campaign which had RFjoint2 inpainting applied. The bottom graphs display the same metrics but for designs from the first design campaign which did not have RFjoint2 inpainting applied. Each non-inpainted structure from RFdiffusion2 had several inpainted variants, hence the y-axis absolute scale difference for the counts (i.e., less non-inpainted structures than inpainted ones). The distributions suggest that adding inpainting in the pipeline improves mean AF2 pLDDT and the AF2 prediction RMSD to the design, on average.

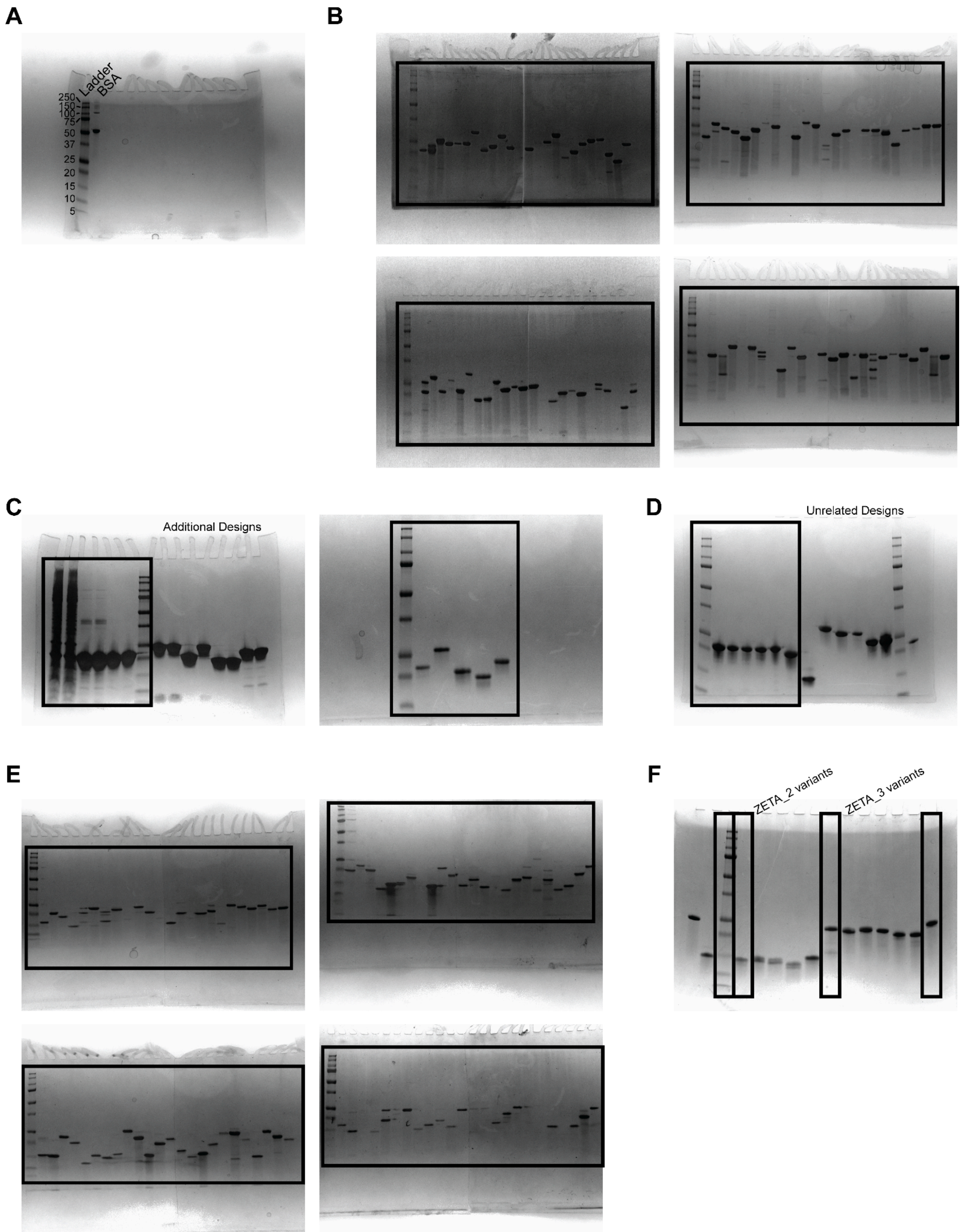

Figure S27. Uncropped gel images used in manuscript. (**A**) Loading control with ladder and BSA. Molecular masses of the ladder standards labeled with kDa units. Gel images with croppings in (**B**) Fig. S4, (**C**) Fig. S6, (**D**) Fig. S12, (**E**) Fig. S18, and (**F**) Fig. S20.

6. SUPPLEMENTAL TABLES

Table S1. Electrospray ionization mass spectrometry results for the characterized hits and A1 mutants.

Design	(E) Expected Mass (Da)	(O) Observed Mass (Da)	Δ (O - E)
**1st Design Campaign**			
A1 (ZETA_1)	18528	18397	-131
A8	25301	25170	-131
B9	20202	20071	-131
C4	18724	18593	-131
F7	21023	20892	-131
**A1 (ZETA_1) Knockout Mutants**			
A1 H118A;H130A;H134A	18330	18199	-131
A1 H130A	18462	18331	-131
A1 H134A	18462	18331	-131
A1 H118A	18462	18331	-131
A1 N17A	18485	18354	-131
A1 D67A	18484	18353	-131
**2nd Design Campaign**			
H10 (ZETA_2)	15748	15616	-132
H6 (ZETA_3)	22046	21915	-131
C5 (ZETA_4)	22585	22454	-131

Note 1: In all cases the observed mass corresponded to the loss of N-terminal methionine (-131 Da).
Note 2: The mass spectrometer has an error of approximately ±1 Da.

Table S2. Comparing the catalytic efficiencies of different designed and engineered (metallo)enzymes.

	Substrate*	pH	k_{cat} (s^{-1})	k_{cat}/K_M (M^{-1} s^{-1})	k_{cat}/k_{uncat}	Ref.
ZETA_1	4MU-PA	8.0	0.51	1.6×10^4	2.4×10^4	
ZETA_2	4MU-PA	8.0	1.5	5.3×10^4	7.3×10^4	
ZETA_3	4MU-PA	8.0	0.31	1.9×10^4	1.5×10^4	
ZETA_4	4MU-PA	8.0	0.081	1.1×10^3	3.9×10^3	
**Computationally Designed Zinc Esterases (from scratch with novel scaffolds; no laboratory evolution)**						
MID1[§,‖]	*p*NPA	8.0	0.13	40	9.4×10^3	24
**Computationally Designed/Engineered Zinc Esterases (from existing scaffolds)**						
$(TRIL9CL23H)_3$ [‖]	*p*NPA	8.0	0.0054	3.1	3.4×10^2	6
^{A104}AB3 [§,‖]	*p*NPA	9.0	0.0068	8	6.2×10^1	11
Ac-IHIHIQI-$CONH_2$ [‖]	*p*NPA	8.0	0.026	62	1.6×10^3	10
**Computationally Designed Esterases (from scratch with novel scaffolds; no laboratory evolution)**						
Win11 (1x redesign)	4MU-Ac	7.4	0.0197	3.9×10^3	1.5×10^3	1
momi	4MU-PA	7.4	0.1	1.2×10^3	7.7×10^3	1
momi120-103 (2x redesign)	4MU-PA	7.4	0.057	2.2×10^5	4.4×10^3	1
**Computationally Designed/Engineered Esterases (from existing scaffolds)**						
FR29 [‖,**]	*p*NPP	7.5	0.0041	34	5.5×10^2	83
ECH13 [‖,**]	*p*NPP	7.5	0.018	309	2.4×10^3	83
ECH19 [‖,**]	*p*NPP	7.5	0.01	227	1.4×10^3	83
AlleyCatE [‖]	(*R*)-*p*NPP	7.5	0.004	5.5	5.4×10^2	84
CC-Hept-Cys-His-Glu [‖,**]	*p*NPA	7.0	0.0005	3.7	1.4×10^2	85
PZD2 [‖]	*p*NPA	7.0	0.00046	2.7	1.3×10^2	86
**Engineered Esterases (from combinatorial libraries of peptides)**						
S-824 [‖]	*p*NPA	7.3	0.0055	1.8	7.4×10^2	87
**Computationally Designed/Engineered Non-Hydrolytic Enzymes (from scratch)**						
G_4-DF_{tet} [†]	4AP	7.0	0.021	25	—	88
RAD29 [†]	Methodol	7.5	0.0314	290	5×10^6	27
**Computationally Designed Non-Hydrolytic Enzymes (from existing scaffolds; no laboratory evolution)**						
DA_20_00 [†]	CBBC/DMAA	7.4	0.000028	0.06 [‡]	4.1 [‡]	3
BH32 [†]	F2PP	—	0.022	700	—	89
LuxSit [†]	DTZ	8.0	~0.025	~1.4×10^3	—	4
RA95 [†]	Methodol	7.5	0.000033	0.053	4.8×10^3	90
cRA-50 [†]	Methodol	—	0.037	180	—	12
dnHEM1 [†]	Amplex Red	7.2	9.5	260	—	91
HG3 [†]	5-NBZ	7.25	0.68	425	5.9×10^5	92
KE70 [†]	5-NBZ	7.25	0.16	80	1.4×10^5	5
**Computational Sequence-Redesigned/-Optimized Enzymes (from existing active enzymes)**						
HG259 [†]	5-NBZ	7.0	—	13×10^4	—	93
HG630 [†]	5-NBZ	7.0	—	15×10^4	—	93
HG649 [†]	5-NBZ	7.0	—	32×10^4	—	93
KE703b [†]	5-NBZ	7.0	—	5.5×10^3	—	93
$KGSADH_{V18C}$ [†]	3-HPA	7.4	7.0	4.0×10^3	—	94
**Catalytic Antibodies**						
43C9 [‖]	*p*NP-ester	9.3	25	4.7×10^5	2.7×10^4	95
D2.4 [‖]	*p*NP-ester	8.3	0.22	9.4×10^3	5.4×10^3	96
CNJ157 [‖]	*p*NP-ester	8.0	0.04	360	2.5×10^3	97
48G7 [‖]	*p*NP-ester	8.2	0.092	230	5.8×10^3	98
CNJ206 [‖]	*p*NPA	8.0	0.0067	83	4.2×10^2	99
**Natural Enzymes**						
S. solfataricus lipase [††]	*p*NPB	6.0	1100	8.8×10^6	—	100
C. albicans lipase [‖]	*p*NPB	6.0	54	1.3×10^5	1.7×10^7	101
hCA II [‖]	*p*NPA	8.0	53	2.5×10^3	3.3×10^6	44
Staph. xylosus lipase [††]	*p*NPB	9.0	0.061	140	—	102

Note 1: Table adapted from previous tables.[24,85]

Note 2: Catalytic antibodies and native metallohydrolases are included for reference.

Note 3: According to the sorting in this table, ZETA_1-4 would be classified under `Computationally Designed Zinc Esterases (from scratch with novel scaffolds; no laboratory evolution)`

* Substrate abbreviation. 4MU-PA: 4-Methylumbelliferyl phenylacetate; 4MU-Ac: 4-Methylumbelliferyl acetate; *p*NPA: *p*-nitrophenylacetate; *p*NPB: *p*-nitrophenylbutyrate; *p*NPP: *p*-nitrophenyl-2-propanoate; *p*NP-ester: 5-((4-(2-(4-nitrophenoxy)-2-oxoethyl)phenyl)amino)-5-oxopentanoic acid (43C9), p-nitrophenyl-N-glycylglutarate (D2.4), 4-(5-(4-nitrophenoxy)-5-oxopentanamido)butanoic acid (CNJ157), 6-(4-nitrophenoxy)-6-oxohexanoic acid (48G7). 5-NBZ: 5-nitro-1,2-benzisoxazole; DTZ: diphenylterazine; CBBC/DMAA: 4-carboxybenzyl trans-1,3-butadiene-1-carbamate and N,N-dimethylacrylamide; F2PP: fluorescein 2-phenylpropanoate; 4AP: 4-aminophenol; 3-HPA: 3-hydroxypropionaldehyde

** Only the catalytic parameters for enzyme acylation were reported; the deacylation step is considerably slower and limits overall turnover efficiency.

† Non-esterase enzymes. G_4-DF_{tet}: diiron O_2-dependent phenol oxidase; DA_20_00: Diels-Alderase; BH32: Morita-Baylis-Hillman reaction enzyme catalyst; LuxSit: luciferase; RA95, cRA-50, RAD29: retroaldolase; dnHEM1: heme peroxidase; HG series, KE series: kemp eliminase; $KGSADH_{Y68T}$: alpha-ketoglutaric semialdehyde dehydrogenase.

‖ Rate enhancement (k_{cat}/k_{uncat}) was calculated using k_{buffer} for *p*NPA hydrolysis from a previous measurement[7]: pH 9.0, 1.1×10^{-4} s^{-1}; pH 8.5, 4.1×10^{-5} s^{-1}; pH 8.0, 1.6×10^{-5} s^{-1}; pH 7.5, 7.4×10^{-6} s^{-1}; pH 7.0, 3.5 × 10-6 s^{-1}; pH 6, 2.9×10^{-6} s^{-1} (for pH 6.0, relative rate constant was abstracted from another previous measurement[103]).

‡ $k_{cat}/K_{M\text{-diene}}\ K_{M\text{-dienophile}}$ for the diels-alderase; k_{cat}/k_{uncat} has units of M in this instance.

~ Estimated values for (1) LuxSit since only the K_M = 18.3 μM and V_{max} = 0.0032 photon s^{-1} molecule^{-1} were reported, where enzyme concentration nor luminescent quantum yield were reported to determine k_{cat}. The successful mutagenic variant LuxSit-i had V_{max} = 0.36 photon s^{-1} molecule^{-1} and k_{cat} = 2.5 s^{-1} reported; thus, we assumed the k_{cat} for LuxSit was also two orders of magnitude lower since its V_{max} was two orders of magnitude lower and V_{max} is proportional to k_{cat}.

§ Normalized to a single zinc site.

†† Kinetic parameters determined at elevated temperatures: 70 °C for *S. solfataricus* lipase; 42 °C for *Staph. xylosus* lipase

Table S3. Pocket metric statistics from the first design campaign.

Measurement	Design					
	**A1 (ZETA_1)**	**A8**	**B9**	**C4**	**F7**	**Inactive**
Substrate Relative SASA	2.4	54	6.2	12	3.4	11 ± 14
Contact Molecular Surface	255	229	250	273	250	248 ± 19
Pocket Volume ($Å^3$)	1316	1337	2500	1108	1008	1294 ± 616
Pocket Hydrophobicity	38	35	30	34	27	28 ± 12

Table S4. Expressed amino acid sequences for the top 5 characterized designs from the first design campaign.

Design	Expressed Sequence (MSG - Design - GSA - WSHPQFEK)	Total Length
A1 (ZETA_1)	**MSG**LSREEIVERAVEAAKINSKLNPKYKKLYEAGAAAAKFTSENGEEYKETLKKENPELYEDALIVKKADDETEAAAKAAKGKTPEEQLEEFLKVALKFLPVFKEKFGITEEEARELARVHTEYFLETGDPDHLKSHIWRTEKILKKLKEE**GSAWSHPQFEK**	162 AAs
A8	**MSG**KLEELAKKIEELAKKYGFTDKEKLLKSYEIGKETGEKLKKFLEKLGVPKEKAEKYGKATRFHILLAGINFGSVEAAKILGEAILKTIEELEKLDPSKLPLSRILKIIALSGERKTFELVAQYIKEKGLESFTYDHPNKDGTFTSEEFTYEEAKKKVEEYDKEIEKLLKLPDSKLDKEELEKFEKEFDEHAEANELFAEAAEIYLKK**GSAWSHPQFEK**	220 AAs
B9	**MSG**DLEELLAVGKSPNAFHNPEVASKTFPLEDVEKVREIHGTTVSLIKEGVPKKLSYRFLGAFLKSELPEDVAQAALDAELTALPSVLKLLGFSQEEIDELVSALREGYENAEEVLKELERLISDPSTAELAEKIRKGLTPENIKKGVEEHEKLNKEVVKKAKELLKKKNS**GSAWSHPQFEK**	182 AAs
C4	**MSG**LDPEELLALSERALGSPDISAEELAELTETVLRAHAEYAERHPELRVLVDAHIRSSAEGVEADPIAFPLAHAFPTGDLAEGARHEAEGLRVLSLPPEEYRRVFEGHIASLPPEVRARTERLIAEGYRLDEEERERHIRIIVEGVQRAGRVLEAR**GSAWSHPQFEK**	168 AAs
F7	**MSG**TKKIYVYISDSVPKSAFDVFVRYLKAAGKEPVELVYIEEEEIEEAAEKPNFLISVGYENADYAIKLAKEKGVVSEVSVGYTEEEKLKILTTVKESGLKAYLVESYKPYLEPYIKKAGLDNVEWIPAKEHPEVVYDKVISGEPAIFIHHGDPELHQKVLEEIEEKIEELENK**GSAWSHPQFEK**	185 AAs

Note 1: The N- and C-terminal residues are introduced by cloning, highlighted in bold.

Table S5. Expressed amino acid sequences for the top 11 characterized designs from the second design campaign.

Design	Expressed Sequence (MSG - Design - GSA - WSHPQFEK)	Total Length
H10 (ZETA_2)	**MSG**ELTPLQQAALKWARKLAERFPELGEEFIAVHLEEARFWEKAGATP EEVDAAGKATLEYYEAIRNGDEEKAVEARKKALDIYNKIVEALKKQPPE VVAAYEAFRPRHEALHRRAEATLRAQYEAR**GSAWSHPQFEK**	138 AAs
F11	**MSG**ELTPLQQAALRWARKLAERFPELGEEFIEVHLEEARFWEKAGATA EEVDEAGRATLEYYEALRNGDEAAAEKARKKALDIYRKVEEALKKQPP EVVKEYEAFAPRHRALHVRGEATLRANYEAR**GSAWSHPQFEK**	138 AAs
H1	**MSG**ELTPLQQAALKWARKLAERFPELGEEFVEVHLEEARFWEKAGATA EEVDEAGKATLEYYEAIRNGDEEKAKVAREKALAIYRKIVERLAKQPPE VVAEYEAFRPRHERLHEEAERTLRALYEAR**GSAWSHPQFEK**	138 AAs
F8	**MSG**ELTPLQKAALEWARKLAERFPELGEEFVEIHIEEARFWEKAGATPE EVREAGRATLEYYEAIRNGDEEAAEKARKKALDIYLKVVEALAKQPPEV VAAYEAFRPRHEELHRRAEETLRALYEAR**GSAWSHPQFEK**	138 AAs
F2	**MSG**ELTPLQKAALEWAKKLAERFPELGEEFIEVHIEEARFWEKAGATPE EVREAGKATLEYYEAIRNGDEAAAEVARKKALDIYNKVVEALKKQPPEV VAEYEAFRPRHEELHRRAEETLRALYEAR**GSAWSHPQFEK**	138 AAs
H6 (ZETA_3)	**MSG**EAERRKEEVRRAYDVALAEEKKIQAKPEFLQHKDESERRRKELEA LSVRERAEEAVRKHTRFAAARFGDDYPKLTVDRFVESILAEARERAEKL KETEEEKRLYVRFFEEVARVLREEFRDVIEREIEELKGLSPEERAERFAE AREEHLKLPHELYFEAFLELVLARLD**GSAWSHPQFEK**	184 AAs
B10	**MSG**EAEEEKERARRAYDVALGVERKREARPEHLQHAAKAEEERERLA ALSTEERLAYAVELHTTVFLADFGDDYPAATVDSVIKRILDRAREEAERL EETPEEKELYVYFFERLAEVLRDEYRDLIEREIERLKALTPEERAARFEE ARERHLYVPHELLFEAALERILARLD**GSAWSHPQFEK**	184 AAs
B5	**MSG**EAEEEKERARRAYDVALGVERKREARPEHLQHREKAEEERERLS KLSTEERLGYAVELHTVVFLSGFGDDYPAATVDSVIKAILDHAREEAERL EETPEEKELYVYFFERLAEVLRDDYRDLIEREIERLKALTPEERRARFEE ARERHLYVPHELLFEAALERILARLD**GSAWSHPQFEK**	184 AAs
D9	**MSG**EAEEEKERARRAYDVALAVERKREARPEHLQHRAKAEEERERLA KLSTEERLGYAVELHTVVFLSEFGDDYPKATVDSVIKKILDIAREEAERL EETPEEKELYVYFFERLSEVLRDEYRDLIEREIERLKALTPEERKARFEE AHERHLYVPHELLFDAALERILARLD**GSAWSHPQFEK**	184 AAs
F12	**MSG**EAEERKREVREAREVAVLFERRVRSRPEYQRYAERSVEERERLE KLTTDERAREAVRLHTAIFAKTFGDRYDELTVESFIEKLLETARELAEREL DTEREKELYVRYHEEIARIIRDVFRDDVERVIDELKALSPEERAKRFEEA ELRHLYVPHELYFEAFEEYILGSLD**GSAWSHPQFEK**	184 AAs
C5 (ZETA_4)	**MSG**KHREVHERFLERAREAARRGPEYREEIVEWYVAEHKKVLDEEYE KGRKIYEELLKELSEKEAIEKLIERTRRLLEELRAKVDEAIAAGRPEEAVR YLEEYAEEIGNIFAFALVQQLTPEQQDEVAAEIARVLYDAVVEEVRELEEL PPEQAEAVRVRHLVKATEYVGEKTIELLEKLK**GSAWSHPQFEK**	192 AAs

Note 1: The N- and C-terminal residues are introduced by cloning, highlighted in bold.

7. SUPPLEMENTAL VIDEOS

Video S1. RFdiffusion2 trajectory for ZETA_1 (A1).

A1_RFdiffusion2_video.mp4

Video S2. PLACER predictions for substrate ensemble of ZETA_1 (A1).

A1_PLACER_subst.mp4

Video S3. PLACER predictions for catalytic residue ensemble of ZETA_1 (A1).

A1_PLACER_catres.mp4

Video S4. PLACER predictions for substrate ensemble of the inactive design H8.

H8_PLACER_subst.mp4

Video S5. PLACER predictions for catalytic residue ensemble of the inactive design H7.

H7_PLACER_pocket.mp4

8. SUPPLEMENTAL DATA

Data S1. Coordinates for all transition state models calculated by density functional theory.

Data S2. Protein and DNA sequences for all ordered designs.

Data S3. PDB design models for all ordered designs.

9. REFERENCES

48. Frisch, M. J. *et al. Gaussian 16 Rev. C.01*. (Wallingford, CT, 2016).

49. Grimme, S., Ehrlich, S. & Goerigk, L. Effect of the damping function in dispersion corrected density functional theory. *J. Comput. Chem.* **32**, 1456–1465 (2011).

50. Bannwarth, C. *et al.* Extended tight‐binding quantum chemistry methods. *Wiley Interdiscip. Rev. Comput. Mol. Sci.* **11**, (2021).

51. Kalvet, I. *Frozen-Conf-Xtb: A Python Program That Performs Frozen Coordinate Conformational Search Using GFN2-XTB Energies (https://github.com/ikalvet/frozen-Conf-Xtb)*. (Github).

52. Kalvet, I. *Invrotzyme: Utility for Building Inverse Rotamer Assemblies out of a Rosetta Matcher/enzdes Constraint File (https://github.com/ikalvet/invrotzyme)*. (Github).

53. Kim, D. E. *et al.* Parametrically guided design of beta barrels and transmembrane nanopores using deep learning. *Proc. Natl. Acad. Sci. U. S. A.* **122**, e2425459122 (2025).

54. Maguire, J. B. *et al.* Perturbing the energy landscape for improved packing during computational protein design. *Proteins* **89**, 436–449 (2021).

55. van Kempen, M. *et al.* Fast and accurate protein structure search with Foldseek. *Nat. Biotechnol.* **42**, 243–246 (2024).

56. Varadi, M. *et al.* AlphaFold Protein Structure Database: massively expanding the structural coverage of protein-sequence space with high-accuracy models. *Nucleic Acids Res.* **50**, D439–D444 (2022).

57. Lienhard, G. E. Enzymatic Catalysis and Transition-State Theory. *Science* **180**, 149–154 (1973).

58. Škrinjar, P., Eberhardt, J., Tauriello, G., Schwede, T. & Durairaj, J. Have protein-ligand cofolding methods moved beyond memorisation? *bioRxiv* 2025.02.03.636309 (2025).

59. Dive, V. *et al.* Phosphinic peptides as zinc metalloproteinase inhibitors. *Cell Mol Life Sci*

61, 2010–2019 (2004).

60. Grobelny, D., Goli, U. B. & Galardy, R. E. Binding energetics of phosphorus-containing inhibitors of thermolysin. *Biochemistry* **28**, 4948–4951 (1989).

61. Wicky, B. I. M. *et al.* Hallucinating symmetric protein assemblies. *Science* **378**, 56–61 (2022).

62. Schmidt, T. G. M. & Skerra, A. The Strep-tag system for one-step purification and high-affinity detection or capturing of proteins. *Nat. Protoc.* **2**, 1528–1535 (2007).

63. Barysaitė, S., Chmeliov, J., Valkunas, L. & Gelzinis, A. Concentration quenching of fluorescence decay kinetics of molecular systems. *J. Phys. Chem. B* **128**, 4887–4897 (2024).

64. Watson, W. F. & Livingston, R. Self-quenching and sensitization of fluorescence of chlorophyll solutions. *J. Chem. Phys.* **18**, 802–809 (1950).

65. Albani, J. R. Fluorescence Quenching. in *Structure and Dynamics of Macromolecules: Absorption and Fluorescence Studies* 141–192 (Elsevier, 2004).

66. 2.3.6.1. Models for instrument calibration. https://www.itl.nist.gov/div898/handbook/mpc/section3/mpc361.htm.

67. Herman, R. A., Scherer, P. N. & Shan, G. Evaluation of logistic and polynomial models for fitting sandwich-ELISA calibration curves. *J. Immunol. Methods* **339**, 245–258 (2008).

68. Chen, H.-Y. & Chen, C. Evaluation of calibration equations by using regression analysis: An example of chemical analysis. *Sensors (Basel)* **22**, 447 (2022).

69. Jarantow, S. W., Pisors, E. D. & Chiu, M. L. Introduction to the use of linear and nonlinear regression analysis in quantitative biological assays. *Curr. Protoc.* **3**, e801 (2023).

70. Wieczorek, M. *et al.* Reliable calibration by nonlinear standard addition method in the presence of additive interference effects. *Monatsh. Chem.* **149**, 1567–1572 (2018).

71. Vallee, B. L., Rupley, J. A., Coombs, T. L. & Neurath, H. The role of zinc in

carboxypeptidase. *J. Biol. Chem.* **235**, 64–69 (1960).

72. Vallee, B. L. & Coombs, T. L. Complex formation of 1,10-phenanthroline with zinc ions and the zinc of alcohol dehydrogenase of horse liver. *J. Biol. Chem.* **234**, 2615–2620 (1959).

73. Fatema, K., Lund, E. & Petering, D. H. Zinc trafficking: 1,10-phenanthroline, glutathione, and other metal binding ligands form adducts with proteomic Zn2. *Metallomics* **15**, mfad026 (2023).

74. Boiwe, T. & Bränden, C. I. X-ray investigation of the binding of 1,10-phenanthroline and imidazole to horse-liver alcohol dehydrogenase. *Eur. J. Biochem.* **77**, 173–179 (1977).

75. Simons, T. J. Measurement of free Zn2+ ion concentration with the fluorescent probe mag-fura-2 (furaptra). *J. Biochem. Biophys. Methods* **27**, 25–37 (1993).

76. Sumida, K. H. *et al.* Improving Protein Expression, Stability, and Function with ProteinMPNN. *J. Am. Chem. Soc.* **146**, 2054–2061 (2024).

77. Kabsch, W. Integration, scaling, space-group assignment and post-refinement. *Acta Crystallogr D* **66**, 133–144 (2010).

78. Winn, M. D. *et al.* Overview of the CCP4 suite and current developments. *Acta Crystallogr D Biol Crystallogr* **67**, 235-242 (2011).

79. McCoy, A. J. *et al.* ıt Phaser crystallographic software. *Journal of Applied Crystallography* **40**, 658-674 (2007).

80. Adams, P. D. *et al.* PHENIX: a comprehensive Python-based system for macromolecular structure solution. *Acta Crystallogr D Biol Crystallogr* **66**, 213-221 (2010).

81. Emsley, P. & Cowtan, K. Coot: model-building tools for molecular graphics. *Acta Crystallogr D Biol Crystallogr* **60**, 2126-2132 (2004).

82. Williams, C. J. *et al.* MolProbity: More and better reference data for improved all-atom structure validation. *Protein Sci* **27**, 293-315 (2018).

83. Richter, F. *et al.* Computational design of catalytic dyads and oxyanion holes for ester hydrolysis. *J. Am. Chem. Soc.* **134**, 16197–16206 (2012).

84. Moroz, Y. S. *et al.* New tricks for old proteins: Single mutations in a nonenzymatic protein give rise to various enzymatic activities. *J. Am. Chem. Soc.* **137**, 14905–14911 (2015).
85. Burton, A. J., Thomson, A. R., Dawson, W. M., Brady, R. L. & Woolfson, D. N. Installing hydrolytic activity into a completely de novo protein framework. *Nat. Chem.* **8**, 837–844 (2016).
86. Bolon, D. N. & Mayo, S. L. Enzyme-like proteins by computational design. *Proc. Natl. Acad. Sci. U. S. A.* **98**, 14274–14279 (2001).
87. Wei, Y. & Hecht, M. H. Enzyme-like proteins from an unselected library of designed amino acid sequences. *Protein Eng. Des. Sel.* **17**, 67–75 (2004).
88. Kaplan, J. & DeGrado, W. F. De novo design of catalytic proteins. *Proc. Natl. Acad. Sci. U. S. A.* **101**, 11566–11570 (2004).
89. Burke, A. J. *et al.* Design and evolution of an enzyme with a non-canonical organocatalytic mechanism. *Nature* **570**, 219–223 (2019).
90. Althoff, E. A. *et al.* Robust design and optimization of retroaldol enzymes: Robust Design and Optimization of Retroaldol Enzymes. *Protein Sci.* **21**, 717–726 (2012).
91. Kalvet, I. *et al.* Design of Heme Enzymes with a Tunable Substrate Binding Pocket Adjacent to an Open Metal Coordination Site. *J. Am. Chem. Soc.* **145**, 14307–14315 (2023).
92. Privett, H. K. *et al.* Iterative approach to computational enzyme design. *Proc. Natl. Acad. Sci. U. S. A.* **109**, 3790–3795 (2012).
93. Rakotoharisoa, R. V. *et al.* Design of efficient artificial enzymes using crystallographically enhanced conformational sampling. *J. Am. Chem. Soc.* **146**, 10001–10013 (2024).
94. Kim, D. *et al.* Enzyme activity engineering based on sequence co-evolution analysis. *Metab. Eng.* **74**, 49–60 (2022).
95. Stewart, J. D. *et al.* Dissection of an antibody-catalyzed reaction. *Proc. Natl. Acad. Sci. U. S. A.* **91**, 7404–7409 (1994).

96. Tawfik, D. S., Lindner, A. B., Chap, R., Eshhar, Z. & Green, B. S. Efficient and selective p-nitrophenyl-ester-hydrolyzing antibodies elicited by a p-nitrobenzyl phosphonate hapten. *Eur. J. Biochem.* **244**, 619–626 (1997).

97. Tawfik, D. S., Green, B. S. & Eshhar, Z. Detection of catalytic monoclonal antibodies. *Anal. Biochem.* **202**, 35–39 (1992).

98. Patten, P. A. *et al.* The immunological evolution of catalysis. *Science* **271**, 1086–1091 (1996).

99. Charbonnier, J. B. *et al.* Crystal structure of the complex of a catalytic antibody Fab fragment with a transition state analog: structural similarities in esterase-like catalytic antibodies. *Proc. Natl. Acad. Sci. U. S. A.* **92**, 11721–11725 (1995).

100. Choi, Y.-H., Park, Y.-J., Yoon, S.-J. & Lee, H.-B. Purification and characterization of a new inducible thermostable extracellular lipolytic enzyme from the thermoacidophilic archaeon Sulfolobus solfataricus P1. *J. Mol. Catal. B Enzym.* **124**, 11–19 (2016).

101. Lan, D.-M. *et al.* A novel cold-active lipase from Candida albicans: cloning, expression and characterization of the recombinant enzyme. *Int. J. Mol. Sci.* **12**, 3950–3965 (2011).

102. Kolling, D. J. *et al.* Biochemical and structural characterization of two site-directed mutants of Staphylococcus xylosus lipase. *Mol. Biotechnol.* **46**, 168–175 (2010).

103. Goren, H. J. & Fridkin, M. The hydrolysis of p-nitrophenylacetate in water. Mechanism and method of measurement. *Eur. J. Biochem.* **41**, 263–272 (1974).
