# Supplementary material for: Computational design of metallohydrolases
Source: Nature. 2025 Dec 3;649(8095):246–53. doi: 10.1038/s41586-025-09746-w (PMC12727532; doi:10.1038/s41586-025-09746-w)
Supplement: Supplementary file 3 [file 41586_2025_9746_MOESM2_ESM.pdf]

nature portfolio

Corresponding author(s): Indrek Kalvet, Donald Hilvert, David Baker

Last updated by author(s): Oct 8, 2025

Reporting Summary

Nature Portfolio wishes to improve the reproducibility of the work that we publish. This form provides structure for consistency and transparency in reporting. For further information on Nature Portfolio policies, see our Editorial Policies and the Editorial Policy Checklist.

Statistics

For all statistical analyses, confirm that the following items are present in the figure legend, table legend, main text, or Methods section.

n/a	Confirmed	
☐	☒	The exact sample size (*n*) for each experimental group/condition, given as a discrete number and unit of measurement
☐	☒	A statement on whether measurements were taken from distinct samples or whether the same sample was measured repeatedly
☐	☒	The statistical test(s) used AND whether they are one- or two-sided<br>*Only common tests should be described solely by name; describe more complex techniques in the Methods section.*
☒	☐	A description of all covariates tested
☒	☐	A description of any assumptions or corrections, such as tests of normality and adjustment for multiple comparisons
☐	☒	A full description of the statistical parameters including central tendency (e.g. means) or other basic estimates (e.g. regression coefficient) AND variation (e.g. standard deviation) or associated estimates of uncertainty (e.g. confidence intervals)
☐	☒	For null hypothesis testing, the test statistic (e.g. *F*, *t*, *r*) with confidence intervals, effect sizes, degrees of freedom and *P* value noted<br>*Give P values as exact values whenever suitable.*
☒	☐	For Bayesian analysis, information on the choice of priors and Markov chain Monte Carlo settings
☒	☐	For hierarchical and complex designs, identification of the appropriate level for tests and full reporting of outcomes
☒	☐	Estimates of effect sizes (e.g. Cohen's *d*, Pearson's *r*), indicating how they were calculated

Our web collection on statistics for biologists contains articles on many of the points above.

Software and code

Policy information about availability of computer code

Data collection

The Rosetta macromolecular modeling suite (https://rosettacommons.org/) is freely available to academic and non-commercial users. Commercial licenses for the suite are available through the University of Washington Technology Transfer Office.

All the data in this study was experimentally measured or computationally generated in the design pipeline created by the authors, which will be available open-source on GitHub upon publication of the manuscript. No external data was acquired.

Code for the computational design of metallohydrolases will be made available upon acceptance of the manuscript at the following repository:
https://github.com/baker-laboratory/Metallohydrolase_Enzyme_Design

The manuscript describing the training and benchmarking of RFdiffusion2 has now been posted in the bioRxiv (Ahern et al. bioRxiv 2025) and has been submitted to Nature Methods. Please refer to the link: https://www.biorxiv.org/content/10.1101/2025.04.09.648075v2. The associated code and pretrained neural network weights are available at:
https://github.com/RosettaCommons/RFdiffusion2

PLACER is publicly available on GitHub at the link: https://github.com/baker-laboratory/PLACER.

April 2023

Data analysis	The molecular mass of each protein was deconvoluted by Bioconfirm software (10.0) using a total entropy algorithm. Data were analyzed and plotted using Python 3.12; analysis was done using numpy 1.26.4 and pandas 2.2.2 and plotting was done using seaborn 0.13.2 and matplotlib 3.9.2.

For manuscripts utilizing custom algorithms or software that are central to the research but not yet described in published literature, software must be made available to editors and reviewers. We strongly encourage code deposition in a community repository (e.g. GitHub). See the Nature Portfolio guidelines for submitting code & software for further information.

Data

Policy information about availability of data

All manuscripts must include a data availability statement. This statement should provide the following information, where applicable:

- Accession codes, unique identifiers, or web links for publicly available datasets
- A description of any restrictions on data availability
- For clinical datasets or third party data, please ensure that the statement adheres to our policy

Source data for the figures will be made available online upon publication of this manuscript. Gene sequences will be deposited to GenBank upon publication of this manuscript. Design models are available for peer-review in the supplemental information and will be made publicly available upon publication of this manuscript. Codon-optimized plasmids that were used in the study will be made publicly available through Addgene upon publication of this manuscript.

Research involving human participants, their data, or biological material

Policy information about studies with human participants or human data. See also policy information about sex, gender (identity/presentation), and sexual orientation and race, ethnicity and racism.

Reporting on sex and gender	No research involving human participants was conducted in this study. Not applicable.
Reporting on race, ethnicity, or other socially relevant groupings	No research involving human participants was conducted in this study. Not applicable.
Population characteristics	No research involving human participants was conducted in this study. Not applicable.
Recruitment	No research involving human participants was conducted in this study. Not applicable.
Ethics oversight	No research involving human participants was conducted in this study. Not applicable.

Note that full information on the approval of the study protocol must also be provided in the manuscript.

Field-specific reporting

Please select the one below that is the best fit for your research. If you are not sure, read the appropriate sections before making your selection.

☒ Life sciences ☐ Behavioural & social sciences ☐ Ecological, evolutionary & environmental sciences

For a reference copy of the document with all sections, see nature.com/documents/nr-reporting-summary-flat.pdf

Life sciences study design

All studies must disclose on these points even when the disclosure is negative.

Sample size	No statistical methods were used to pre-determine the sample sizes in this study. The number of designs we ordered for screening (192) corresponds to the number of DNA fragments that can fit on a single commercial eBlock synthesized by IDT.
Data exclusions	No sample was excluded from the data analysis.
Replication	Measurements for protein concentration were performed in triplicate. All standard curves were measured in triplicate and used on the same day as experiments. Measurements of enzyme kinetics were done in triplicate and replicated with biological duplicates. During analysis of the purification protocol for the best enzyme designs (ZETA1-4), Coomassie-stained SDS PAGE gels were done in duplicate, which is reflected in a supplemental figure in the supplemental information. Zinc affinity measurements were done in duplicates. Circular dichroism measurements were performed in duplicates. Measurements of the lower bound for the total enzyme turnover were measured in triplicate. Size exclusion chromatography was at least repeated in biological duplicates.
Randomization	No randomization was used in this study.
Blinding	The author performing the zinc affinity measurements was blinded to the sample type. Researchers were not blinded for any other experiments.

Reporting for specific materials, systems and methods

We require information from authors about some types of materials, experimental systems and methods used in many studies. Here, indicate whether each material, system or method listed is relevant to your study. If you are not sure if a list item applies to your research, read the appropriate section before selecting a response.

Materials & experimental systems

n/a	Involved in the study
☒	☐ Antibodies
☒	☐ Eukaryotic cell lines
☒	☐ Palaeontology and archaeology
☒	☐ Animals and other organisms
☒	☐ Clinical data
☒	☐ Dual use research of concern
☒	☐ Plants

Methods

n/a	Involved in the study
☒	☐ ChIP-seq
☒	☐ Flow cytometry
☒	☐ MRI-based neuroimaging

Plants

Seed stocks	No research involving plants was conducted in this study. Not applicable.
Novel plant genotypes	No research involving plants was conducted in this study. Not applicable.
Authentication	No research involving plants was conducted in this study. Not applicable.
